# Supplementary material for: Unprotected peptide macrocyclization and stapling via a fluorine-thiol displacement reaction
Source: Nat Commun. 2022 Jan 17;13:350. doi: 10.1038/s41467-022-27995-5 (PMC8763920; doi:10.1038/s41467-022-27995-5)
Supplement: Supplementary file 1 — Supplementary Information [file 41467_2022_27995_MOESM1_ESM.pdf]

Supplementary Information

Unprotected Peptide Macrocyclization and Stapling via A Fluorine-Thiol Displacement Reaction

Md Shafiqul Islam[1], Samuel L. Junod[2], Si Zhang[1], Zakey Yusuf Buuh[1], Yifu Guan[1], Mi Zhao[1], Kishan H Kaneria[1], Parmila Kafley[1], Carson Cohen[1], Robert Maloney[1], Zhigang Lyu[1], Vincent A. Voelz[1], Weidong Yang[2], Rongsheng E. Wang[1,*]

1. Department of Chemistry, Temple University, 1901 N. 13th Street, Philadelphia, PA, 19122

2. Department of Biology, Temple University, 1900 N. 12th Street, Philadelphia, PA, 19122

* To whom correspondence should be addressed: Phone (215) 204-1855. Email: rosswang@temple.edu

Table of Contents

Supplementary Methods

Chemical Synthesis…… S2

Supplementary Discussion

Supplementary Tables…… S7

Experimental Figures…… S14

^{1}H- and ^{13}C- NMR Spectra for New Compounds…… S47

LC-Chromatogram and MS-Spectra for Peptides…… S55

Supplementary Methods:

Chemical Synthesis.

General Procedures. All chemicals and solvents used were purchased from either Fisher Scientific, Sigma Aldrich or VWR and were used directly without any further purification. All small molecules and building blocks were synthesized following traditional organic chemistry procedures. Regular phase flash column chromatography with manually loaded silica gel (grade 60, 230-400 mesh, Fisher Scientific) was used to purify synthesized compounds. High resolution ESI-MS was obtained at the Wistar Institute, using a ThermoFisher Scientific Q Exactive HF-X mass spectrometer coupled to a ThermoFisher Vanquish Horizon UHPLC system. NMR data for small molecules was recorded on a 500 MHz Bruker Advance with TMS as an internal standard. Peptides were synthesized on solid-phase using Fmoc chemistry. After cleavage from resins, the crude mixtures were precipitated out by diethyl ether, resuspended, and purified using Waters 1525 series preparative high-performance liquid chromatography (HPLC) loaded with the XBridge Prep C18 column (25 cm x 19 mm, particle size 5 µm). Peptide purity and mass spectra were recorded on the Agilent 1100 series liquid chromatography-mass spectrometry (LC-MS) that was equipped with an Ascentis® Express C8 analytical column (5 cm x 2.1 mm, particle size 2.7 µm). The LC-MS program ran with mobile phase A (0.1% TFA in water) and mobile phase B (0.1% TFA in acetonitrile). For each run, acetonitrile was linearly increased from 5% to 95% within 10 min.

Small Molecule Model Reactions. To a mixture of 20 µL of Tris buffer (3M) and 20 µL of DMF was added 10 µL of the stock DMF solution for the model compound **1** (640 mM). After 10 min of incubation at room temperature, 10 µL of methyl hydrazine, cysteine, or benzyl thiol (640 mM, DMF) together with 20 µL of water (for methyl hydrazine) or TCEP solution (2M) (for cysteine or benzyl thiol) were added. After adjusting its final pH to 9.0, the mixture was aliquoted equally into 4 sample vials (20 µL each), which were subsequently incubated at 37ºC. At the desired time point (0,4h, 8h, and 12h) of incubation, one vial each was collected to run LC-MS analysis of the sample. To determine the reaction progress, the UV peak areas (254 nm) of the model compound **1** before and after the 12h incubation were integrated and the relative yields of the reaction were calculated based on the decreased percentage of the peak areas for compound **1**.

Supplementary Figure 1.1: Small Molecule Model Reactions.

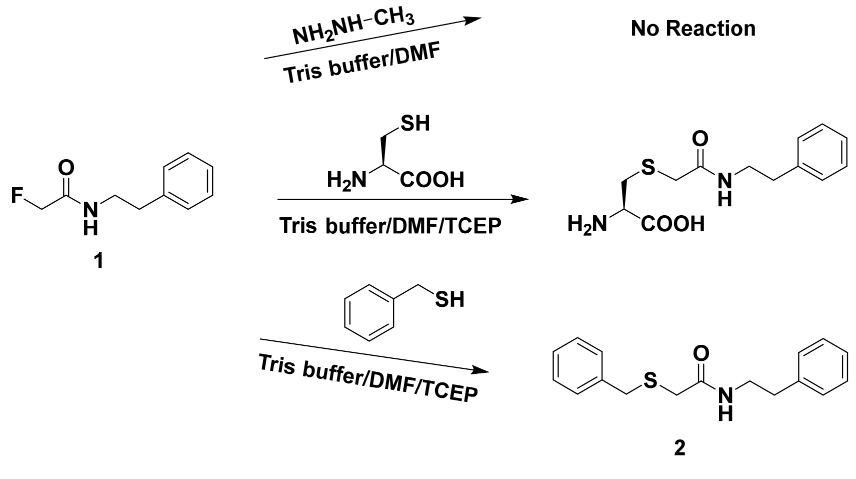

Building Block Synthesis.

Supplementary Figure 1.2: Synthesis of 2-(9H-Fluoren-9-ylmethoxycarbonylamino)-3-(2-fluoro-acetylamino)-propionic acids (compounds **15**/**16**).

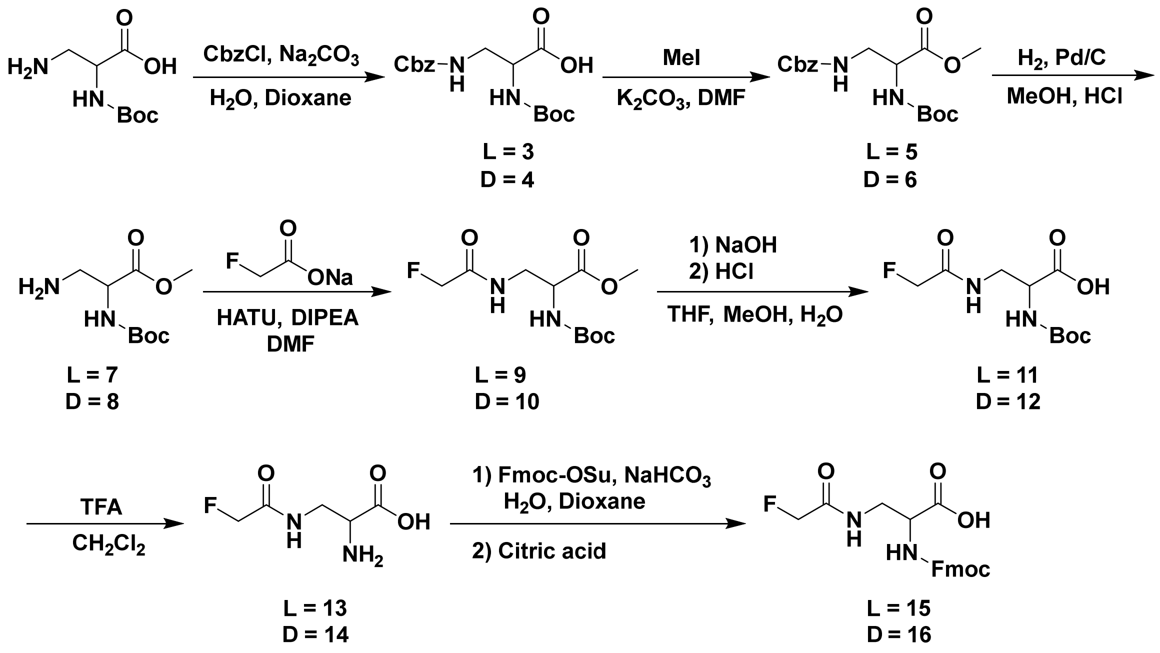

*Compound **1***. In a flame-dried 50 mL round bottom flask, sodium fluoroacetate (200 mg, 2 mmol) and HATU (912 mg, 2.4 mmol) were mixed with DIPEA (418μL, 2.4 mmol) in 15 mL anhydrous DMF. Around 10 min later, phenylethylamine (251 μL, 2 mmol) was added to the mixture. After overnight stirring, the reaction was quenched with water and the product was extracted with ethyl acetate. The collected organic layer was dried over anhydrous sodium sulfate, concentrated, and purified through silica gel column chromatography (hexane/ethyl acetate: 3/1) to afford the desired product as a white solid (277 mg, 1.52 mmol, 76% yield). ^{1}H NMR (500 MHz, $CDCl_3$): δ 7.34-7.31 (m, 2H), 7.26-7.20 (m, 3H), 4.76 (d, J = 47.5 Hz, 2H), 3.60 (q, J = 6.5 Hz, 2H), 2.86 (t, J = 7.5 Hz, 2H); ^{13}C NMR (126 MHz, $CDCl_3$): δ 167.6 (d, J = 17.1 Hz), 138.4, 128.8 (d, J = 5.0 Hz), 126.7, 80.2 (d, J = 186.1 Hz), 40.0, 35.6; ^{19}F NMR (471 MHz, $CDCl_3$): δ -224.78; HRMS (ESI) m/z calculated for $C_{10}H_{13}FNO$ $[M + H]^+$: 182.0976, found 182.0971.

Compound 2. The conversion of model compound **1** to the fluorine displaced product **2** was scaled up in 5 mL final volume following the procedure described in "*Small Molecule Model Reactions*". The reaction was quenched with 20 mL brine and the product was extracted with 30 mL of ethyl acetate three times. The organic layer was combined, dried, and concentrated under reduced pressure. The crude mixture was purified by flash column chromatography (25% ethyl acetate/hexane) to afford 80 mg white solid (70.7% yield). ^{1}H NMR (500 MHz, $CDCl_3$): δ 7.12-7.39 (m, 10H), 6.73 (s, 1H), 3.57 (s, 2H,), 3.46 (q, 2H), 3.09 (s, 2H), 2.79 (t, 2H); ^{13}C NMR (126 MHz, $CDCl_3$): δ 168.39, 138.65, 137.06, 128.97, 128.87, 128.77, 128.74, 127.46, 126.67, 40.71, 37.30, 35.43, 35.36. ESI-MS m/z calculated for $C_{17}H_{19}NOS$ $[M + H]^+$: 286.1, found 286.1.

Compound 3. Boc-Dap-OH (1 g, 4.9 mmol) was dissolved in the mixture of water (17 mL) and dioxane (47 mL). Na_2CO_3 (1.04 g, 9.8 mmol) in water (5 mL) was added to the flask, and the mixture was cooled on ice bath. Cbz-Cl (1.07 mL, 7.5 mmol) was then added dropwise, after which the mixture was stirred at room temperature overnight. The next day, dioxane was removed under reduced pressure, and the solution's pH was adjusted to approximately 9.0 with 1 M sodium hydroxide. The solution was extracted twice with ethyl acetate to remove the unreacted Cbz-Cl. The pH was subsequently adjusted to 4.0 with 1 M HCl and the acidic solution was then extracted three times by ethyl acetate, with the organic layers combined and dried over sodium sulfate. The organic phase was finally vacuum dried to afford 1.6 gram of crude product (96.5% yield) and was used directly for the next step without purification.

Compound 4. Compound **4** was synthesized following the same procedure for the enantiomer, compound **3**, with a 97.4% yield.

Compound 5. The previously synthesized compound **3** (1.6 g, 4.73 mmol) was dissolved in 20 mL of dry DMF. Potassium carbonate (2.29 g, 16.56 mmol) was added, and stirred at room temperature for 20 min. The reaction mixture was then cooled in an ice bath followed by the addition of methyl iodide (353 μL, 5.676 mmol). After overnight stirring at room temperature, the mixture was diluted with 200 mL water, and extracted three times with 60 mL ethyl acetate. The organic layers were combined, dried over anhydrous sodium sulfate, and concentrated under reduced pressure. The crude mixture was purified by flash column chromatography (ethyl acetate/hexane: 1/4) to finally afford 1.58 gram of product in viscous oil form (94% yield). ^{1}H NMR (500 MHz, $CDCl_3$): δ 7.36-7.30 (m, 5H), 5.46 (s, 1H, br), 5.20 (s, 1H, br), 5.08 (s, 2H), 4.37 (s, 1H, br), 3.73 (s, 3H,), 3.58 (s, 2H, br) 1.42 (s, 9H); ^{13}C NMR (126 MHz, $CDCl_3$): δ 171.18, 156.72, 155.43, 136.24, 128.56, 128.23, 128.15, 80.31, 67.03, 54.04, 52.71, 42.97, 28.30; HRMS(ESI) m/z calculated for $[C_{12}H_{17}N_2O_4$, M - Boc + H$]^+$: 253.1188, found 253.1181.

Compound 6. Compound **6** has been synthesized following the same procedure for compound **5**, with a 93.5% yield. ^{1}H NMR (500 MHz, $CDCl_3$): δ 7.35-7.28(m, 5H), 5.63 (s, 1H, br), 5.47 (s, 1H, br), 5.07 (s, 2H), 4.37 (s, 1H, br), 3.71 (s, 3H,), 3.56 (s, 2H, br) 1.43 (s, 9H); ^{13}C NMR (126 MHz, $CDCl_3$): δ 171.29, 156.83, 155.56, 136.33, 128.52, 128.17, 128.12, 80.19, 66.94, 54.08, 52.63, 42.85, 28.29; HRMS(ESI) m/z calculated for $[C_{12}H_{17}N_2O_4$, M - Boc + H$]^+$: 253.1188, found 253.1182.

Compound 7. The previously synthesized compound **5** (1.58 g, 4.48 mmol) was dissolved in 20 mL of methanol and cooled on ice bath. A small amount of hydrogen chloride (370 μL) in methanol (5 mL) was added dropwise in order to quench the nucleophilic amine generated in situ during follow up hydrogenolysis. For hydrogenolysis, 10% Pd/C (200 mg) was added with subsequent stirring at room temperature under the hydrogen atmosphere. After overnight stirring, the reaction mixture was filtered through celite to remove Pd/C. The solvent was also evaporated under reduced pressure to afford crude compound **7** with almost quantitative yield. The crude product was used for the next step without any purification.

*Compound **8***. As an enantiomer to compound **7**, compound **8** has been synthesized following the same procedure for compound **7**.

*Compound **9***. Sodium fluoroacetate (100 mg, 1 mmol), HATU (418.3 mg, 1.1 mmol), and compound **7** (436.4 mg, 2 mmol) were dissolved in 10 mL of DMF and stirred at room temperature for 20 min. DIPEA (608.5 µL, 3.5 mmol) was then added. The mixture was stirred overnight, before the reaction was quenched with 100 mL of brine. After extraction (three times) with 50 mL ethyl acetate, the organic layers were combined and dried over anhydrous sodium sulfate. The crude was vacuum concentrated, loaded onto silica column and purified by ethyl acetate/hexane (1:2) to afford 215 mg of oil-like product with a 77.3% yield. ^{1}H NMR (500 MHz, $CDCl_3$): δ 7.03 (s, 1H, br), 5.63 (s, 1H, br), 4.69-4.79 (d, J=50 Hz, 2H,), 4.39 (s, 1H, br), 3.71 (s, 3H,), 3.66 (t, J=5.0 Hz, 2H,), 1.38 (s, 9H); ^{13}C NMR (126 MHz, $CDCl_3$): δ 170.95, 168.47, 155.69, 80.84, 80.35, 79.36, 53.54, 52.72, 40.92, 28.19; HRMS(ESI) m/z calculated for [$C_6H_{12}FN_2O_3$, M - Boc + H]$^+$: 179.0832, found 179.0824.

*Compound **10***. Compound **10** was prepared similarly to compound **9**, with a 75% yield. ^{1}H NMR (500 MHz, $CDCl_3$): δ 6.94 (s, 1H, br), 5.55 (s, 1H, br), 4.81-4.72 (d, J=45 Hz, 2H,), 4.41 (s, 1H, br), 3.74 (s, 3H,), 3.68 (t, J=7.5 Hz, 2H,), 1.41 (s, 9H); ^{13}C NMR (126 MHz, $CDCl_3$): δ 170.90, 168.41, 155.69, 80.87, 80.46, 79.40, 53.49, 52.81, 41.07, 28.23; HRMS(ESI) m/z calculated for [$C_6H_{12}FN_2O_3$, M - Boc + H]$^+$: 179.0832, found 179.0825.

*Compound **11***. Compound **9** (225 mg, 0.81 mmol) was dissolved in 4 mL of tetrahydrofuran and 2 mL of methanol. The solution was cooled in an ice-bath and 0.89 mL of 1 M sodium hydroxide (0.89 mmol) was added. After 15 min of stirring, the ice-bath was removed followed by subsequent stirring at room temperature for 1 h. The reaction was then quenched with 60 mL of water, and the mixture was washed once with 30 mL of ethyl acetate. The remaining mixture in the aqueous phase was cooled on ice-bath again, with the pH adjusted to 4.0 by 1 M HCl. At this point, the solution was extracted three times with 50 mL of ethyl acetate each. The organic layers were combined and dried over anhydrous sodium sulfate. The solvent was removed under reduced pressure by rotavapor, to afford 192.3 mg of crude product (~ 90% yield) that was used directly for the next step.

*Compound **12***. Compound **12** was synthesized following the same procedure for the enantiomer, **11**, with a 91% yield.

*Compound **13***. For Boc deprotection, compound **11** (200 mg, 0.76 mmol) was dissolved in 5 mL of dichloromethane and cooled in an ice-bath. Trifluoroacetic acid (2.5 mL) was added dropwise, and the mixture was kept stirring on the ice-bath for 10 min, after which the stirring was continued at room temperature for 1h. The solvent was removed under vacuum, to afford 111 mg of crude product with an 89.5% yield.

*Compound **14***. Compound **14** was prepared similarly from compound **12**, with an 88% yield.

*Compound **15***. Towards 200 mg compound **13** (1.22 mmol) in 3.5 mL of ice-cold 10% sodium carbonate solution, Fmoc-OSu (452 mg, 1.34 mmol) in 4 mL of dioxane was added dropwise. The mixture was left stirring overnight at room temperature. Dioxane was removed by vacuum, followed by the addition of 15 mL of water. The solution was washed with 10 mL of diethyl ether once. The aqueous phase was cooled with ice bath, and the solution's pH was adjusted to 3.5 by citric acid. Ethyl acetate was then used to extract the solution for three times (30 mL each). The combined organic phase was dried by anhydrous sodium sulfate, vacuum concentrated, and purified by flash column chromatography (2.5% Methanol /96.5% DCM /1% acetic acid). Finally, 396 mg of product in white solid form was obtained with a 91.5% yield. ^{1}H NMR (500 MHz, CD_3OD): δ 7.72 (d, *J*=5 Hz, 2H), 7.60 (s, 2H, br), 7.32 (t, *J*=7.5 Hz, 2H), 7.25 (t, *J*=7.5 2H,), 4.70-4.79 (d, 1H), 4.35 (s, 1H, br), 4.27 (d, *J*=10 Hz, 2H), 4.15 (t, *J*=7.5 Hz, 1H,), 3.71-3.54 (m, 2H); ^{13}C NMR (126 MHz, CD_3OD): δ 169.73, 169.58, 157.16, 143.86, 141.16, 127.39, 126.77, 124.85, 119.53, 80.35, 78.89, 66.77, 48.14, 39.63; HRMS(ESI) m/z calculated for [$C_{20}H_{20}FN_2O_5$, M + H]$^+$: 387.1356, found 387.1351.

*Compound **16***. Compound **16** has been synthesized following the same procedure for compound **15**, with a 90.7% final yield. ^{1}H NMR (500 MHz, CD_3OD): δ 7.76 (d, J=10 Hz, 2H), 7.64 (m, 2H), 7.36 (t, *J*=7.5 Hz, 2H), 7.28 (t, *J*=7.5 Hz, 2H), 4.82-4.72 (dd, *J*=50 and 2.5 Hz, 2H), 4.37 (m, 1H), 4.32 (m, 2H), 4.20 (t, *J*=7.5 Hz, 1H), 3.74-3.56 (m, 2H); ^{13}C NMR (126 MHz, CD_3OD): δ 171.99, 169.73, 157.17, 143.87, 141.18, 127.39, 126.77, 124.86, 119.52, 80.34, 78.83, 66.77, 48.12, 39.60; HRMS(ESI) m/z calculated for [$C_{20}H_{20}FN_2O_5$, M + H]$^+$: 387.1356, found 387.1350.

*Compound **17***. Towards a mixture of 2 mL of DMF and 4 mL of Tris (3M solution) were added 470 µL of compound **9** (600 mM in DMF), 500 µL of benzyl thiol (600 mM in DMF), and 525 µL of TCEP solution (600 mM). After the final pH was adjusted to 8.5, the mixture was left stirring at 37 ^{o}C for 12h. The reaction was then quenched with 20 mL of brine and the product was extracted with 30 mL of ethyl acetate three times. The organic layers were combined, dried over anhydrous sodium sulfate, and concentrated under reduced pressure. The crude mixture was purified by flash column chromatography (25% ethyl acetate/hexane) to finally afford 50.2 mg of compound **17** as a white solid (73% yield). ^{1}H NMR (500 MHz, $CDCl_3$): δ 7.24-7.34 (m, 5H), 7.08 (s,1H), 5.49 (s, 1H), 4.39 (m, 1H,), 3.77 (s, 3H), 3.72 (s, 2H), 3.58 (m, 2H), 3.11 (s, 2H), 1.45 (s, 9H); ^{13}C NMR (126 MHz, $CDCl_3$): δ 171.09, 169.48, 155.57, 136.95, 129.07, 128.75, 127.90, 80.39, 53.94, 52.78, 41.62, 36.93, 35.04, 28.31; HRMS(ESI) m/z calculated for [$C_{13}H_{19}N_2O_3S$, M - Boc + H]$^+$: 283.1116, found 283.1109.

Supplementary Discussion:

Supplementary Table 1. Molecular weights/yields of cyclized model peptides and Axin analogues.

Peptide	Theoretical MW in Da	Observed MW [M+H]	Observed MW [M+2H]/2	Observed MW [M+3H]/3	Observed MW [M+4H]/4	% yield
**23**	1015.2	1016.3	508.8			78%
**24**	1015.2	1016.3	508.8			76%
**25**	1015.2	1016.3	508.8			77%
**27**	1015.2	1016.3	508.8			74%
**29**	1693.0			565.3	424.1	22%
**31**	1725.0		863.0	575.8	432.2	23%
**33**	1582.9		791.9	528.4		27%
**35**	2007.9		1005.1	670.5		55%
**36**	2021.9		1012.2	675.2		59%
**37**	2036.0		1019.2	679.9		49%
**38**	2050.0		1026.1	684.5		52%
**39**	2041.9		1022.2	681.9		63%
**40**	2041.9		1022.1	681.8		52%
**42**	2007.9		1005.2	670.5		58%
**43**	2021.9		1012.2	675.2		62%
**44**	2036.0		1019.1	679.9		60%
**45**	2050.0		1026.1	684.6		55%
**46**	2041.9		1022.2	681.9		59%
**47**	2041.9		1022.2	681.9		65%
**49**	2036.0		1019.1	679.9		54%
**50**	2050.0		1026.1	684.6		43%
**51**	2041.9		1022.2	681.9		58%
**53**	2036.0		1019.1	679.9		39%
**54**	2050.0		1026.1	684.5		50%
**55**	2041.9		1022.2	681.9		51%
**56***	1870.0		936.2	624.5		73%
**57**	1048.2	1049.0	525.1			64%
**58**	1048.2	1049.1	525.1			61%
**59**	1048.2	1049.1	525.1			58%

*: Control peptides stapled by ring-closing metathesis. **Bold**: peptide numbering.

Supplementary Table 2. ^{1}H and ^{13}C chemical shifts assignments of peptide **57**.

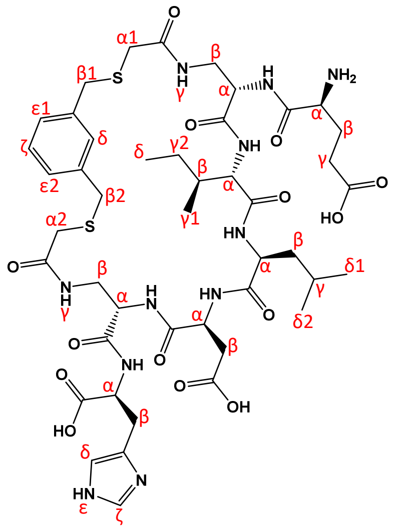

Peptide **57** (^{1}H) — δ (ppm)

AA	NH	α	β	γ	δ	ε	ζ	α1	α2	β1	β2	γ1	γ2	δ1	δ2	ε1	ε2
**Glu**		3.86	1.91	2.32													
**X_L (Top)**	8.74	4.50	3.14/ 3.47	7.75													
**Ile**	8.22	4.10	1.75		0.78							0.78	1.10/ 1.38				
**Leu**	7.92	4.29	1.46	1.61										0.78	0.78		
**Asp**	8.1	4.51	2.54/ 2.68														
**X_L (Bottom)**	7.99	4.35	3.17/ 3.47	7.92													
**His**	8.26	4.44	2.98/ 3.06		7.10 - 7.30	8.44	7.10 - 7.30										
**Staple**					7.10 - 7.30		7.10 - 7.30	3.04	3.04	3.75	3.75					7.10 - 7.30	7.10 - 7.30

Peptide **57** (^{13}C) — δ (ppm)

AA	α	β	γ	δ	ζ	α1	α2	β1	β2	γ1	γ2	δ1	δ2	ε1	ε2
**Glu**	51.8	26.8	29.3												
**X_L (Top)**	53.3	40.8													
**Ile**	58.1	36.6		11.4						23.4	24.6				
**Leu**	51.5	40.48	24.3									15.7	21.8		
**Asp**	50.1	36.3													
**X_L (Bottom)**	53.3	40.8													
**His**	52.2	27.2		117.2	127.8										
**Staple**				130.1	127.8	34.4	34.8	35.8	35.8					129.1	129.1

Supplementary Table 3. ^{1}H and ^{13}C chemical shifts assignments of peptide **58**.

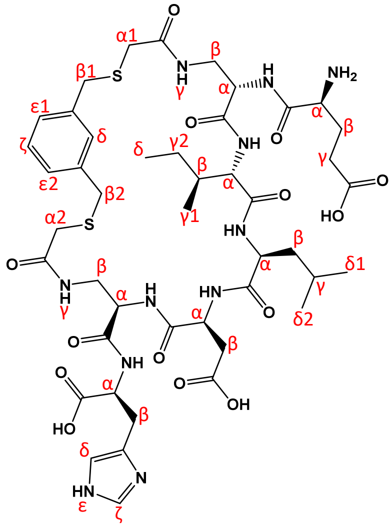

Peptide **58** (^{1}H) **δ (ppm)**

AA	NH	α	β	γ	δ	ε	ζ	α1	α2	β1	β2	γ1	γ2	δ1	δ2	ε1	ε2
**Glu**		3.91	1.96	2.32													
**X$_L$ (Top)**	8.82	4.63	3.28/ 3.48	7.99													
**Ile**	8.13	4.12	1.76		0.80							0.8	1.05/ 1.38				
**Leu**	8.20	4.17	1.49	1.54										0.8	0.8		
**Asp**	8.30	4.35	2.61/ 2.71														
**X$_D$ (Bottom)**	8.07	4.27	3.36	8.09													
**His**	8.16	4.47	3.01/ 3.11		7.57	8.75	7.10 - 7.30										
**Staple**					7.10 - 7.30		7.10 - 7.30	3.36	3.36	3.77	3.77					7.10 - 7.30	7.10 - 7.30

Peptide **58** (^{13}C) **δ (ppm)**

AA	α	β	γ	δ	ζ	α1	α2	β1	β2	γ1	γ2	δ1	δ2	ε1	ε2
**Glu**	52.1	26.7	29.6												
**X$_L$ (Top)**	53.0	41.4													
**Ile**	58.1	36.8		11.3						21.6	24.8				
**Leu**	52.1	40.4	24.1									15.5	23.3		
**Asp**	51.1	36.1													
**X$_D$ (Bottom)**	54.0	40.9													
**His**	52.2	34.4		120.3	117.1										
**Staple**				130.3	129.2	41.0	41.0	35.9	35.9					127.9	127.9

Supplementary Table 4. ^{1}H and ^{13}C chemical shifts assignments of peptide **59**.

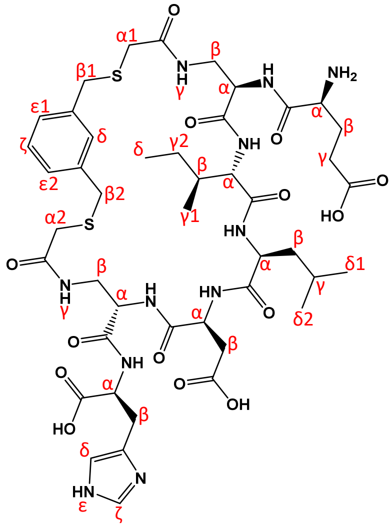

Peptide **59** (^{1}H)								δ (ppm)									
**AA**	**NH**	**α**	**β**	**γ**	**δ**	**ε**	**ζ**	**α1**	**α2**	**β1**	**β2**	**γ1**	**γ2**	**δ1**	**δ2**	**ε1**	**ε2**
**Glu**		3.84	1.91	2.31													
**X_D (Top)**	8.76	4.50	3.04/ 3.51	7.76													
**Ile**	8.22	4.15	1.77		0.80							0.80	1.09/ 1.37				
**Leu**	7.95	4.27	1.46	1.60										0.80	0.80		
**Asp**	8.12	4.50	2.54/ 2.66														
**X_L (Bottom)**	8.02	4.34	3.04/ 3.51	7.94													
**His**	8.32	4.50	3.04		7.11 - 7.35	8.76	7.11 - 7.35										
**Staple**					7.11 - 7.35		7.11 - 7.35	3.04	3.04	3.77	3.77					7.11 - 7.35	7.11 - 7.35

Peptide **59** (^{13}C)							δ (ppm)								
**AA**	**α**	**β**	**γ**	**δ**	**ζ**	**α1**	**α2**	**β1**	**β2**	**γ1**	**γ2**	**δ1**	**δ2**	**ε1**	**ε2**
**Glu**	51.8	26.7	29.3												
**X_D (Top)**	52.2	40.8													
**Ile**	58.0	36.6		11.4						21.8	24.6				
**Leu**	51.6	40.5	24.0									15.7	23.4		
**Asp**	51.6	36.1													
**X_L (Bottom)**	53.4	40.8													
**His**	50.2	26.7		117.7	127.8										
**Staple**				130.2	129.1	34.4	34.4	35.8	35.8					127.8	127.8

Supplementary Table 5. Summary of the molecular weights (MWs) and yields of all the stapled functional peptides.

Peptide	Theoretical MW in Da	Observed MW [M+2H]/2	Observed MW [M+3H]/3	Observed MW [M+4H]/4	Observed MW [M+5H]/5	Observed MW [M+6H]/6	% yield
**60**	2081.8	1042.2					59%
**61**	2081.8	1042.2					51%
**62**	2081.8	1042.3					56%
**63***	1909.9	956.4					51%
**65**	2871.3		958.5	719.2	575.6	479.8	54%
**67**	2871.3		958.5	719.1	575.6	479.7	53%
**68***	2699.4		901.3	676.2	542.5	451.1	63%
**70**	3344.3		1115.7	837.0	669.8		64%
**72**	3344.3		1115.7	837.0	669.8		61%
**73***	3172.2		1058.1	794.1	635.4		37%
**75**	2601.6	1301.5	868.0	651.3	521.3		74%
**77**	2601.6	1301.8	868.1	651.3	521.1		73%
**78***	2428.9	1215.4	810.7	608.3			42%
**80**	2232.1	1117.4	745.4				65%
**81**	2246.1	1124.4	750.0				69%
**82**	2238.0	1120.4	747.3				74%
**83**	2314.1	1158.4	772.5				55%
**85**	2232.1	1117.4	745.4				69%
**86**	2246.1	1124.7	750.0				71%
**87**	2238.0	1120.4	747.2				79%
**88**	2314.1	1158.4	772.5				55%
**89***	2108.2	1055.5	703.9				58%
**91**	2657.6	1329.6	886.7				75%
**93**	2657.6	1329.6	886.6				74%
**94***	2527.8	1264.6	843.4				40%

*: Control peptides stapled by ring-closing metathesis. **Bold:** peptide numbering.

Supplementary Table 6. Summary of simulation trajectory data for stapled/unstapled Axin and HIV peptides.

peptide	Fluoro substrates	Simulation time (μs)
**34**	$L_{i,\ i+4}L$	77.40
**35**	$L_{i,\ i+4}L$	76.25
**36**	$L_{i,\ i+4}L$	82.10
**37**	$L_{i,\ i+4}L$	76.90
**38**	$L_{i,\ i+4}L$	84.35
**39**	$L_{i,\ i+4}L$	75.20
**40**	$L_{i,\ i+4}L$	82.25
**41**	$D_{i,\ i+4}D$	62.60
**42**	$D_{i,\ i+4}D$	74.10
**43**	$D_{i,\ i+4}D$	71.85
**44**	$D_{i,\ i+4}D$	83.45
**45**	$D_{i,\ i+4}D$	76.55
**46**	$D_{i,\ i+4}D$	76.75
**47**	$D_{i,\ i+4}D$	76.40
**48**	$L_{i,\ i+4}D$	68.10
**49**	$L_{i,\ i+4}D$	78.40
**50**	$L_{i,\ i+4}D$	84.10
**51**	$L_{i,\ i+4}D$	65.25
**52**	$D_{i,\ i+4}L$	66.50
**53**	$D_{i,\ i+4}L$	79.45
**54**	$D_{i,\ i+4}L$	82.05
**55**	$D_{i,\ i+4}L$	84.80
**60**	$L_{i,\ i+4}L$	51.65
**61**	$L_{i,\ i+4}D$	60.00
**62**	$D_{i,\ i+4}L$	63.30
	total	1859.75
	average	74.39

Supplementary Table 7. Quantification of the cell penetration of HIV C-CA binding peptides.

Peptide	Mean Intensity Ratio	N (cell number)	Peptide	Mean Intensity Ratio	N (cell number)
**63**[*]	1.00 ± 0.04	108	**61**	5.75 ± 0.07	121
**60**	4.76 ± 0.09	81	**62**	3.07 ± 0.08	115

*: Control peptides stapled by ring-closing metathesis. **Bold**: peptide numbering.

Supplementary Table 8. Quantification of the cell penetration of Axin analogues.

Peptide	Mean Intensity Ratio	N (cell number)	Peptide	Mean Intensity Ratio	N (cell number)
**68**[*]	1.00 ± 0.02	91	**66**	0.33 ± 0.01	102
**64**	0.36 ± 0.02	102	**67**	5.05 ± 0.14	110
**65**	4.86 ± 0.15	101	**FITC only**	0.30 ± 0.02	104

*: Control peptides stapled by ring-closing metathesis. **Bold:** peptide numbering.

Supplementary Table 9. Quantification of the cell penetration of stapled Axin analogue **65** during pathway blocking studies.

Pathway Blocker	Mean Intensity Ratio	N	Pathway Blocker	Mean Intensity Ratio	N
Control[*]	1.00 ± 0.04	52	Cytochalasin D	0.10 ± 0.01	60
Nystatin	1.16 ± 0.03	48	$NaClO_3$	0.12 ± 0.01	63
Chlorpromazine	0.13 ± 0.01	61			

*: Control: solvent vehicle only.

Supplementary Table 10. Quantification of the cell penetration of stapled Axin analogue **67** during pathway blocking studies.

Pathway Blocker	Mean Intensity Ratio	N	Pathway Blocker	Mean Intensity Ratio	N
Control[*]	1.00 ± 0.02	54	Cytochalasin D	0.99 ± 0.02	56
Nystatin	0.17 ± 0.01	59	$NaClO_3$	0.20 ± 0.01	49
Chlorpromazine	0.16 ± 0.01	53			

*: Control: solvent vehicle only.

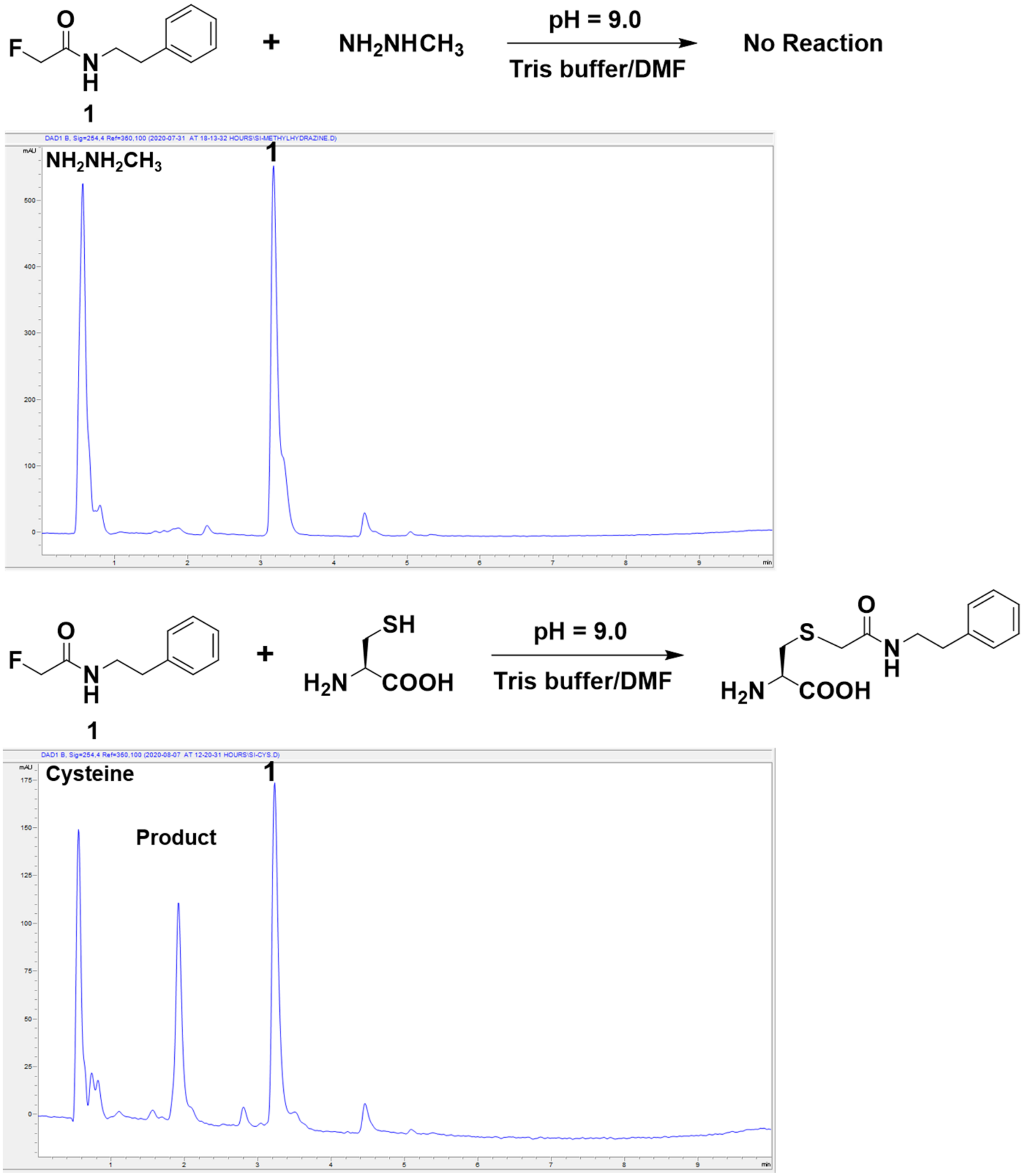

Supplementary Figure 1.3. The reaction of the fluoroacetamide-containing model compound **1** with methyl hydrazine and cysteine, respectively. For the reaction with cysteine, the mixture contained 500 mM TCEP to ensure a reducing environment. The reaction progress was monitored by LC-MS after 12h of incubation at 37°C.

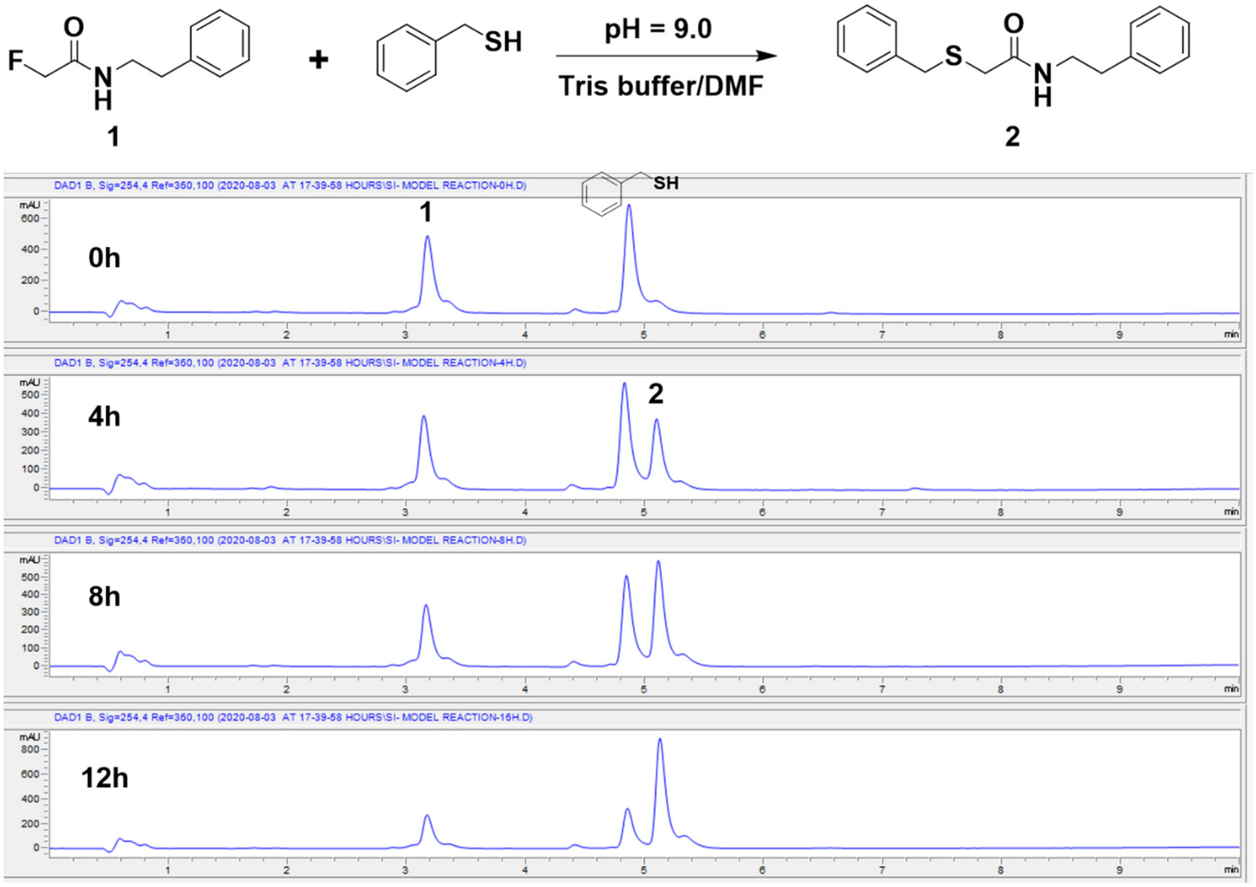

Supplementary Figure 2. Time-dependent fluorine displacement reaction between the model compound **1** and benzyl thiol. The mixture contained 500 mM TCEP in the Tris/DMF solution. The reaction progress was monitored by LC-MS.

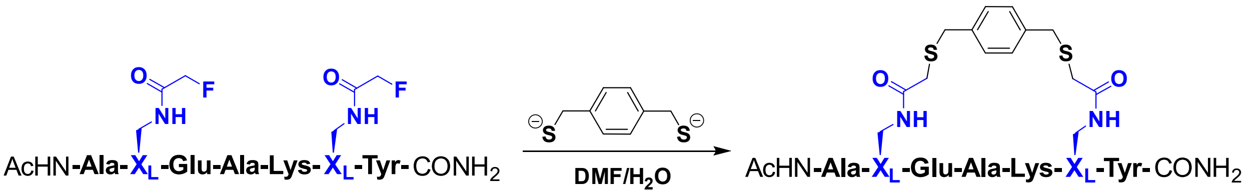

18 **19**

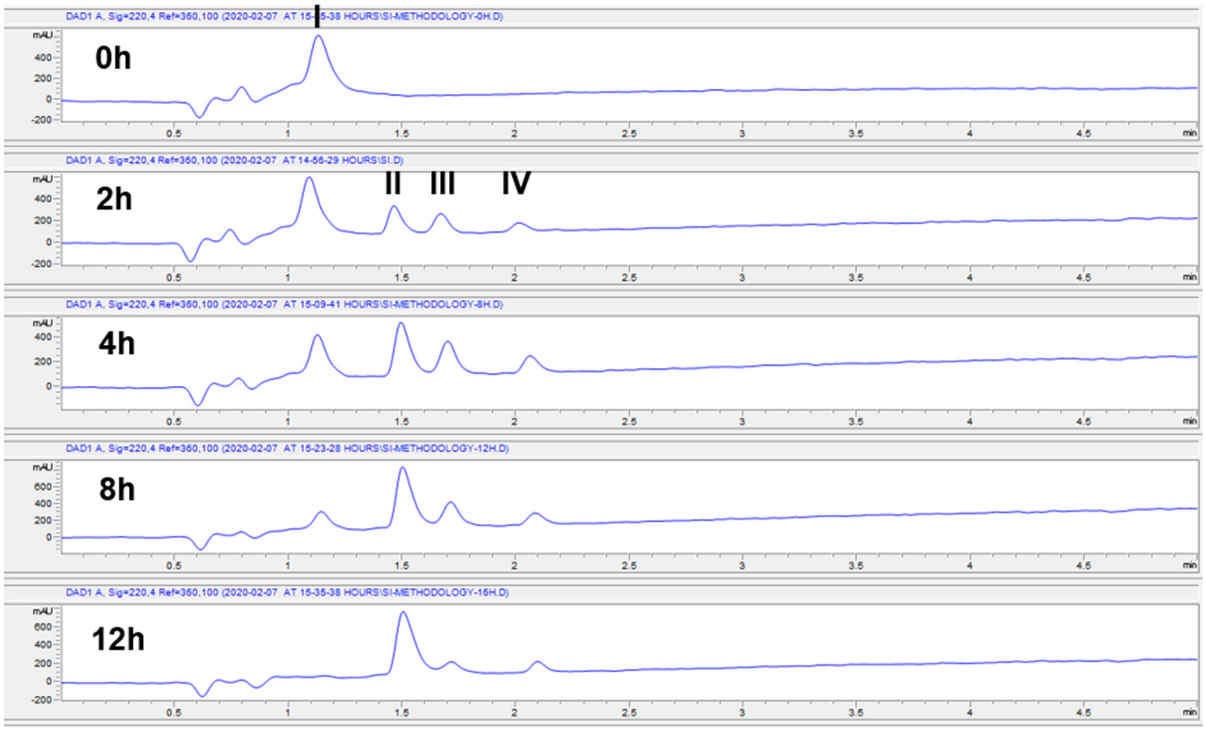

Supplementary Figure 3. Time-dependent stapling of the linear model peptide **18** with the linker 1,4-benzenedimethanethiol. The reaction progress was monitored by LC-MS. The peak **I** is starting peptide **18**; peak **II** is the desired product **19**; peak **III** is the noncyclic byproduct modified by one equivalent of linker; peak **IV** is the noncyclic byproduct modified by two equivalents of linkers.

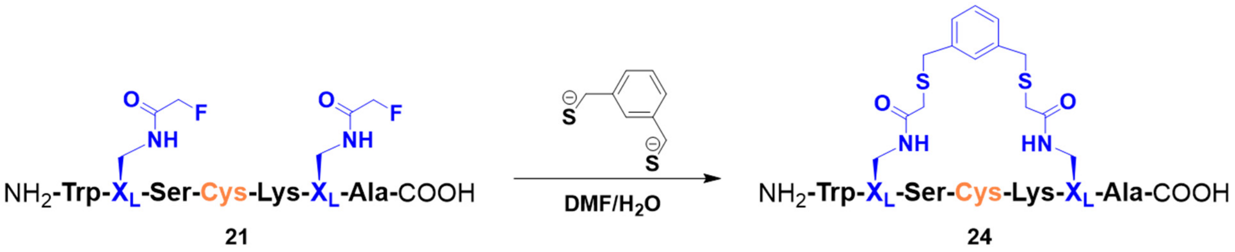

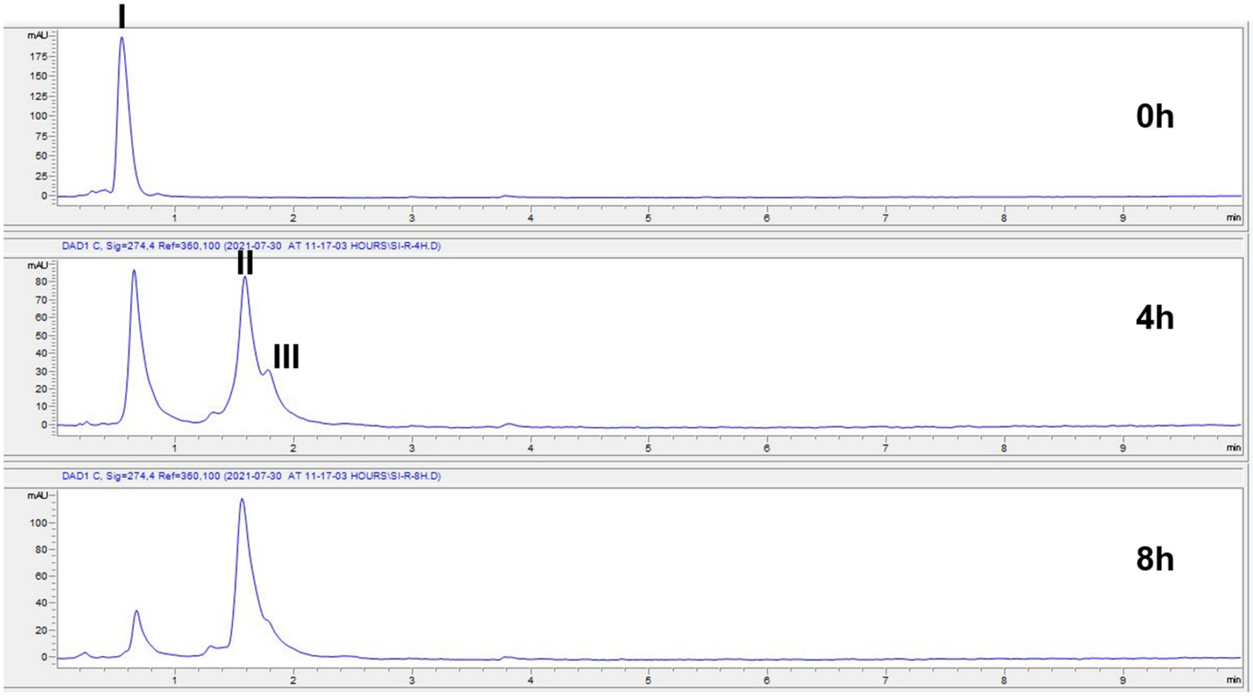

Supplementary Figure 4. Time-dependent stapling of the linear model peptide **21** with the linker, 1,3-benzenedimethanethiol. Reaction progress was monitored by LC-MS. Peak **I** is the starting peptide **21**; peak **II** is the desired product **24**; peak **III** is the noncyclic byproduct derivatized with one equivalent of linker.

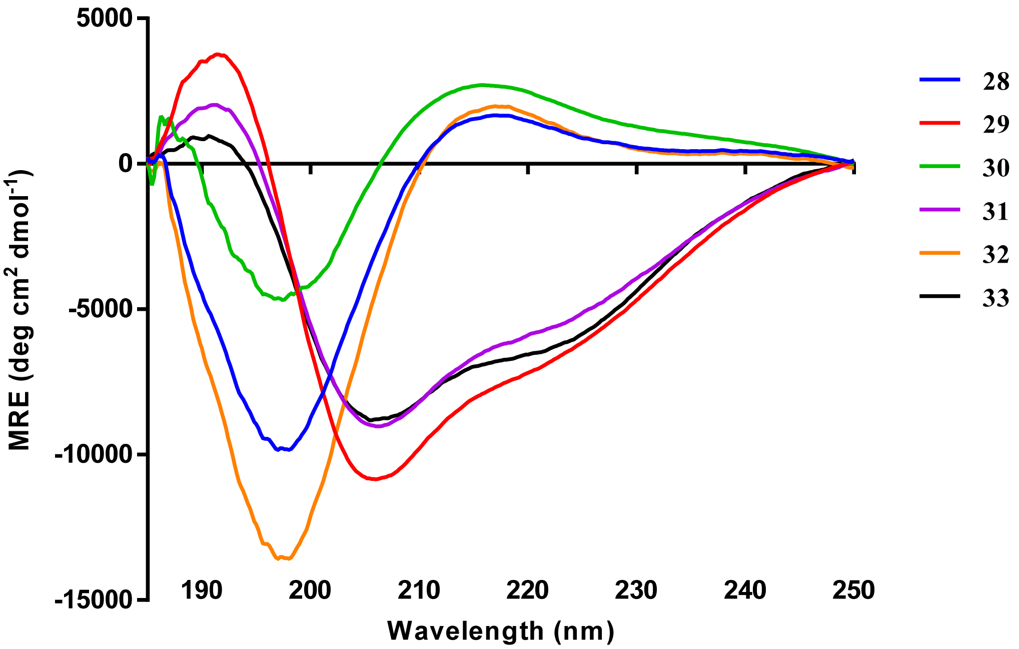

Supplementary Figure 5. Circular dichroism (CD) spectra of the cysteine-containing peptides in Figure 2b. Peptides **28**, **30**, **32** and **29**, **31**, **33** represent before and after terminal FTDR stapling, respectively.

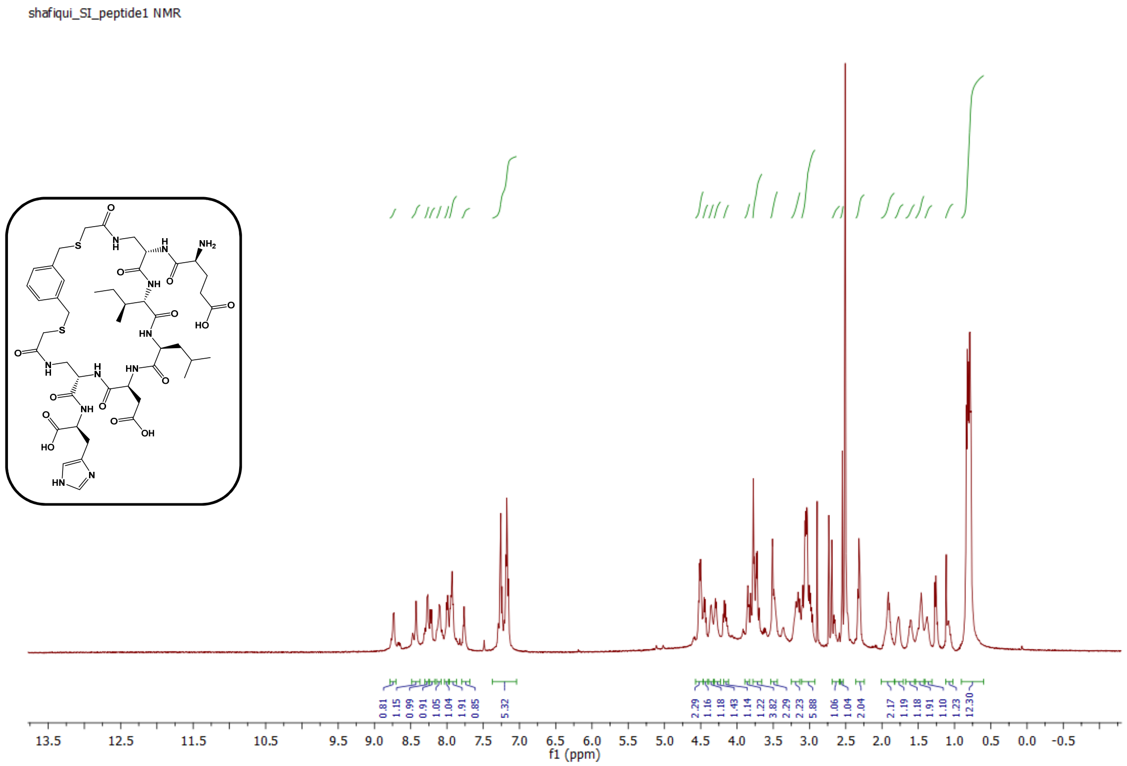

Supplementary Figure 6. ^{1}H NMR spectrum of peptide **57**.

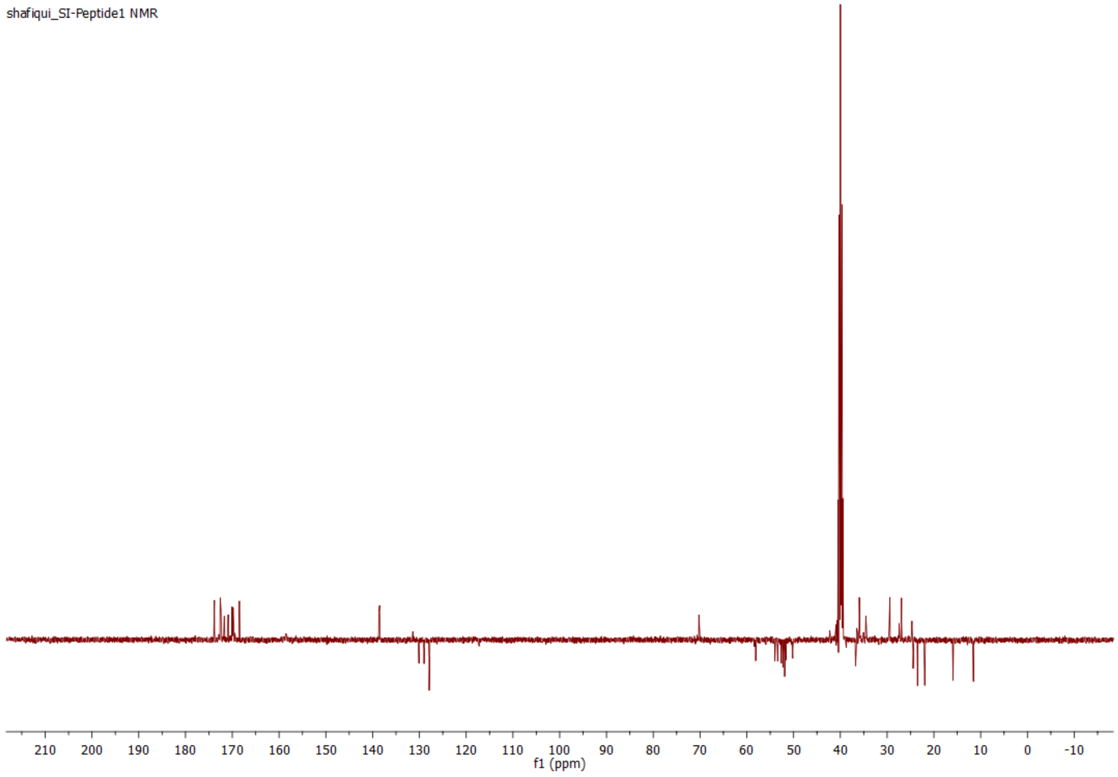

Supplementary Figure 7. APT ^{13}C NMR spectrum of peptide **57**.

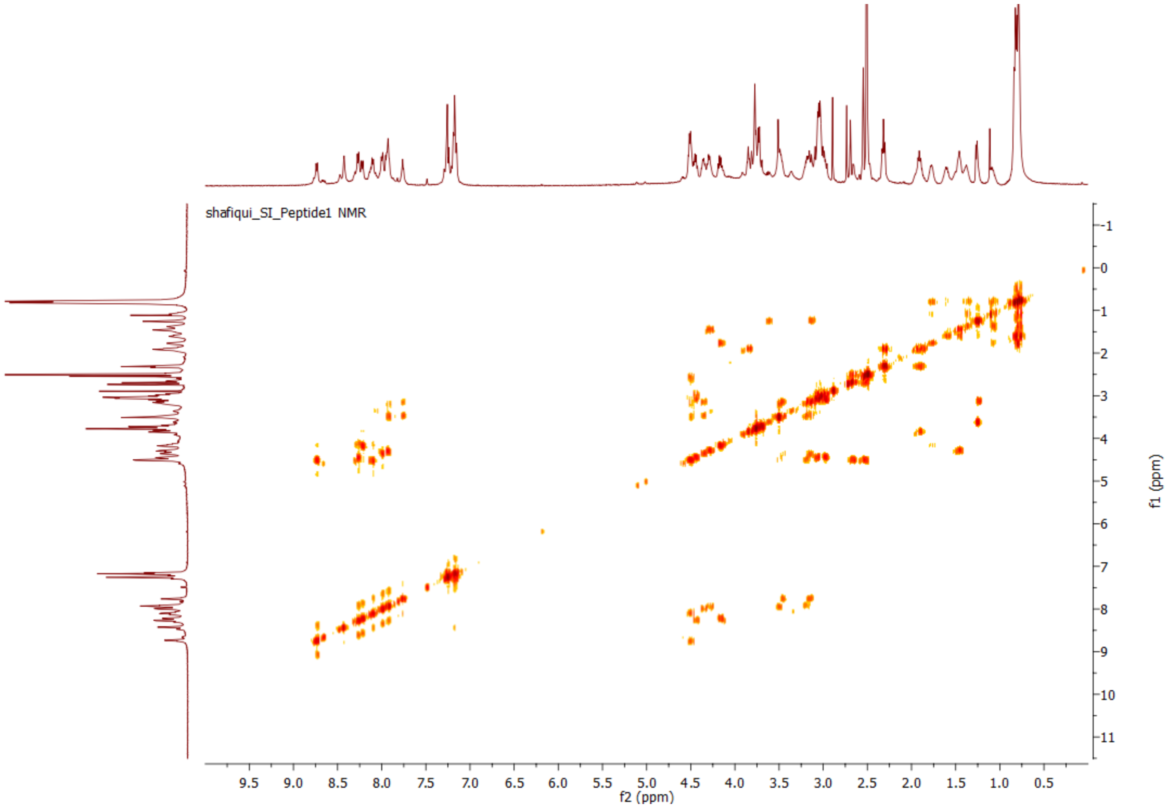

Supplementary Figure 8. COSY NMR spectrum of peptide **57**.

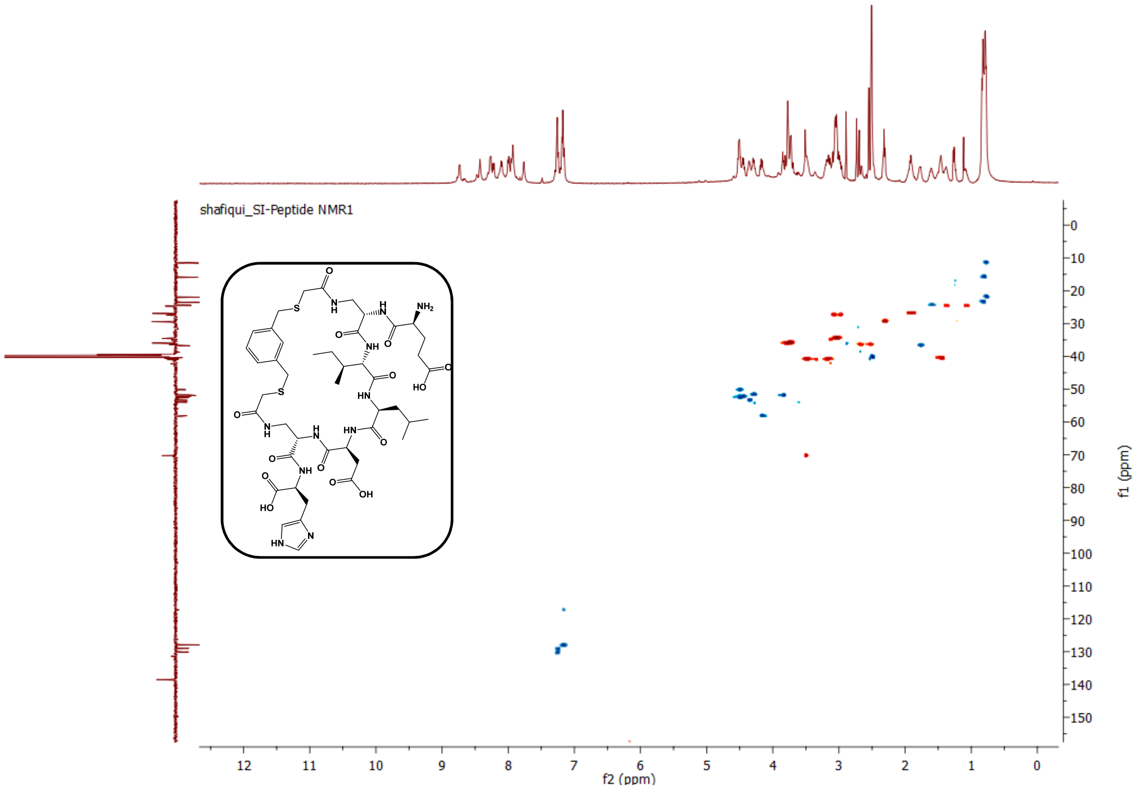

Supplementary Figure 9. ^{1}H-^{13}C HSQC NMR spectrum of peptide **57**.

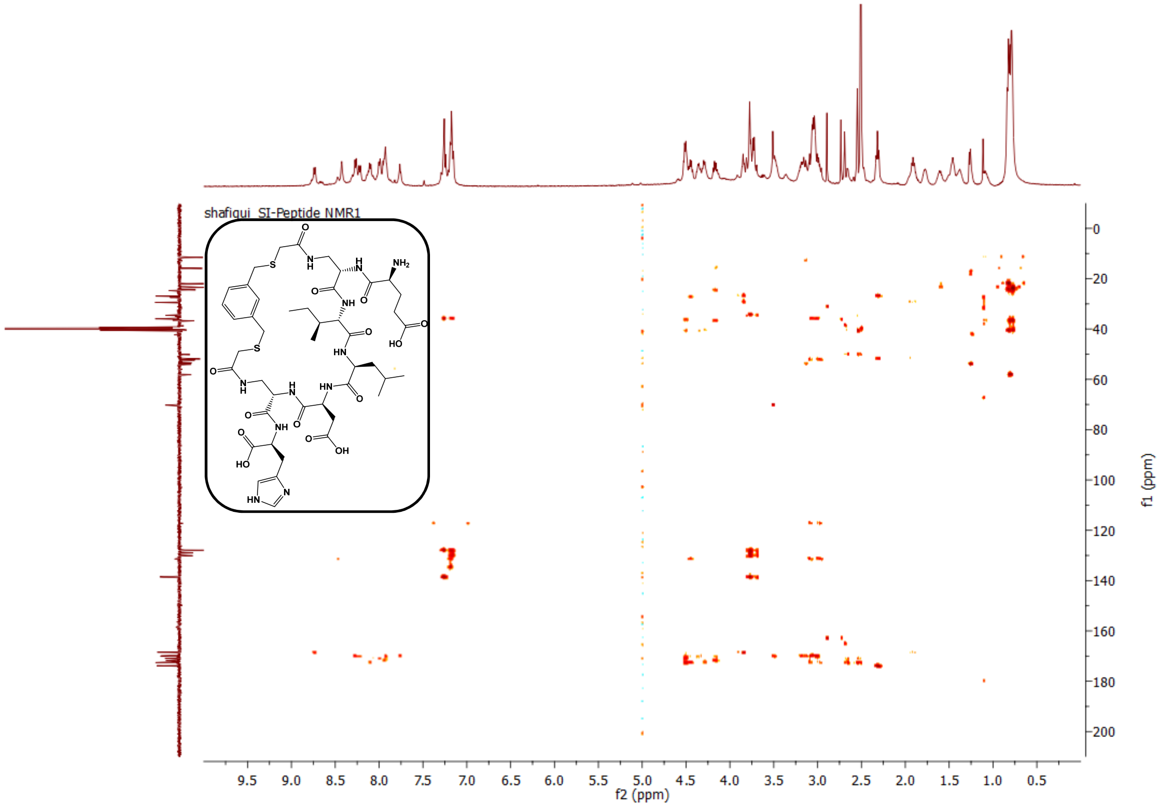

Supplementary Figure 10. ^{1}H-^{13}C HMBC NMR spectrum of peptide **57**.

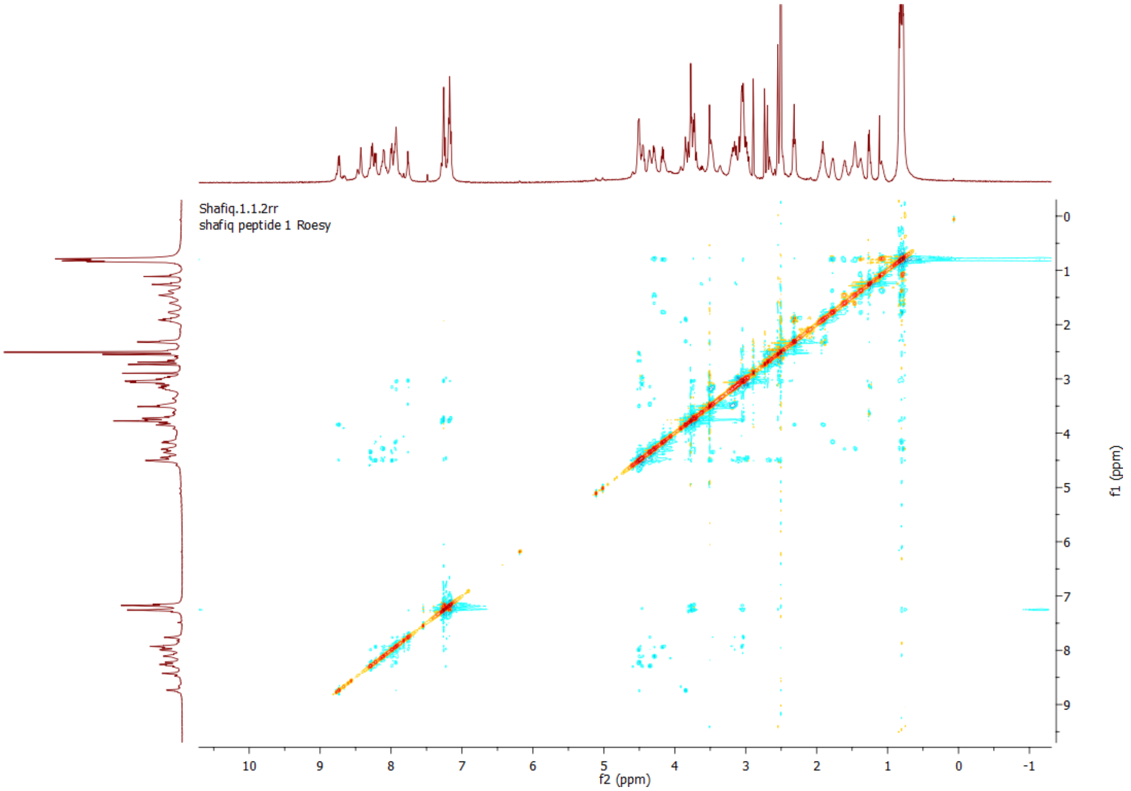

Supplementary Figure 11. ROESY NMR spectrum of peptide **57**.

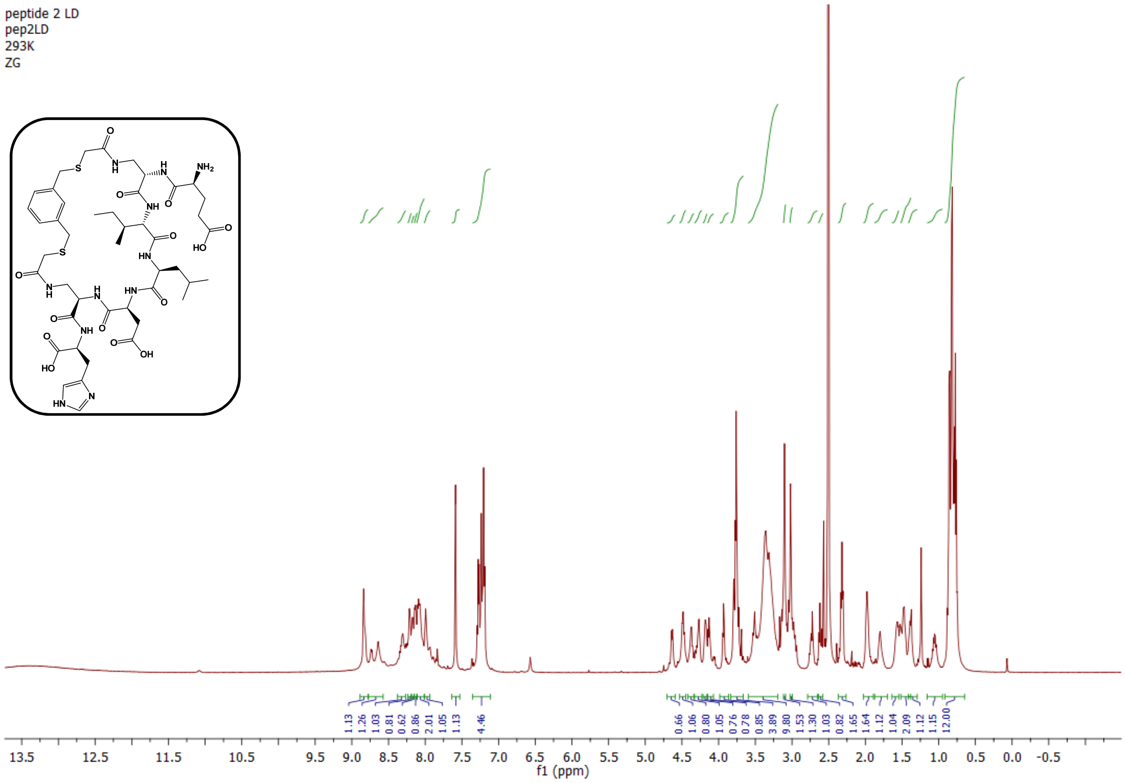

Supplementary Figure 12. ^{1}H NMR spectrum of peptide **58**.

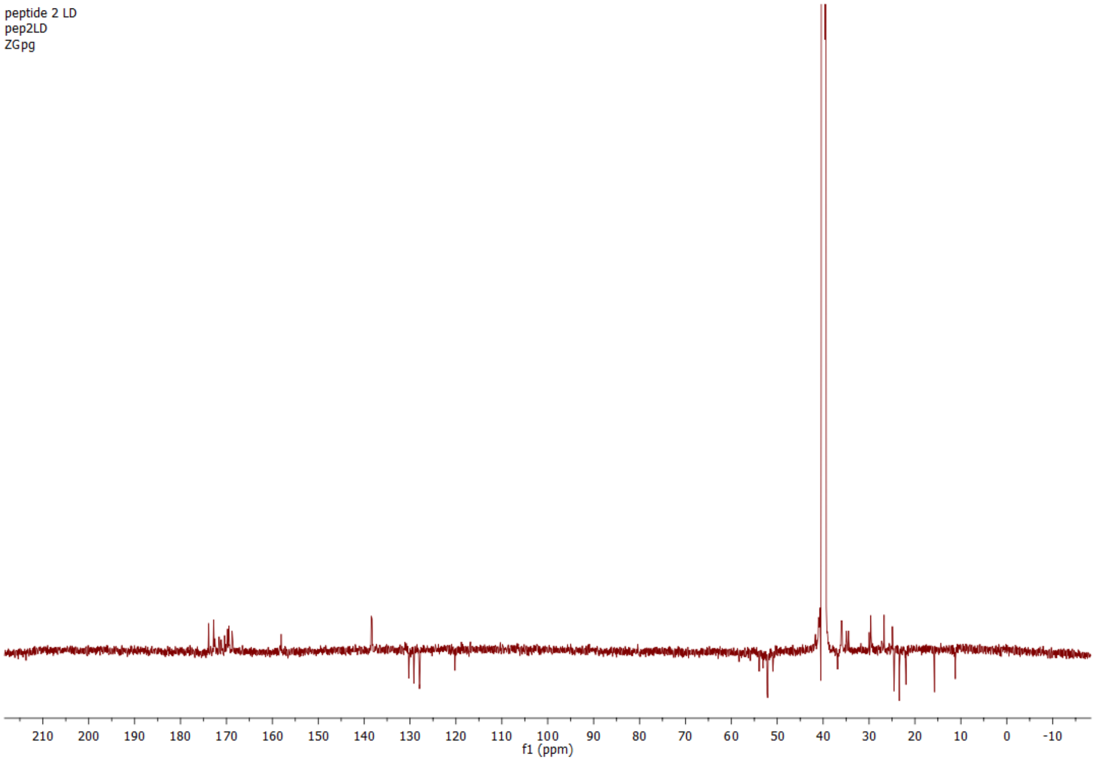

Supplementary Figure 13. APT ^{13}C NMR spectrum of peptide **58**.

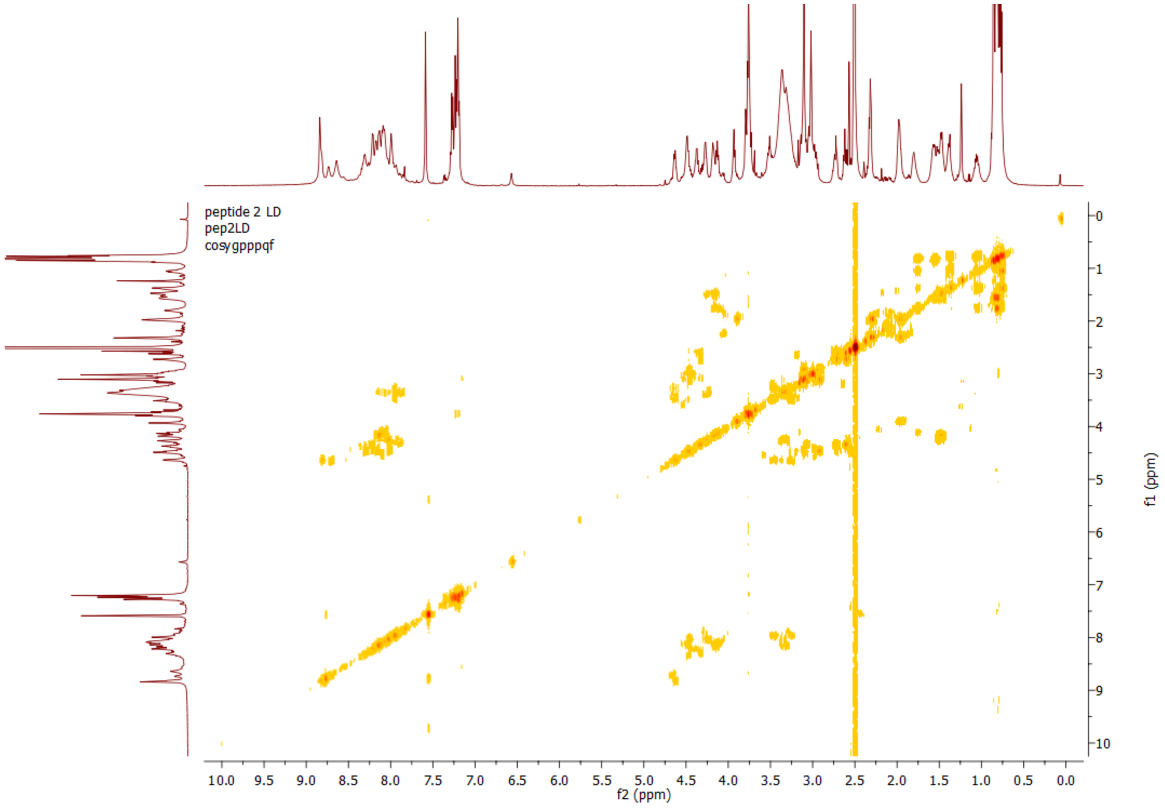

Supplementary Figure 14. COSY NMR spectrum of peptide **58**.

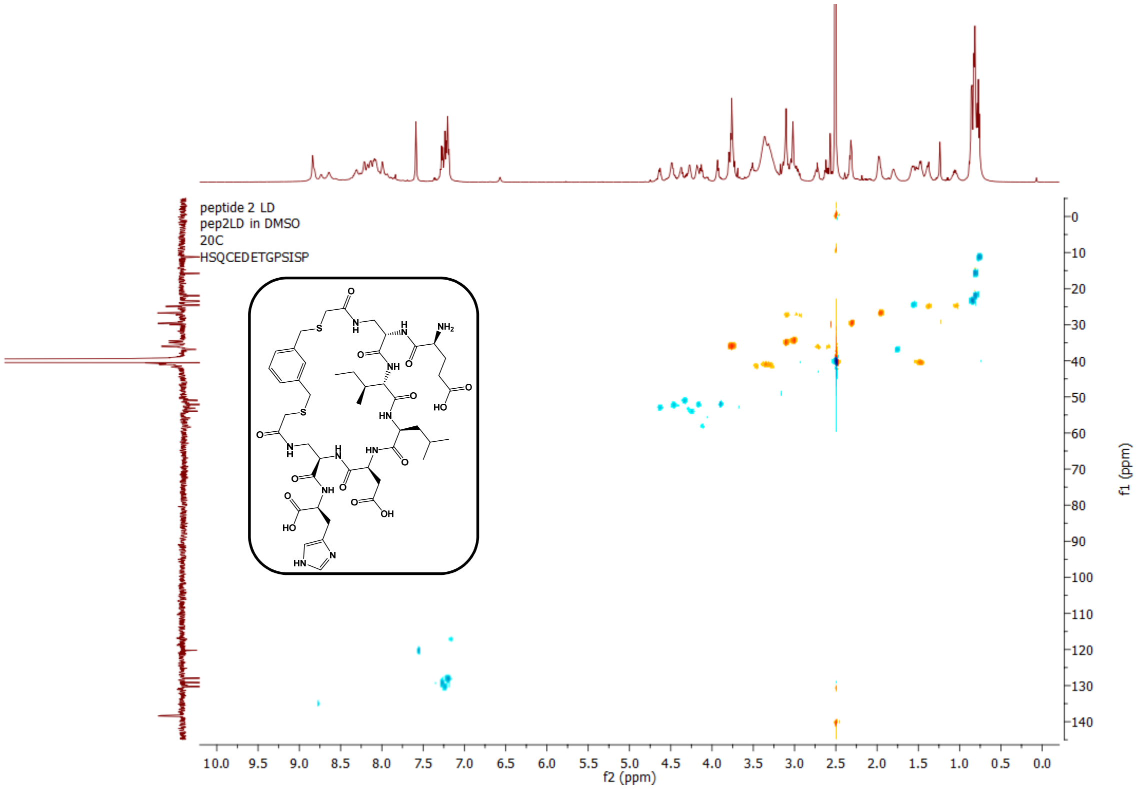

Supplementary Figure 15. ^{1}H-^{13}C HSQC NMR spectrum of peptide **58**.

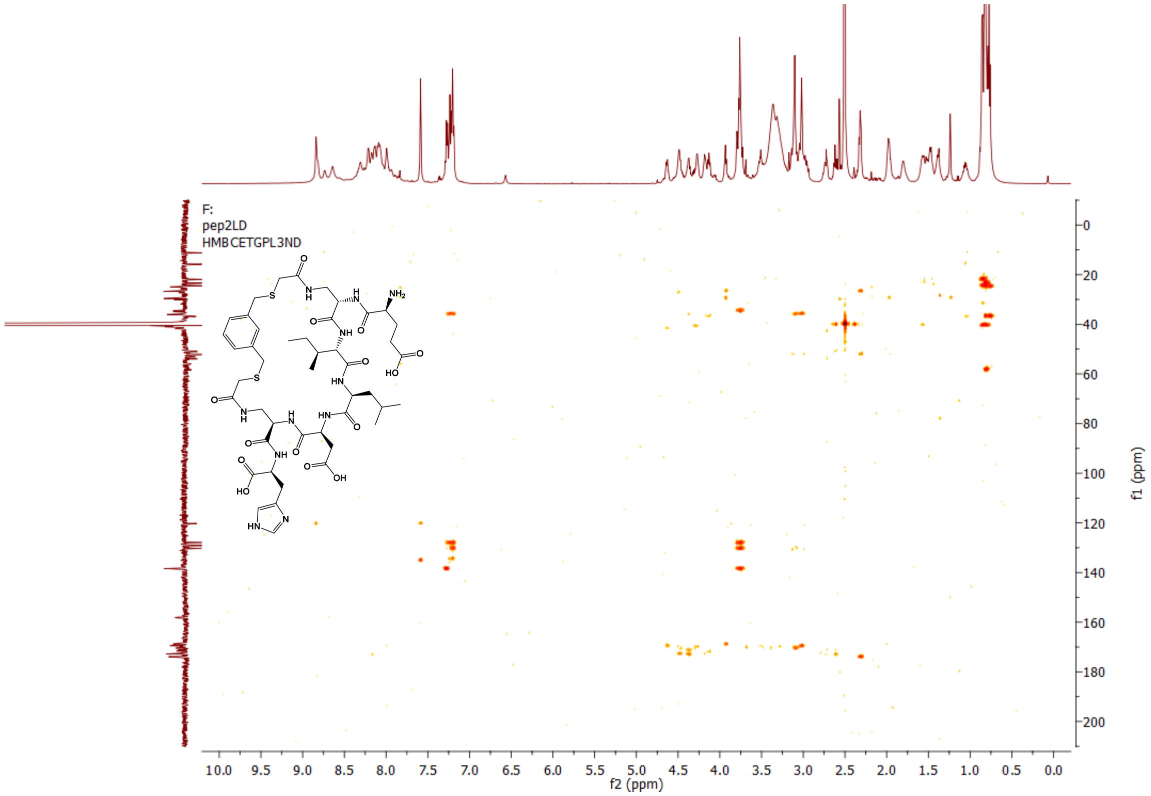

Supplementary Figure 16. ^{1}H-^{13}C HMBC NMR spectrum of peptide **58**.

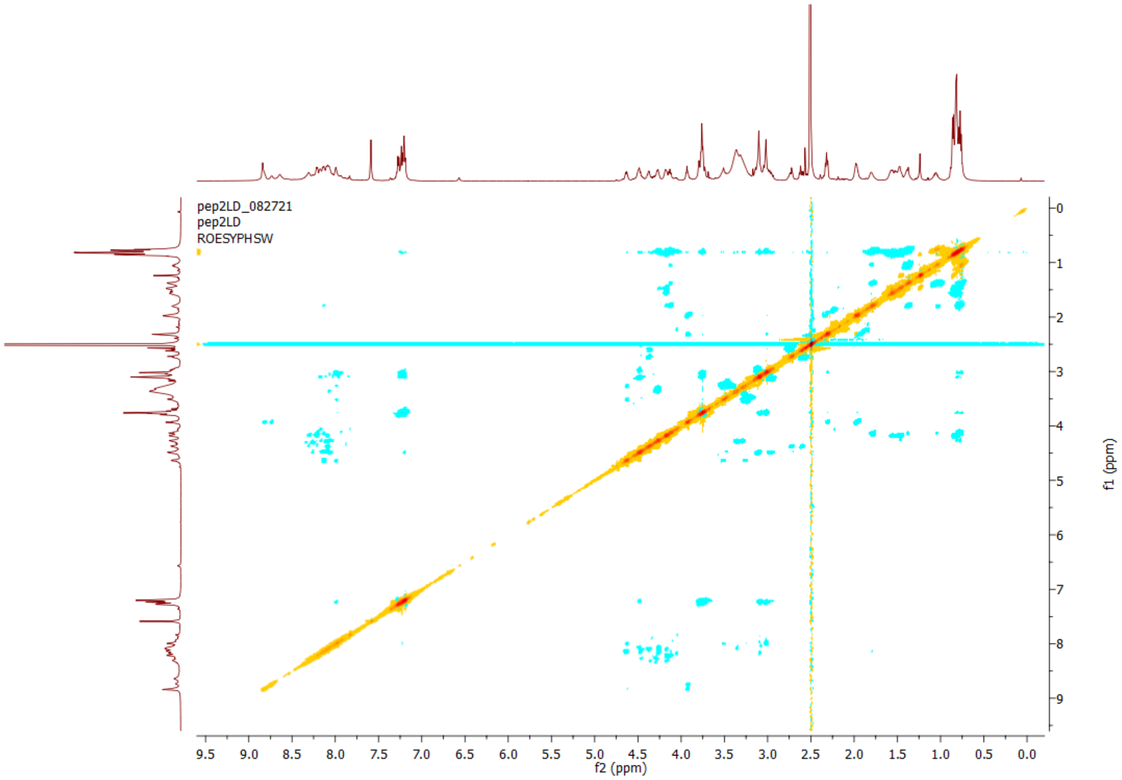

Supplementary Figure 17. ROESY NMR spectrum of peptide **58**.

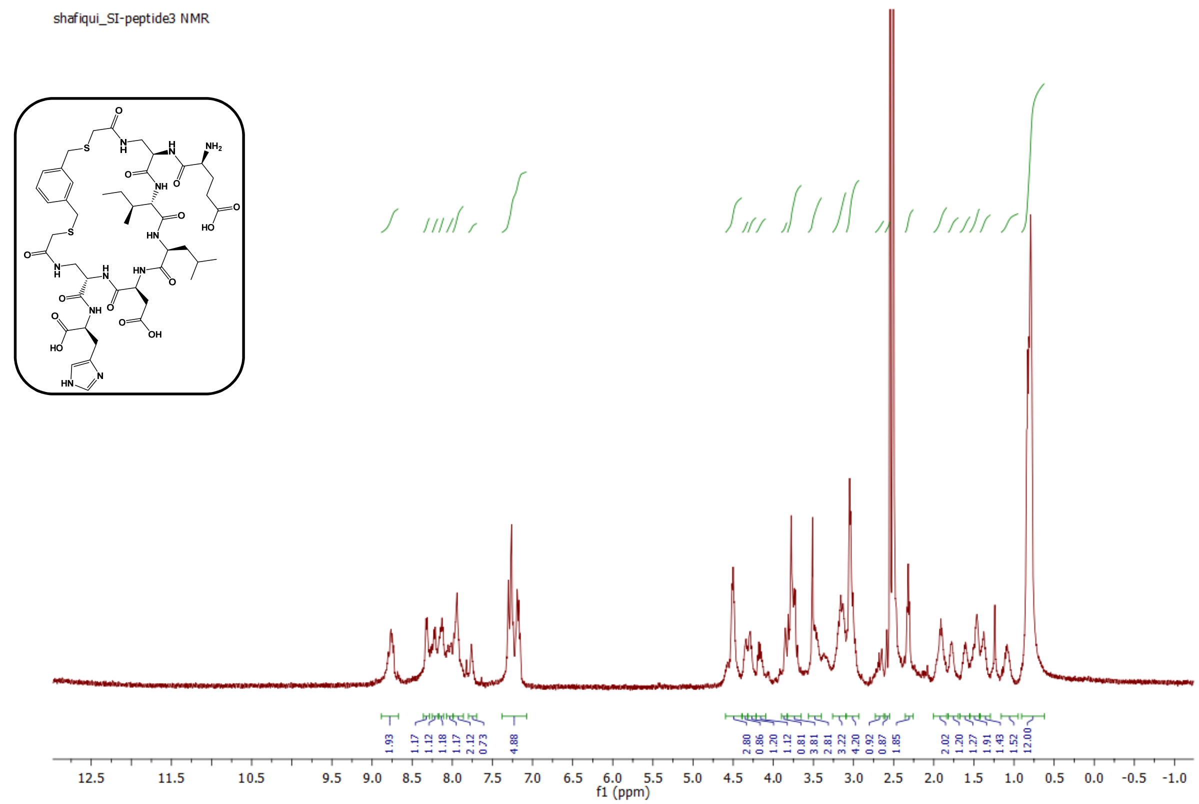

Supplementary Figure 18. ^{1}H NMR spectrum of peptide **59**.

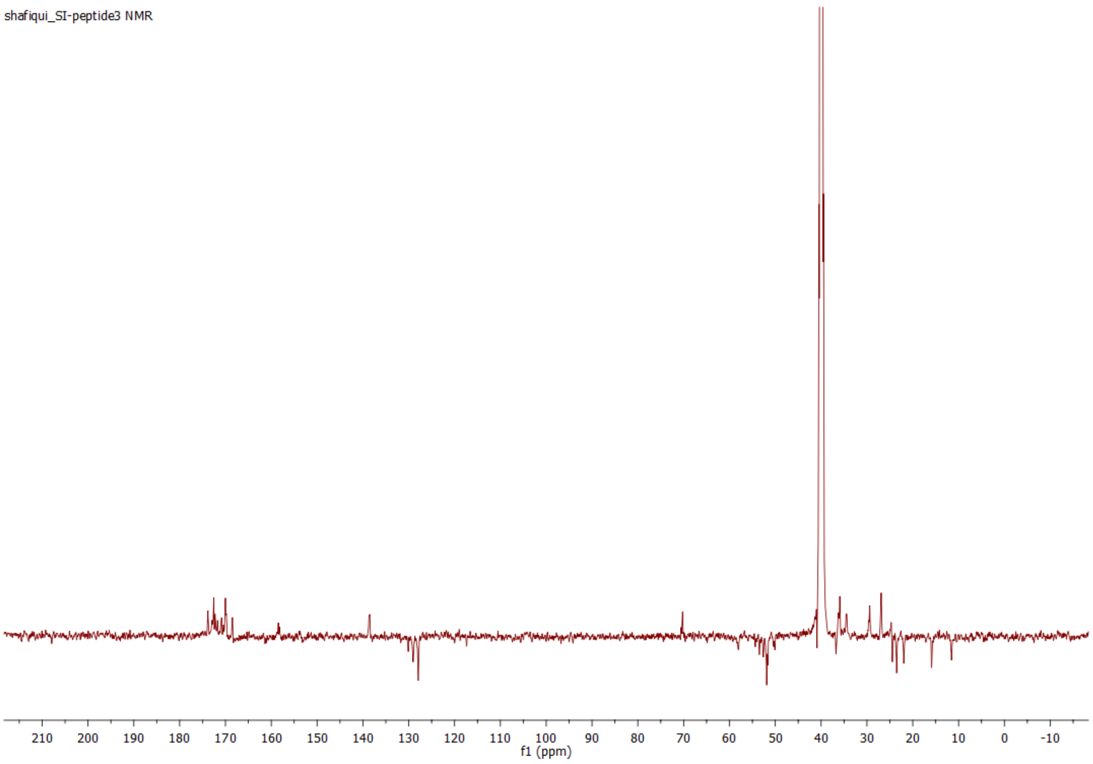

Supplementary Figure 19. APT ^{13}C NMR spectrum of peptide **59**.

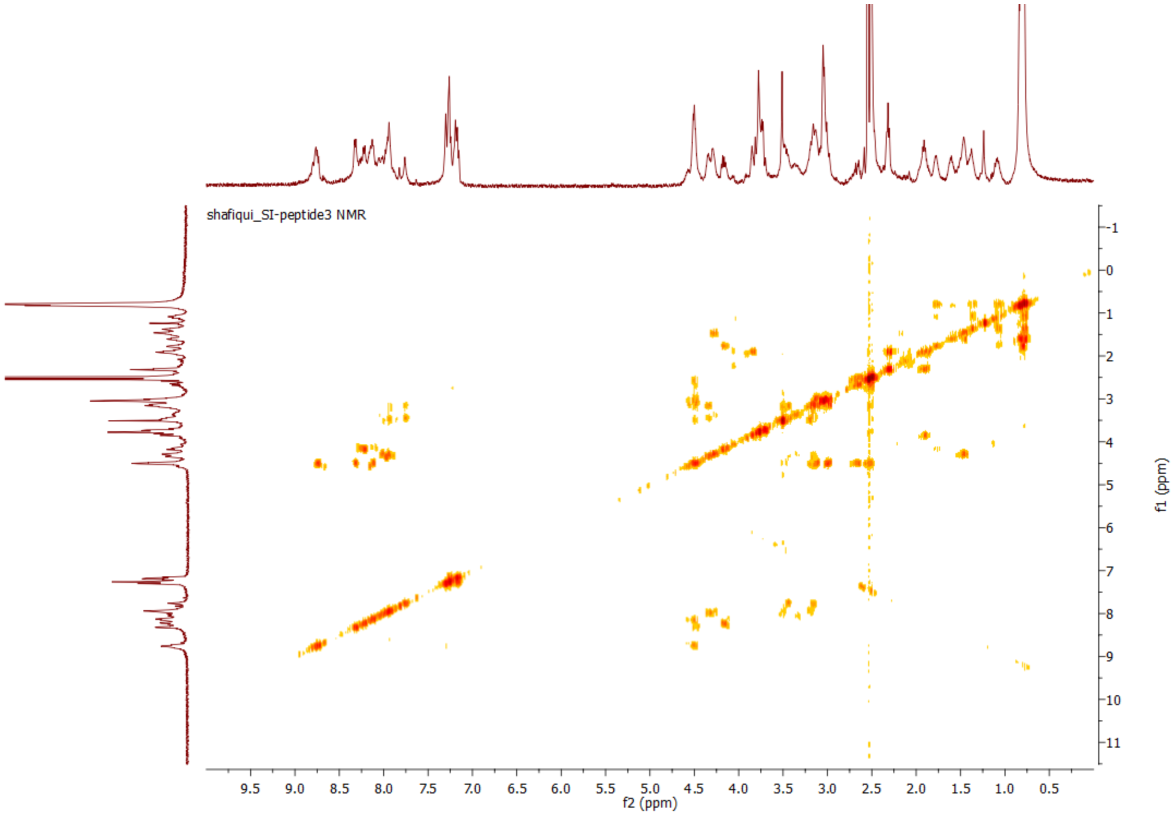

Supplementary Figure 20. COSY NMR spectrum of peptide **59**.

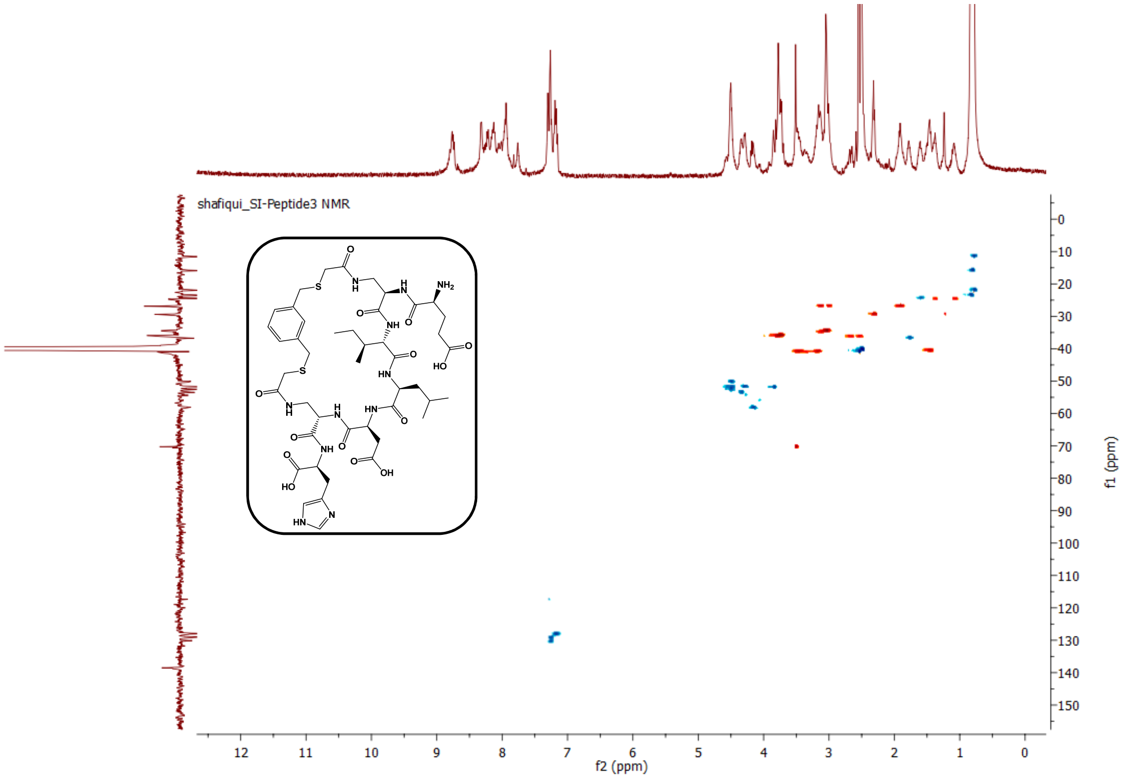

Supplementary Figure 21. ^{1}H-^{13}C HSQC NMR spectrum of peptide **59**.

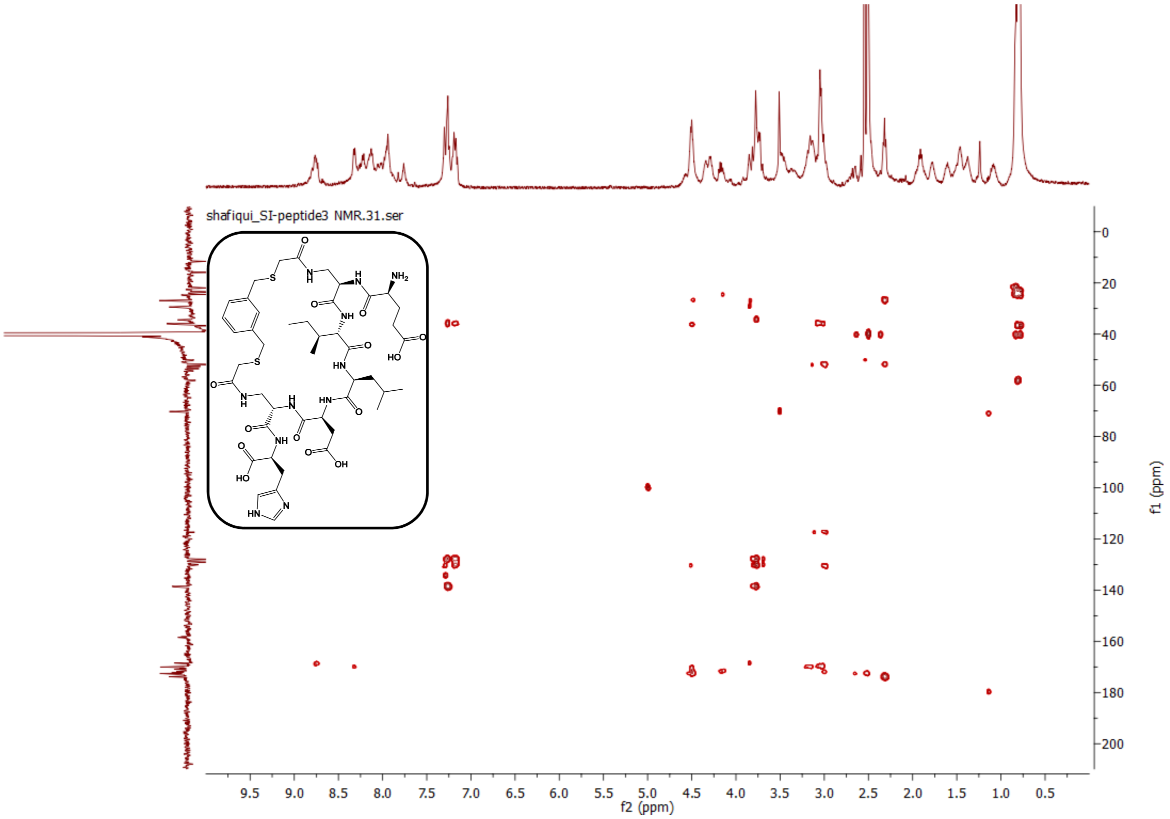

Supplementary Figure 22. ^{1}H-^{13}C HMBC NMR spectrum of peptide **59**.

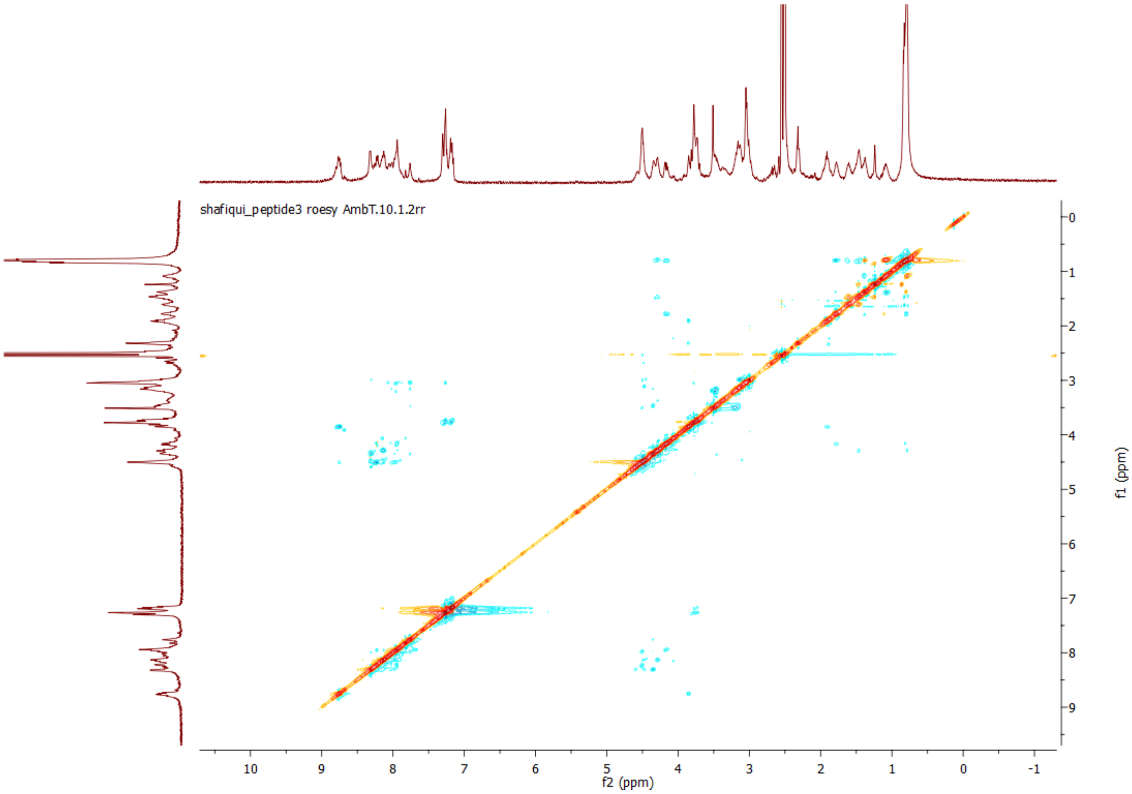

Supplementary Figure 23. ROESY NMR spectrum of peptide **59**.

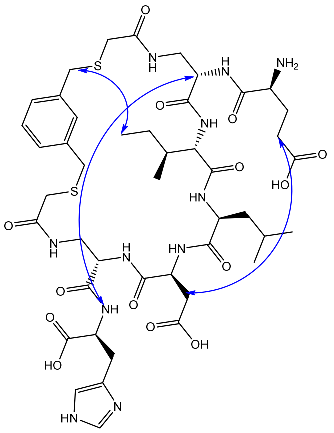

Peptide 57

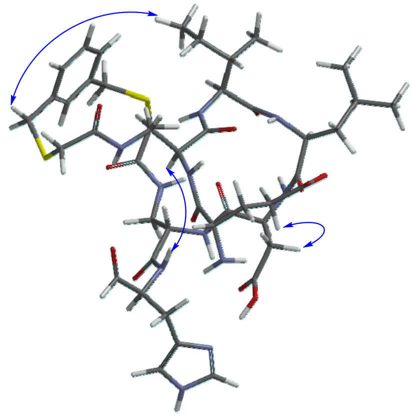

Peptide 57

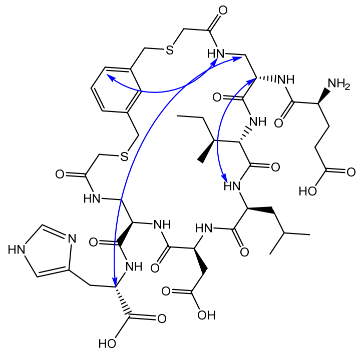

Peptide 58

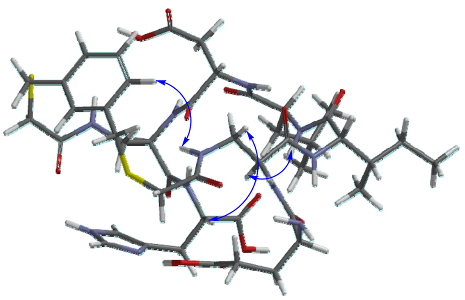

Peptide 58

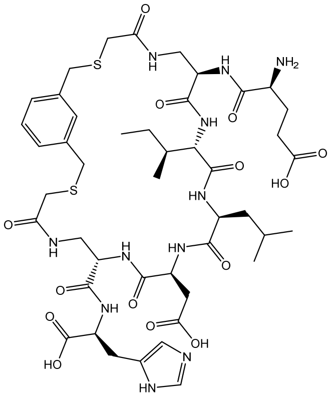

Peptide 59

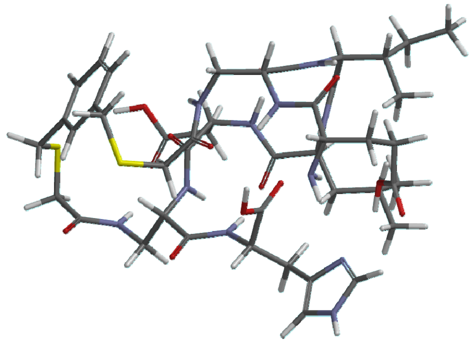

Peptide 59

Supplementary Figure 24. Minimized geometries of peptides **57-59** generated by Spartan'14 suite. Blue arrows indicate the experimentally observed nonsequential NOE connectivities.

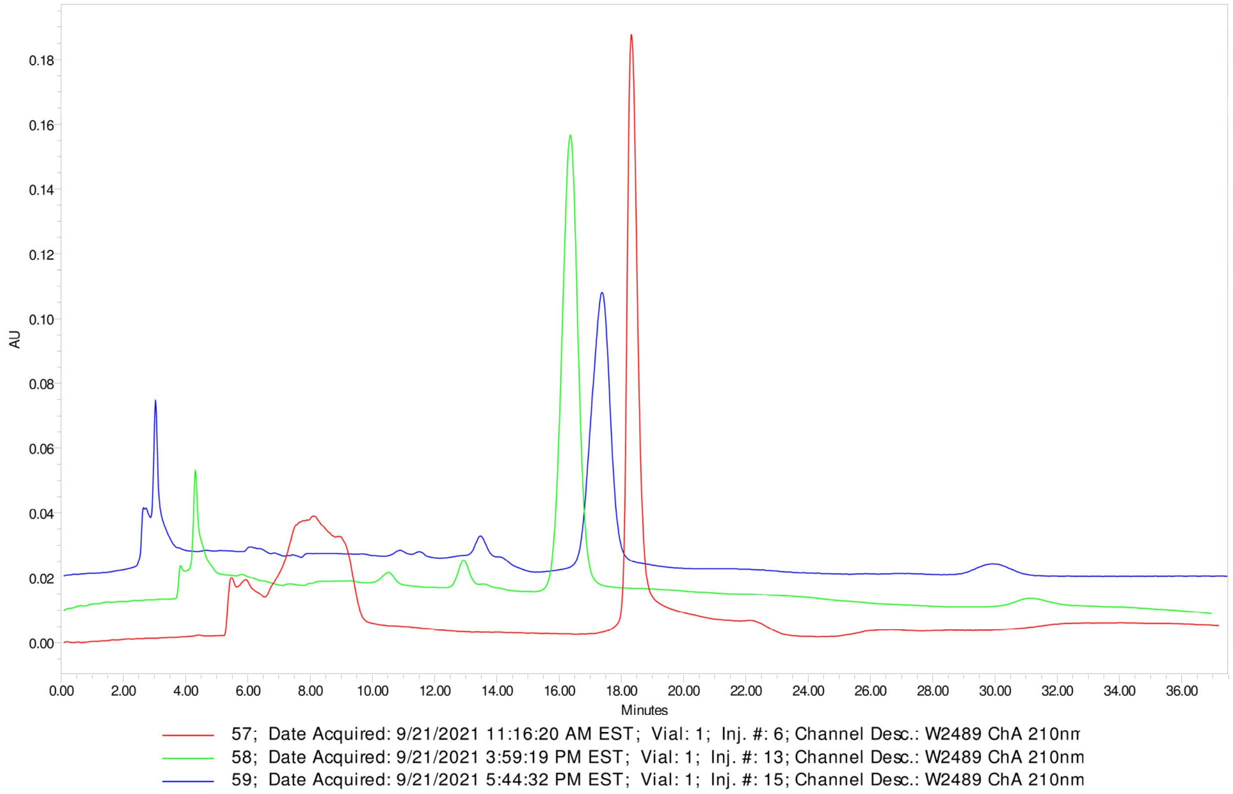

Supplementary Figure 25. Elution of peptides **57-59** on the Astec® CHIROBIOTIC® TAG Chiral HPLC column (5 µm particle size, L x I.D. 15 cm x 4.6 mm). The elution phase was 90% ammonium formate (5 mM, pH 3-3.5) and 10% acetonitrile, with a flow rate of 1 mL/min.

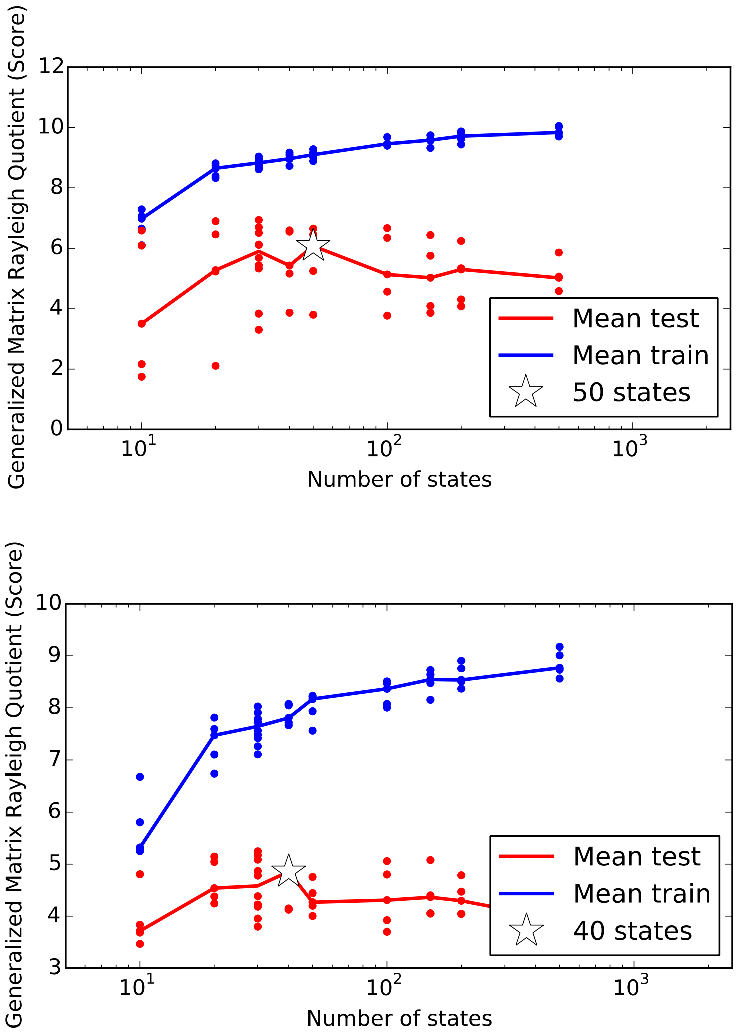

Supplementary Figure 26. Generalized matrix Rayleigh quotient (GMRQ) scores, shown versus the number of states used to construct the MSMs, shown for MSMs of 1,3-benzenedimethanethiol stapled (L,D) Axin peptide (top) and unstapled (L,D) Axin peptide (bottom). Five-fold cross-validation was used, where the MSM was trained on 4/5 of the input data, and the GMRQ score computing using the remaining 1/5 of the data, in five separate trials. While the mean GMRQ score for the training data (blue) continues to increase with the number states, the mean score for the testing data reaches a peak at around 50 states (marked with a star). Based on the general consistency of these results for most of the peptide systems, we chose to construct MSMs using 50 states.

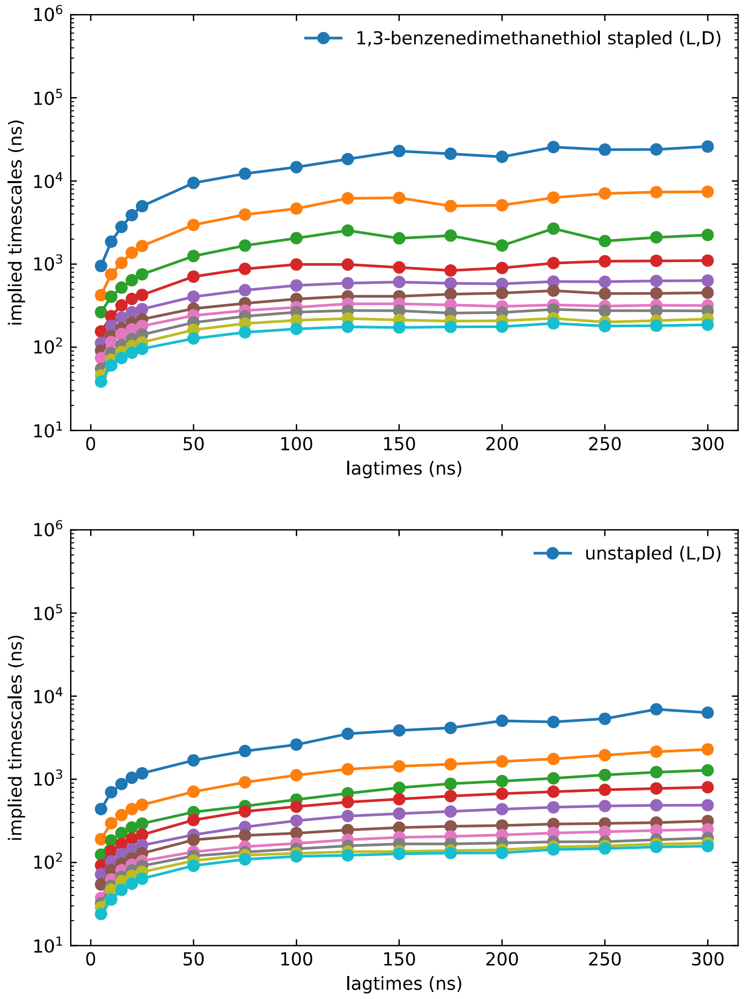

Supplementary Figure 27. Slowest ten implied timescales for MSMs of 1,3-benzenedimethanethiol stapled (L,D) Axin peptide (top) and unstapled (L,D) Axin peptide (bottom), plotted as a function of lagtime, τ. Implied timescales are calculated as $t_i = \tau/\ln \lambda_i$ where λ_i are the eigenvalues of the MSM transition matrix. Uncertainties are estimated using a per-trajectory bootstrap procedure.

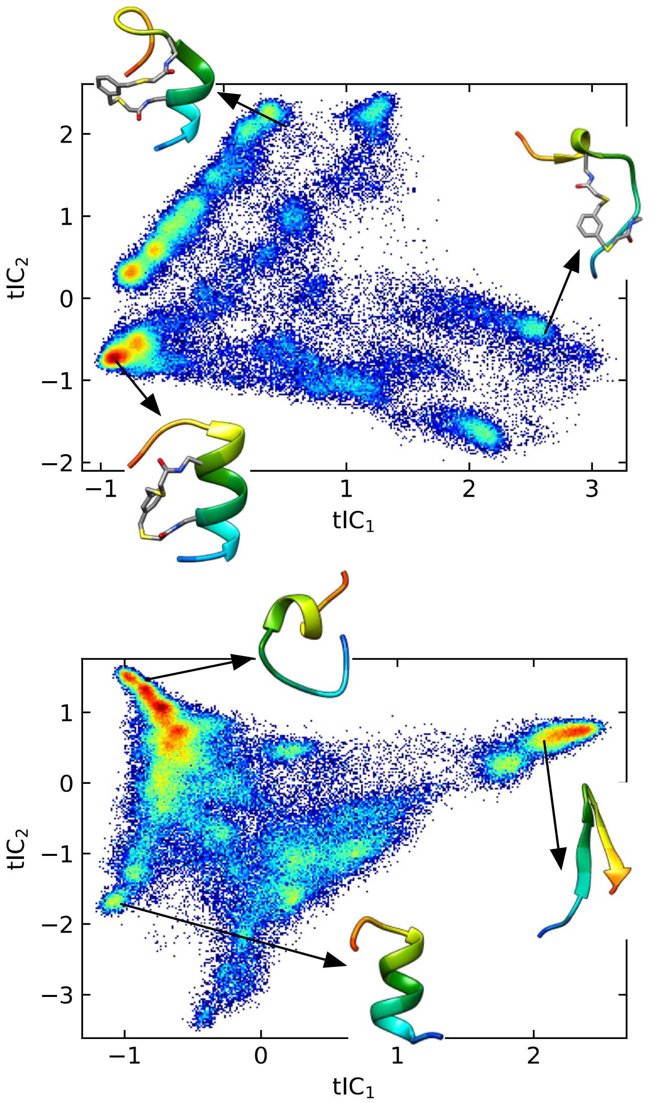

Supplementary Figure 28. Examples of trajectory data projected on the first two tICs, with representative structures, for 1,3-benzenedimethanethiol stapled (L,D) Axin peptide (top), and unstapled (L,D) Axin peptide (bottom). The color scale represents regions of high (red) to low (blue) density.

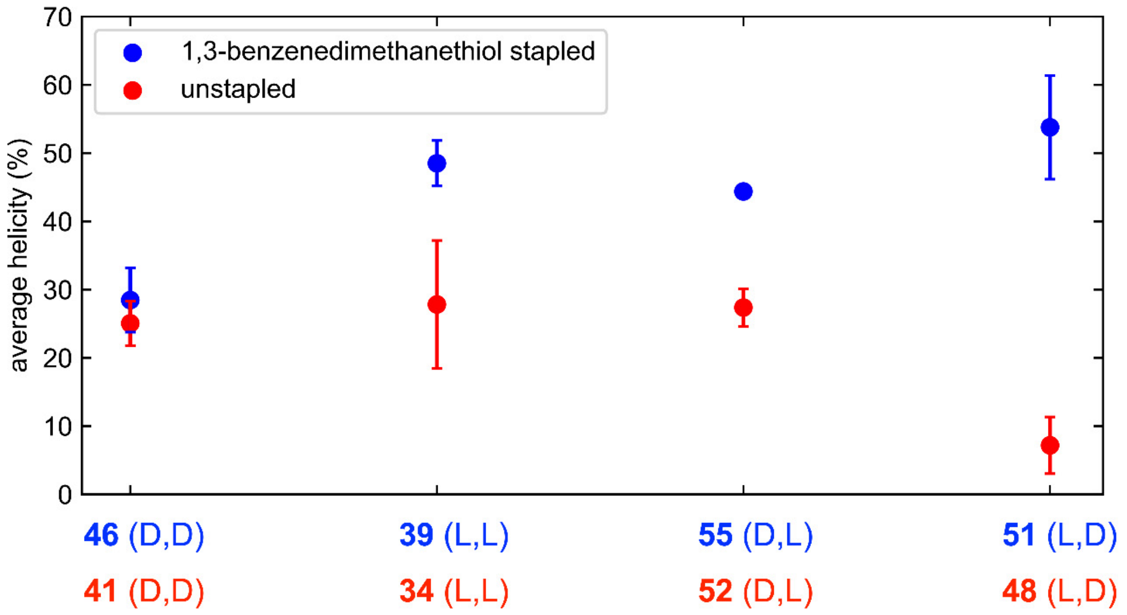

Supplementary Figure 29. Comparison between the helicities of 1,3-benzenedimethanethiol stapled Axin peptides (blue circles) and unstapled Axin peptides (red circle). Uncertainties (vertical bars) were calculated as SEM using a bootstrap procedure in which different MSMs (n=5) were constructed by sampling the input trajectory data with replacement.

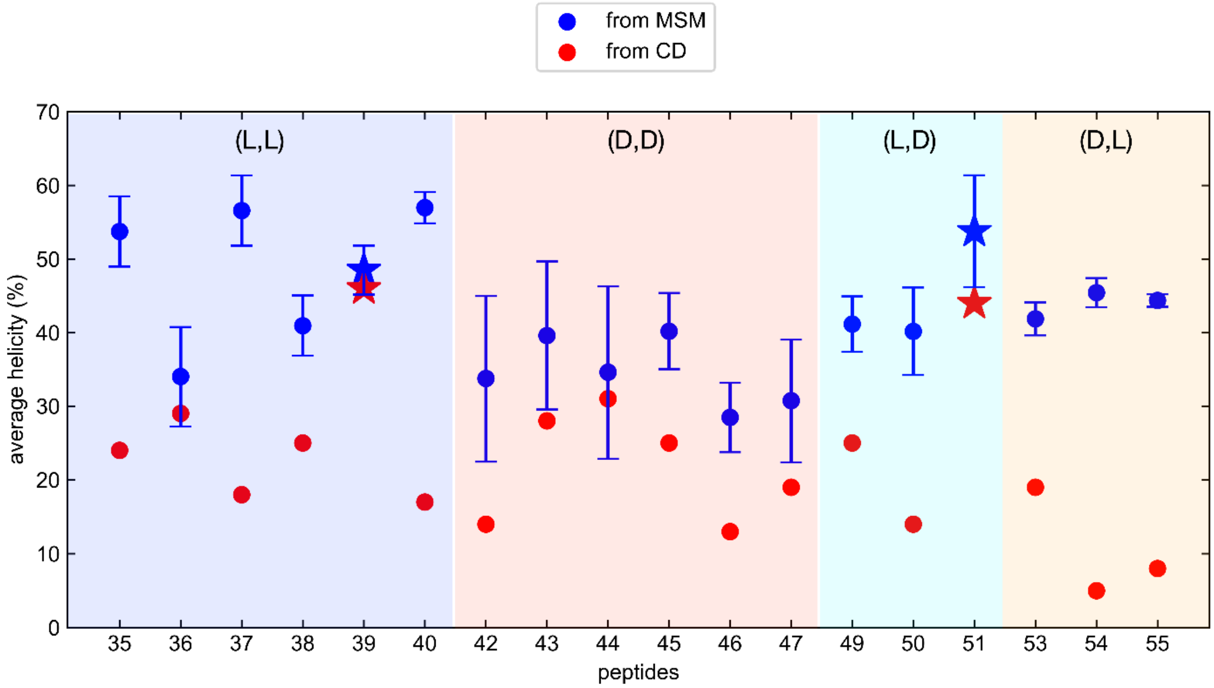

Supplementary Figure 30. Comparison of predicted (blue) and experimental (red) helicties for stapled Axin peptides **35-40**, **42-47**, **49-51**, and **53-55**. Peptides **39** and **51** are labeled with star makers to denote the highest experimentally measured helicities. Predicted data are presented as mean values ± SEM (vertical bars) calculated using a bootstrap procedure in which n=5 different MSMs were constructed by sampling the input trajectory data with replacement.

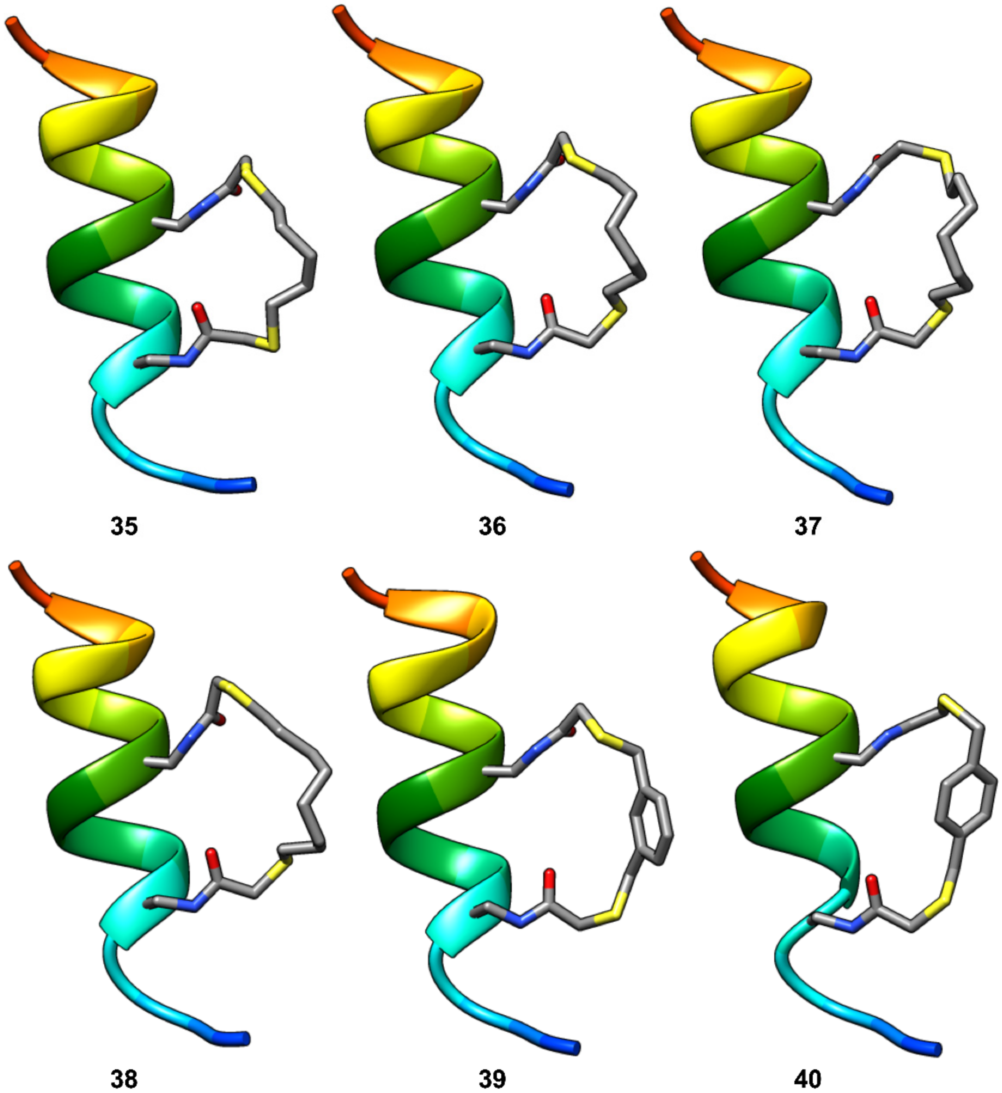

Supplementary Figure 31. Minimized structures of stapled Axin peptides crosslinked at the *i*, *i*+4 positions.

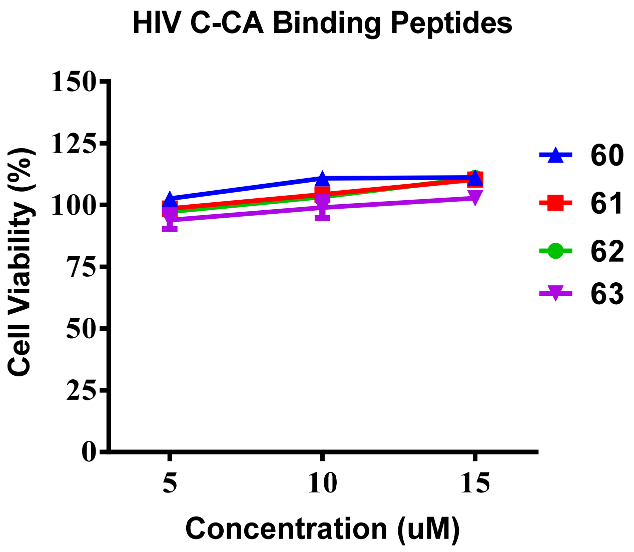

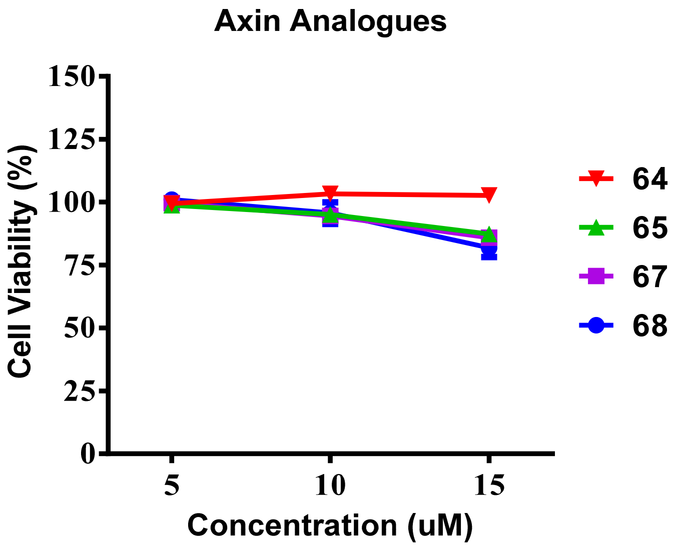

Supplementary Figure 32. Viability of cells after treatment with FITC-labeled peptide analogues. HEK293T cells were treated with HIV C-CA binding peptides, while DLD-1 cells were treated with Axin analogues at doses of 5, 10, or 15 µM. In each set, the cells treated with solvent only were normalized as the 100% control. Data represent mean values ± SD from n=3 biologically independent sample measurements.

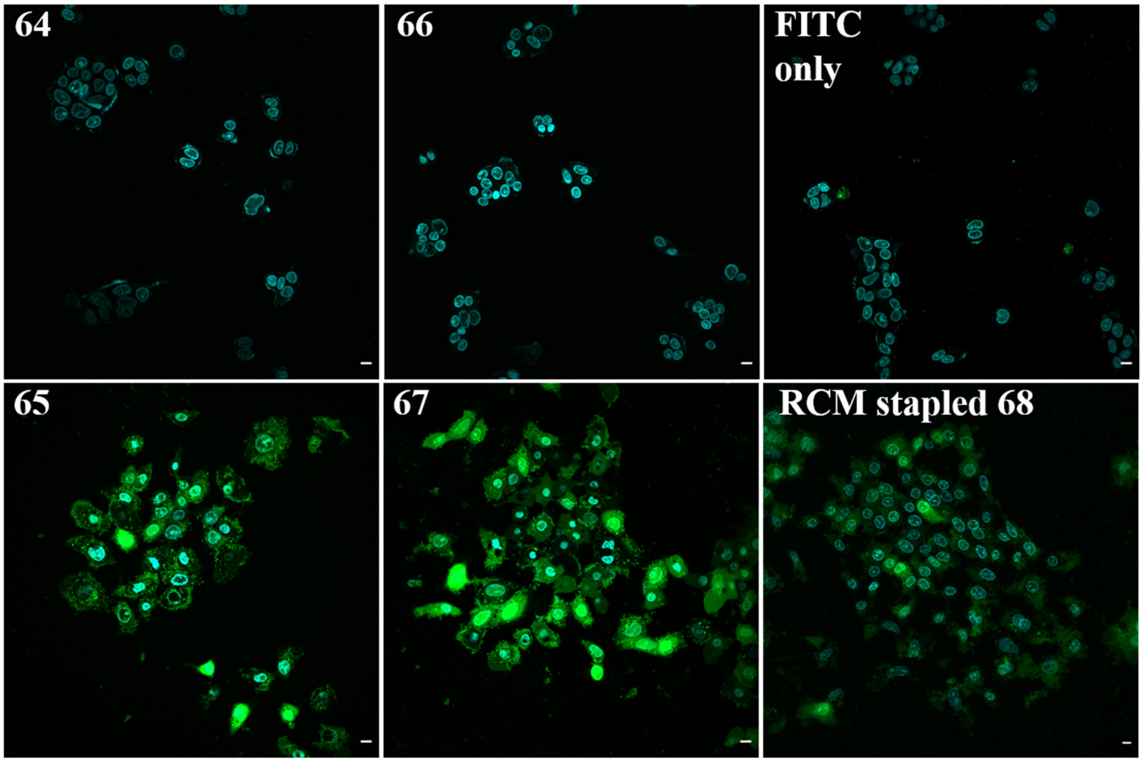

Supplementary Figure 33. Raw imaging data for DLD-1 cells treated with 10 μM of FITC-labeled Axin analogues. Scale bar: 5μm.

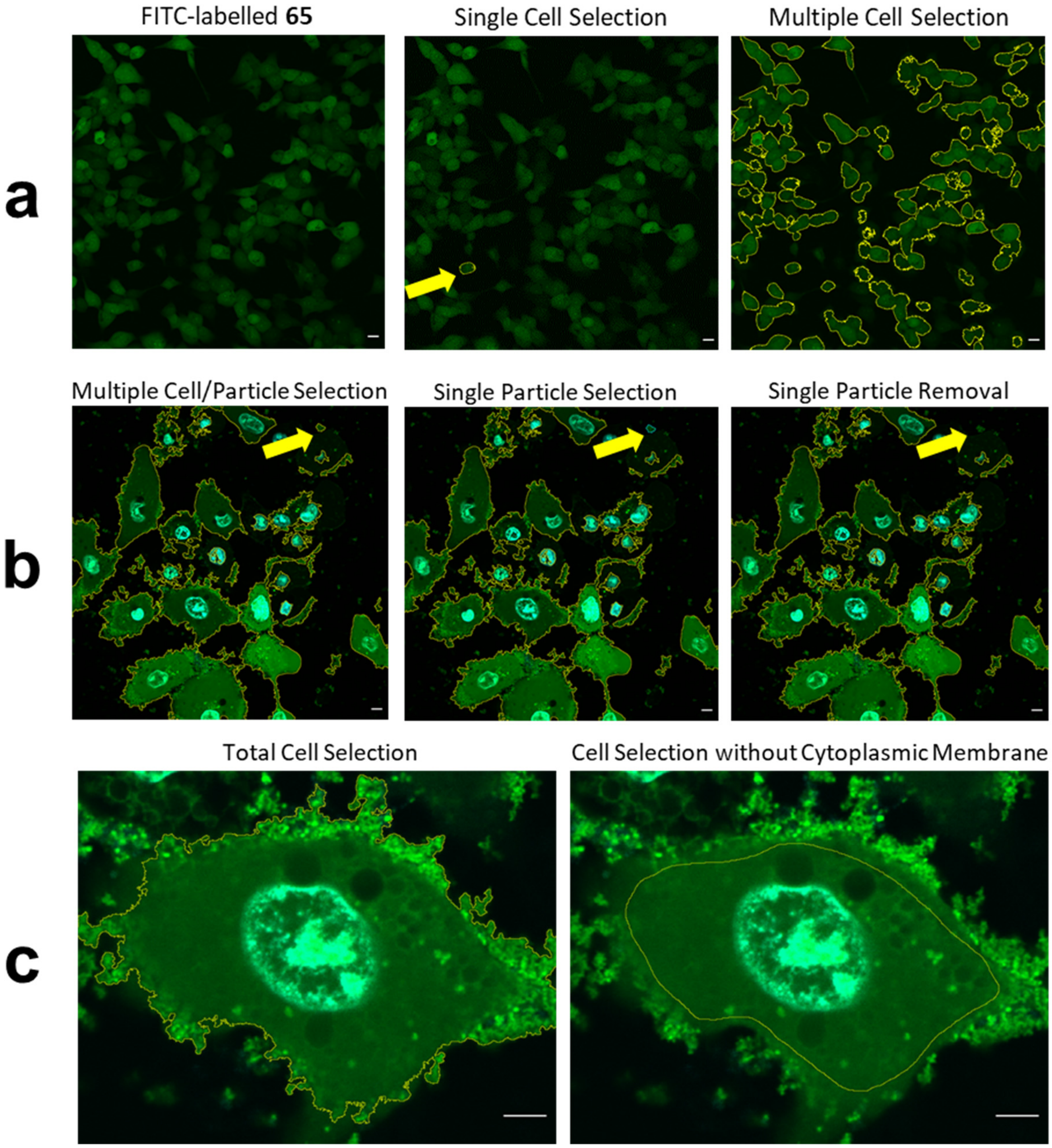

Supplementary Figure 34. Representative cell selection for FITC-labeled Axin analogue **65** using the NIH ImageJ analyze particles function. **a** Single and multiple cell selection for the FITC-labeled peptide. **b** Extracellular particle selection and removal. **c** The removal of the cytoplasmic membrane from the mean intensity measurement. Scale bar: 5μm.

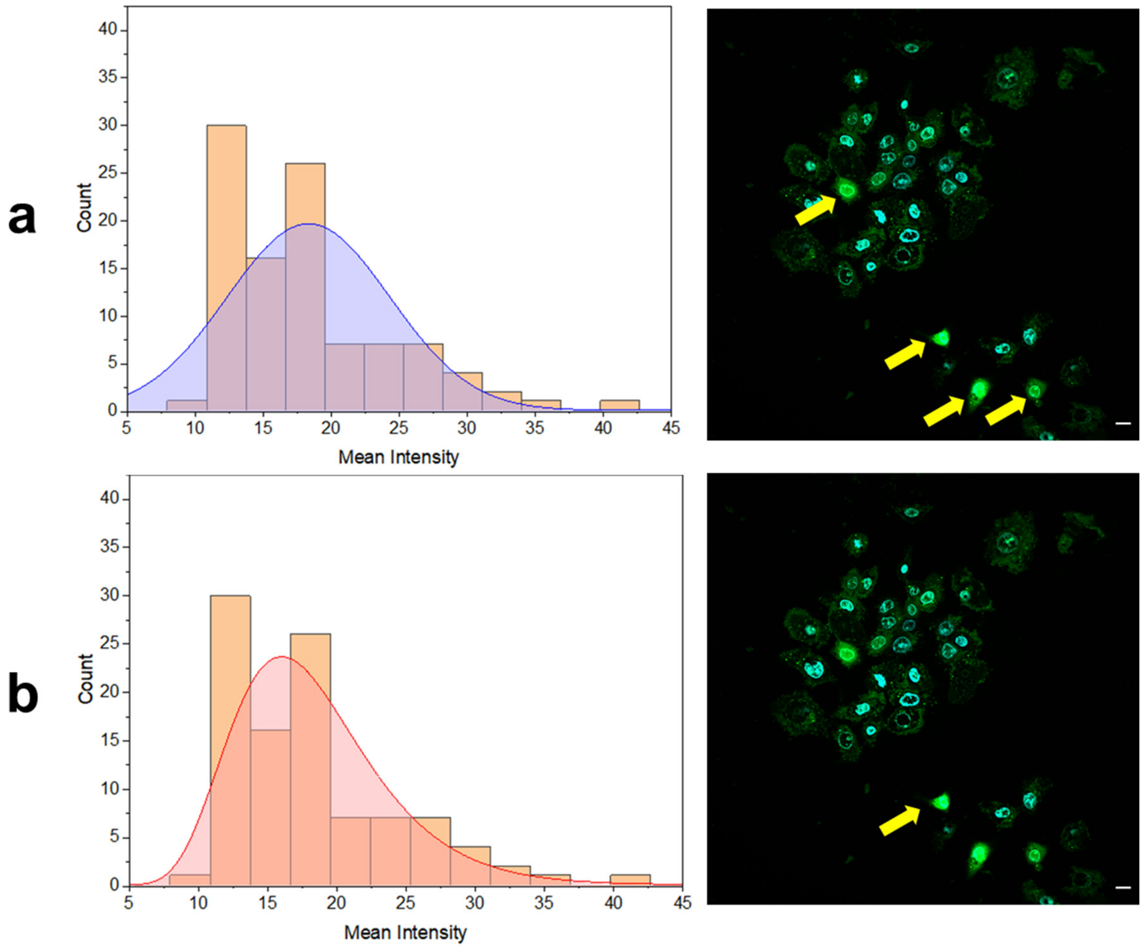

Supplementary Figure 35. Outlier exclusion for cells treated with FITC-labeled Axin analogue **65**. **a** Histogram of the mean intensities of the FITC-labeled peptide with a normal distribution curve applied. With a normal distribution, four cells (yellow arrows) with values 31.13, 32.41, 35.64, and 42.38 is excluded from the data set. **b** Histogram of the mean intensities of the FITC-labeled peptide with the best fit, lognormal distribution curve applied. With a lognormal distribution, one cell (yellow arrow) with a value 42.38 is excluded from the data set. Scale bar: 5μm.

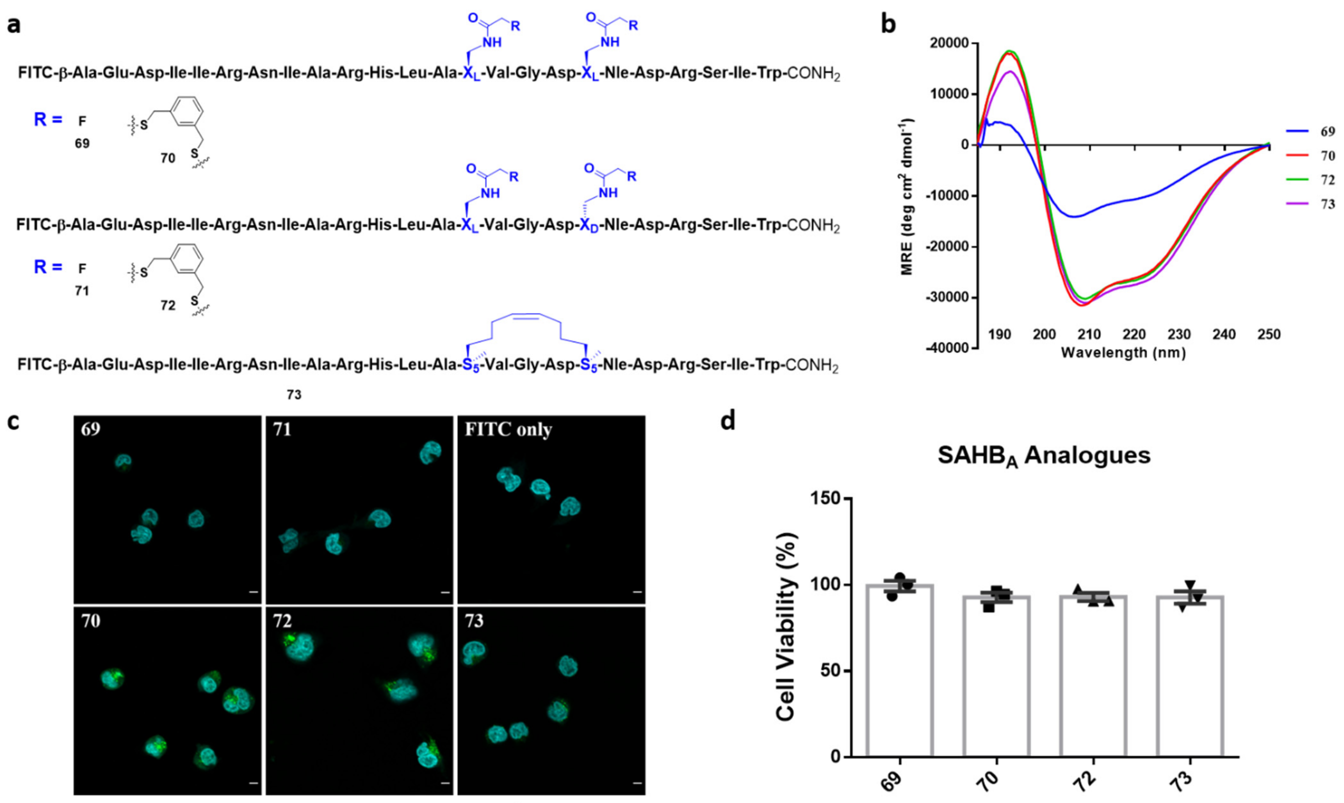

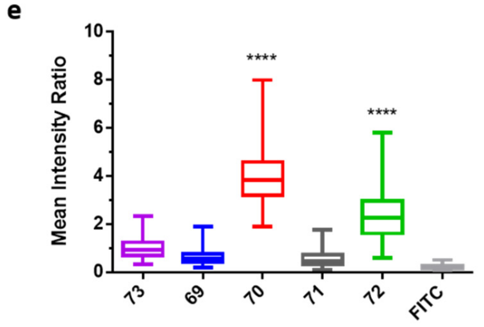

f

Peptide	Mean Intensity Ratio	N (cell number)	Peptide	Mean Intensity Ratio	N (cell number)
**73***	1.00 ± 0.03	151	**71**	0.56 ± 0.03	133
**69**	0.66 ± 0.03	125	**72**	2.41 ± 0.09	132
**70**	4.05 ± 0.11	129	**FITC only**	0.22 ± 0.01	123

*: Control peptides stapled by ring-closing metathesis.

Supplementary Figure 36. FTDR-based stapling of stabilized alpha-helix of BCL-2 domains (SAHBA) peptide analogues. **a** Chemical structures of peptides **69-72.** The reported SAHBA peptide **73** was stapled by RCM. **b** CD spectra of the SAHBA peptide analogues. **c** Fluorescent confocal microscopy images of the Jurkat cells treated with peptides **69-73**. Blue: nucleus stained by Hoechst 33342 ; Green: FITC-labeled peptides; Scale bar: 5μm. **d** Viability of Jurkat cells after imaging studies with SAHBA peptide analogues. The cells only treated with solvent were normalized as the control (100%). Data represent mean ± SEM for three independent repeats. **e** Quantification analysis of the cell penetration of peptides **69-73**. The mean intracellular intensity of RCM-stapled peptide **73** was normalized as 1. Box plots show 25%-75% quantiles, median values (bullseye), and minimal and maximal values (whiskers). Two-sided unpaired t test with Welch's correction has been applied. "****" represents $p < 0.0001$. **f** Table summary of the quantification (mean ± SEM) of the peptides' cell penetration and sample sizes (N).

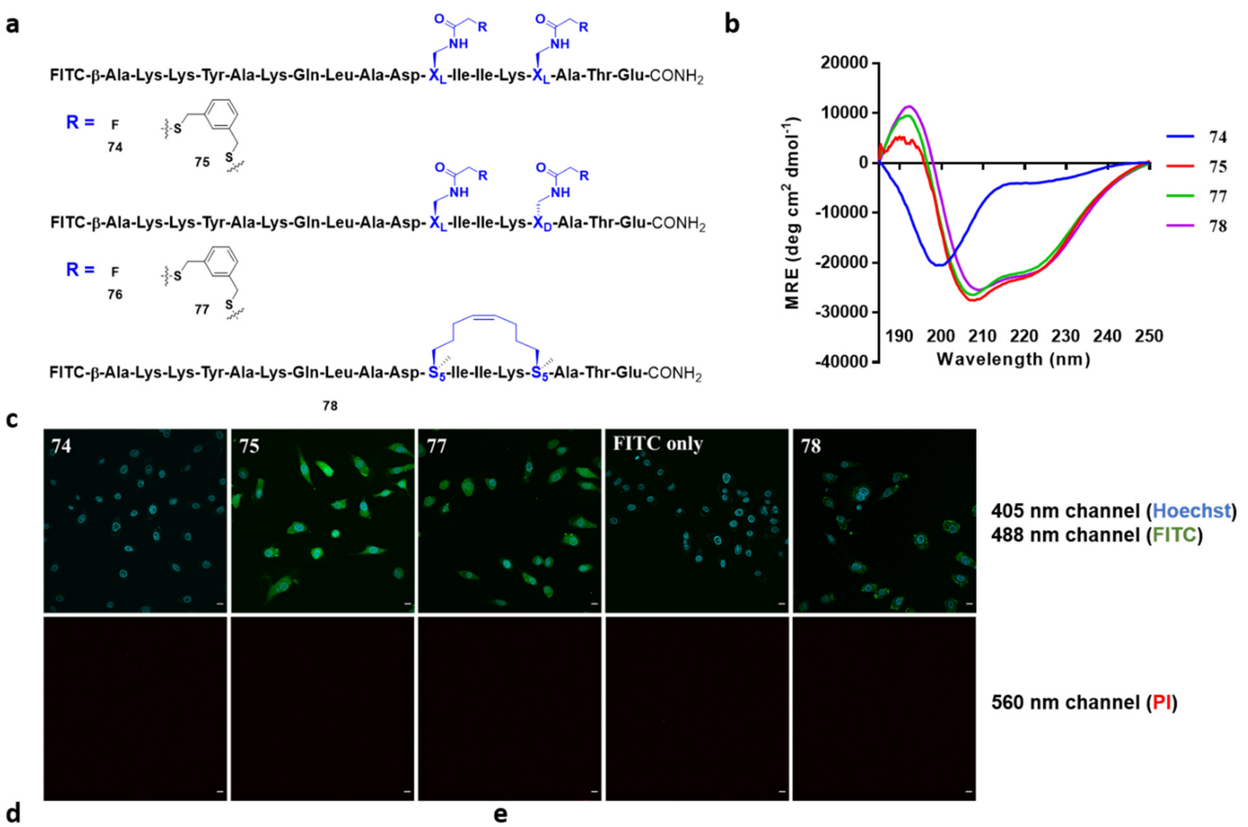

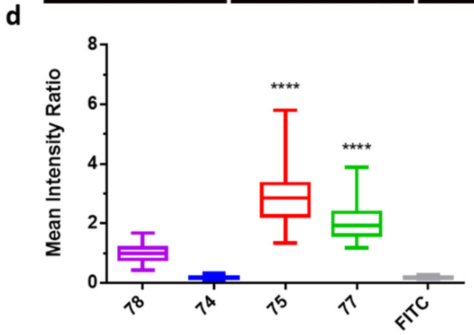

e

Peptide	Mean Intensity Ratio	N (cell number)	Peptide	Mean Intensity Ratio	N (cell number)
**78***	1.00 ± 0.02	149	**77**	2.00 ± 0.05	125
**74**	0.18 ± 0.004	124			
**75**	2.92 ± 0.08	125	**FITC only**	0.185 ± 0.002	139

*: Control peptides stapled by ring-closing metathesis.

Supplementary Figure 37. FTDR-based stapling of A-kinase targeting Stapled Anchoring Disruptors (STAD) peptide analogues. **a** Chemical structures of peptides **74-77.** The reported STAD peptide **78** (stapled by RCM) was prepared as well. **b** CD spectra of the related STAD peptide analogues. **c** Fluorescent confocal microscopy images of HeLa cells treated with STAD peptide analogues. Blue: nucleus stained by Hoechst 33342 ; Green: FITC-labeled peptides. Red: Propidium Iodide (PI) staining of dead cells; Scale bar: 5µm. **d** Quantification analysis of the cell penetration of STAD peptides **74-75, 77-78**. The mean intracellular intensity of the RCM-stapled peptide **78** was normalized as 1. Box plots show 25%-75% quantiles, median values (bullseye), and minimal and maximal values (whiskers). Two-sided unpaired t test with Welch's correction has been applied. "****" represents $p < 0.0001$. **e** Table summary of the quantification (mean ± SEM) of the peptides' cell penetration and sample sizes (N).

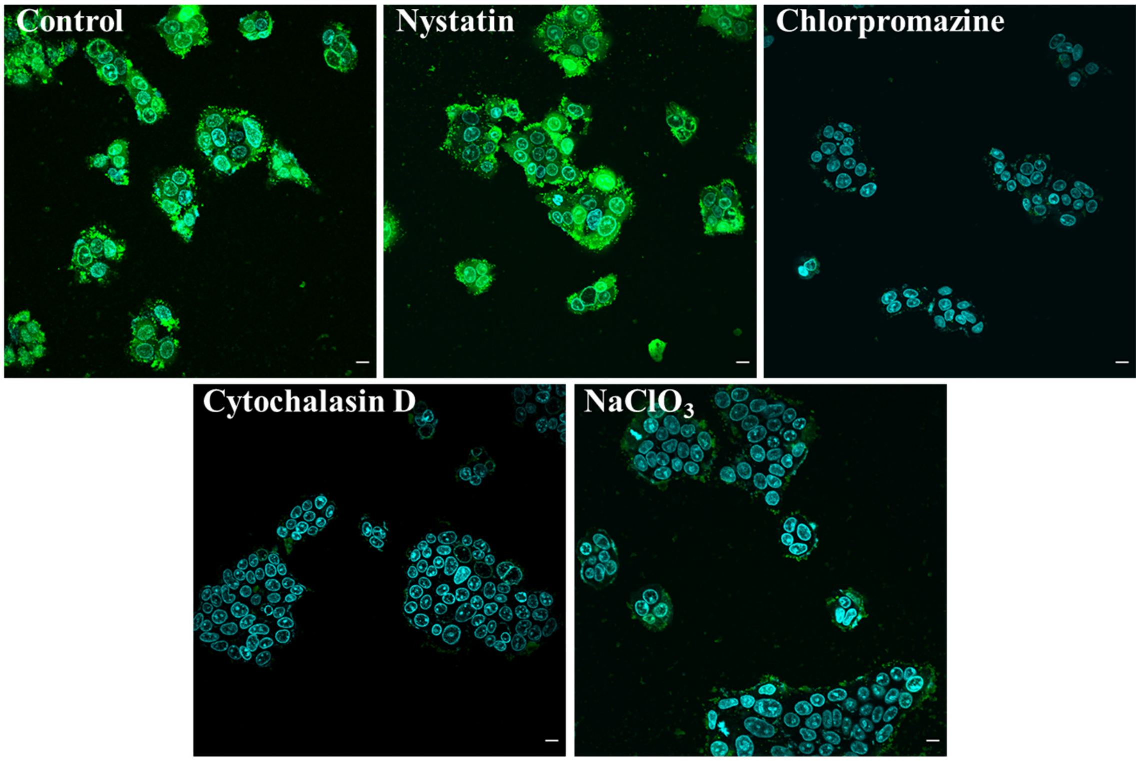

Supplementary Figure 38. Fluorescent confocal microscopy images of the DLD-1 cells treated with the stapled Axin analogue **65** in the presence of different endocytotic pathway blockers (Nystatin, Chlorpromazine, Cytochalasin D, and $NaClO_3$). Green: FITC-labeled peptide **65**; Blue: nucleus stained by Hoechst 33342. Control: cells pretreated with vehicle control and then incubated with peptide **65**. Scale bar: 5μm.

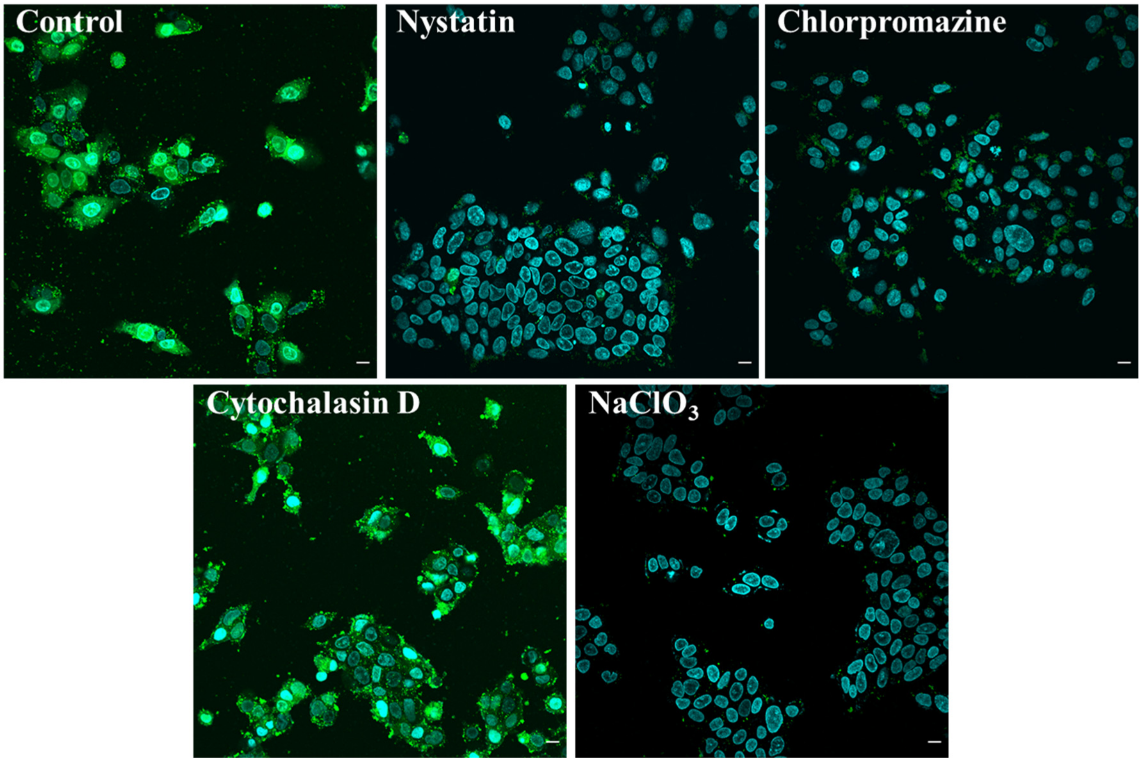

Supplementary Figure 39. Fluorescent confocal microscopy images of the DLD-1 cells treated with the stapled Axin analogue **67** in the presence of different endocytotic pathway blockers (Nystatin, Chlorpromazine, Cytochalasin D, and $NaClO_3$). Green: FITC-labeled peptide **67**; Blue: nucleus stained by Hoechst 33342. Control: cells pretreated with vehicle control and then incubated with peptide **67**. Scale bar: 5μm.

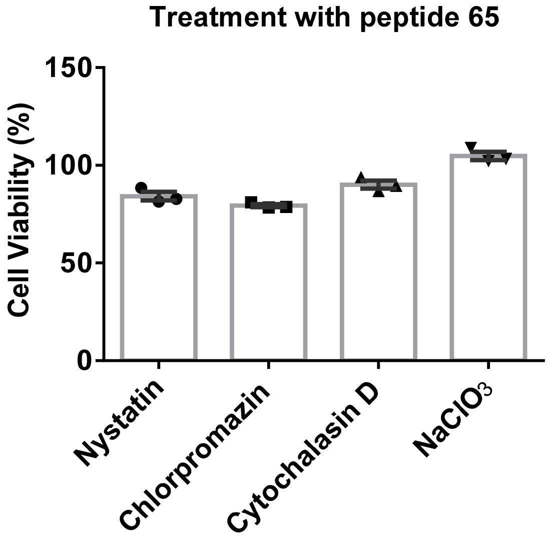

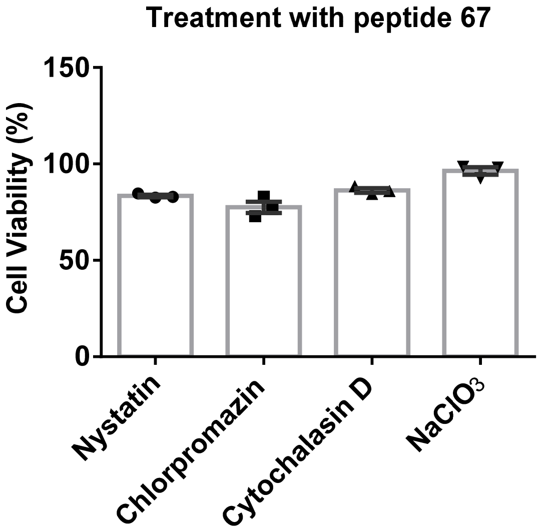

Supplementary Figure 40. Viability of DLD-1 cells after imaging studies with representative stapled Axin analgoues (**65** or **67**). Cells were pre-treated with different small molecule blockers of endocytic pathways before imaging studies. The viability of cells treated with only vehicle control were normalized as 100%. Data represent mean values ± SEM from n=3 biologically independent sample measurements.

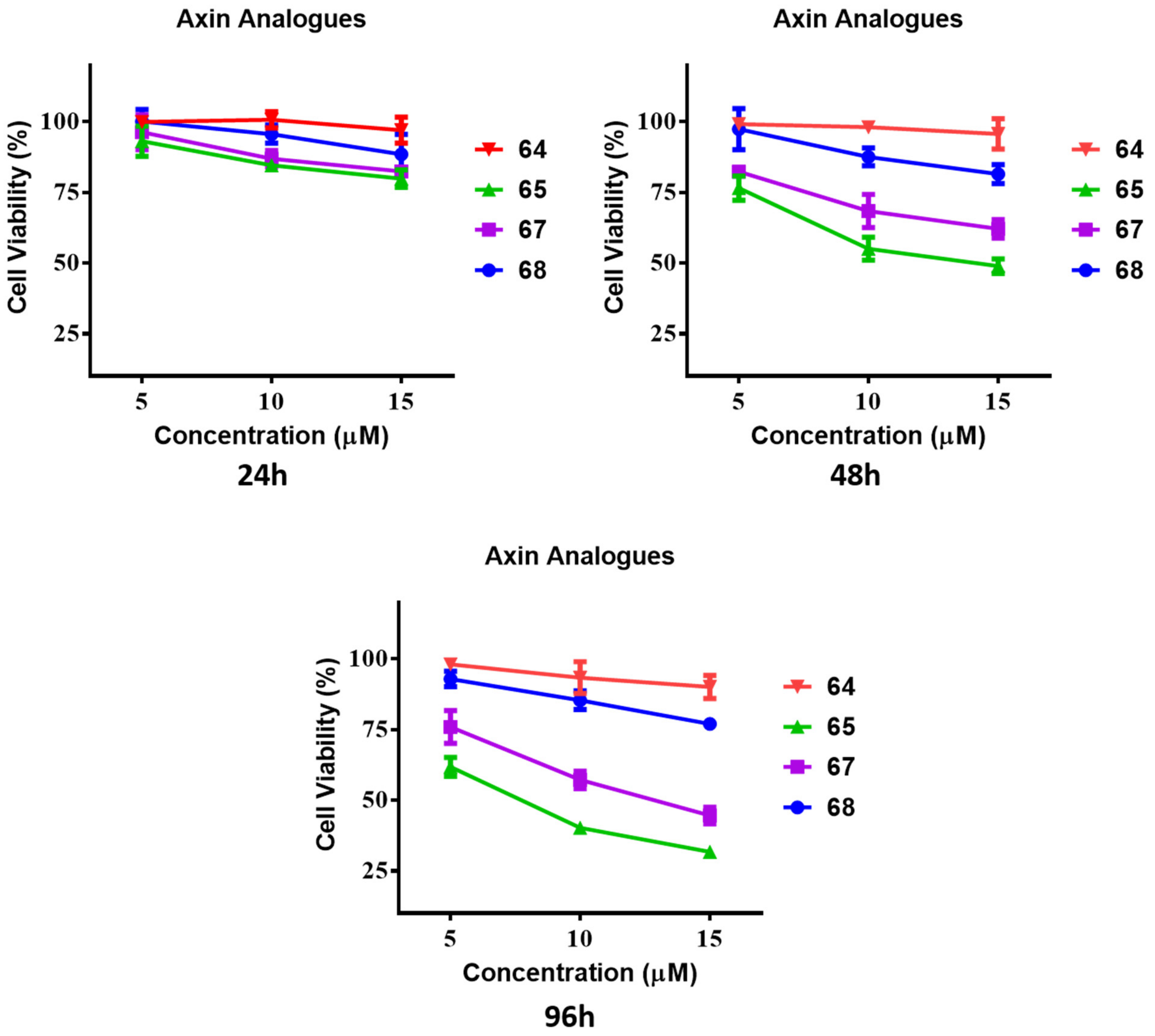

Supplementary Figure 41. Time-dependent cytotoxicity (24h) and/or growth inhibition (48h, 96h) of Axin peptide analogues (**64**, **65**, **67**, **68**) on DLD-1 colon cancer cells. Data represent mean values ± SD from n=3 biologically independent sample measurements.

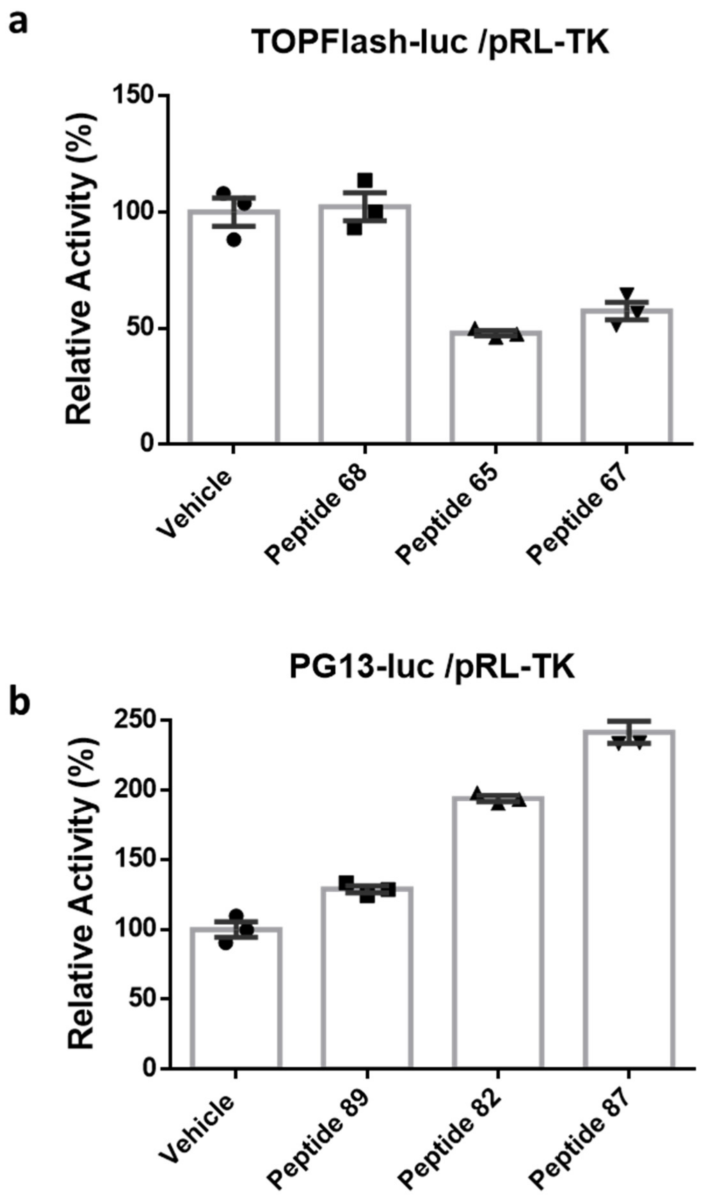

Supplementary Figure 42. Luciferase reporter assays. **a** Wnt-responsive TOPFlash luciferase reporter assay results of Axin peptide analogues (15 μM final concentration) in DLD-1 cells. **b** p53-responsive PG13 luciferase reporter assay results of p53 peptide analogues (15 μM final concentration) in HCT116 wt cells. Data represent mean values ± SEM from three biologically independent sample measurements.

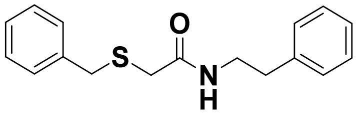

2

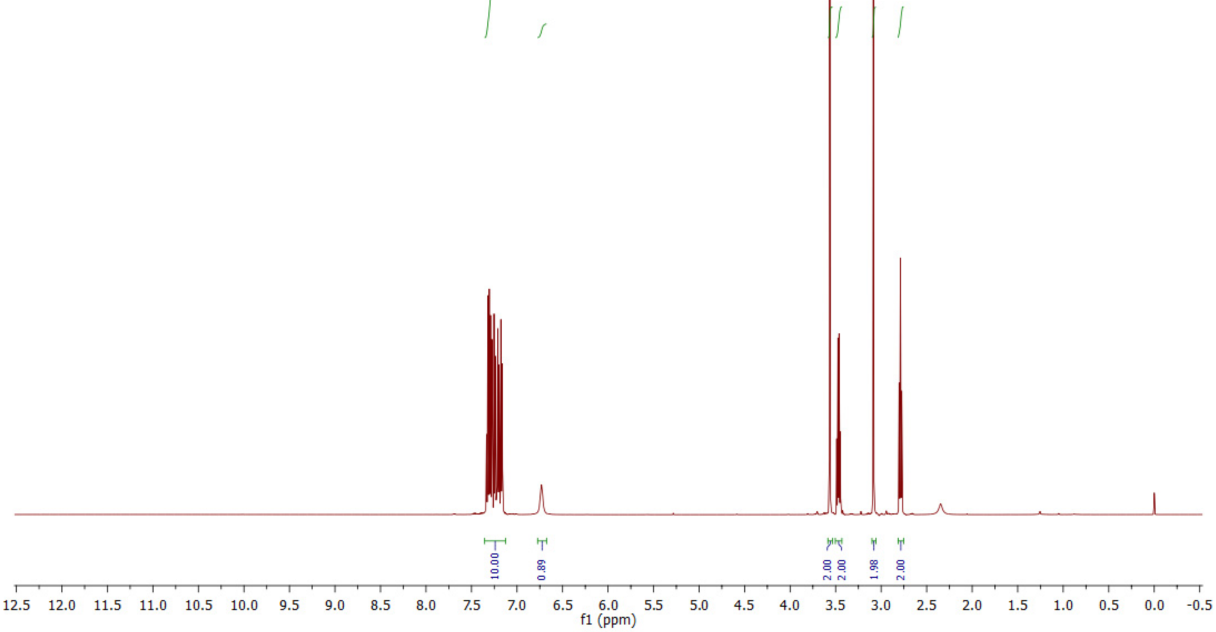

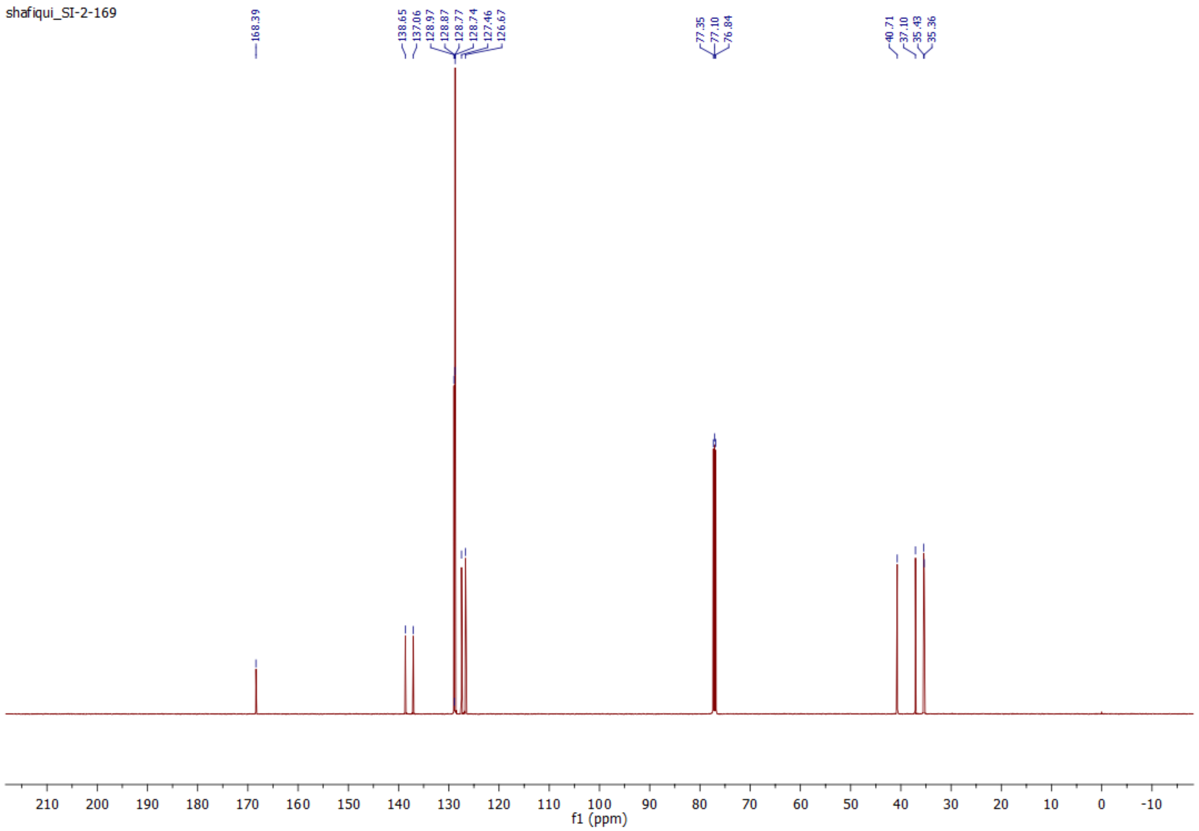

Supplementary Figure 43. ^{1}H- and ^{13}C- NMR Spectra for compound **2**.

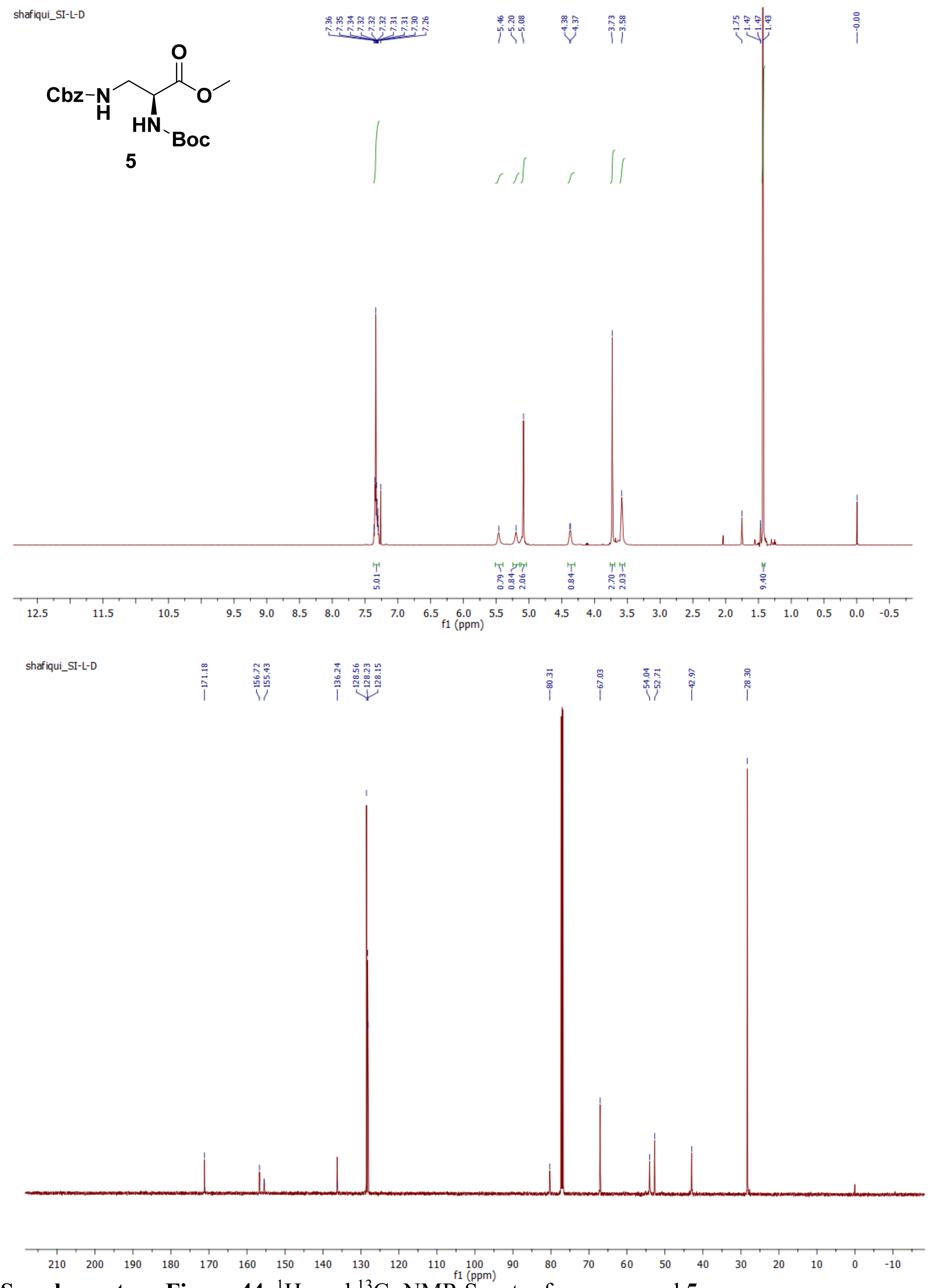

Supplementary Figure 44. ^{1}H- and ^{13}C- NMR Spectra for compound **5**.

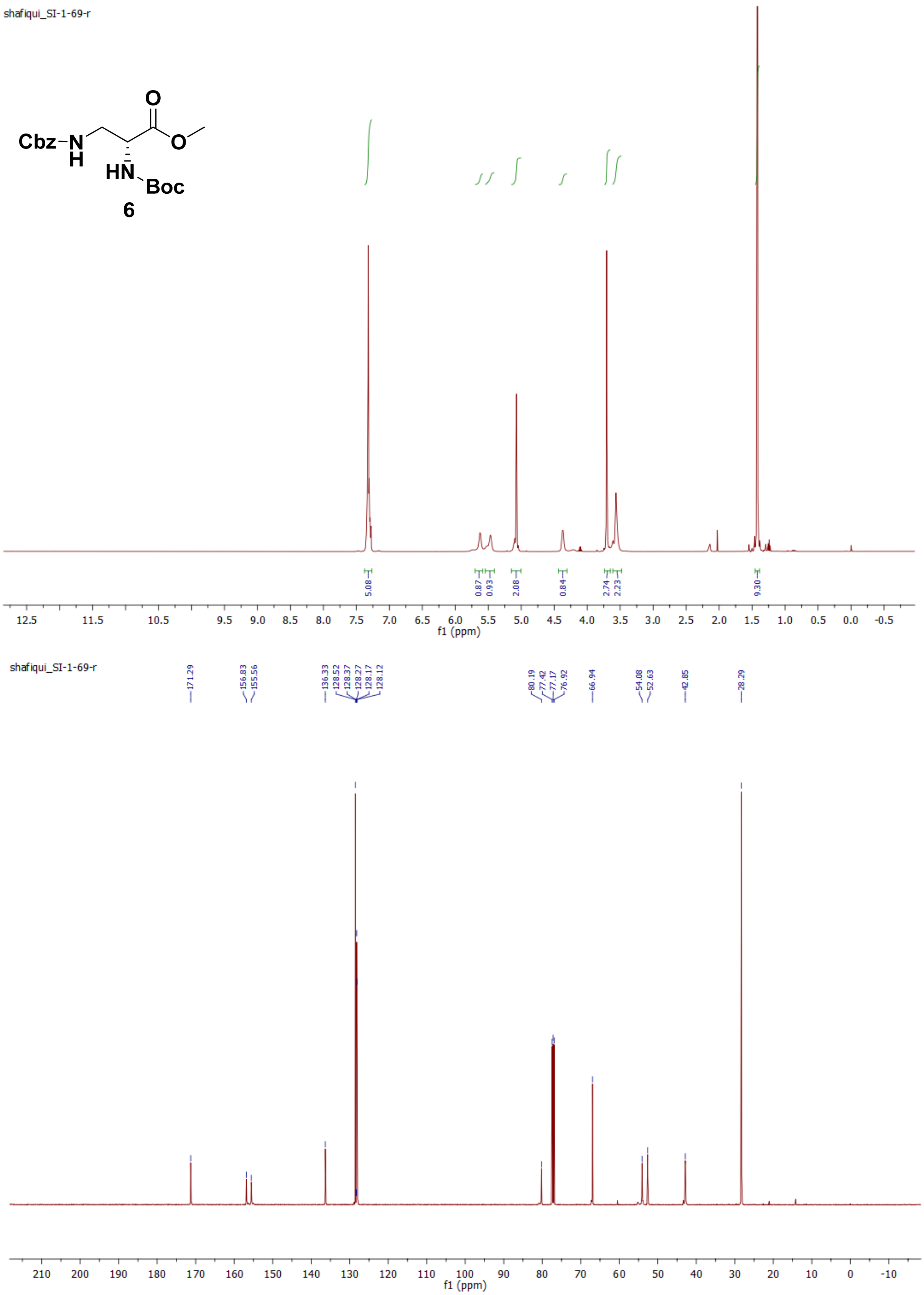

Supplementary Figure 45. ^{1}H- and ^{13}C- NMR Spectra for compound **6**.

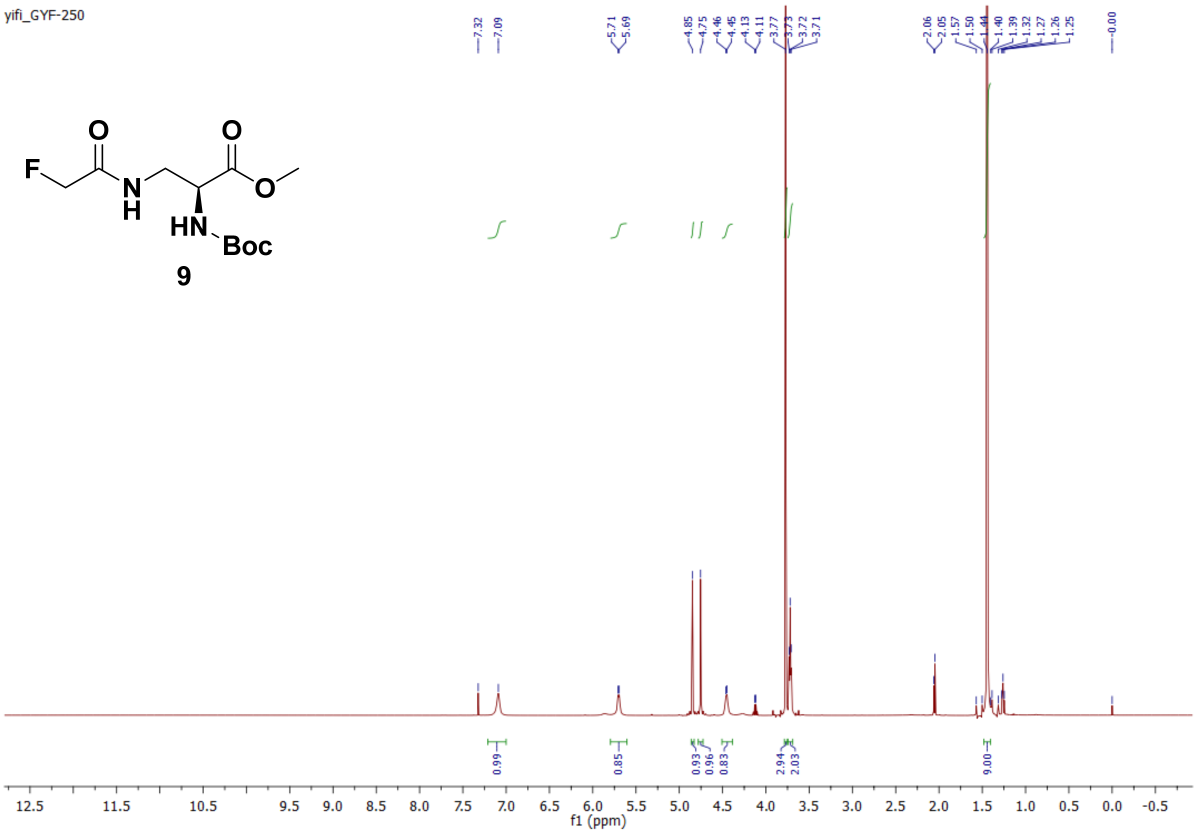

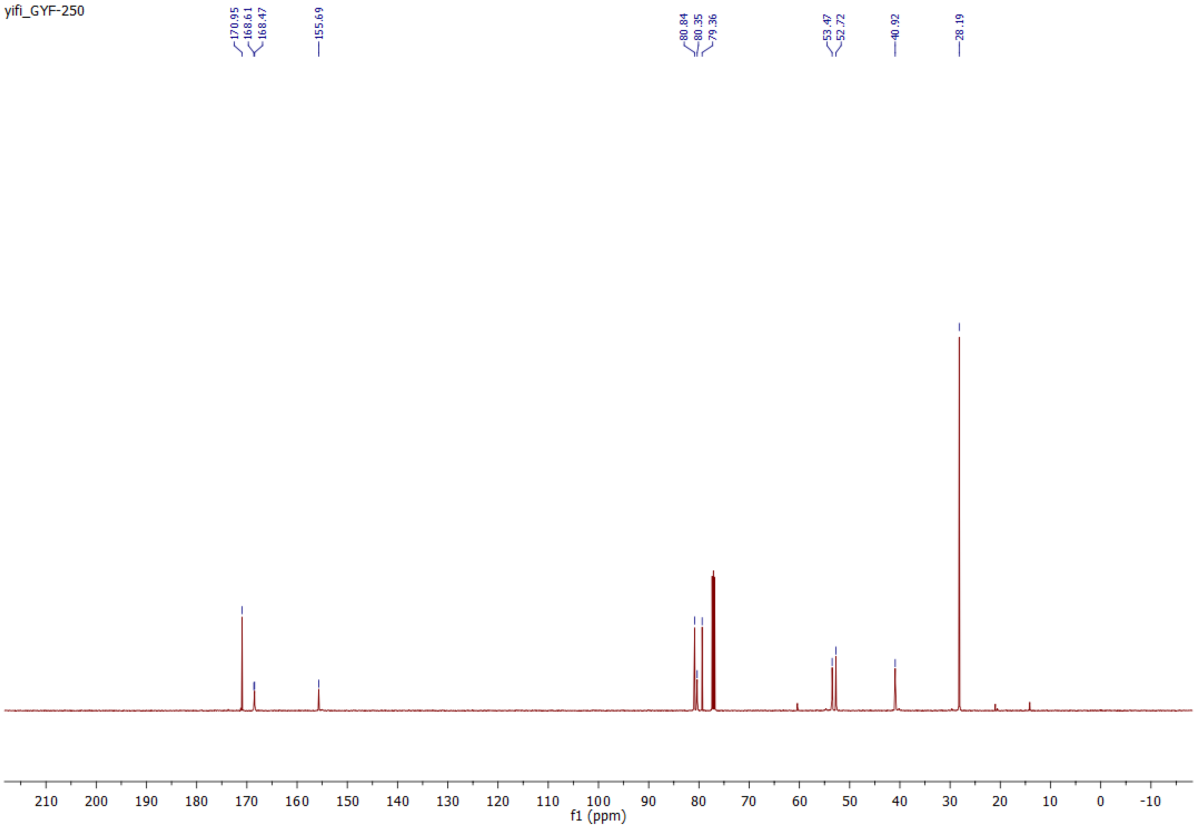

Supplementary Figure 46. ^{1}H- and ^{13}C- NMR Spectra for compound **9**.

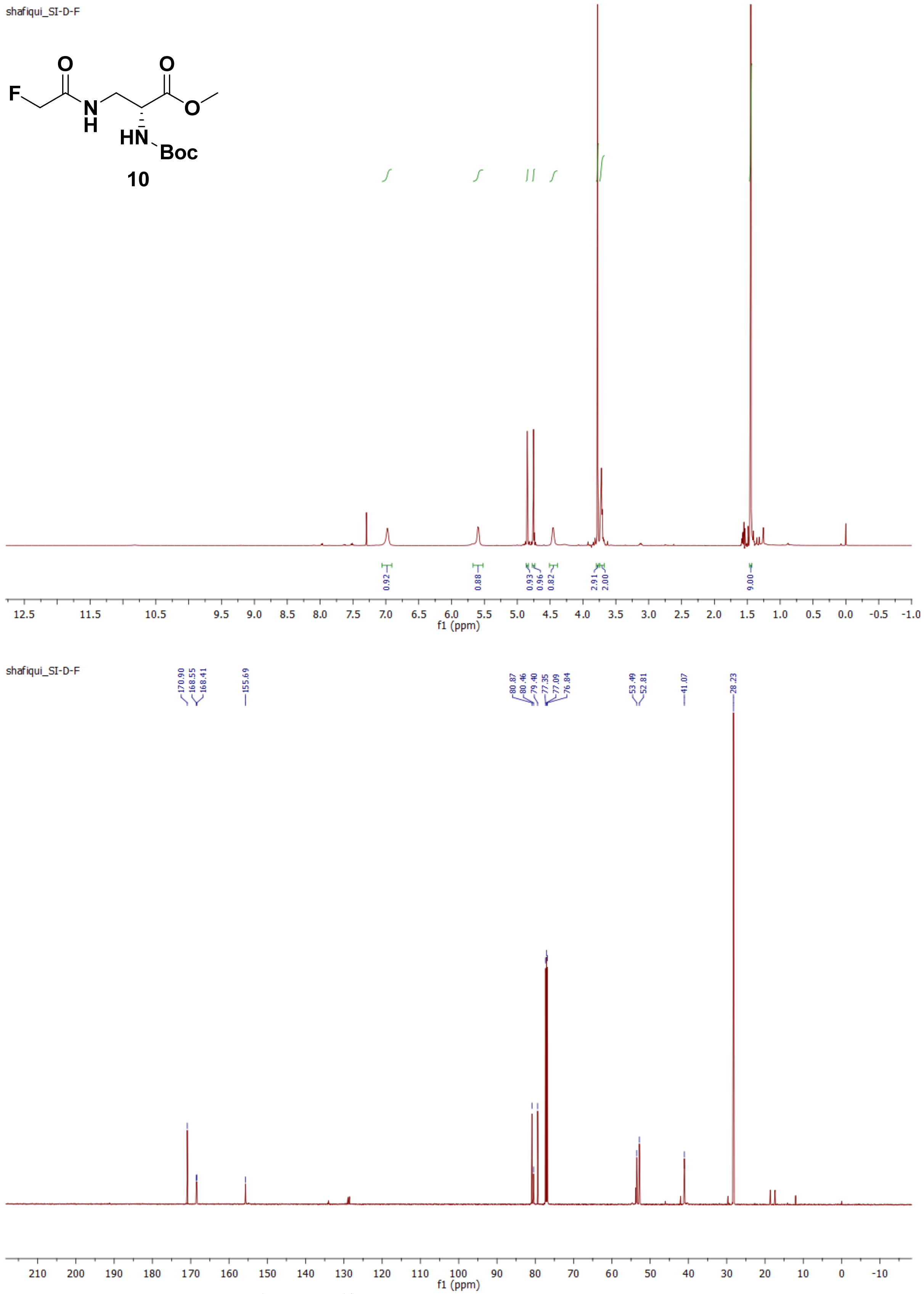

Supplementary Figure 47. ^{1}H- and ^{13}C- NMR Spectra for compound **10.**

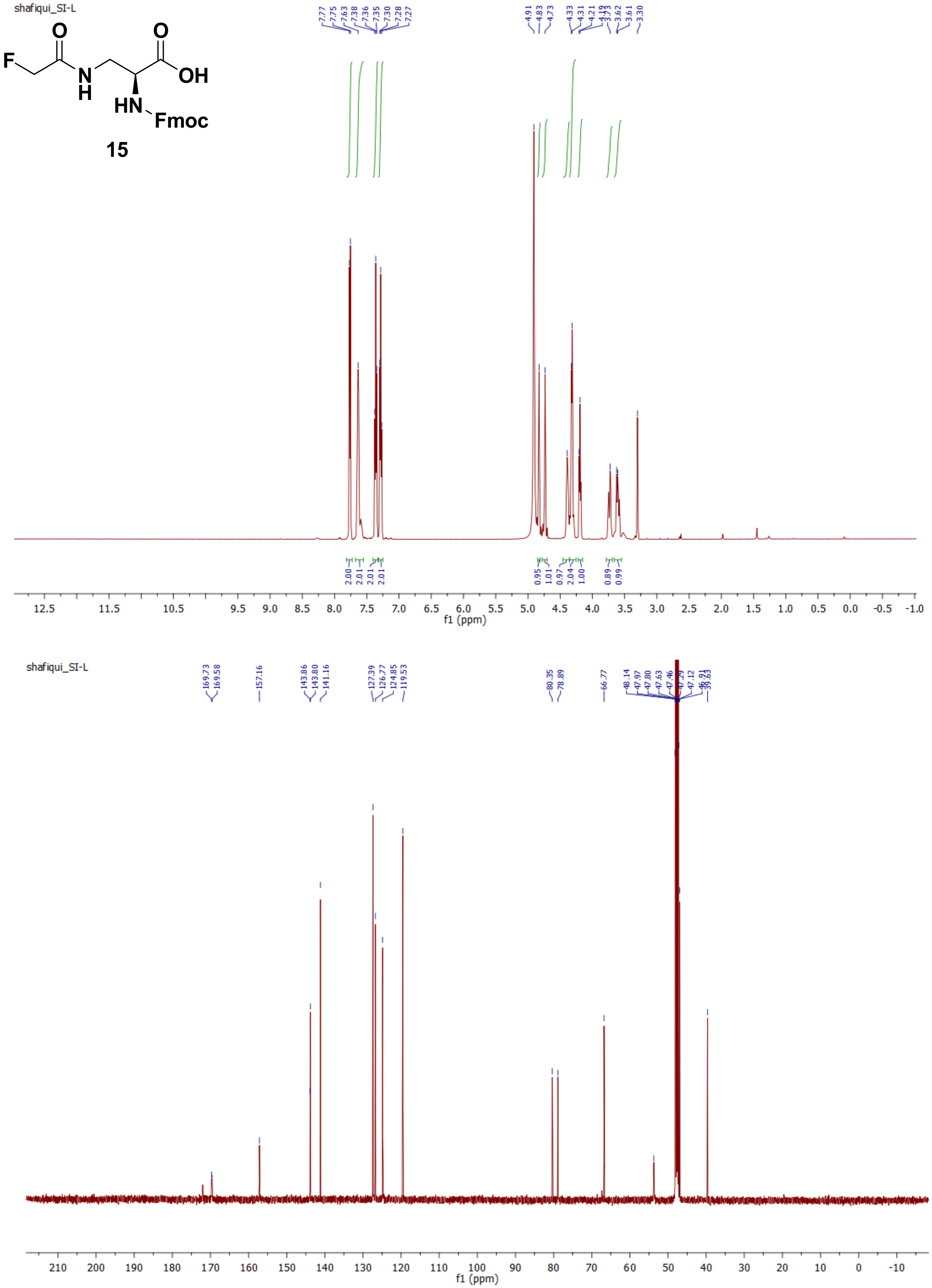

Supplementary Figure 48. ^{1}H- and ^{13}C- NMR Spectra for compound **15.**

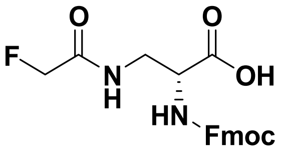

16

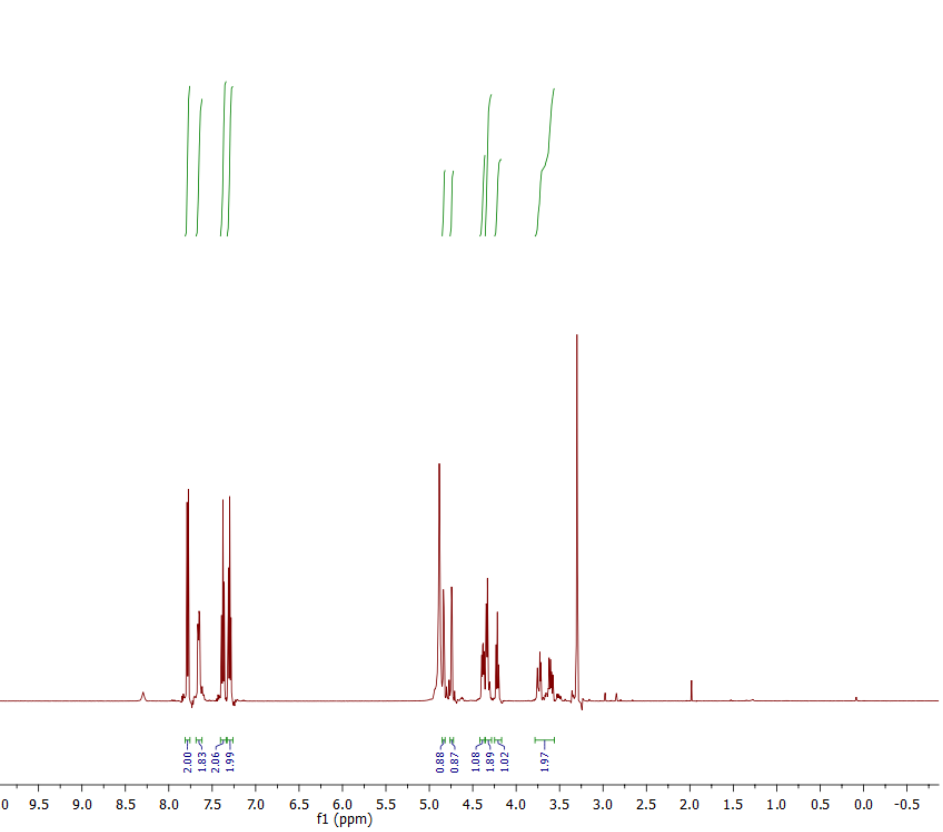

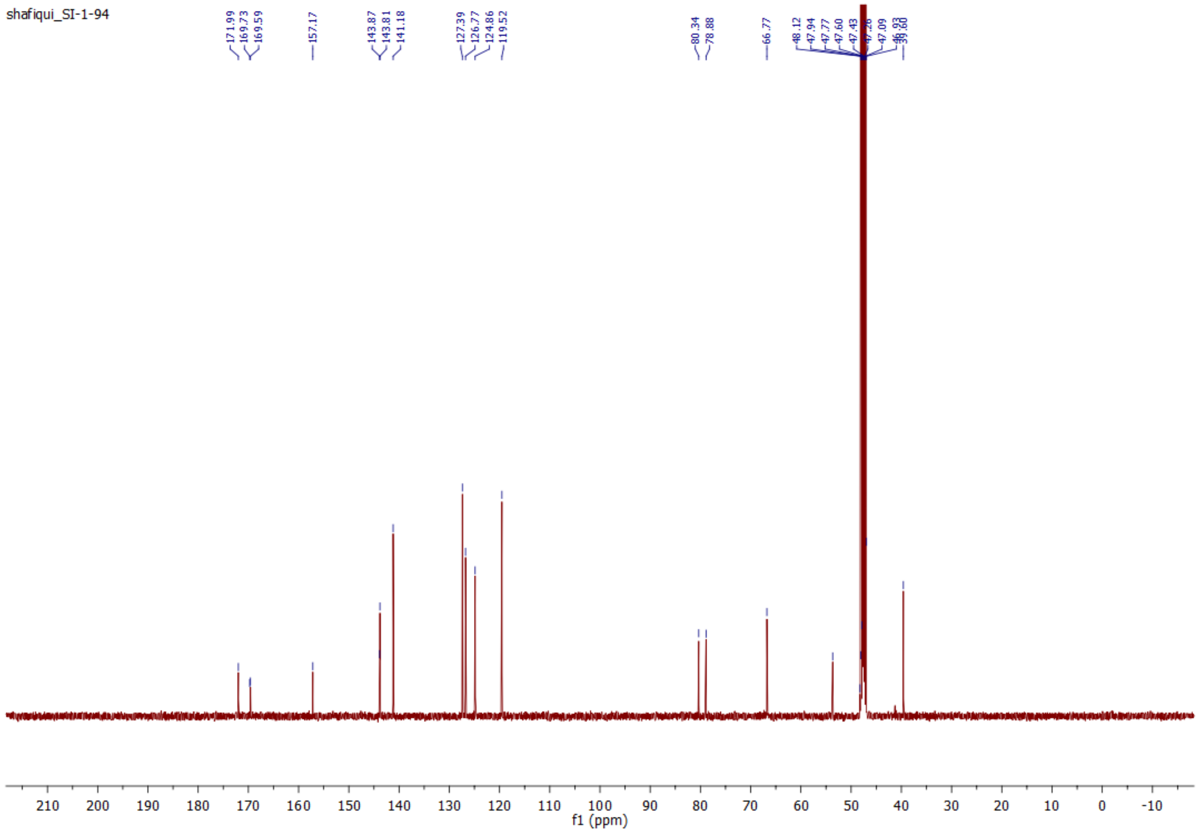

Supplementary Figure 49. ^{1}H- and ^{13}C- NMR Spectra for compound **16**.

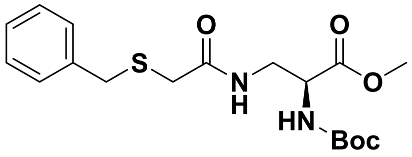

17

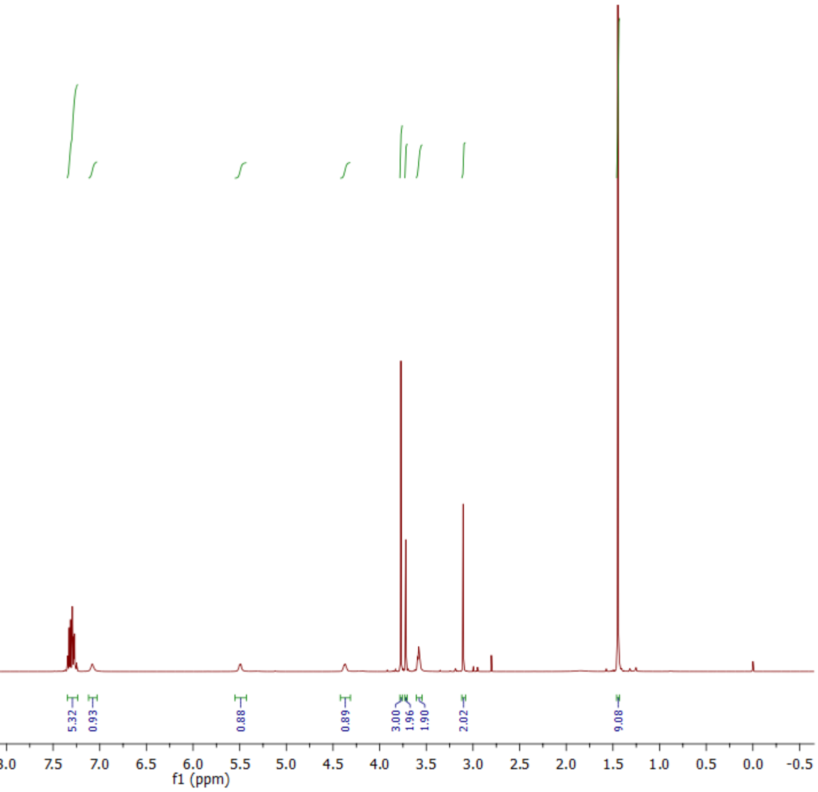

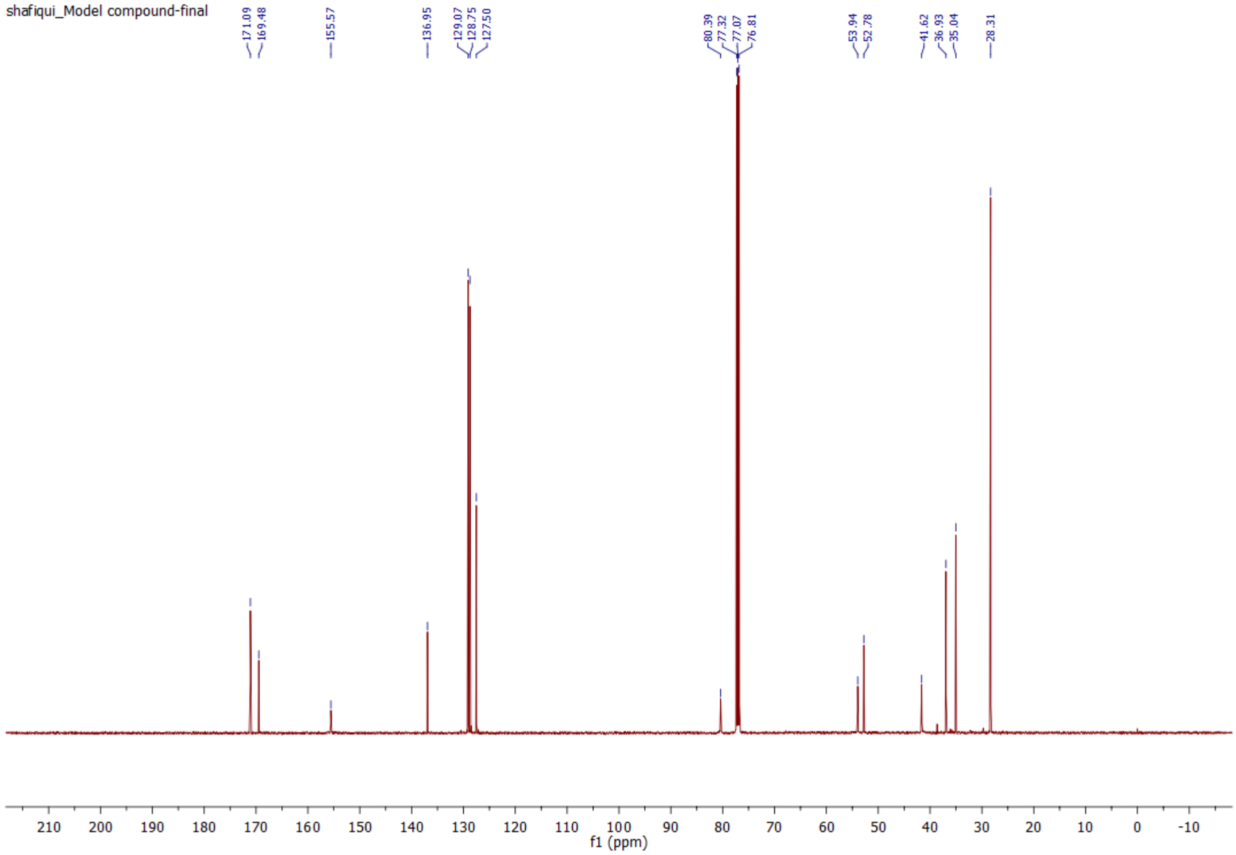

Supplementary Figure 50. ^{1}H- and ^{13}C- NMR Spectra for compound **17**.

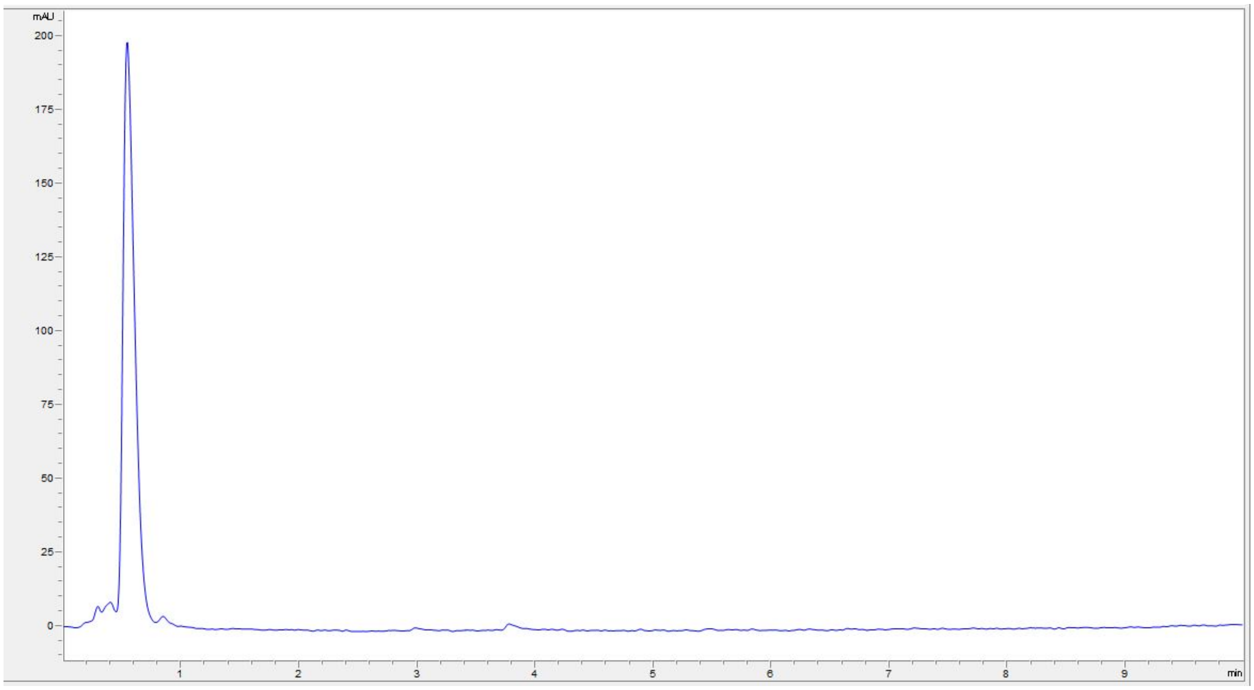

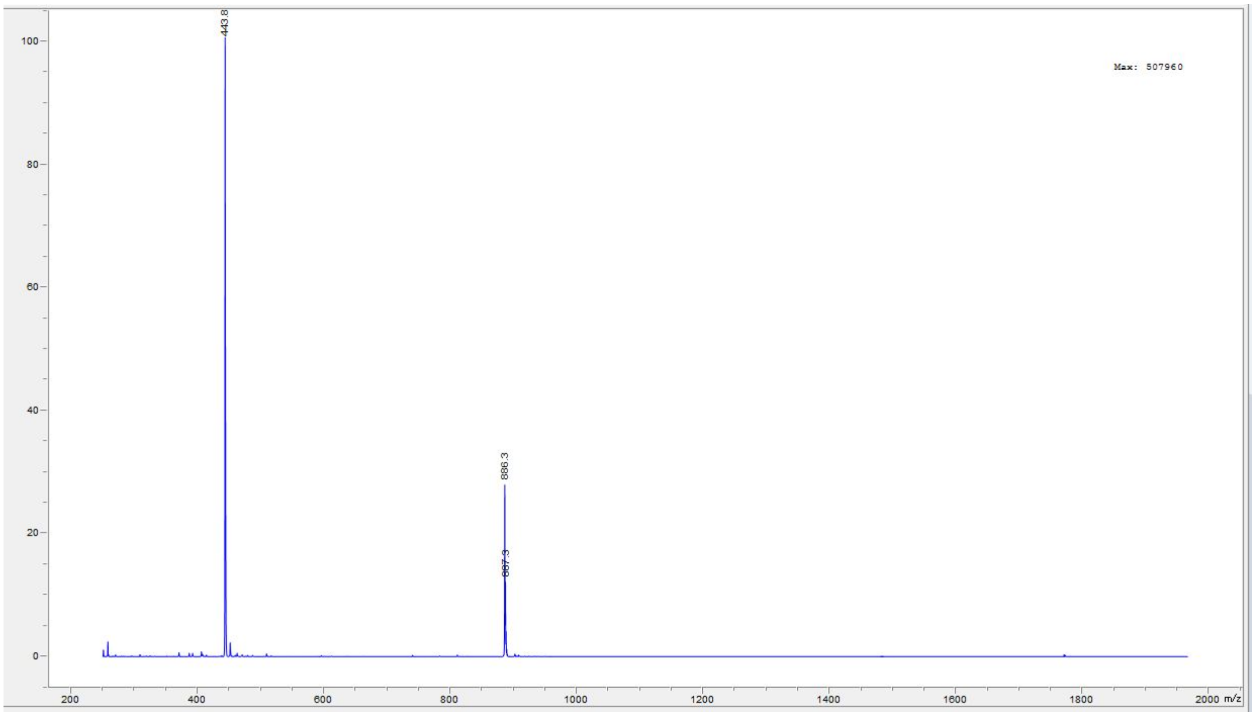

Supplementary Figure 51. LC-MS Spectra for peptide **20.**

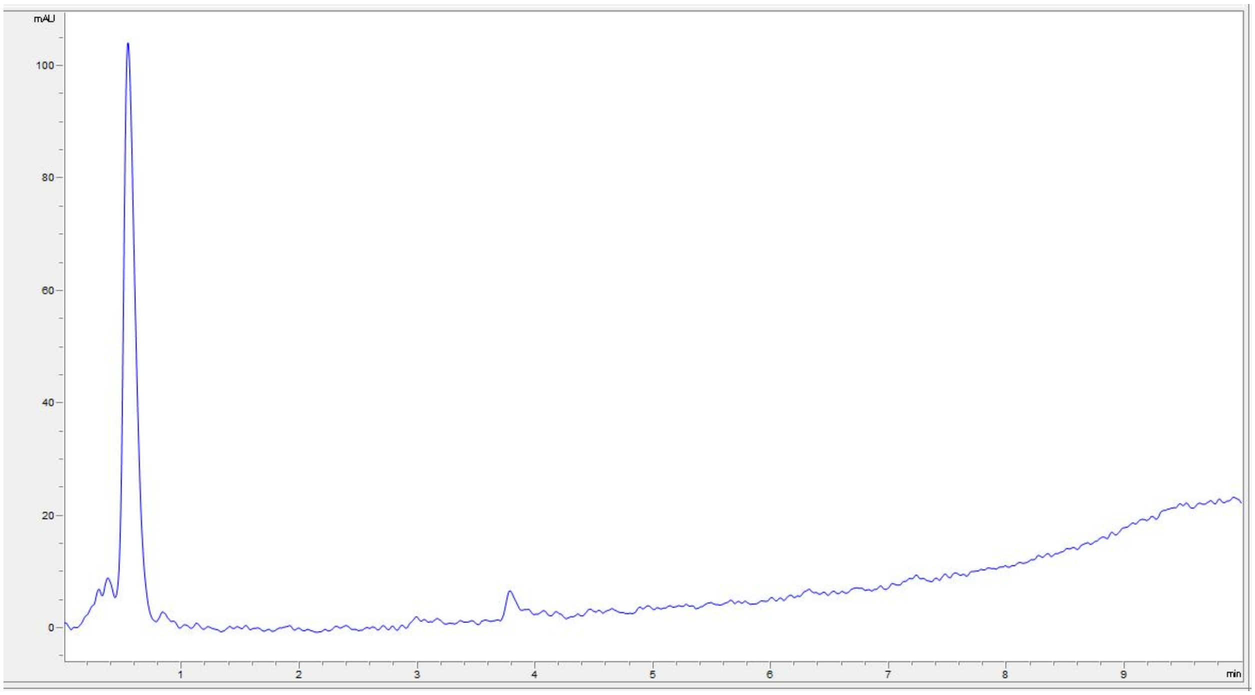

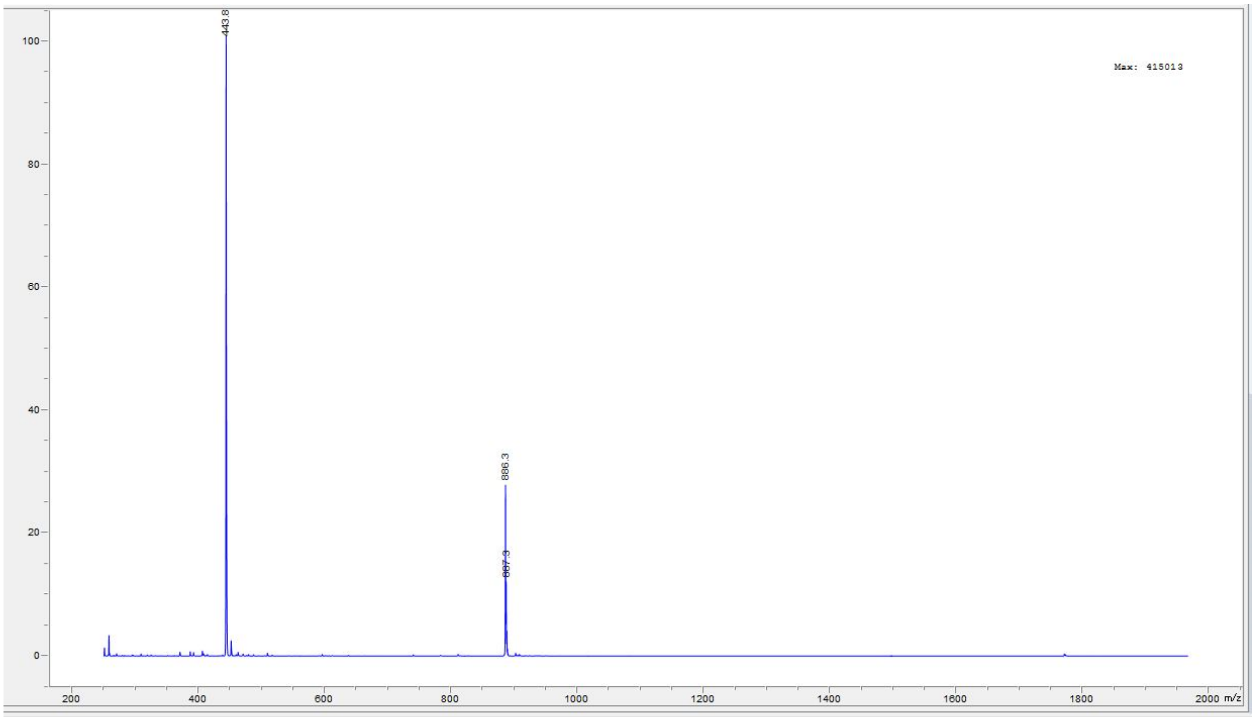

Supplementary Figure 52. LC-MS Spectra for peptide **21.**

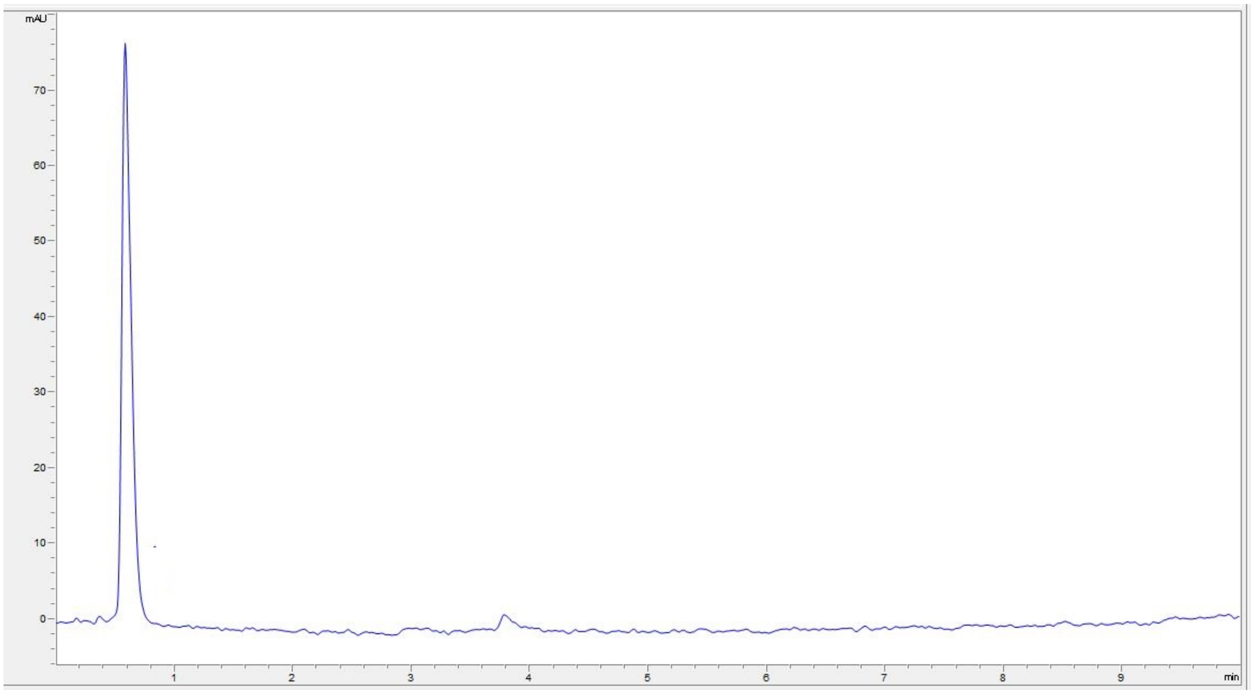

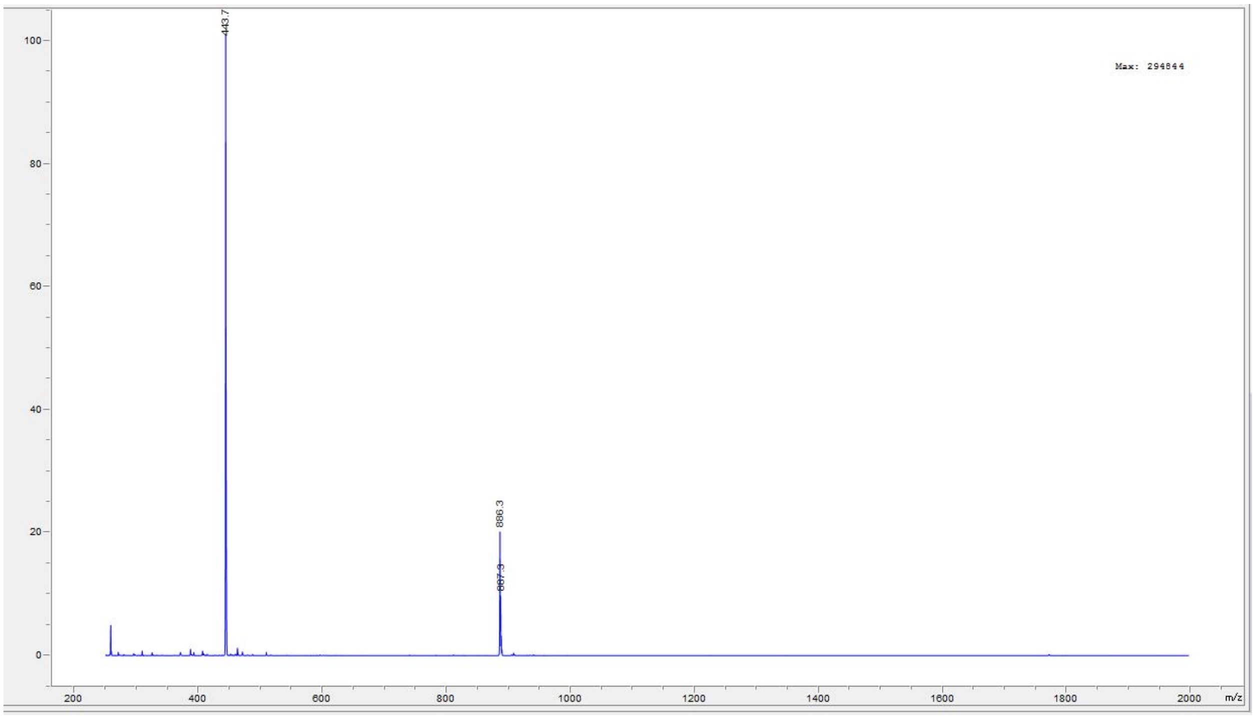

Supplementary Figure 53. LC-MS Spectra for peptide **22.**

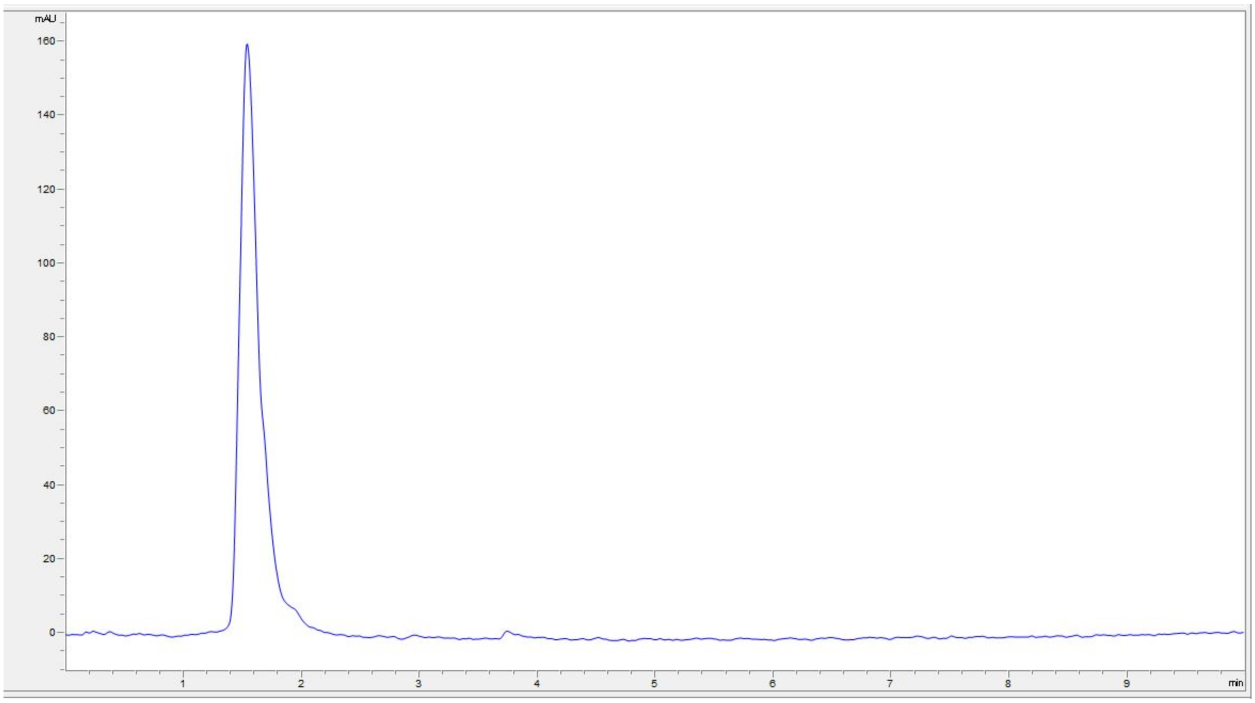

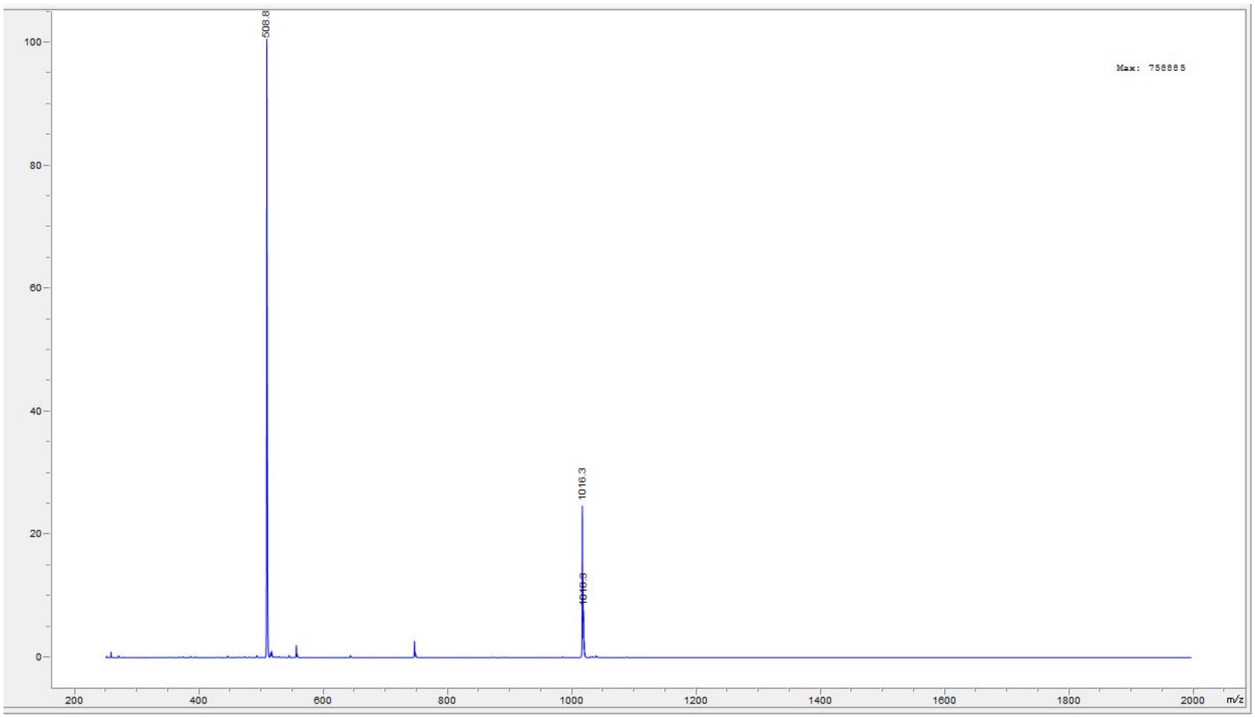

Supplementary Figure 54. LC-MS Spectra for peptide **23.**

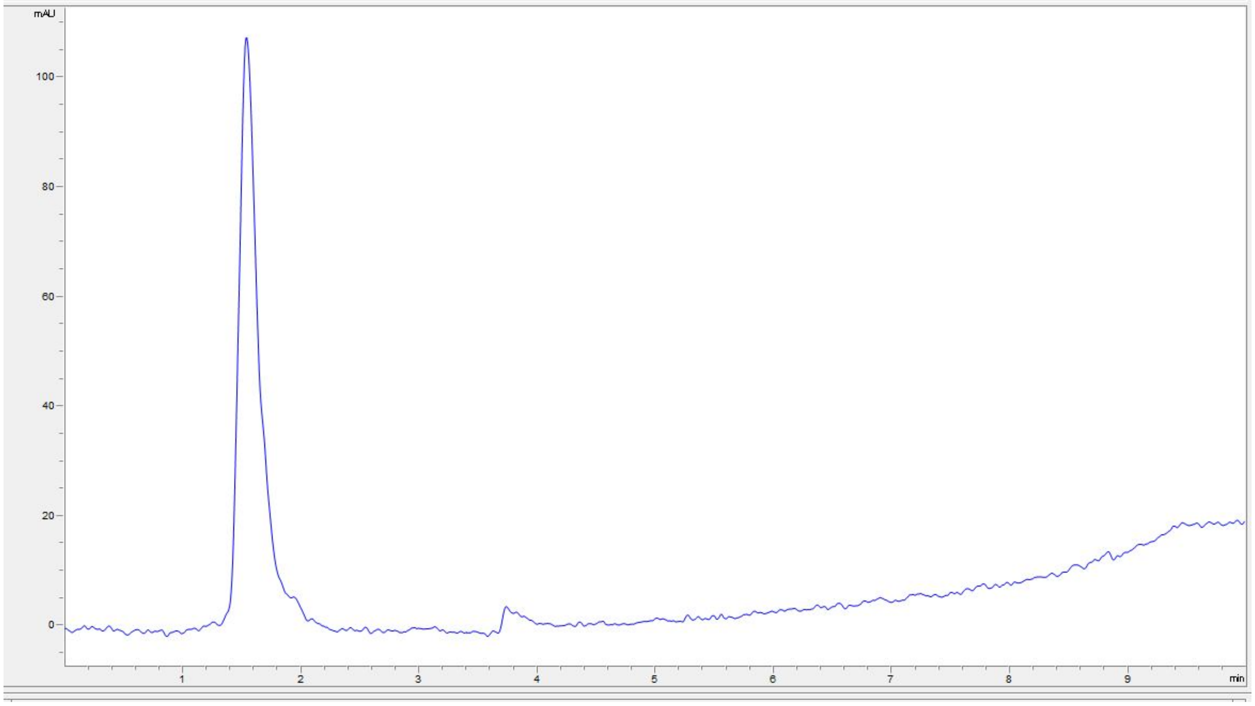

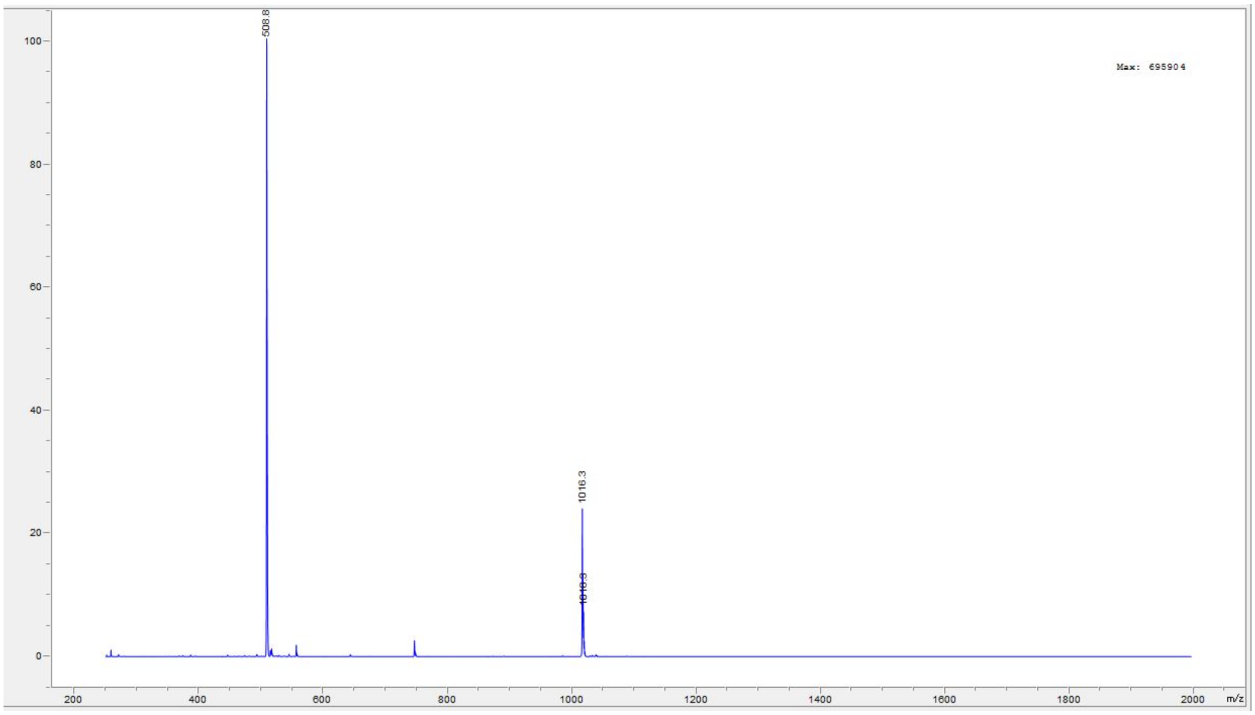

Supplementary Figure 55. LC-MS Spectra for peptide **24.**

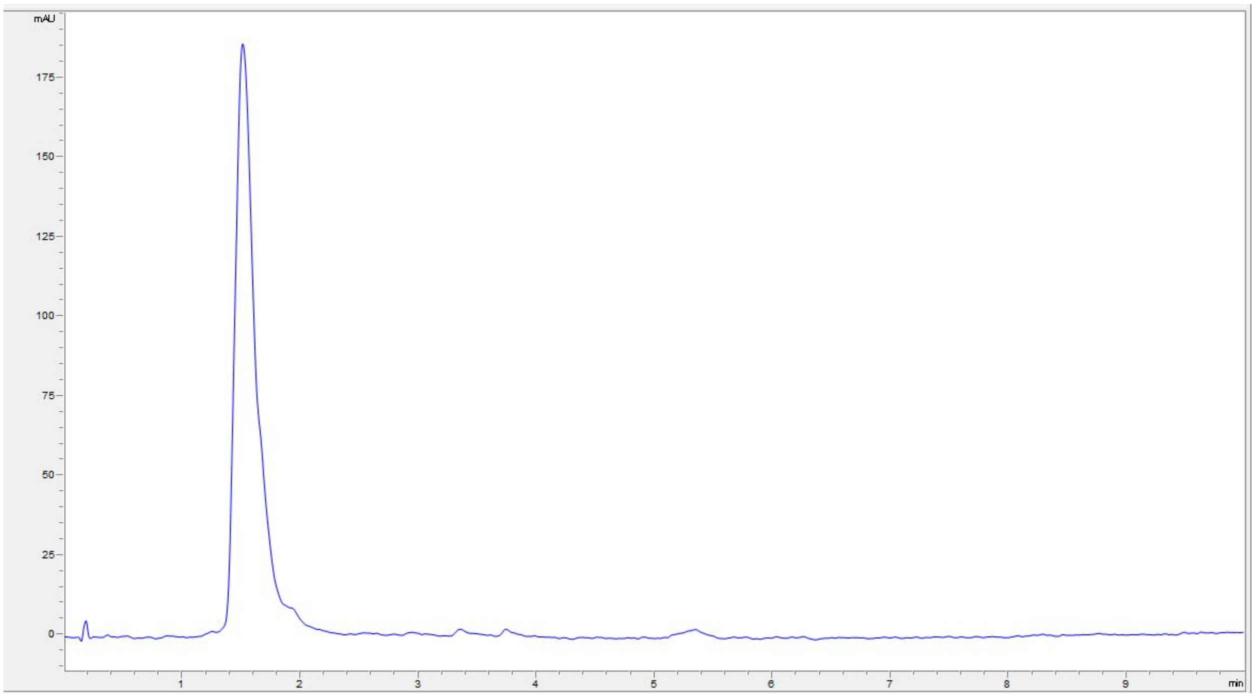

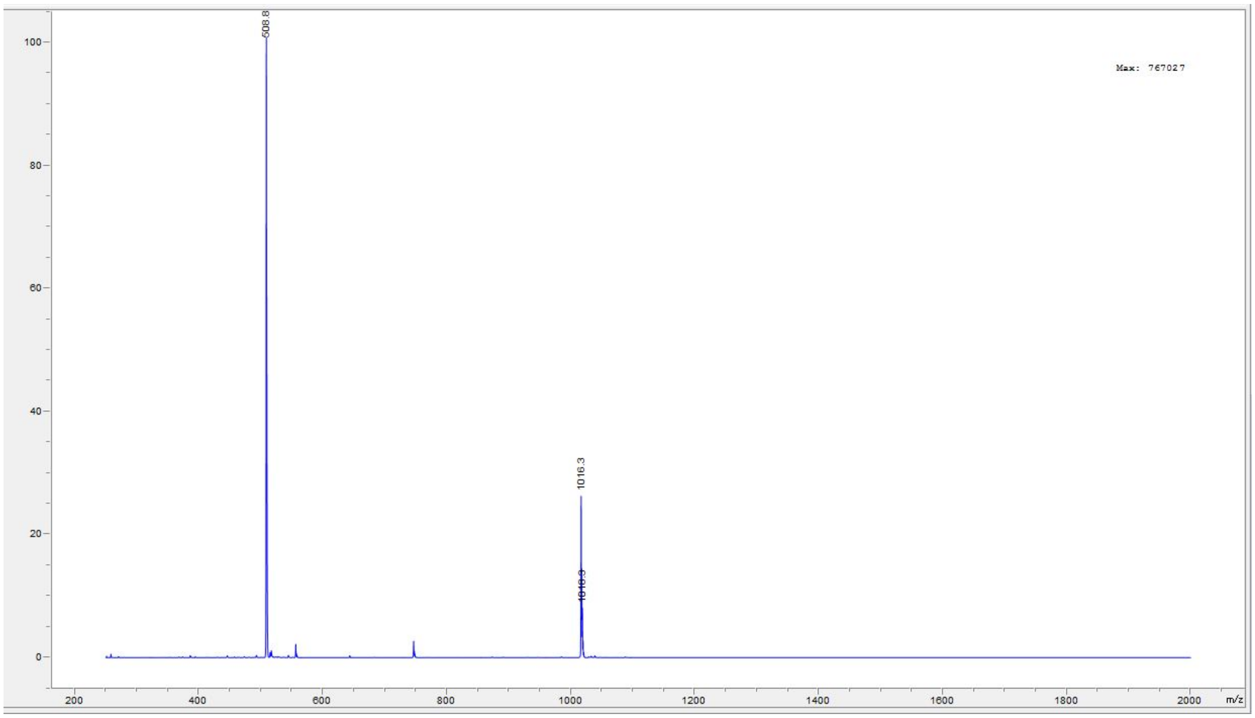

Supplementary Figure 56. LC-MS Spectra for peptide **25.**

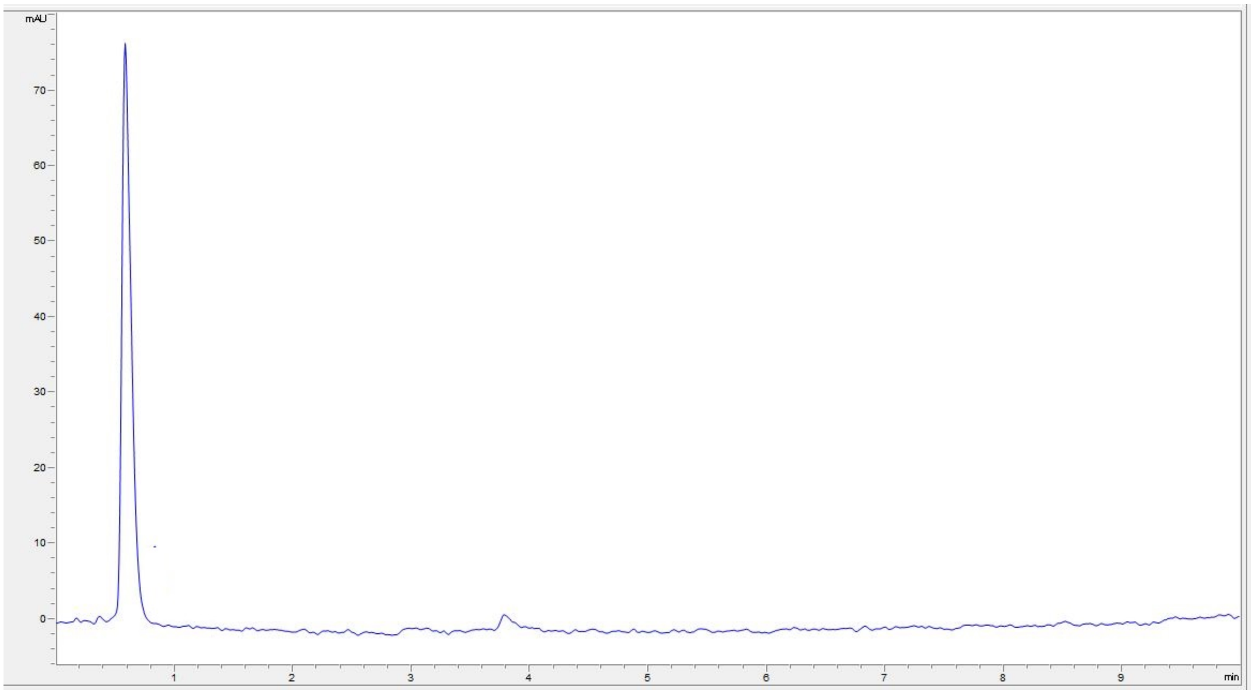

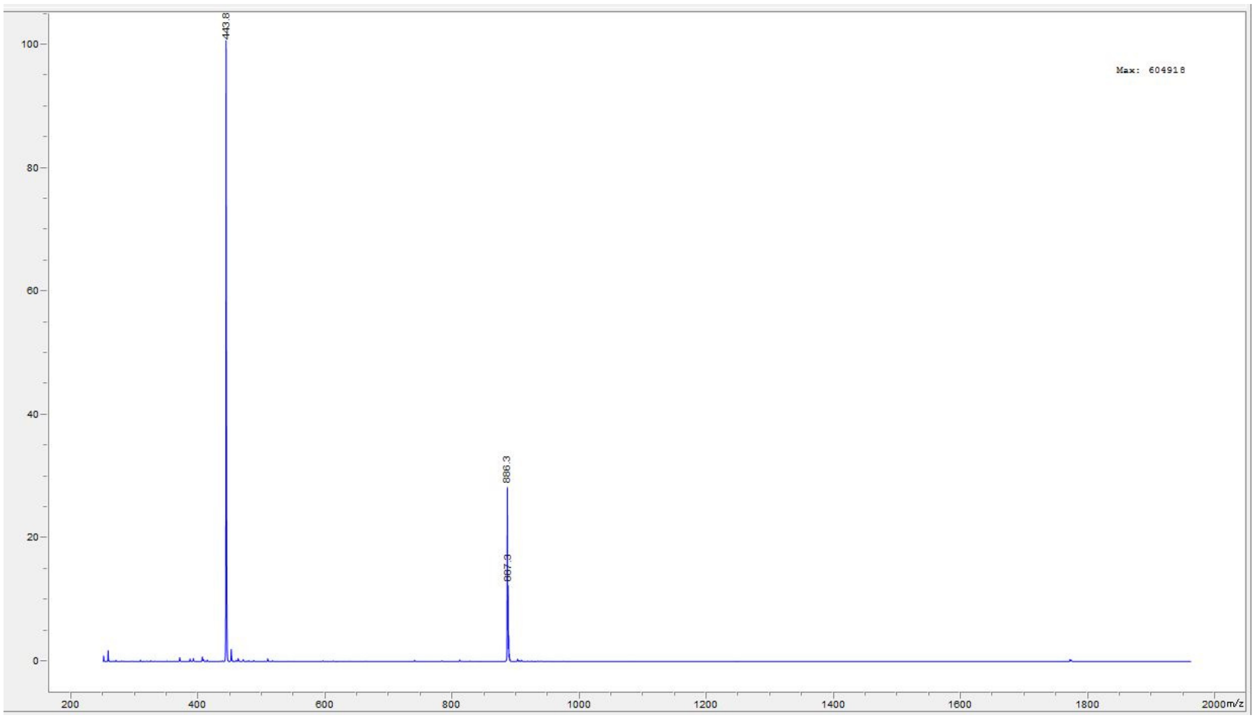

Supplementary Figure 57. LC-MS Spectra for peptide **26.**

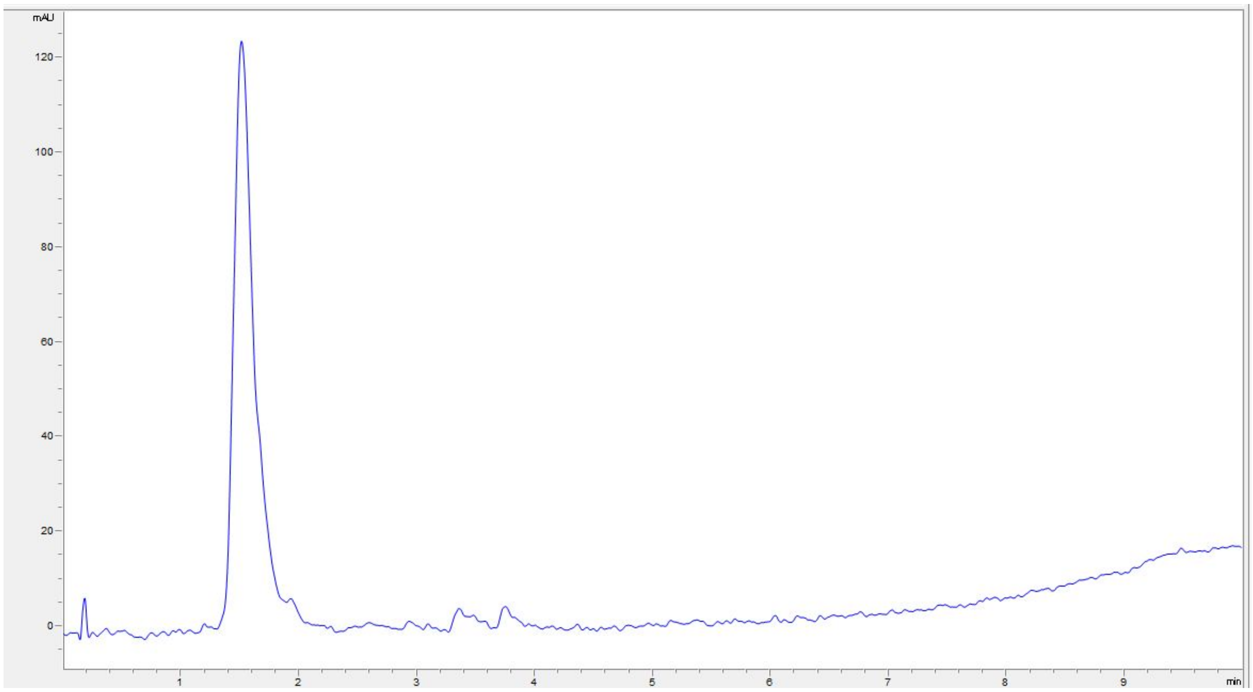

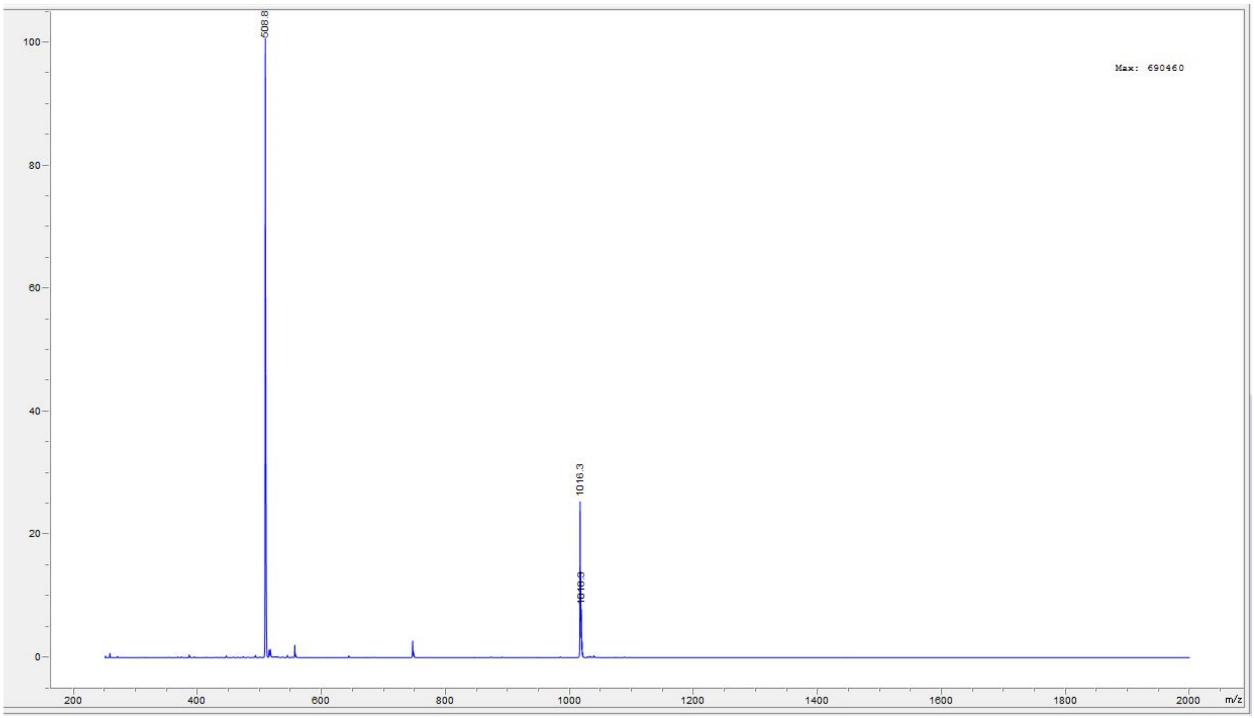

Supplementary Figure 58. LC-MS Spectra for peptide **27.**

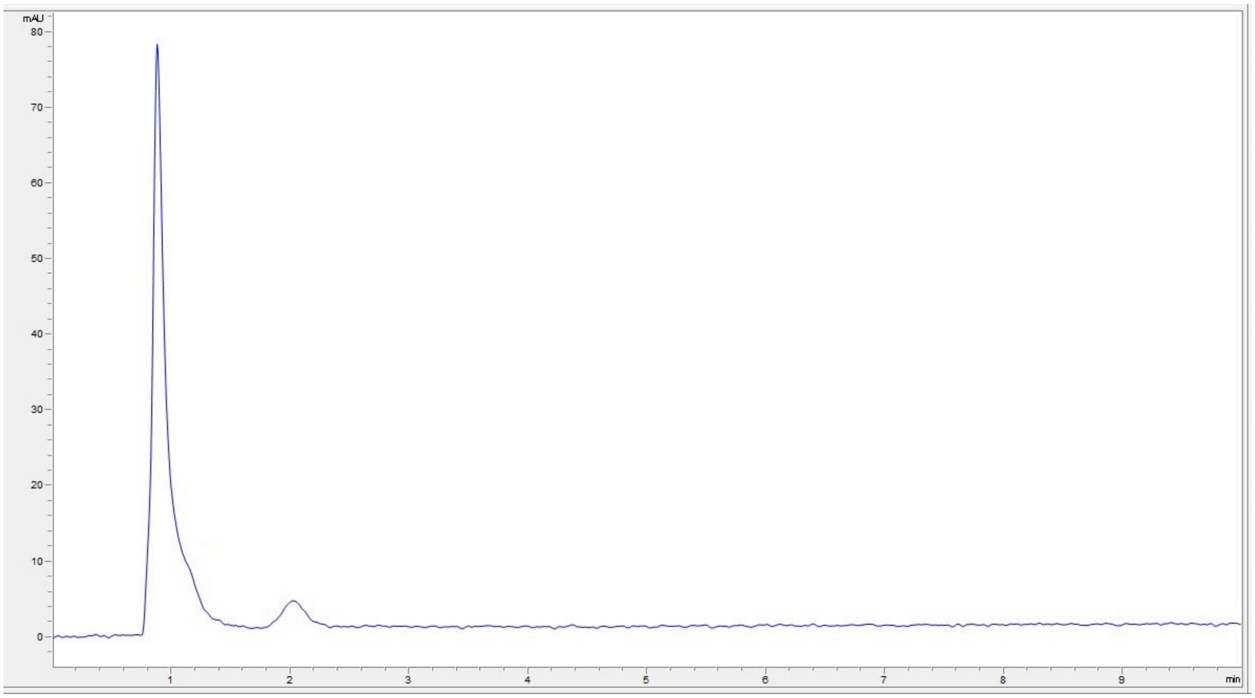

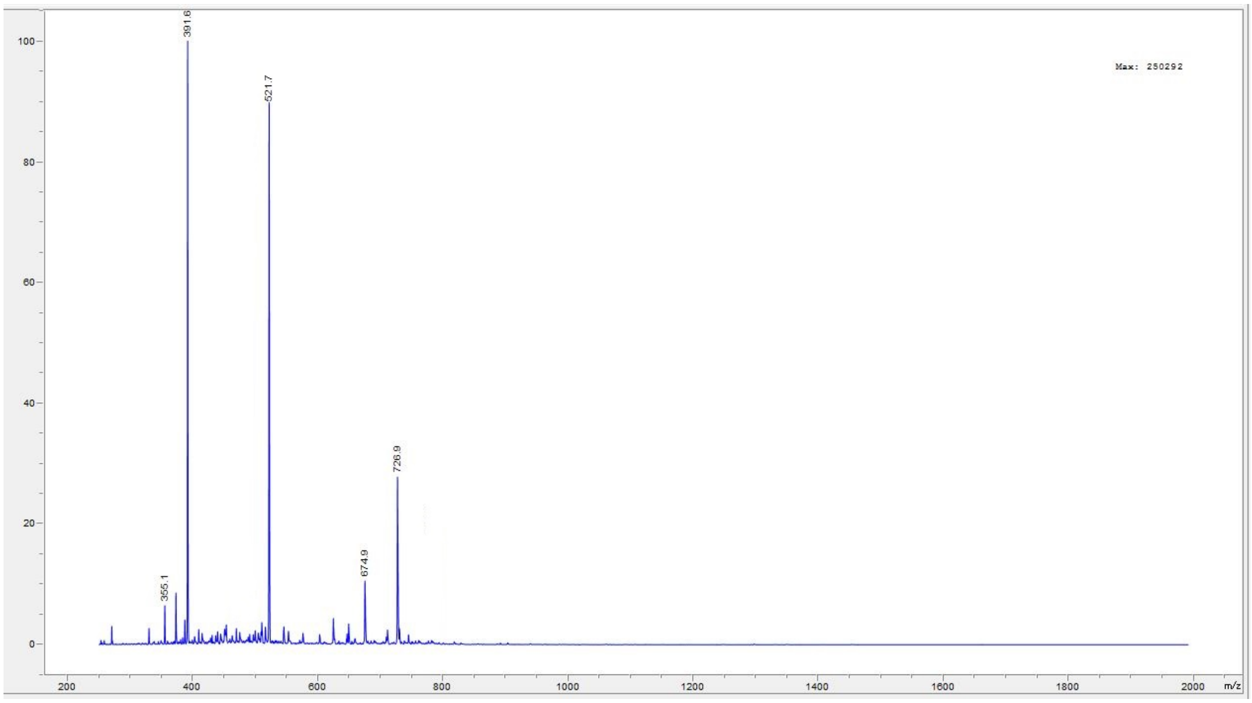

Supplementary Figure 59. LC-MS Spectra for peptide **28.**

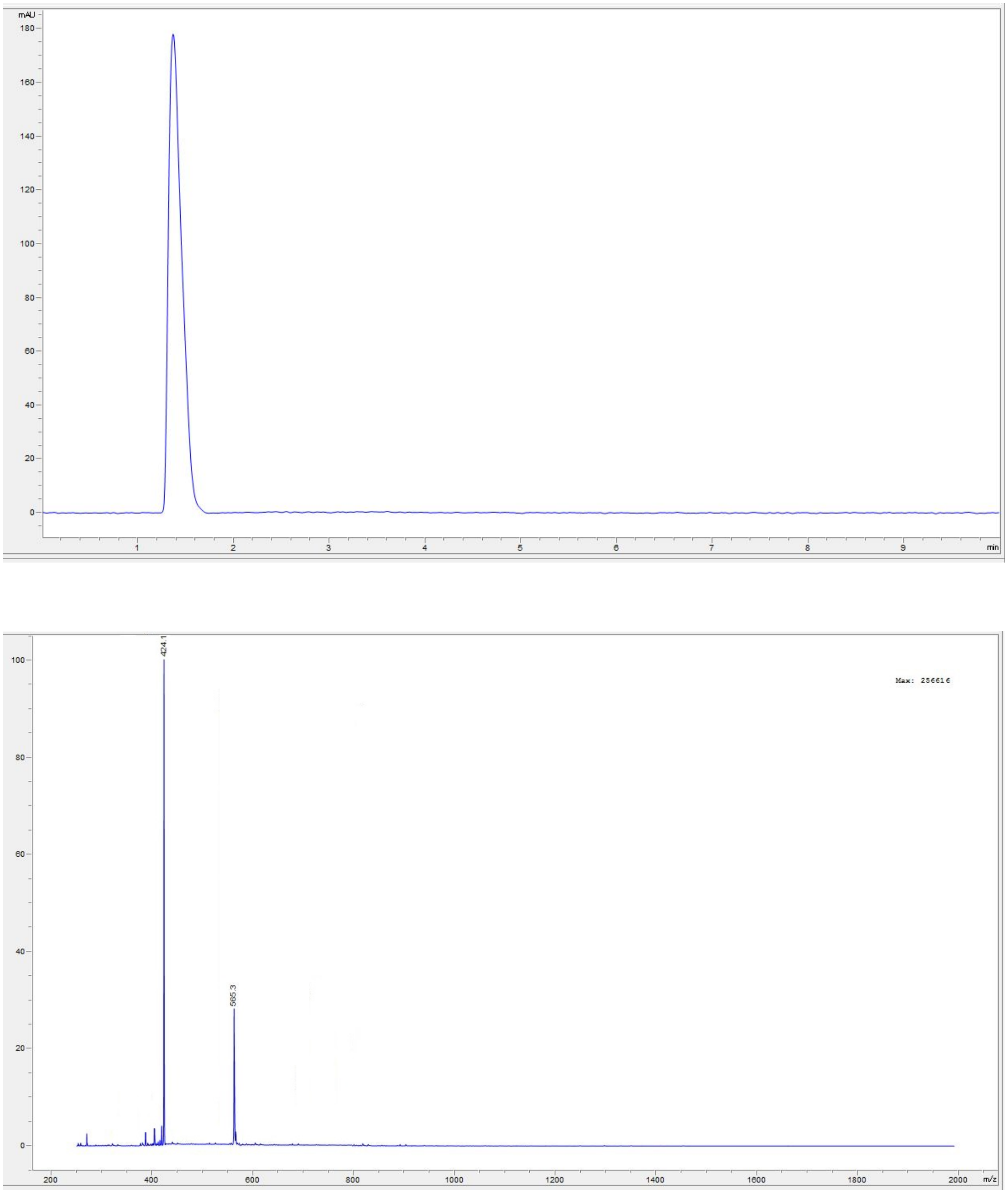

Supplementary Figure 60. LC-MS Spectra for peptide **29.**

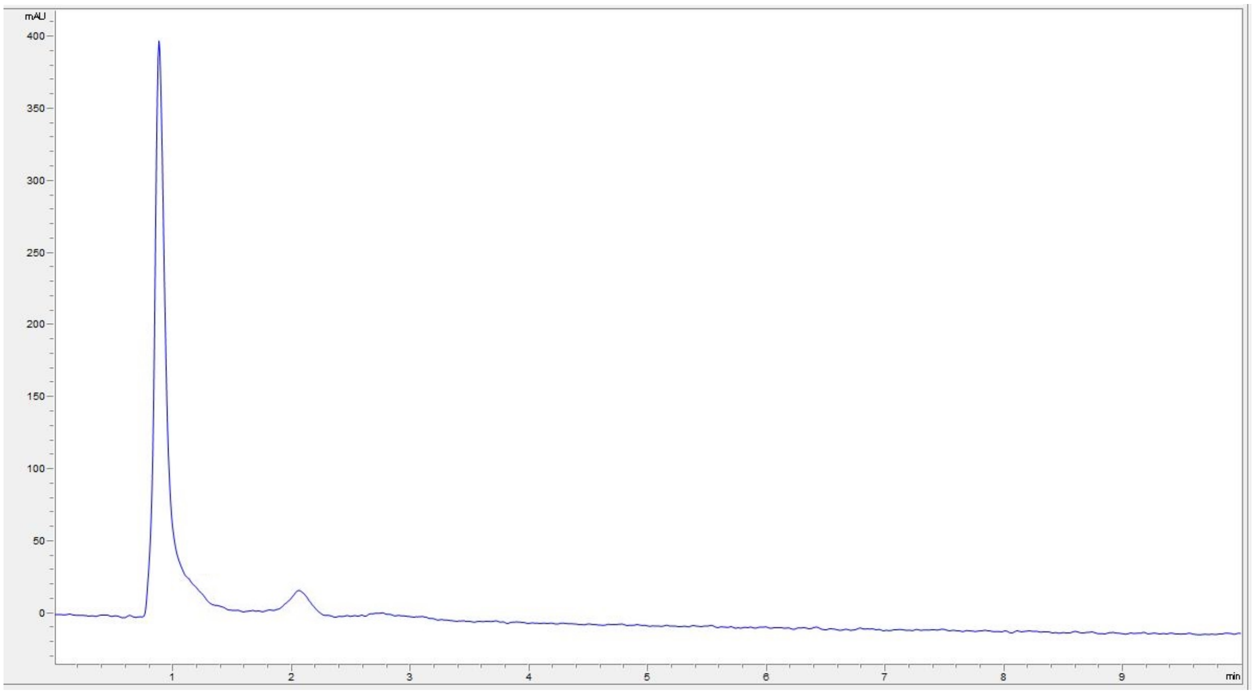

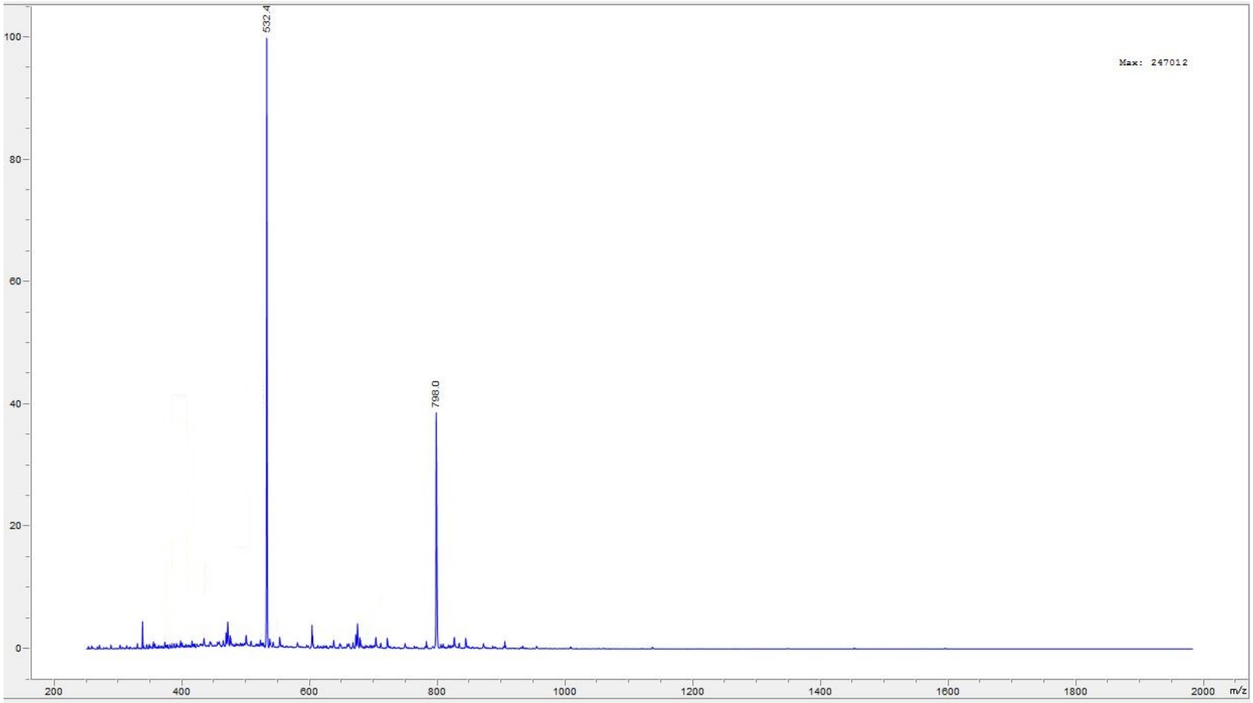

Supplementary Figure 61. LC-MS Spectra for peptide **30.**

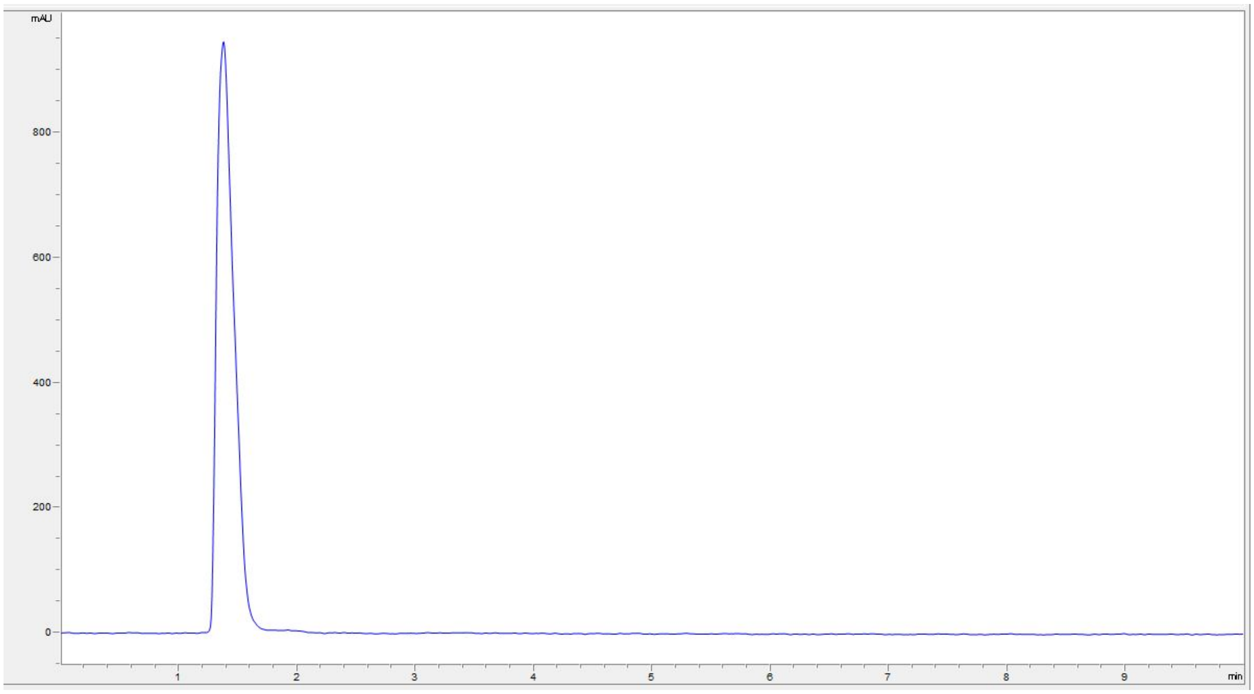

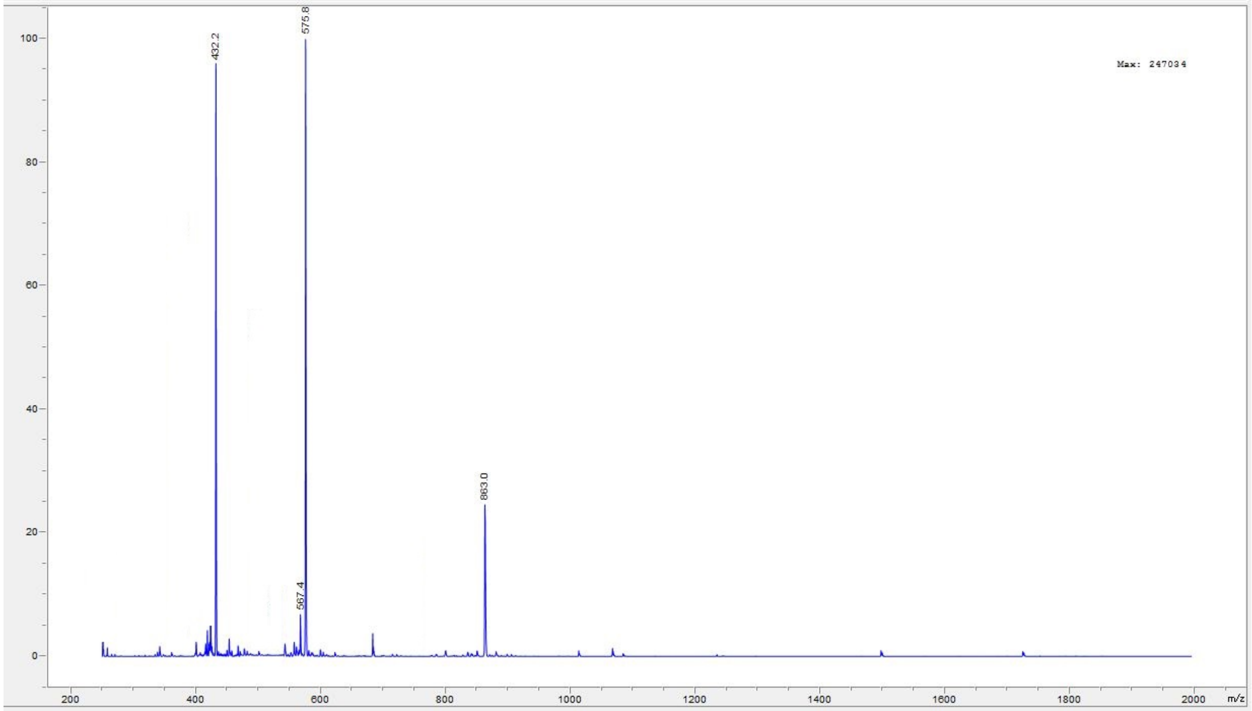

Supplementary Figure 62. LC-MS Spectra for peptide **31.**

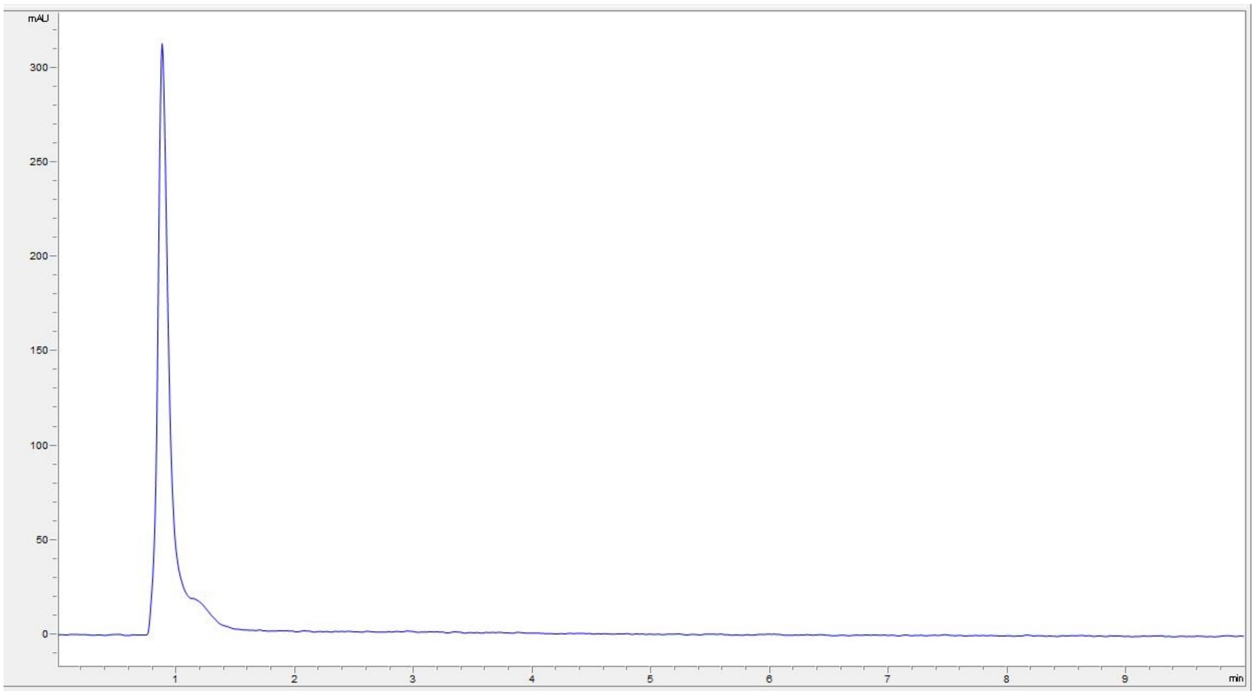

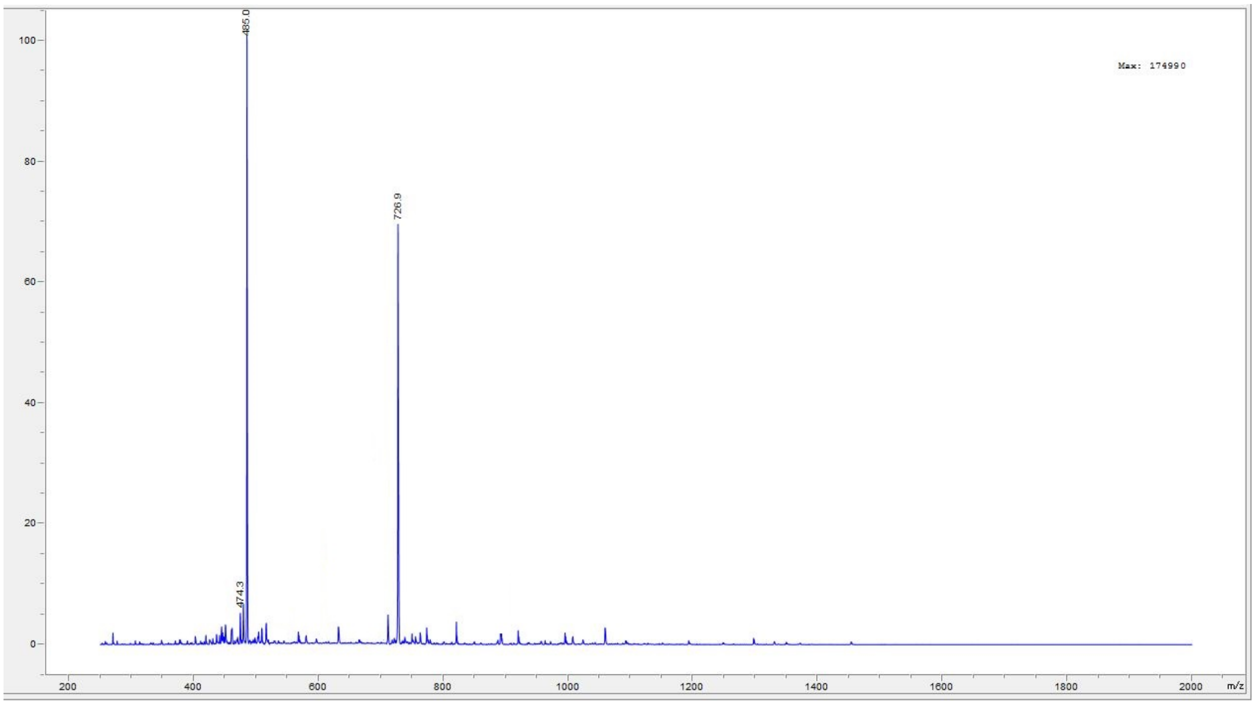

Supplementary Figure 63. LC-MS Spectra for peptide **32.**

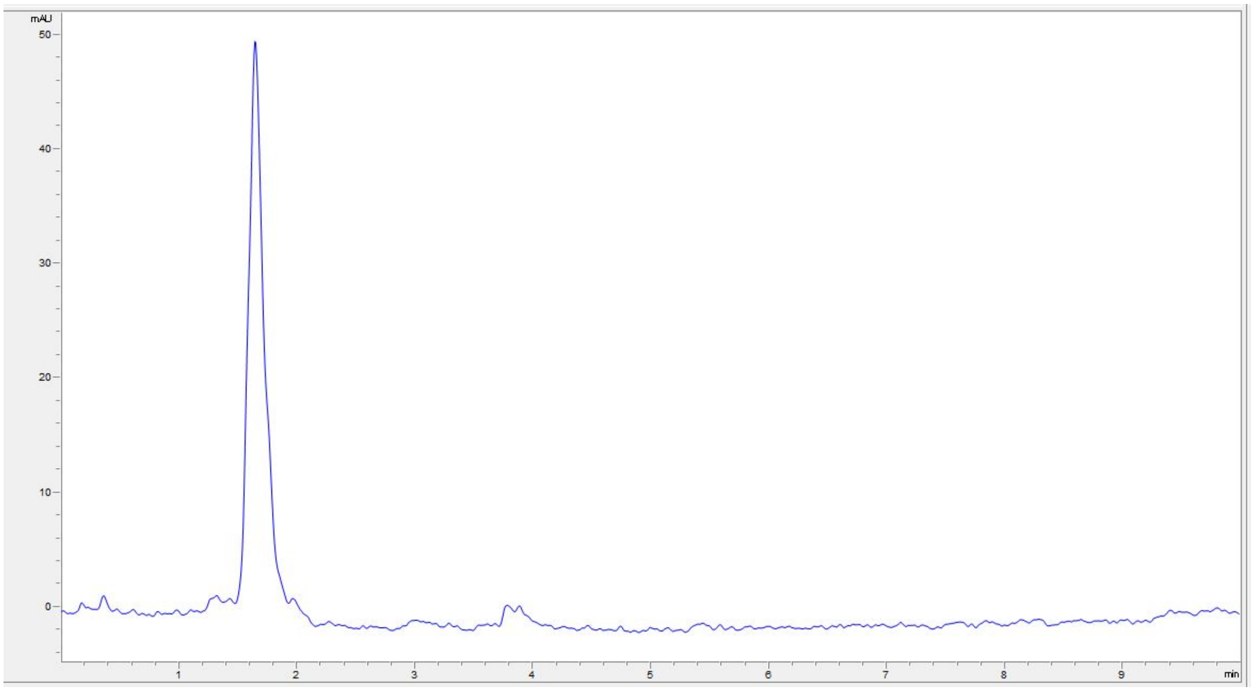

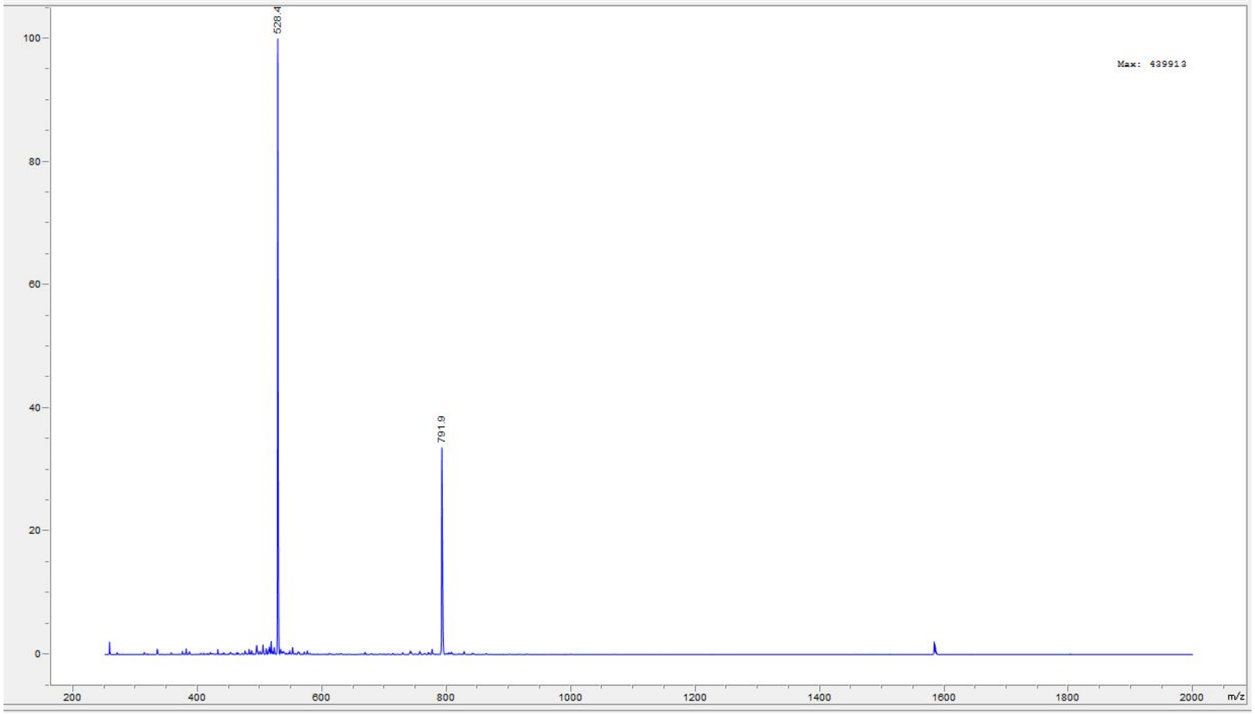

Supplementary Figure 64. LC-MS Spectra for peptide **33.**

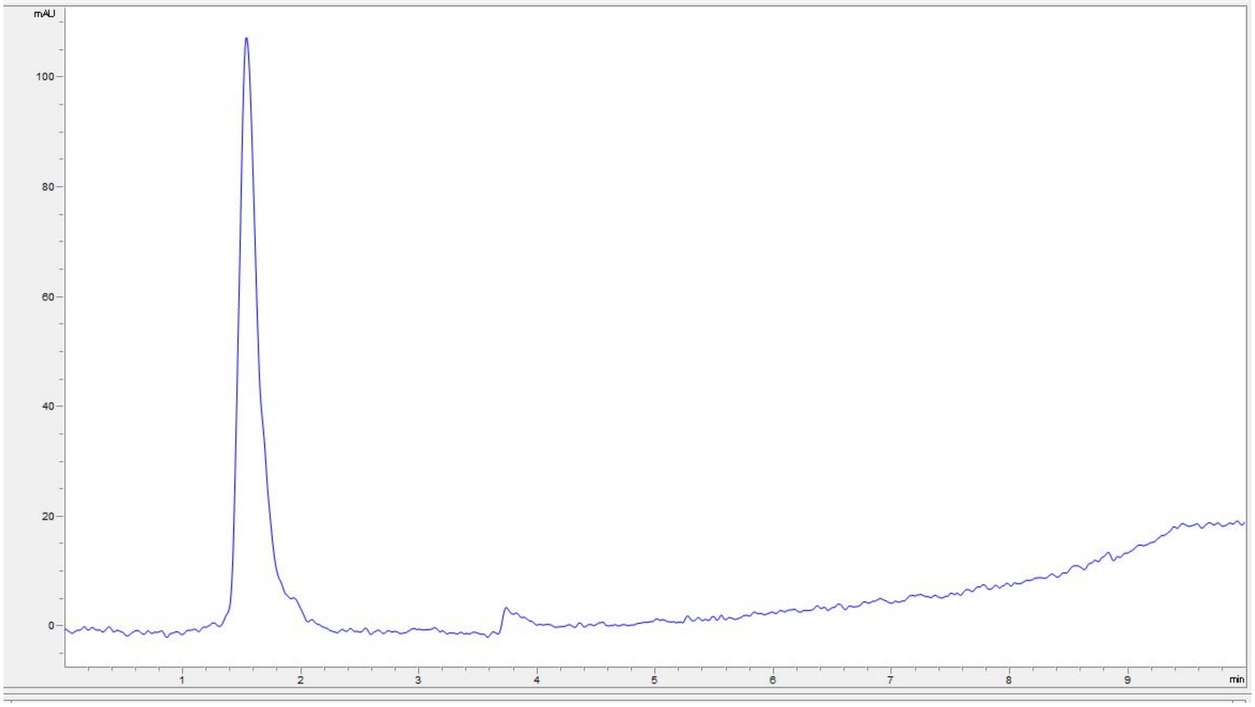

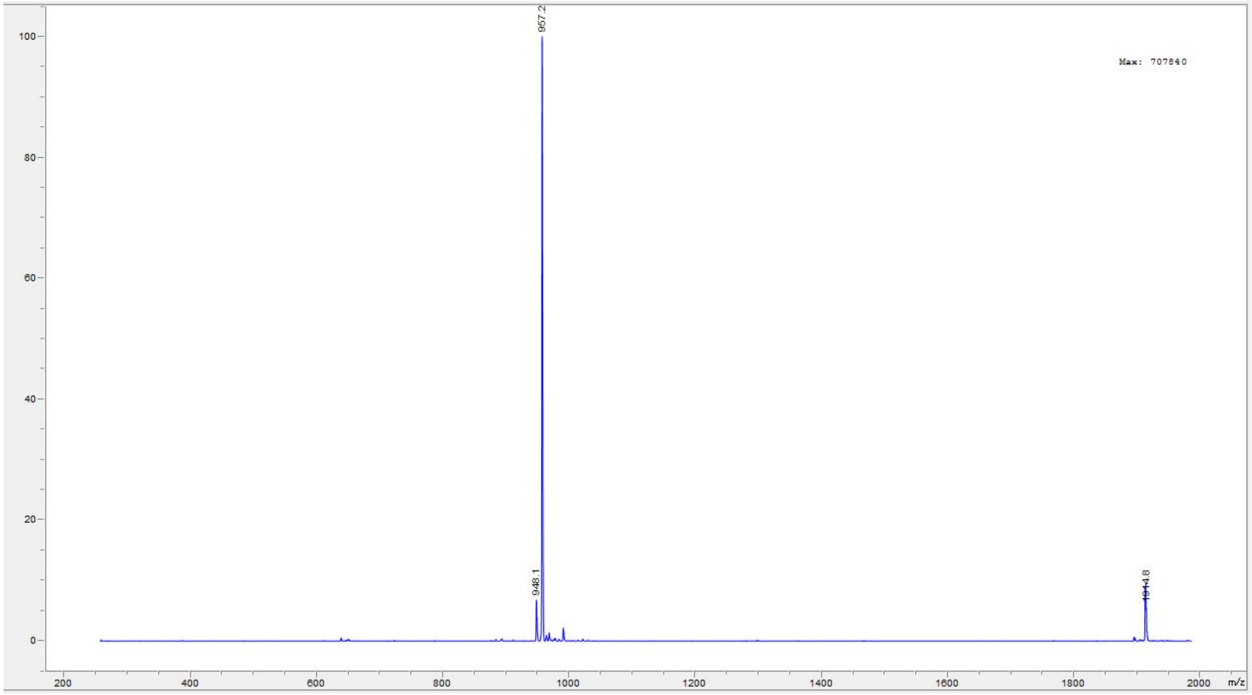

Supplementary Figure 65. LC-MS Spectra for peptide **34.**

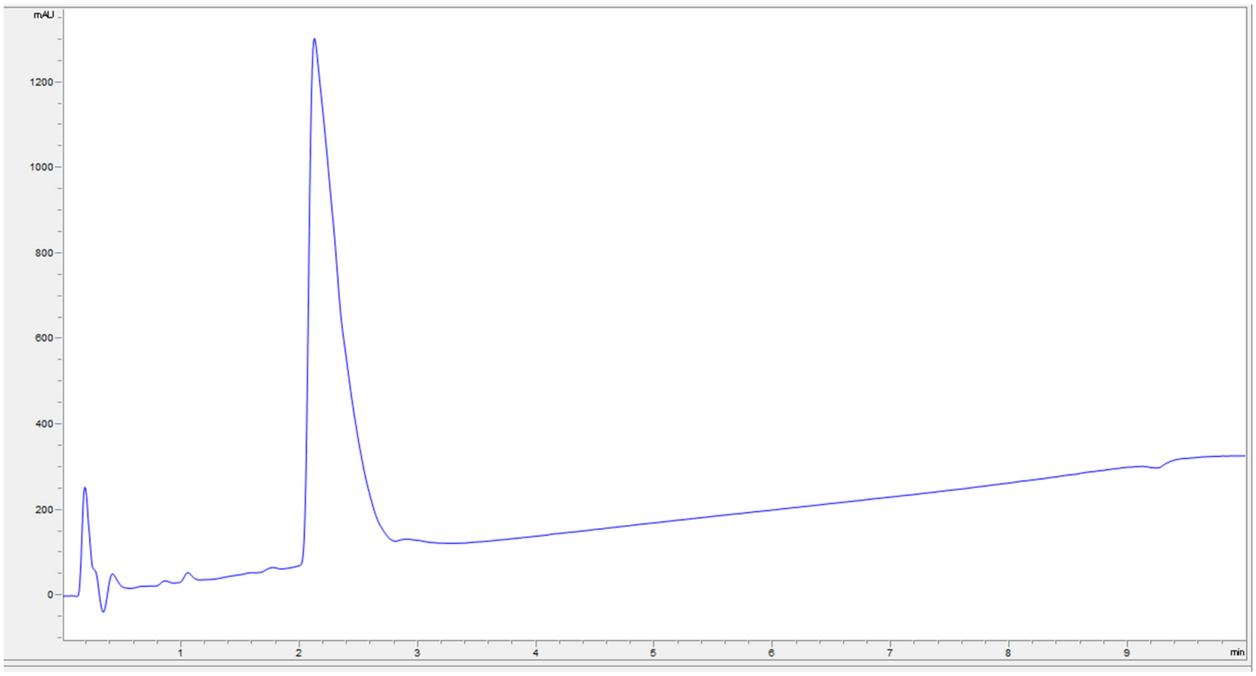

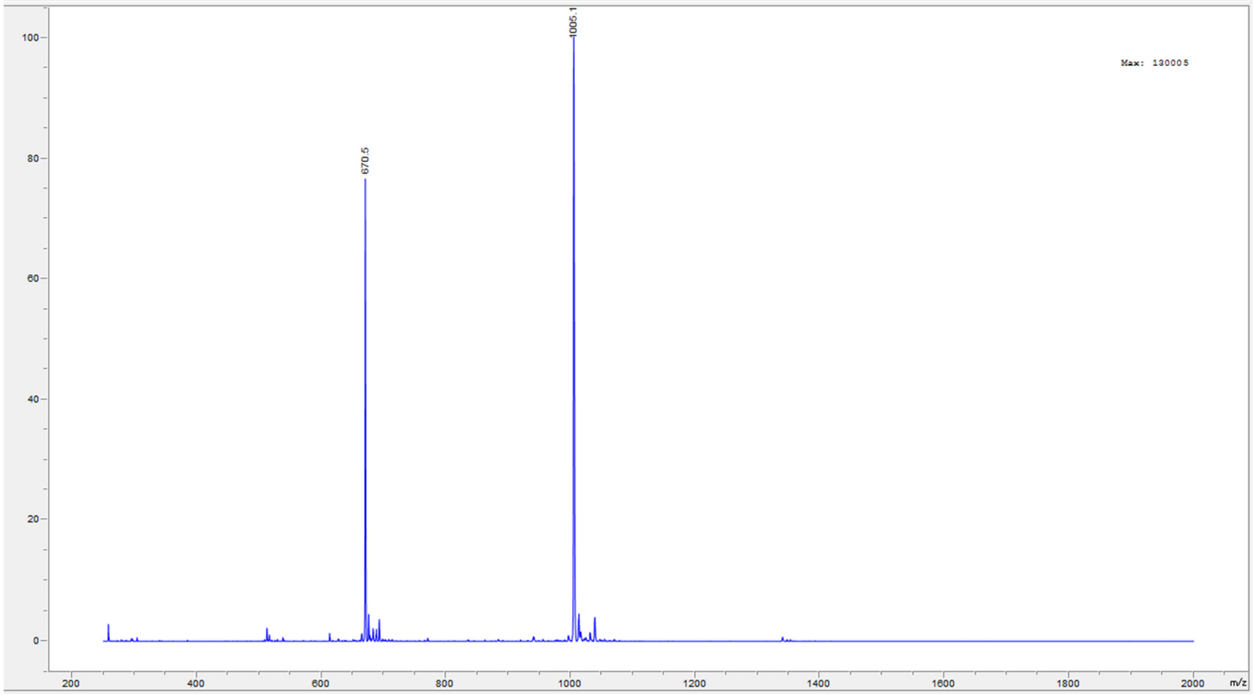

Supplementary Figure 66. LC-MS Spectra for peptide **35.**

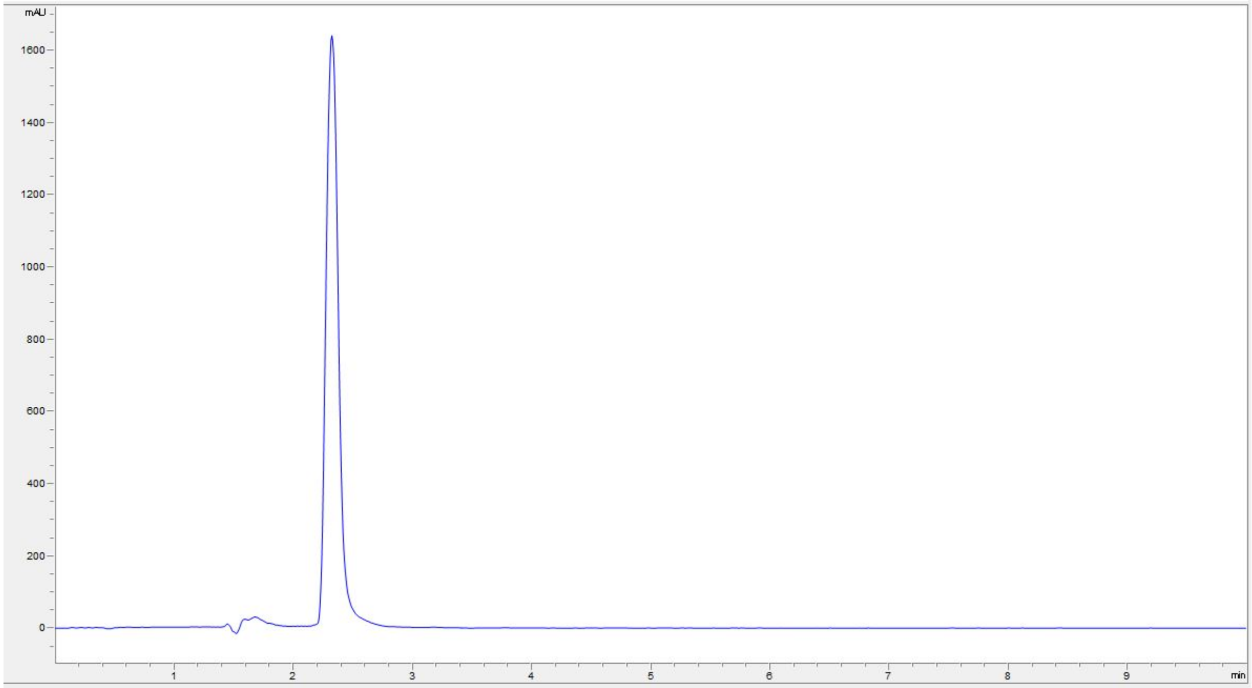

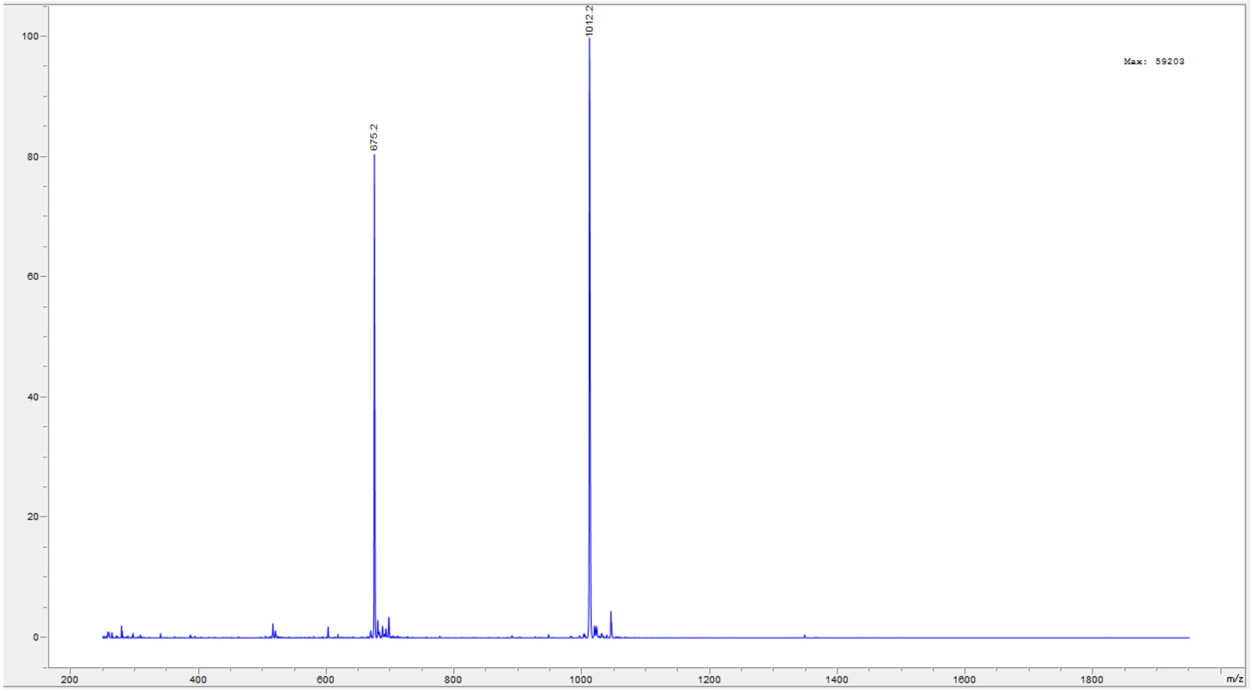

Supplementary Figure 67. LC-MS Spectra for peptide **36.**

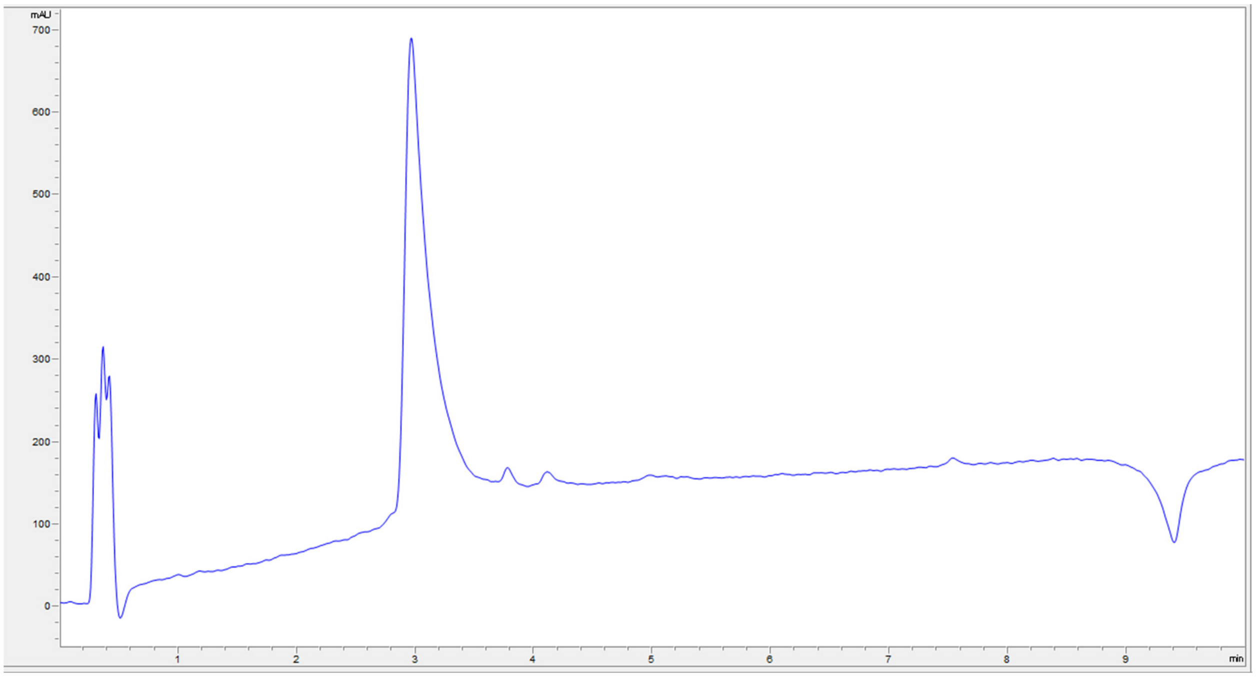

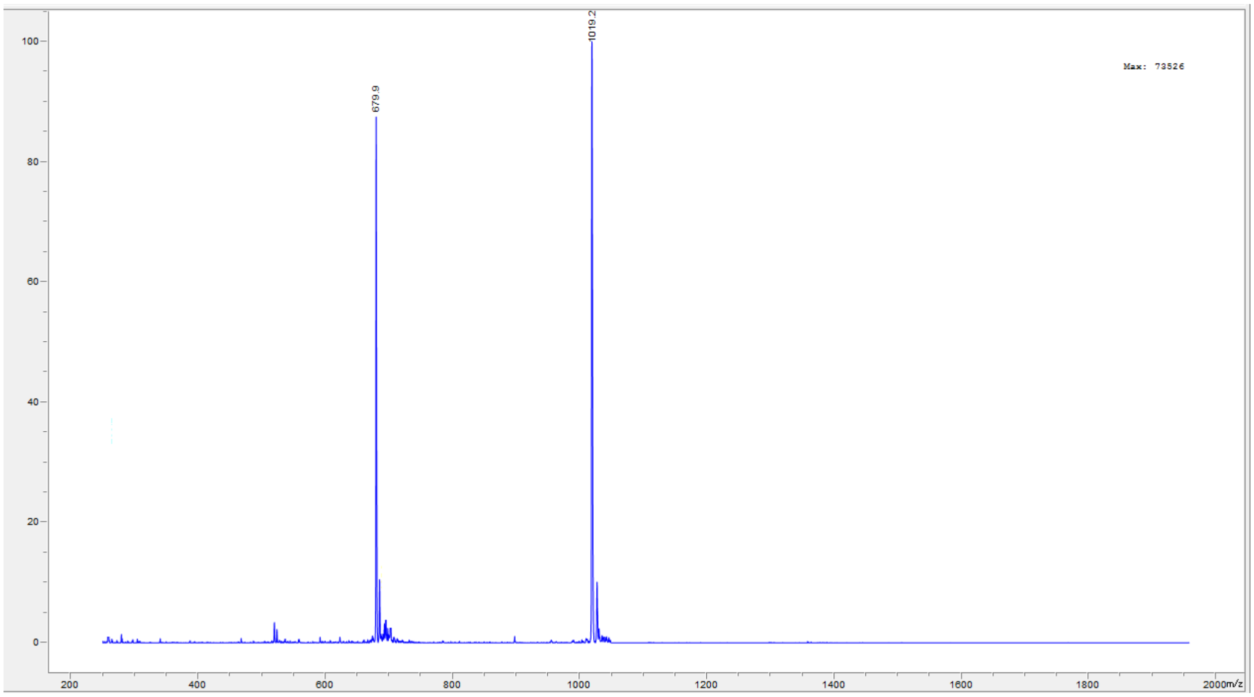

Supplementary Figure 68. LC-MS Spectra for peptide **37.**

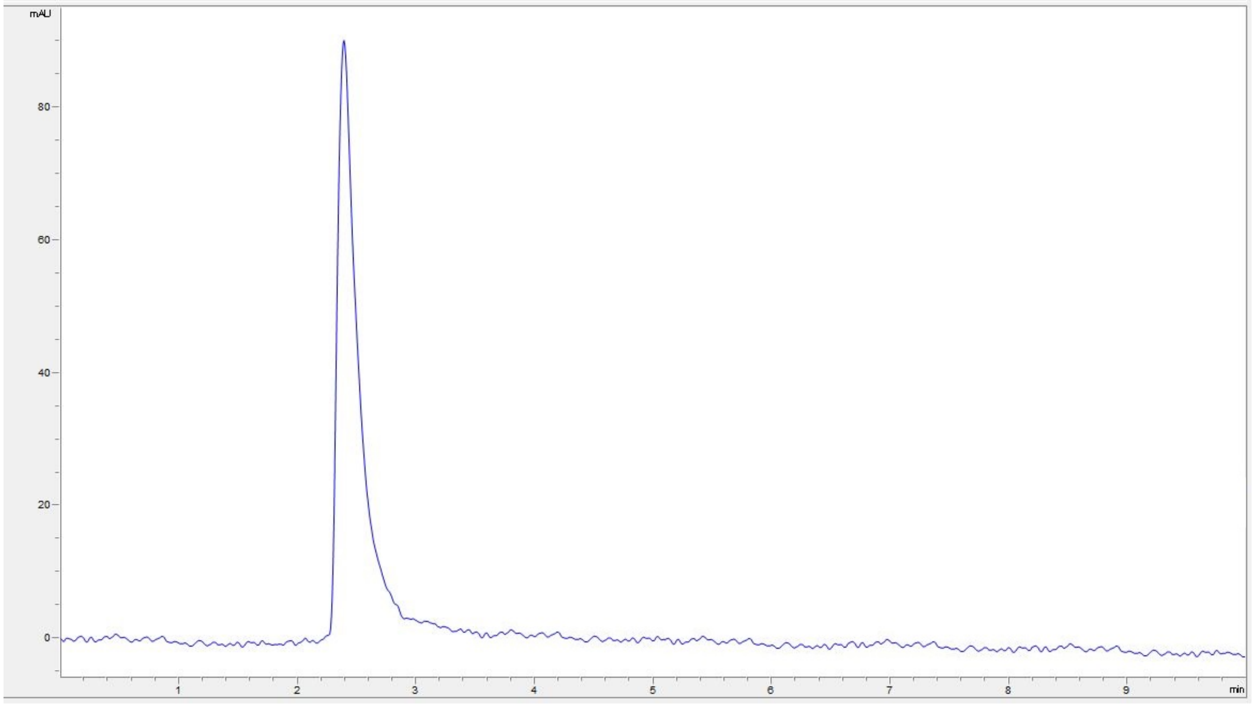

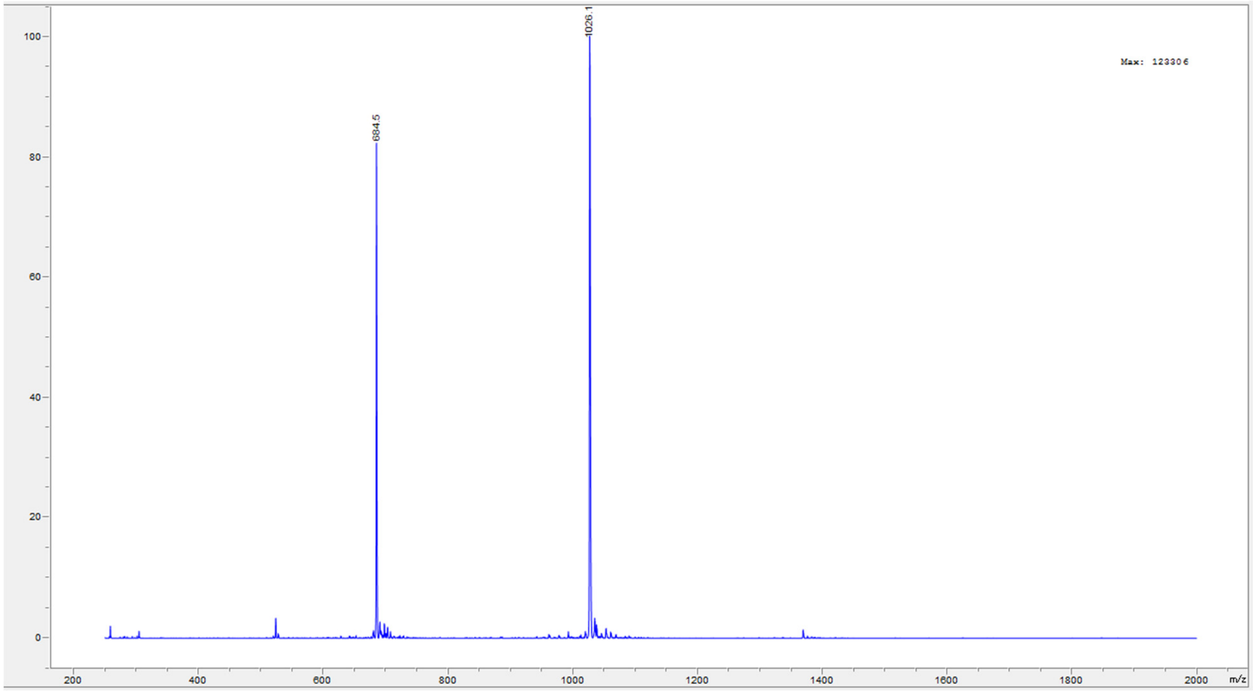

Supplementary Figure 69. LC-MS Spectra for peptide **38.**

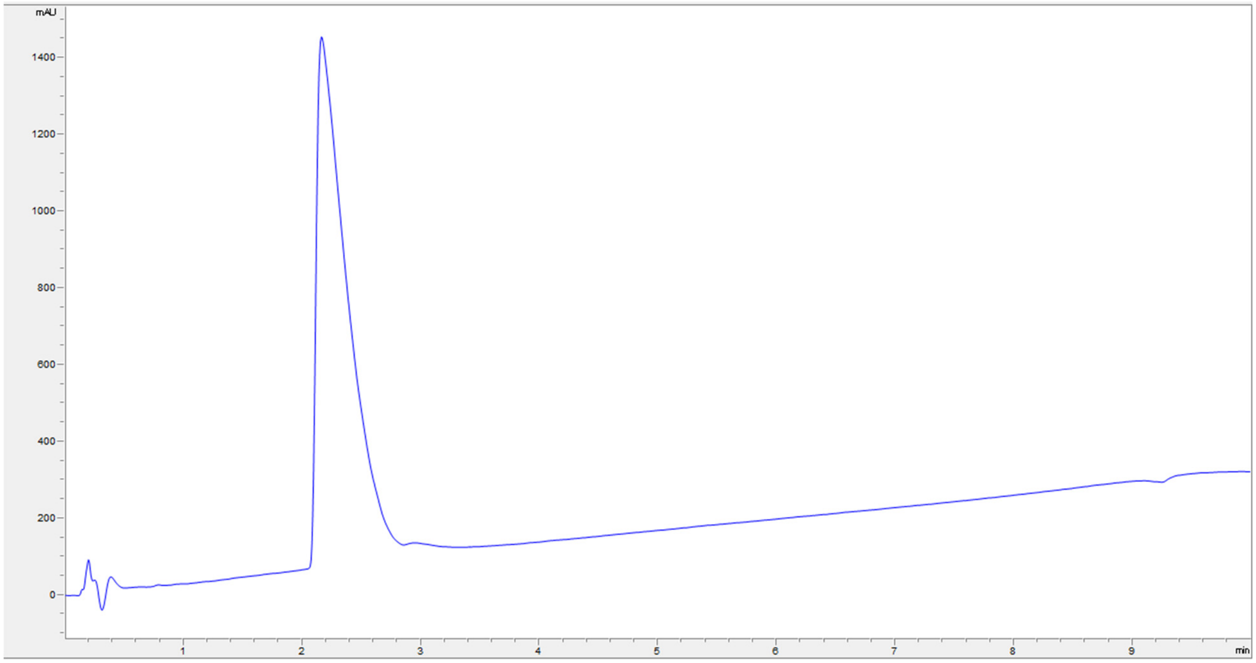

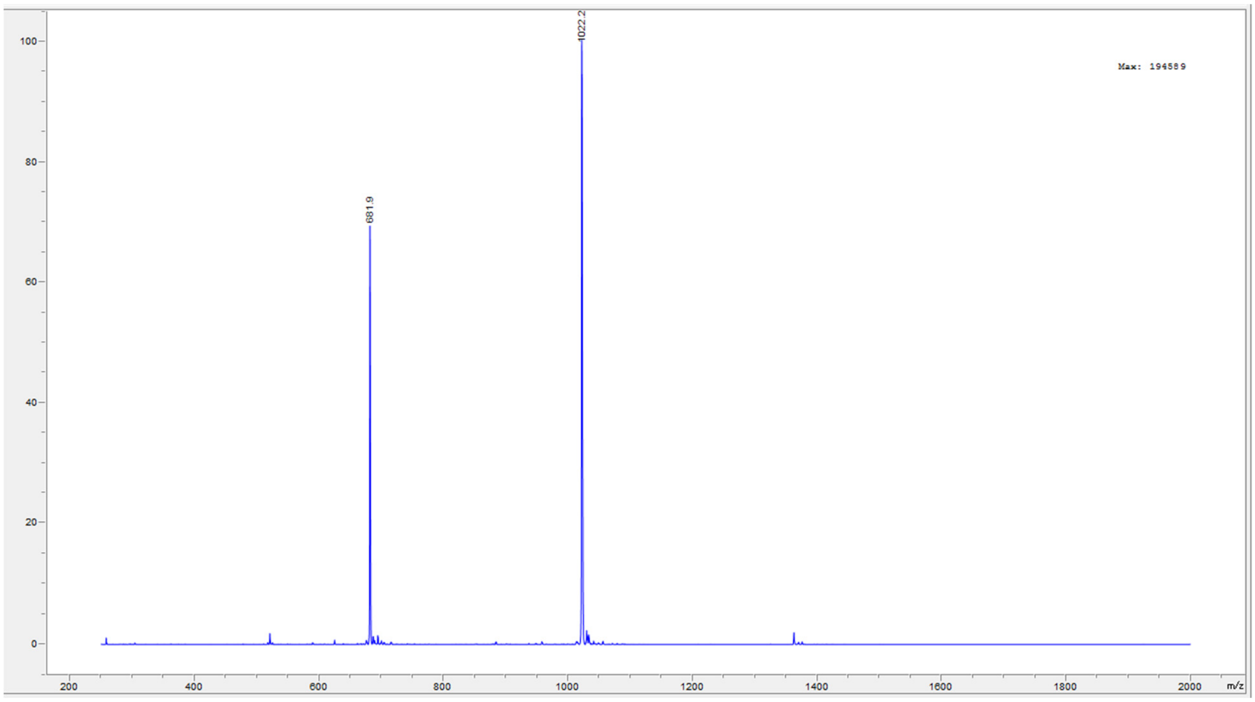

Supplementary Figure 70. LC-MS Spectra for peptide **39.**

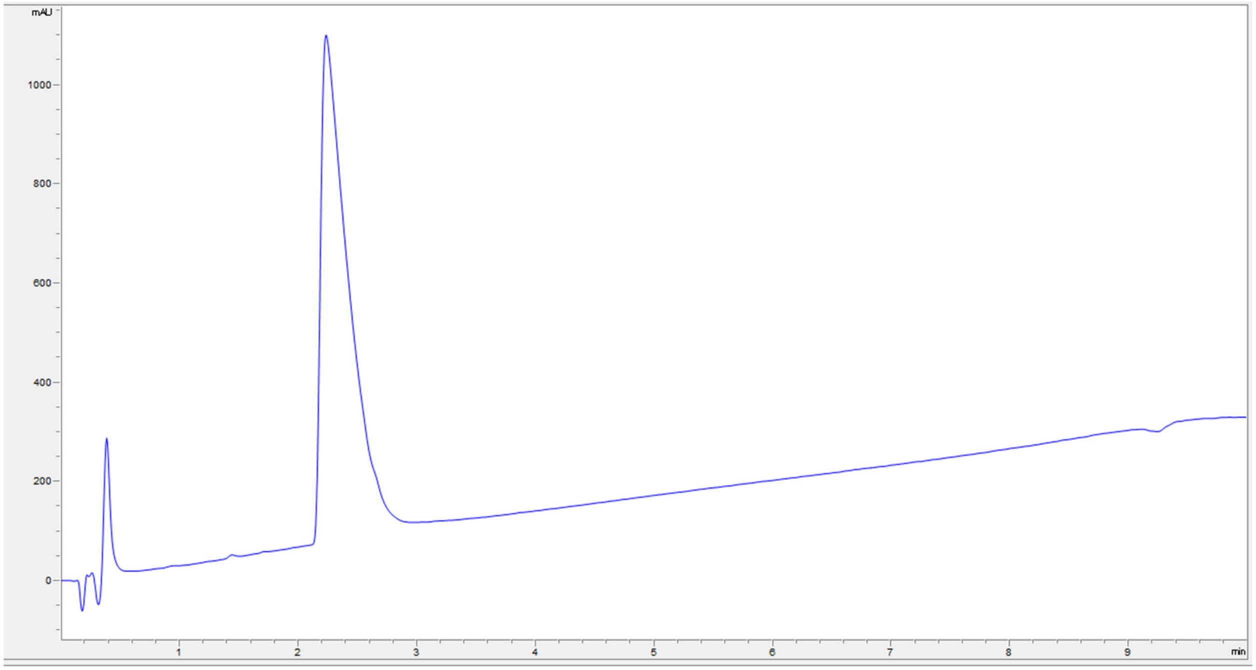

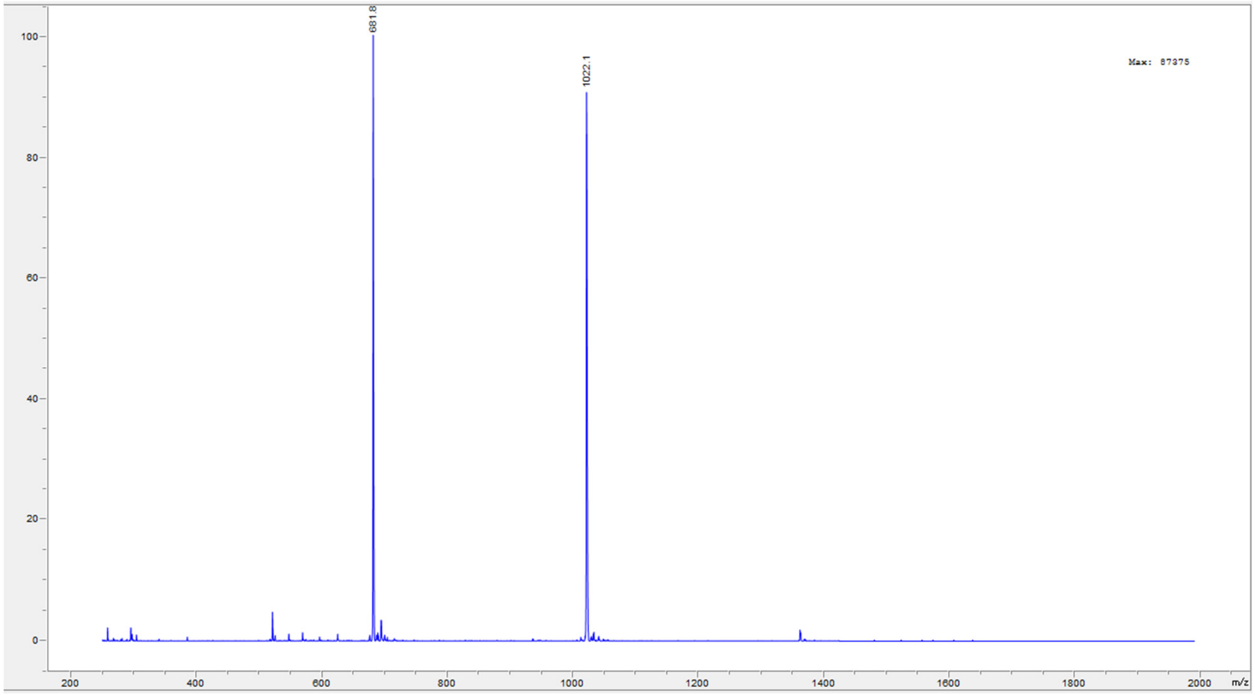

Supplementary Figure 71. LC-MS Spectra for peptide **40.**

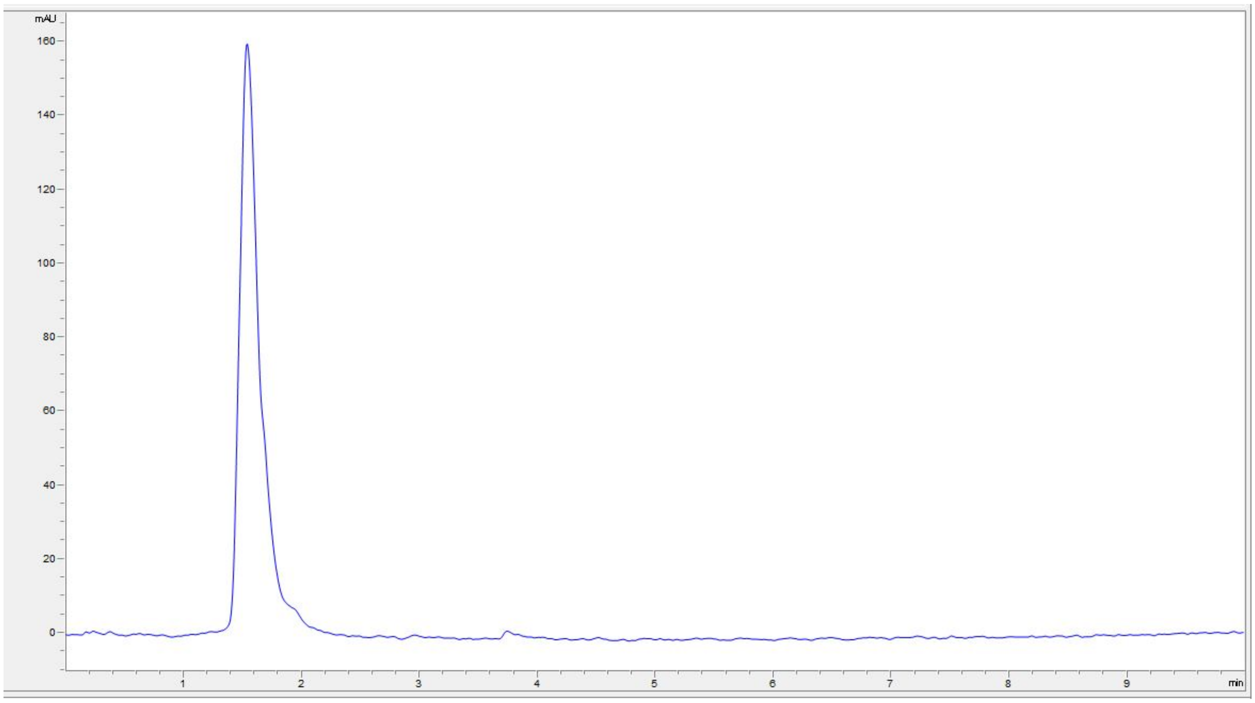

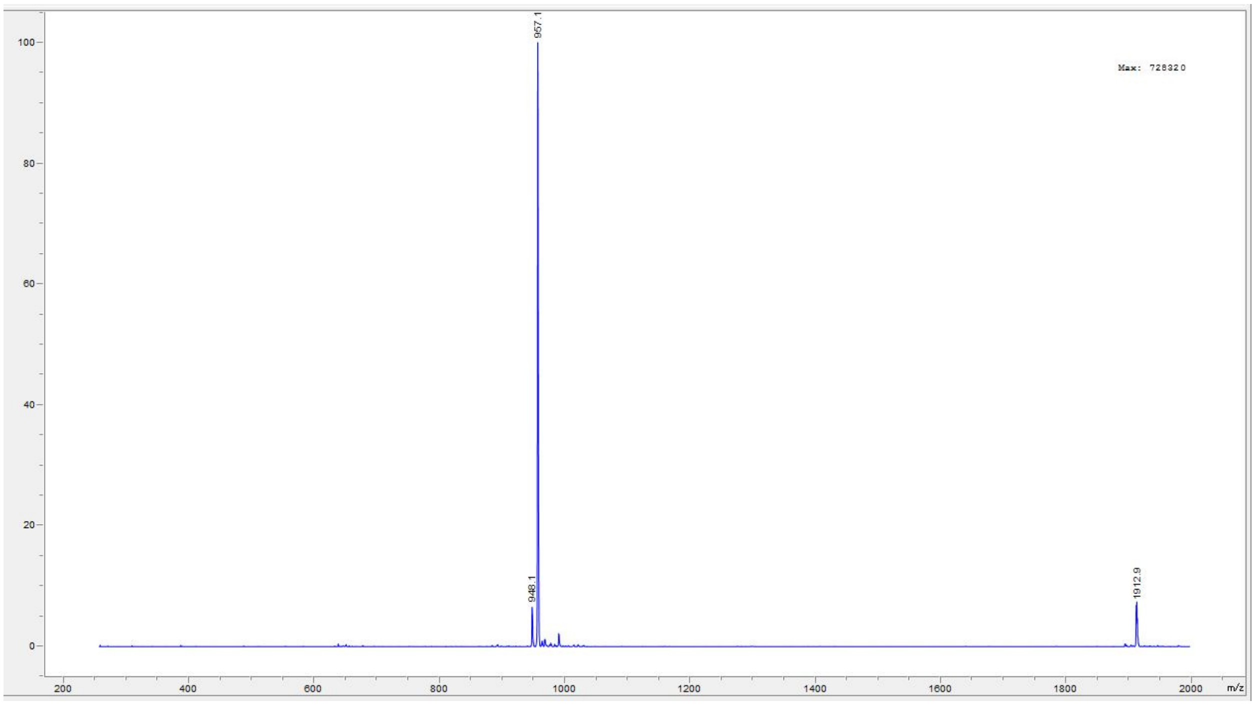

Supplementary Figure 72. LC-MS Spectra for peptide **41.**

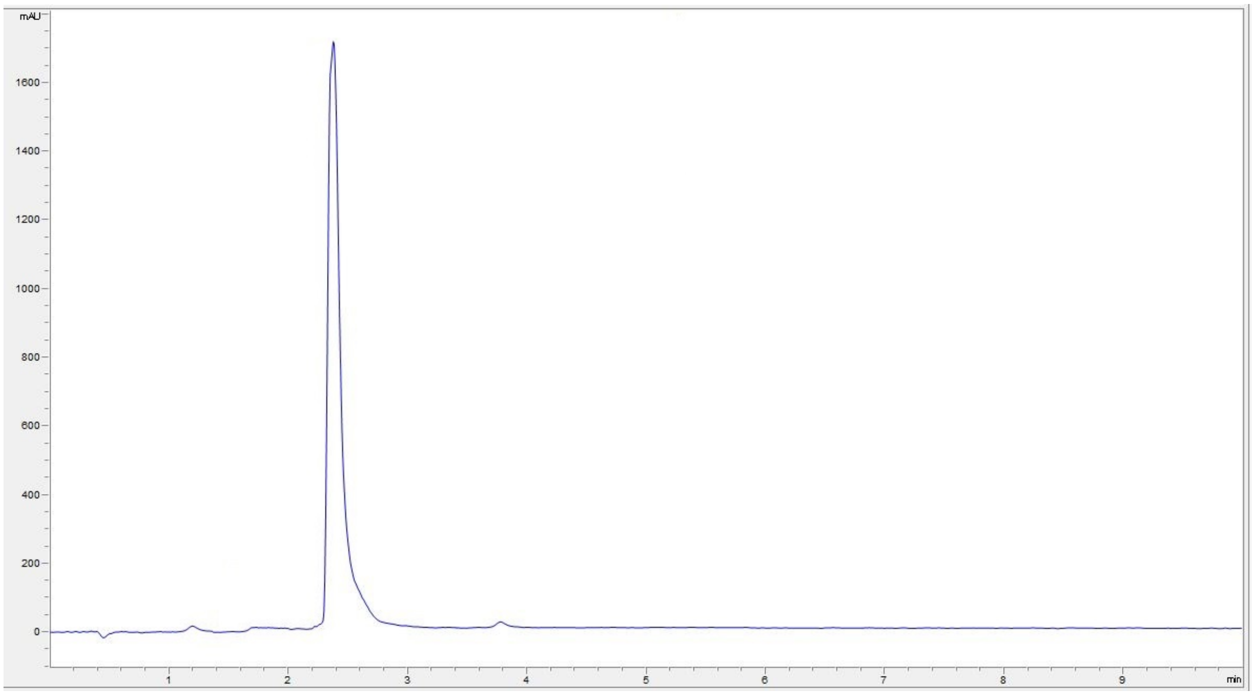

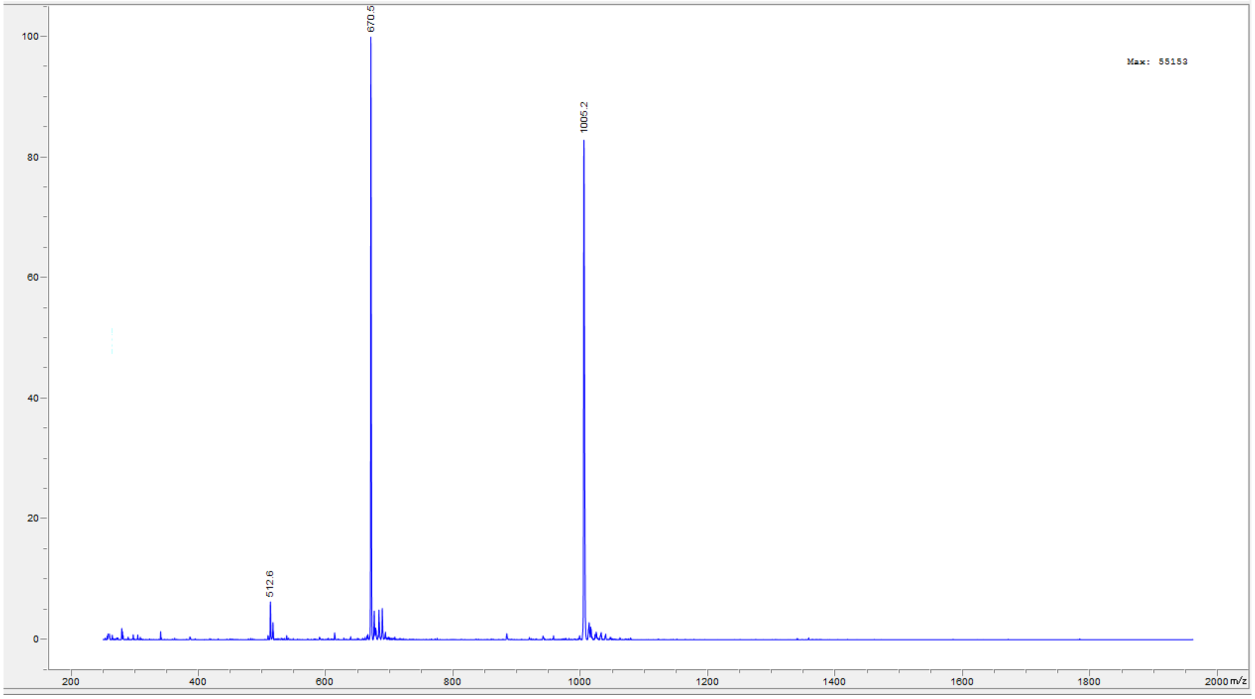

Supplementary Figure 73. LC-MS Spectra for peptide **42.**

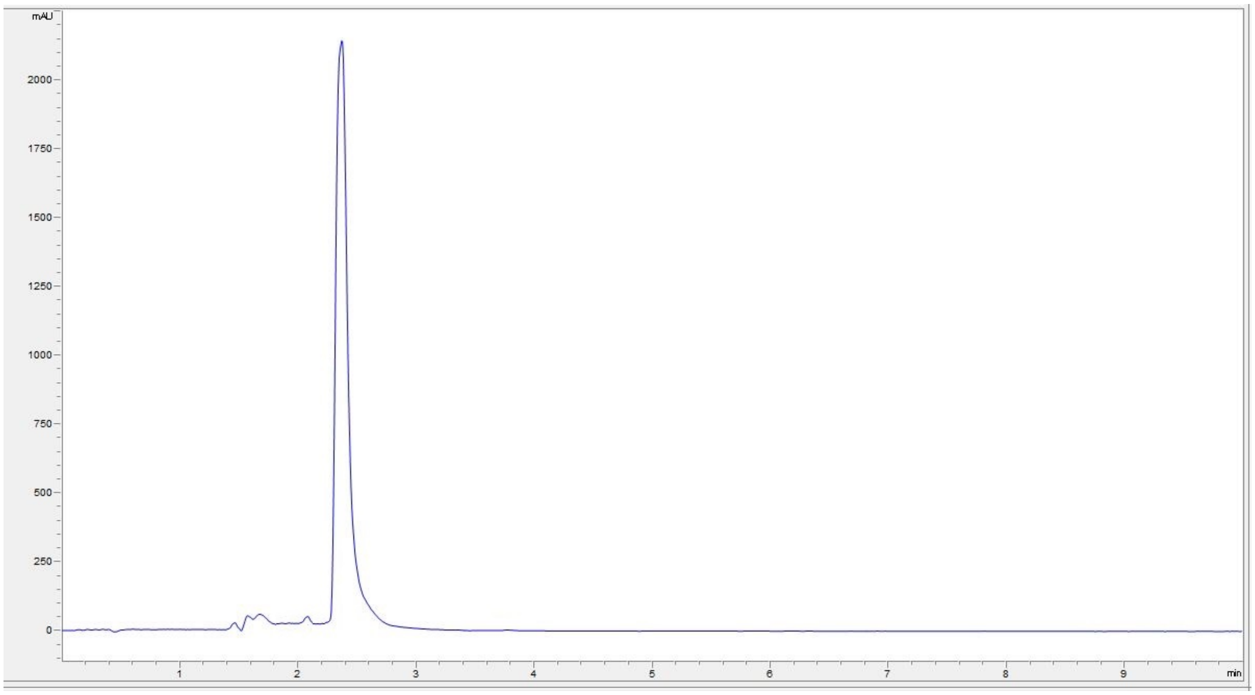

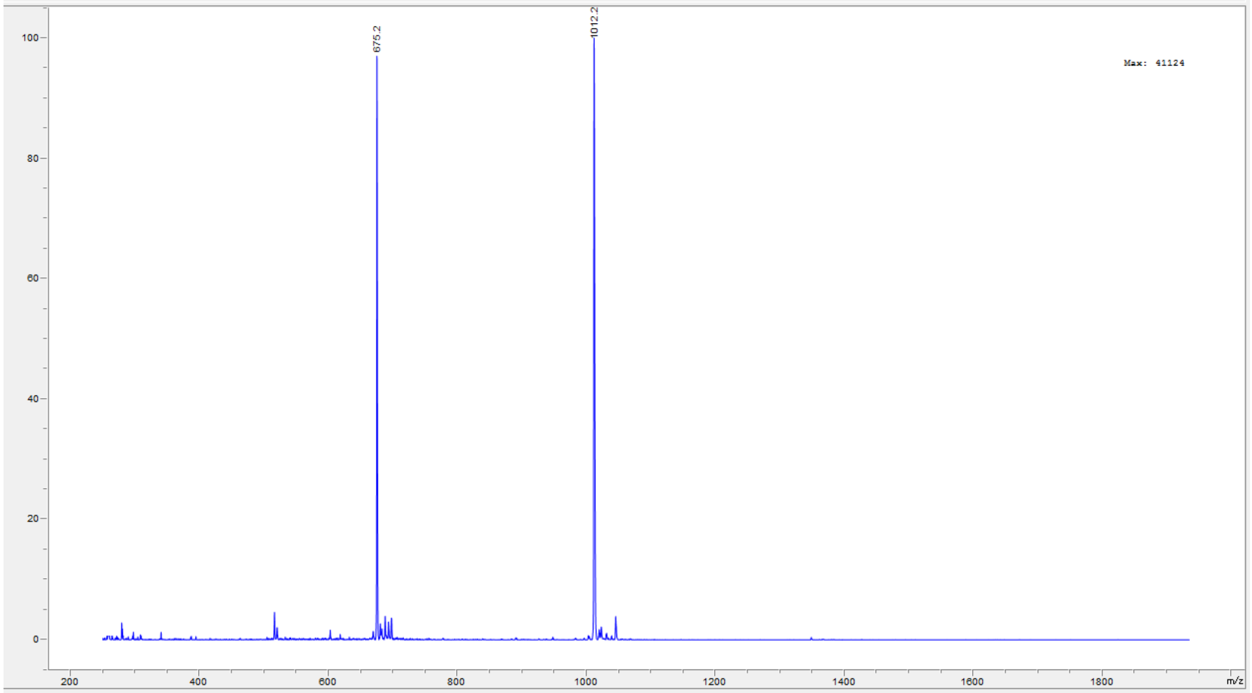

Supplementary Figure 74. LC-MS Spectra for peptide **43.**

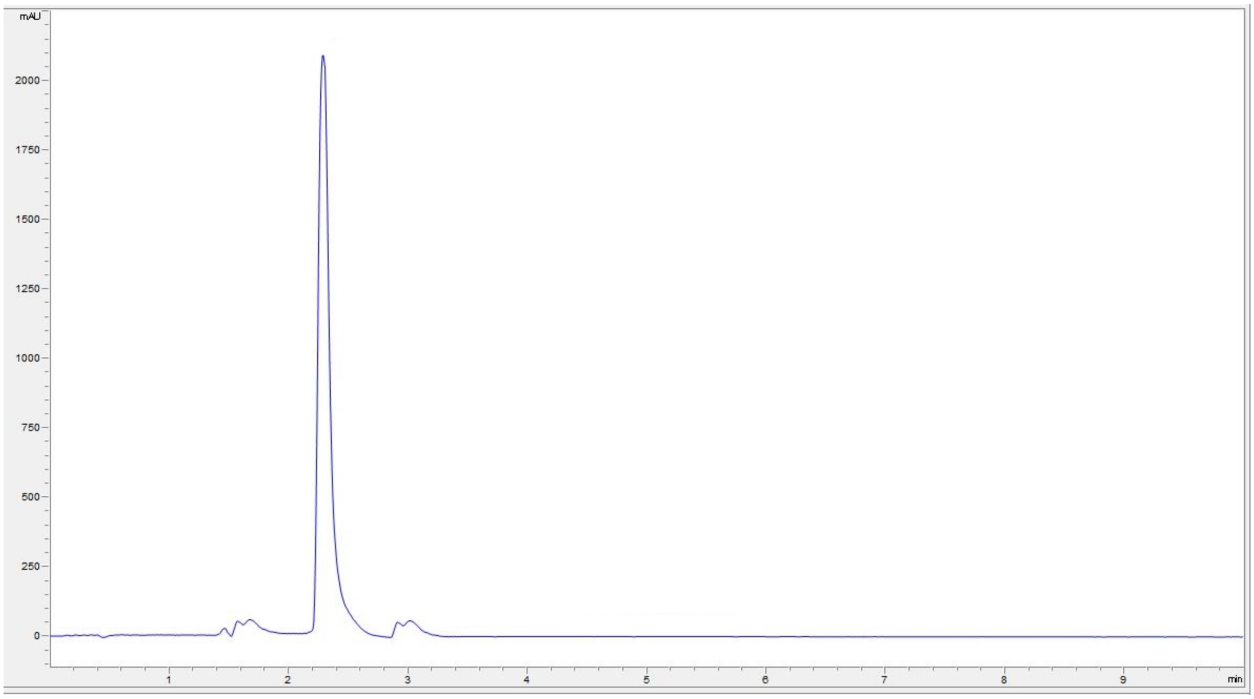

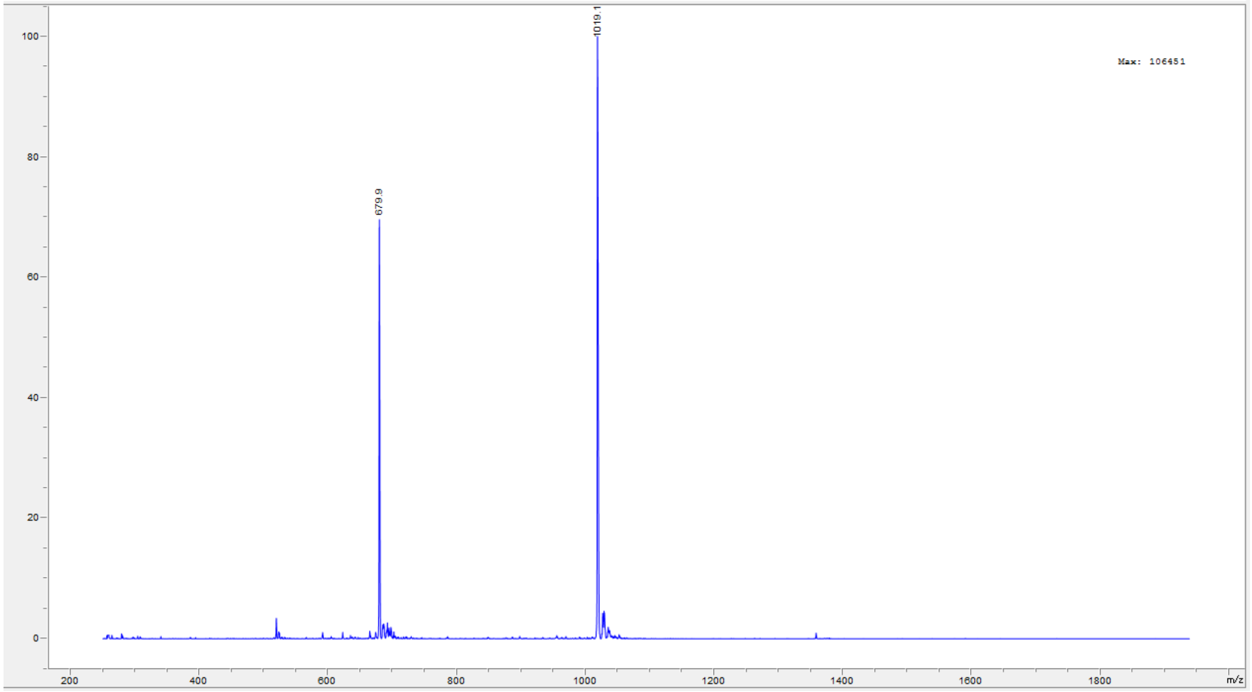

Supplementary Figure 75. LC-MS Spectra for peptide **44.**

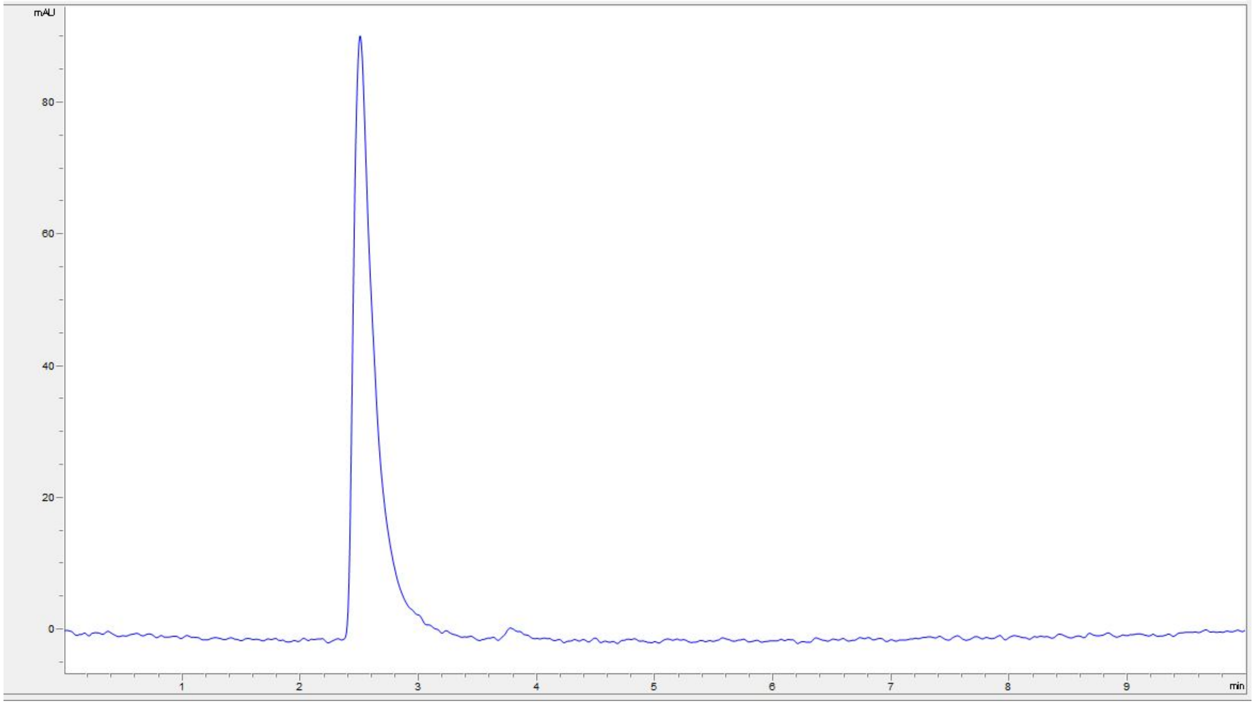

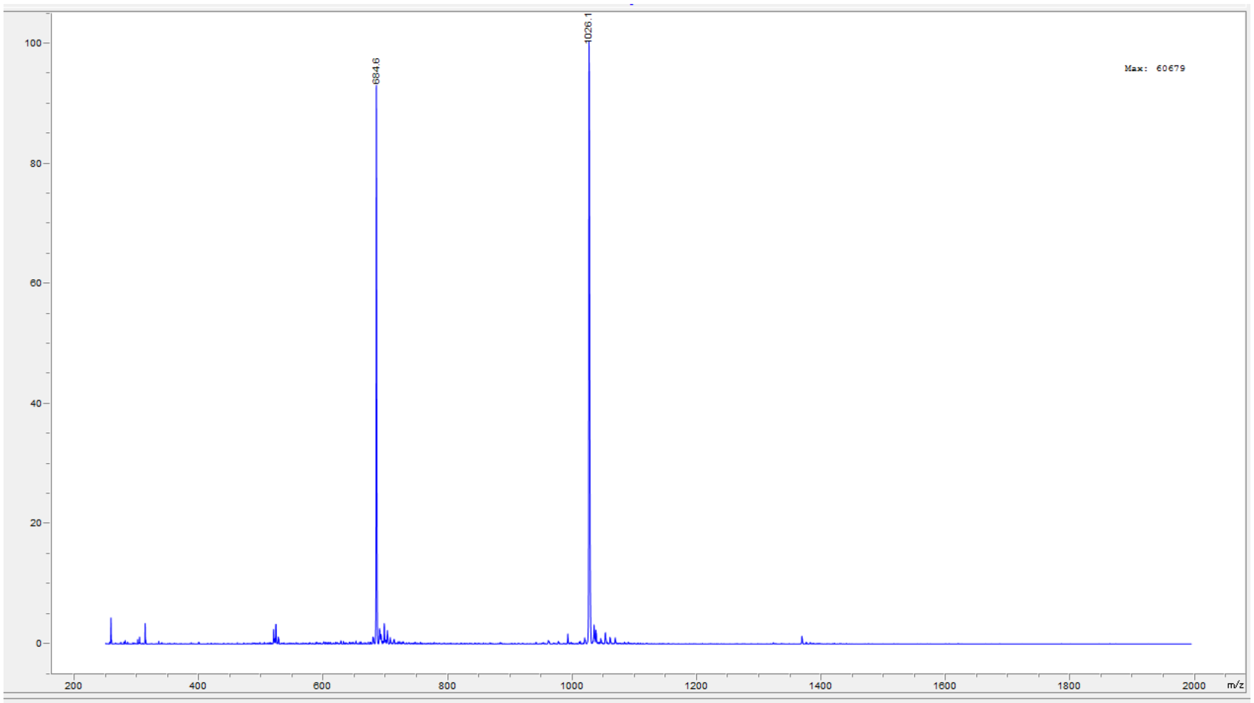

Supplementary Figure 76. LC-MS Spectra for peptide **45.**

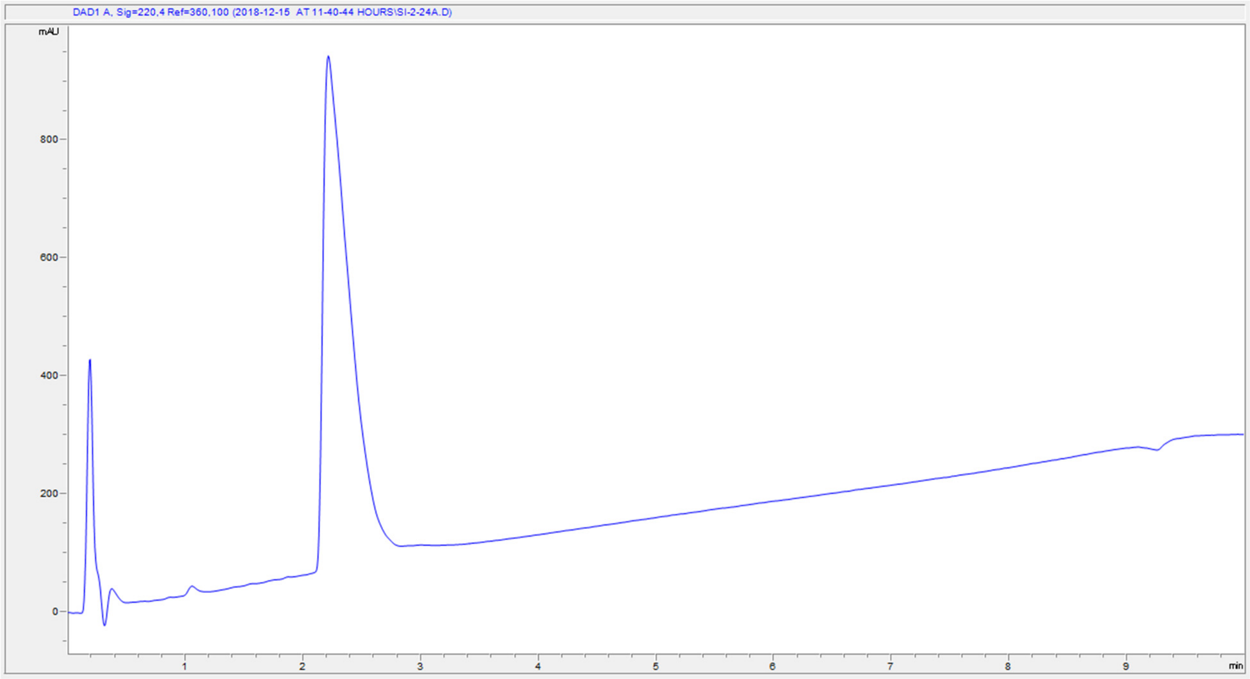

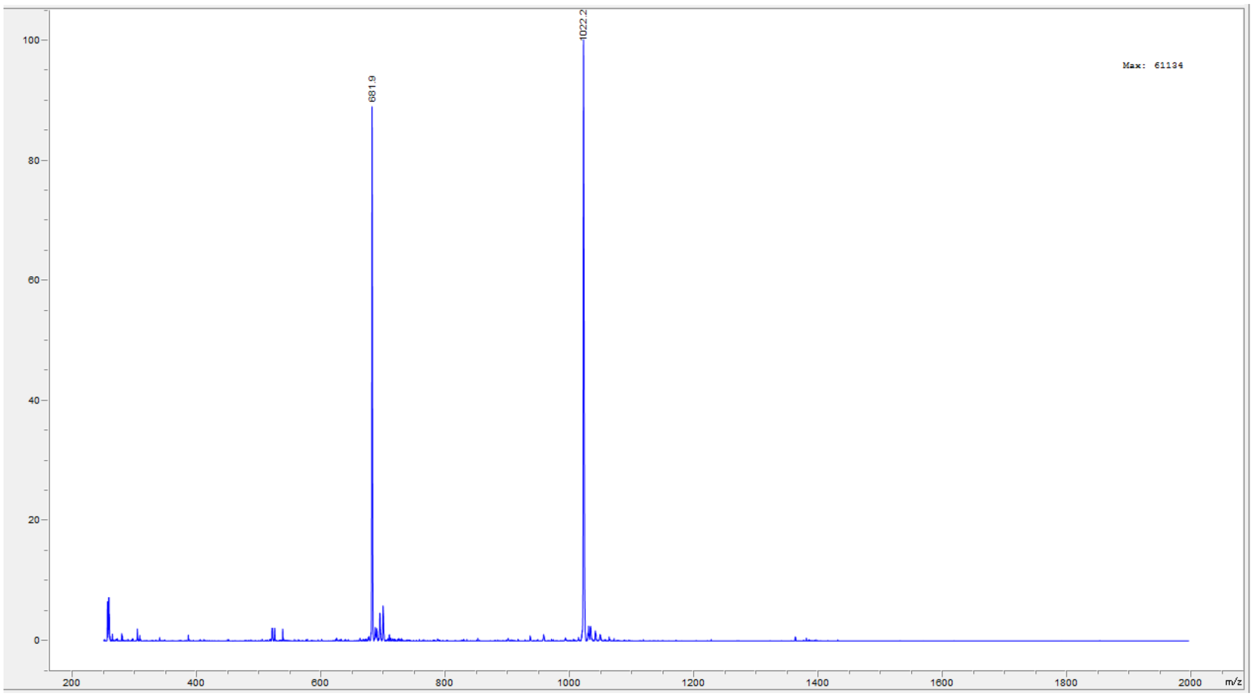

Supplementary Figure 77. LC-MS Spectra for peptide **46.**

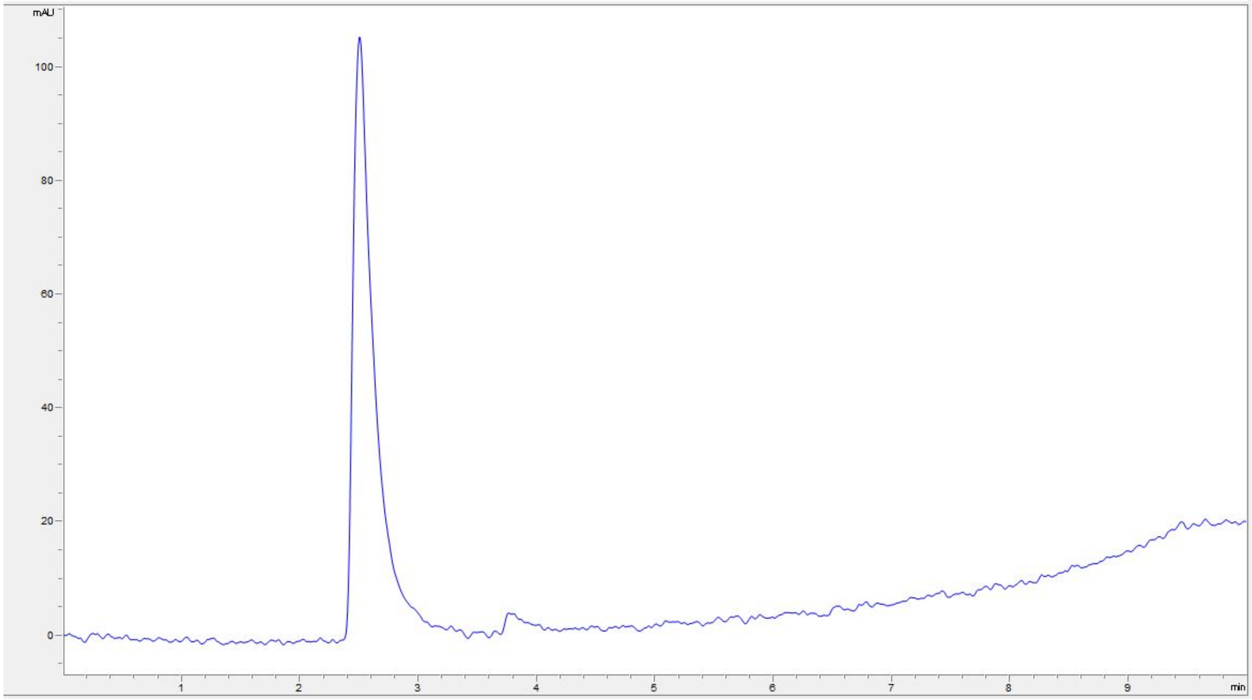

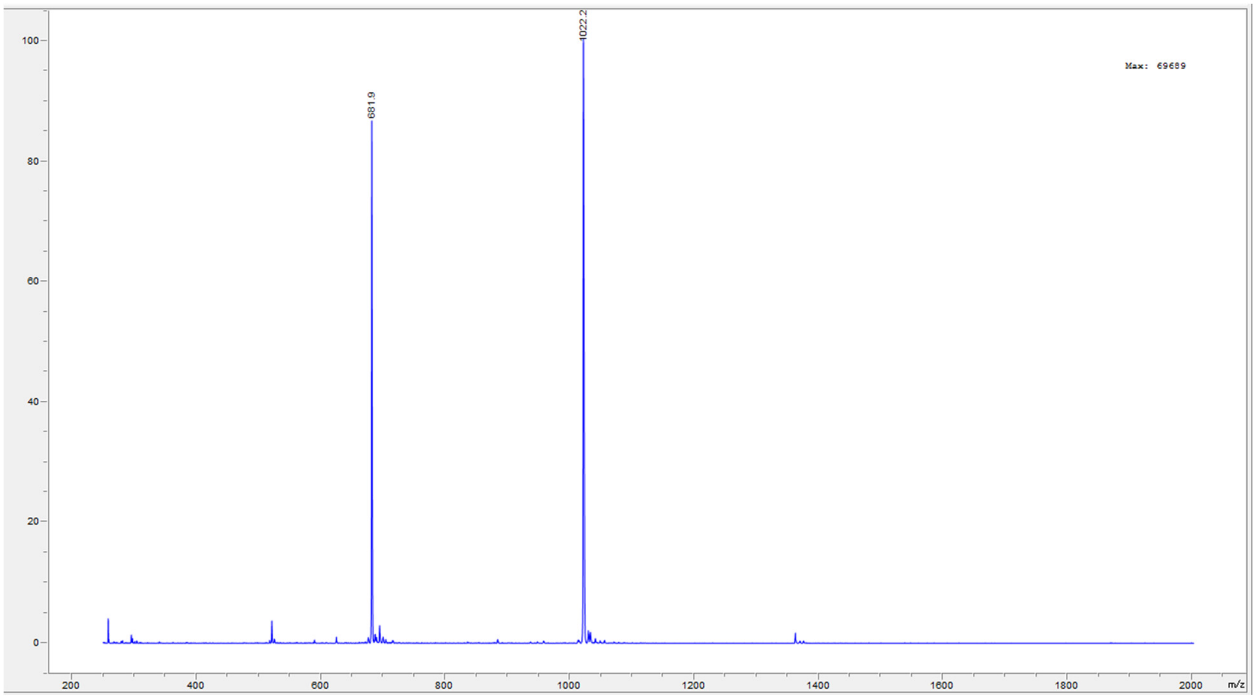

Supplementary Figure 78. LC-MS Spectra for peptide **47.**

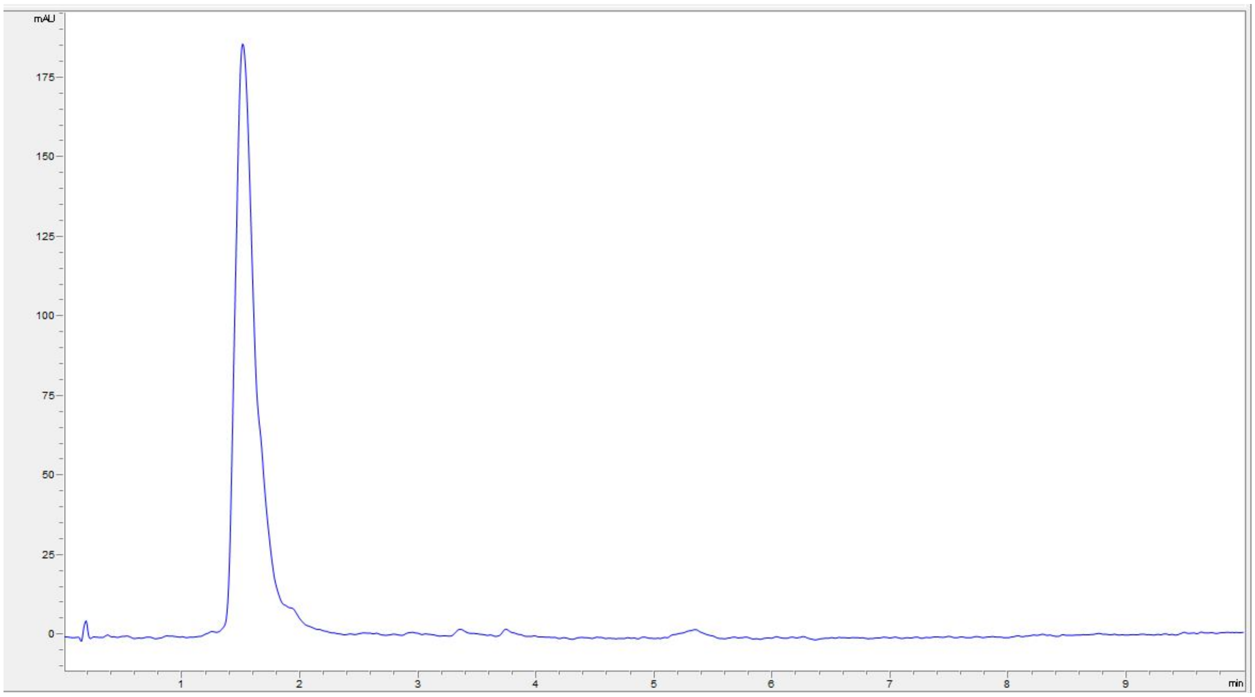

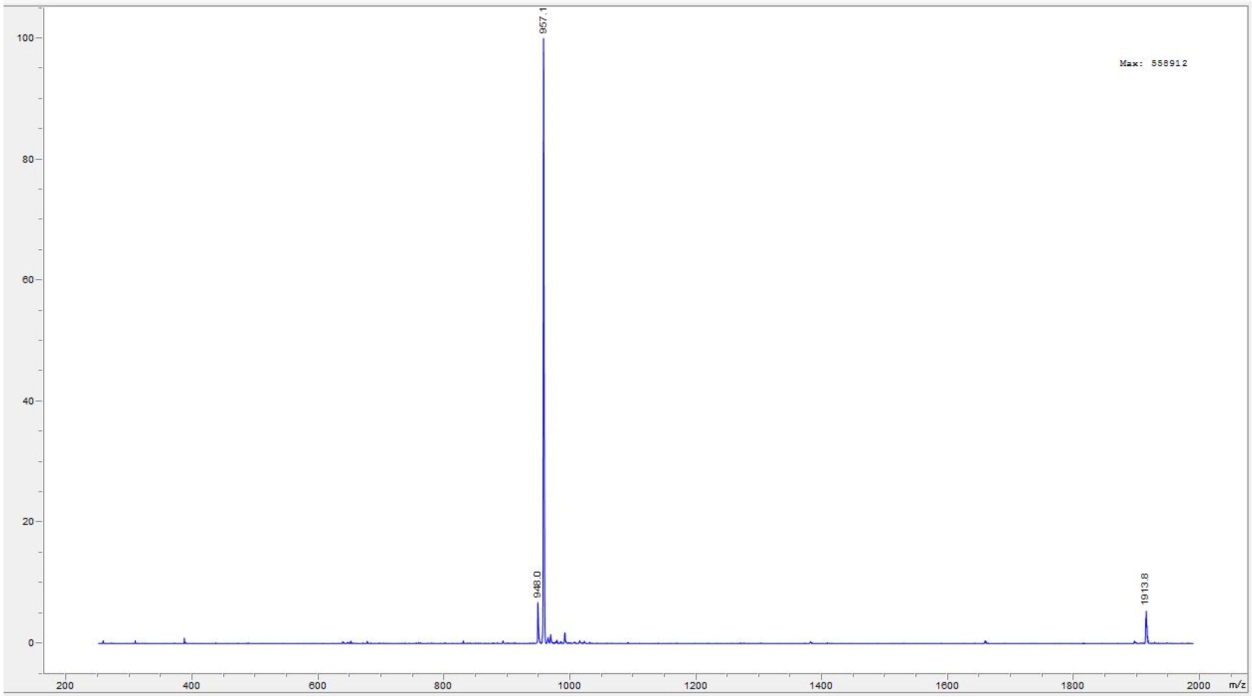

Supplementary Figure 79. LC-MS Spectra for peptide **48.**

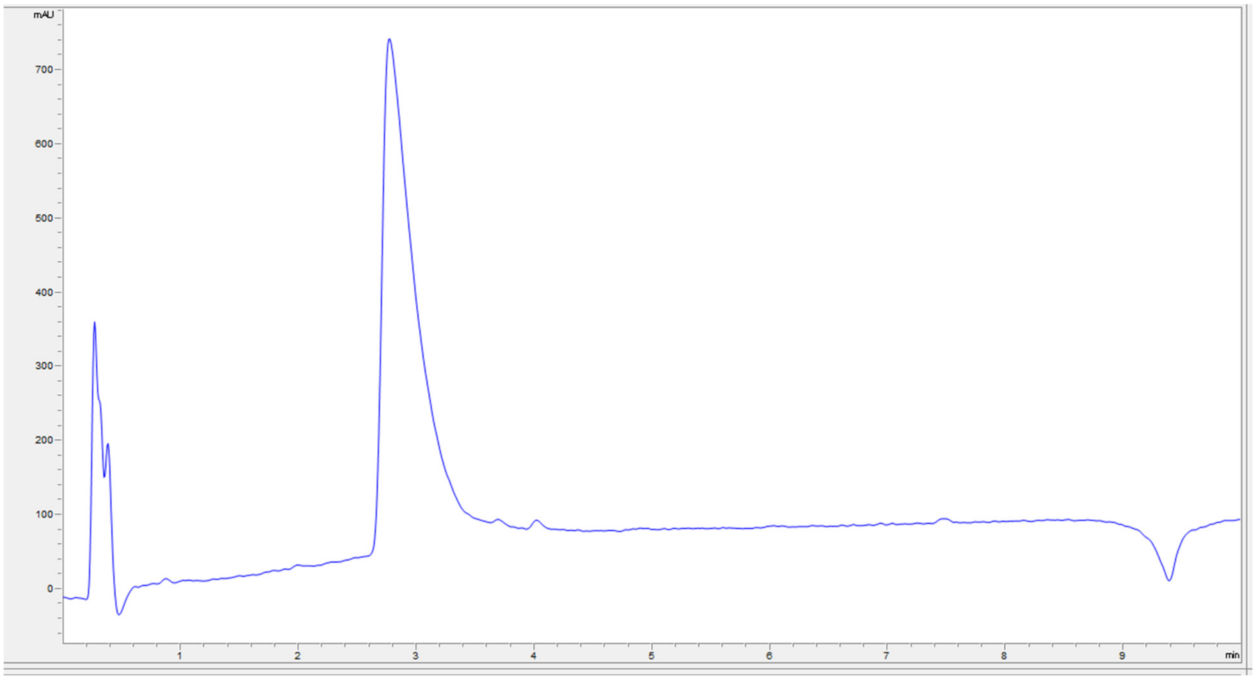

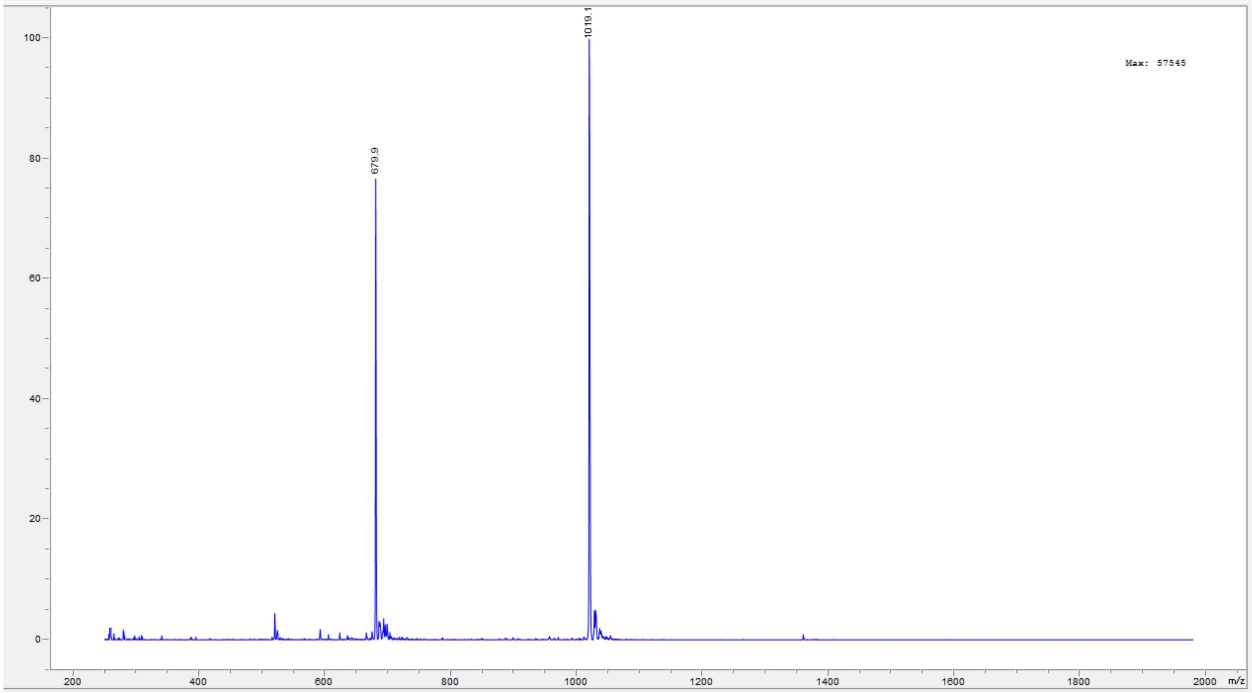

Supplementary Figure 80. LC-MS Spectra for peptide **49.**

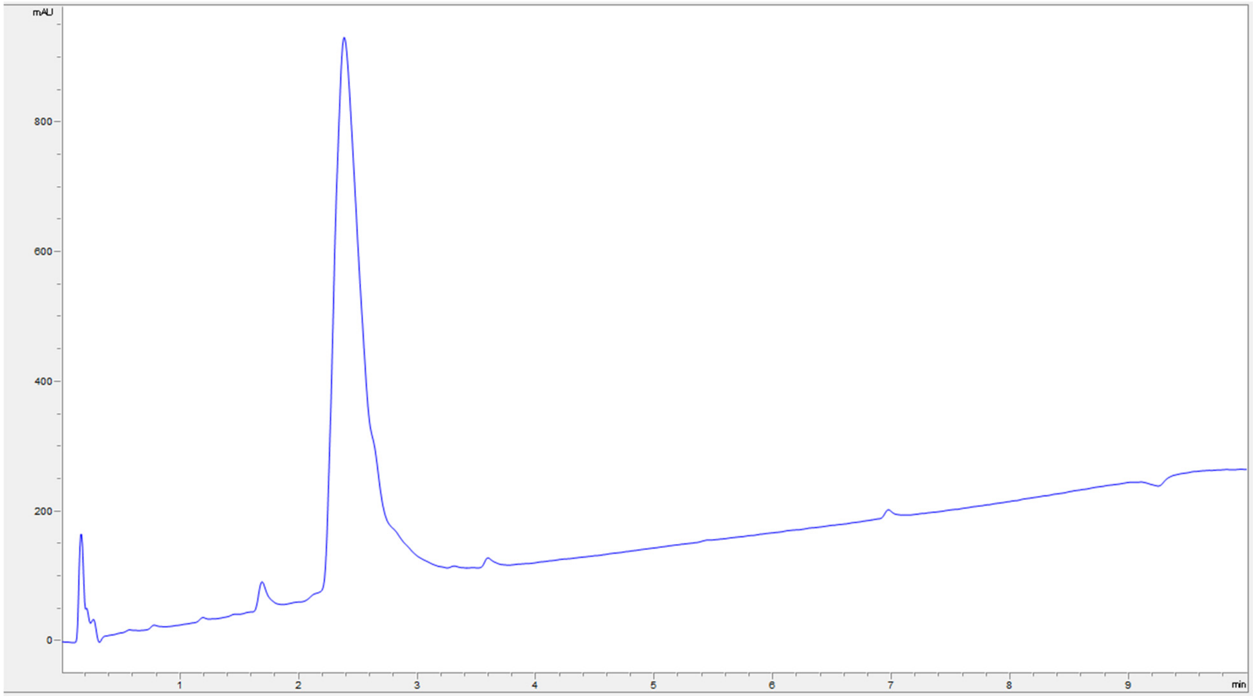

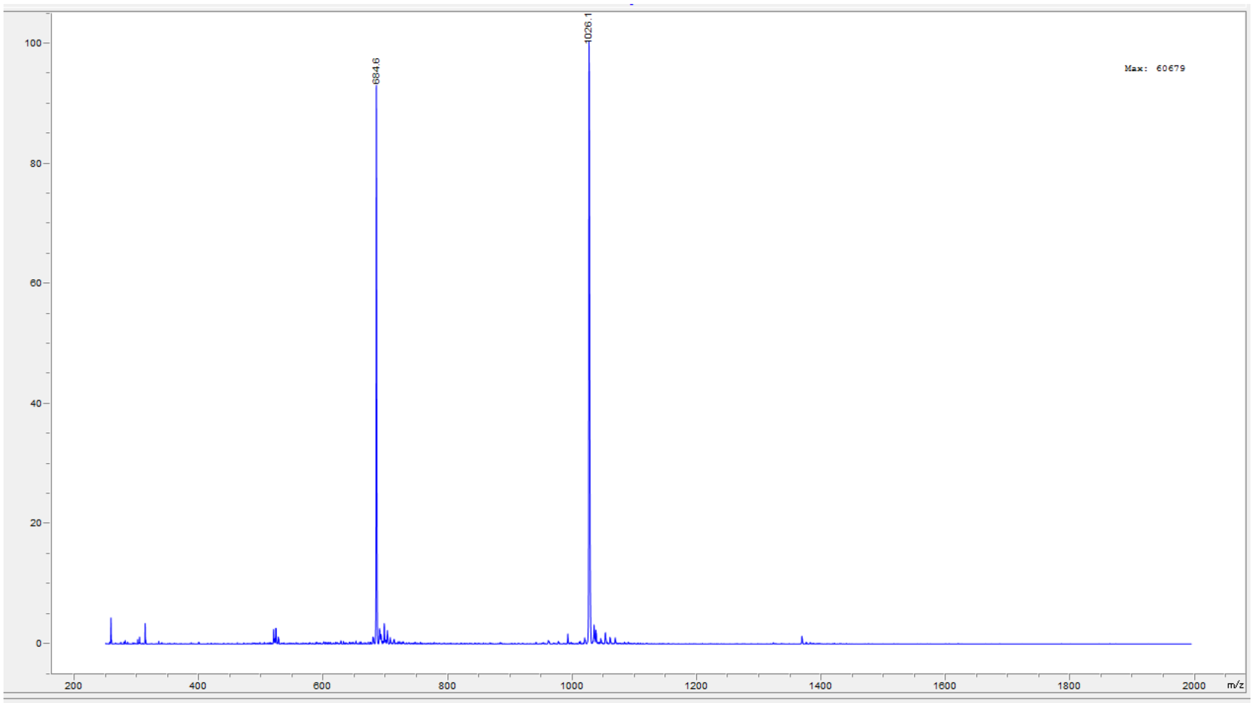

Supplementary Figure 81. LC-MS Spectra for peptide **50.**

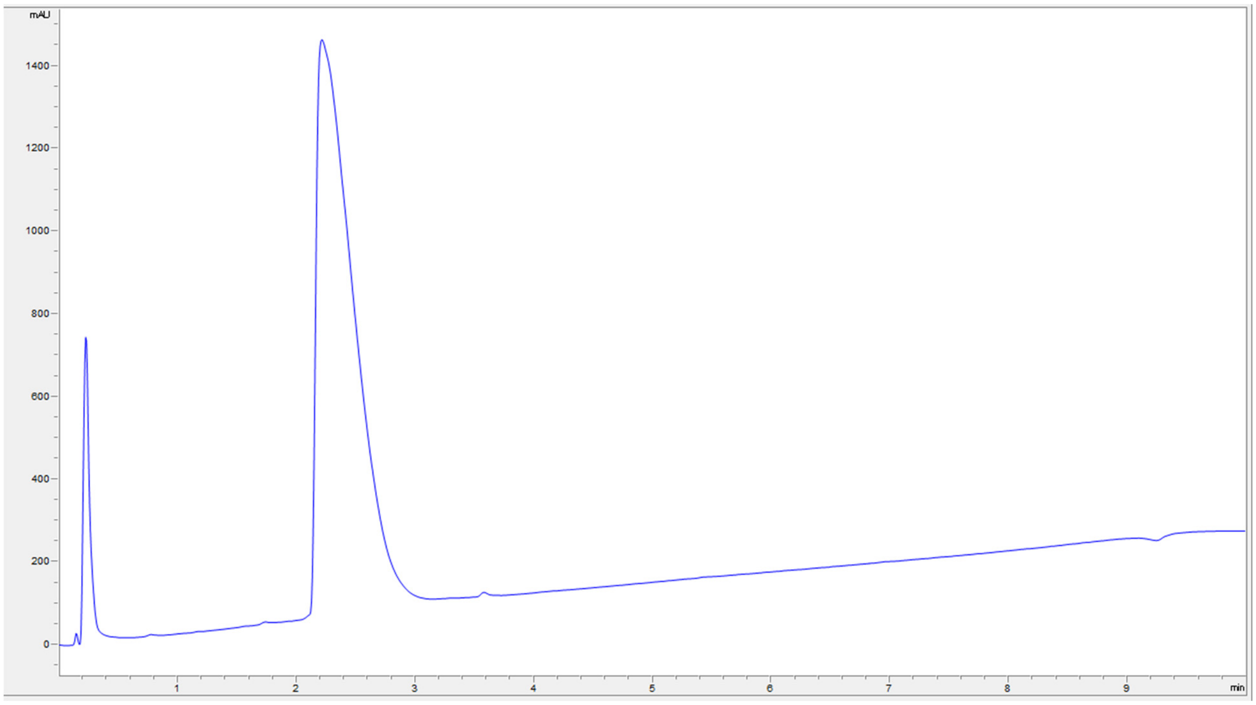

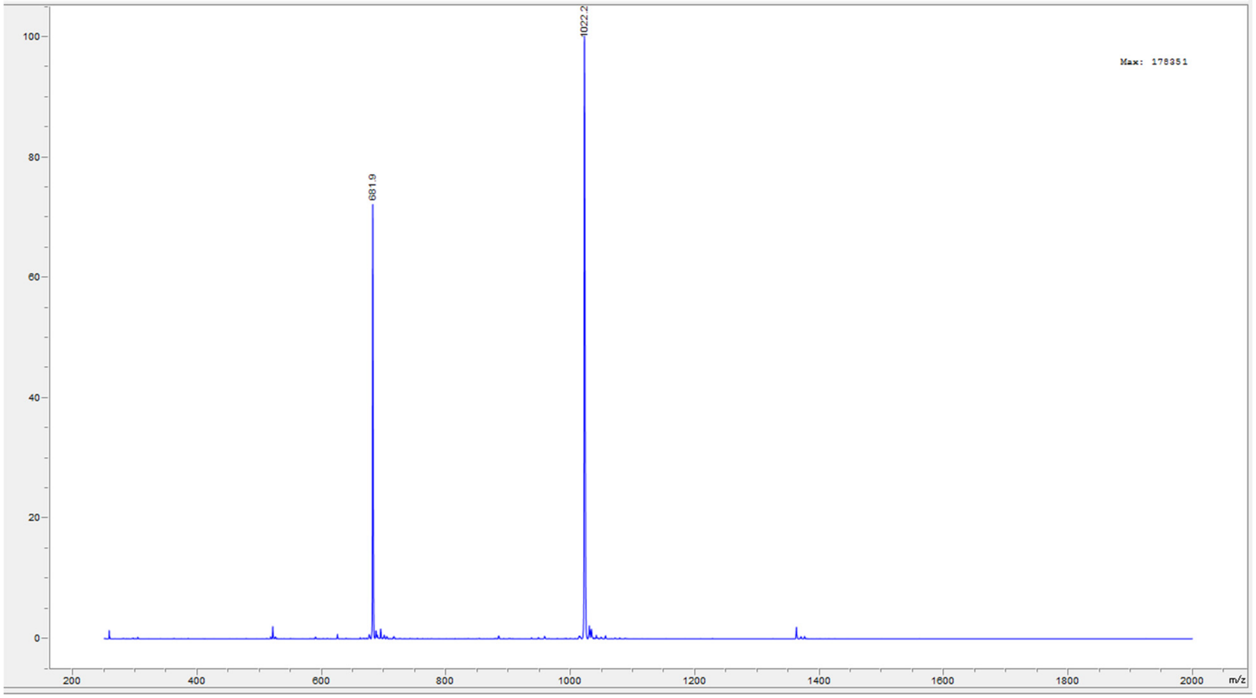

Supplementary Figure 82. LC-MS Spectra for peptide **51.**

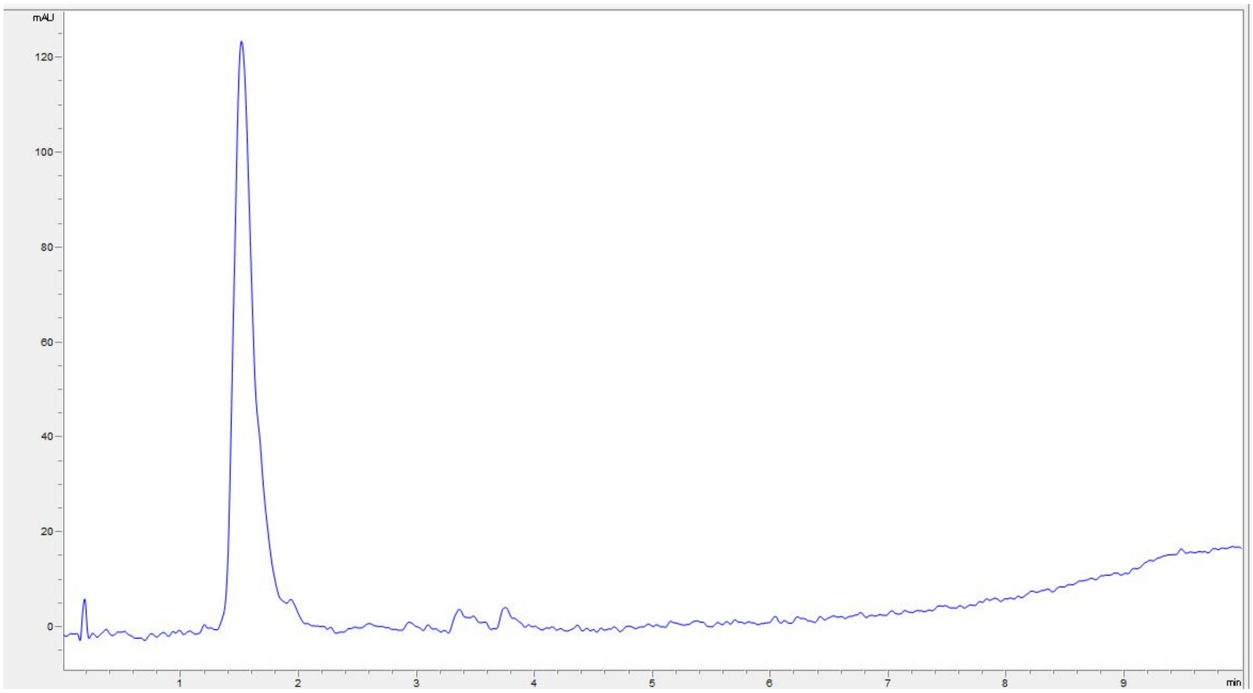

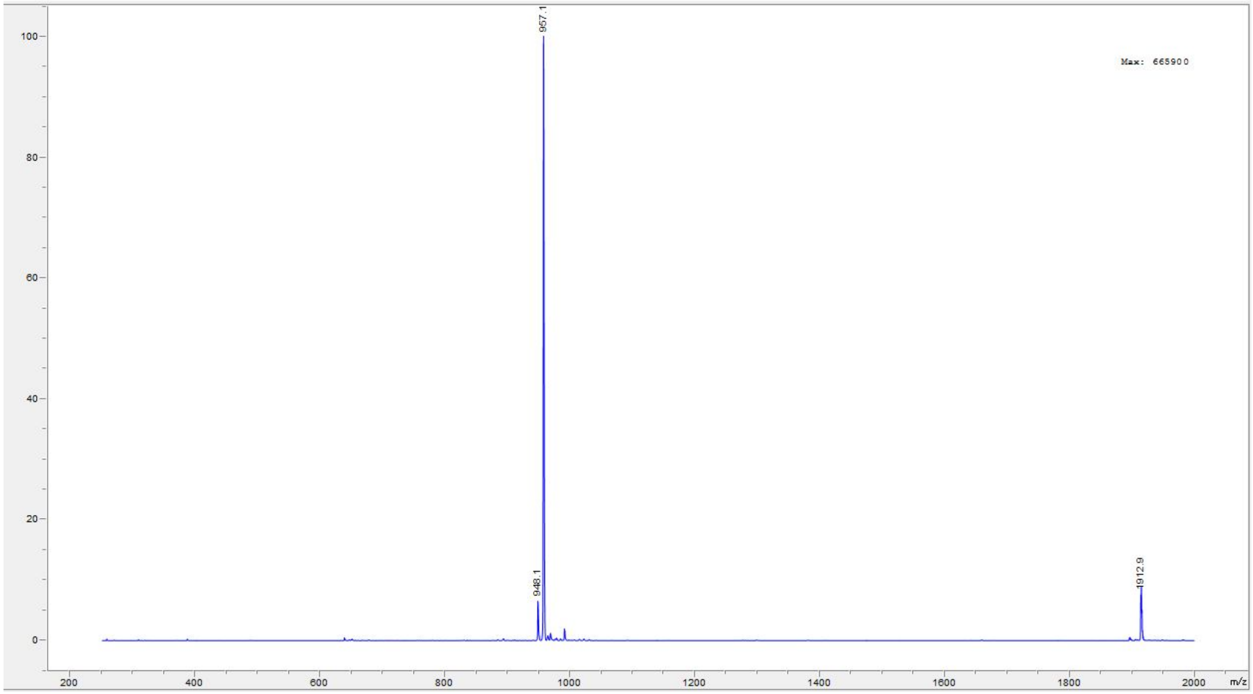

Supplementary Figure 83. LC-MS Spectra for peptide **52.**

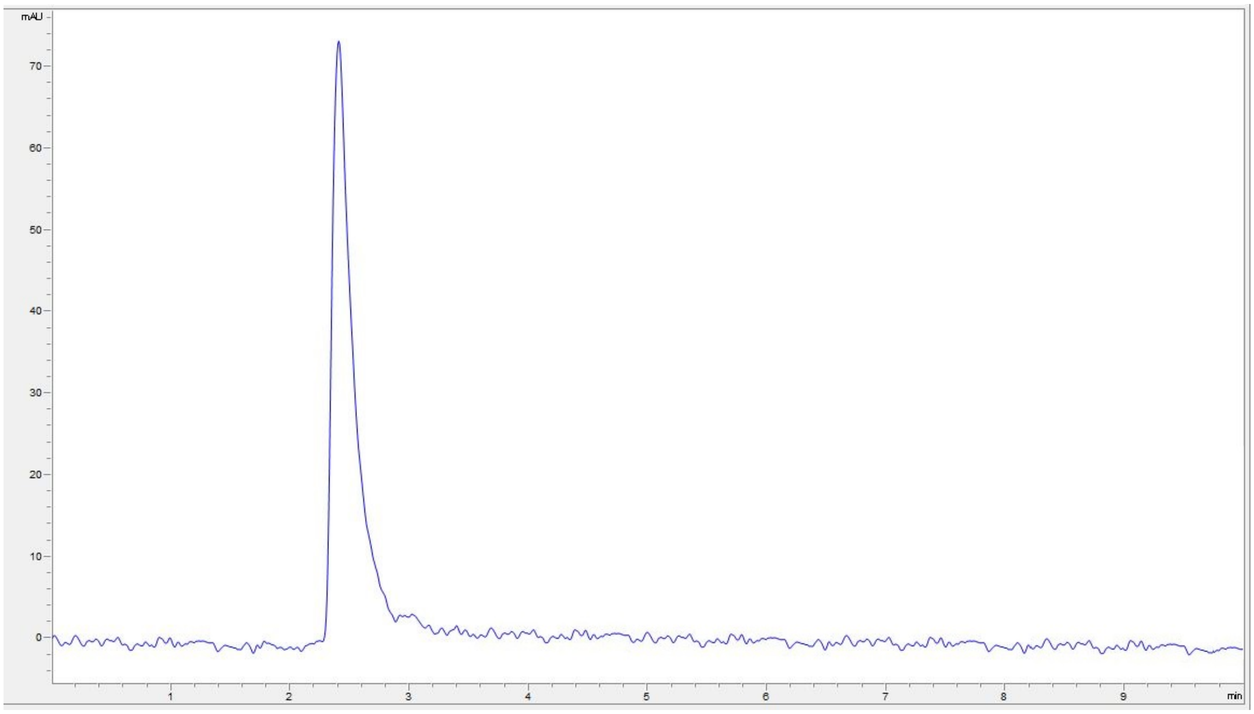

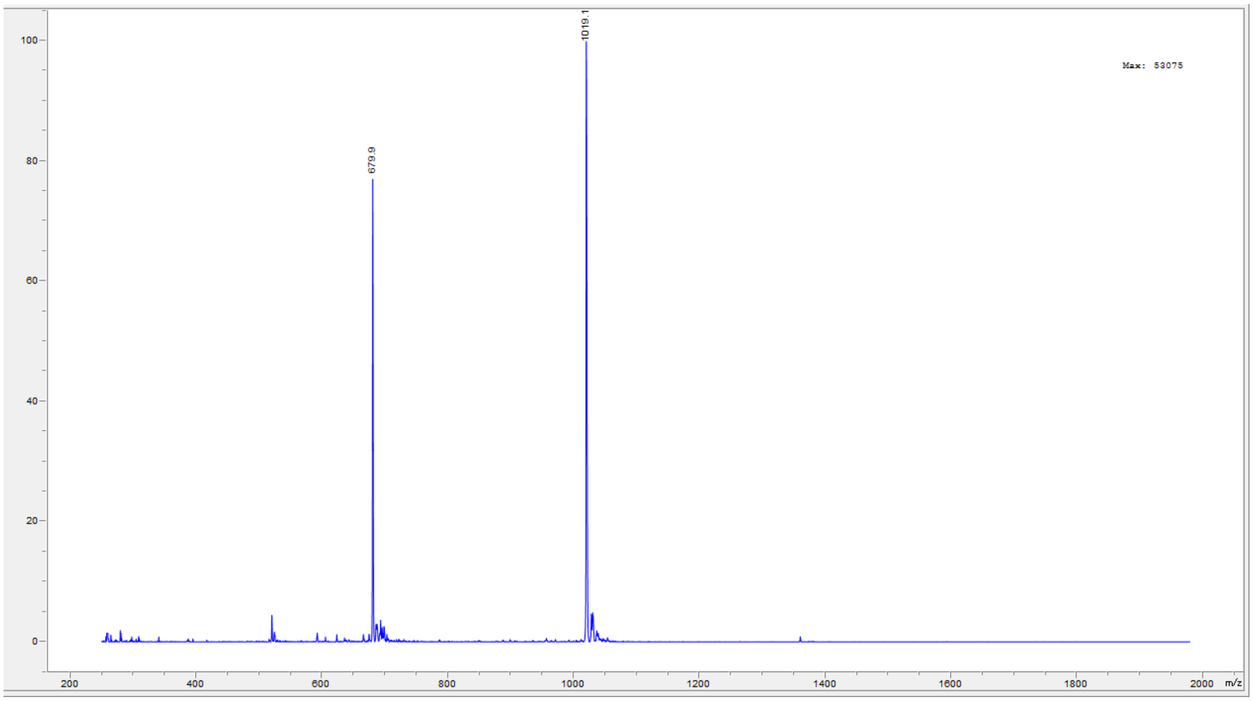

Supplementary Figure 84. LC-MS Spectra for peptide **53.**

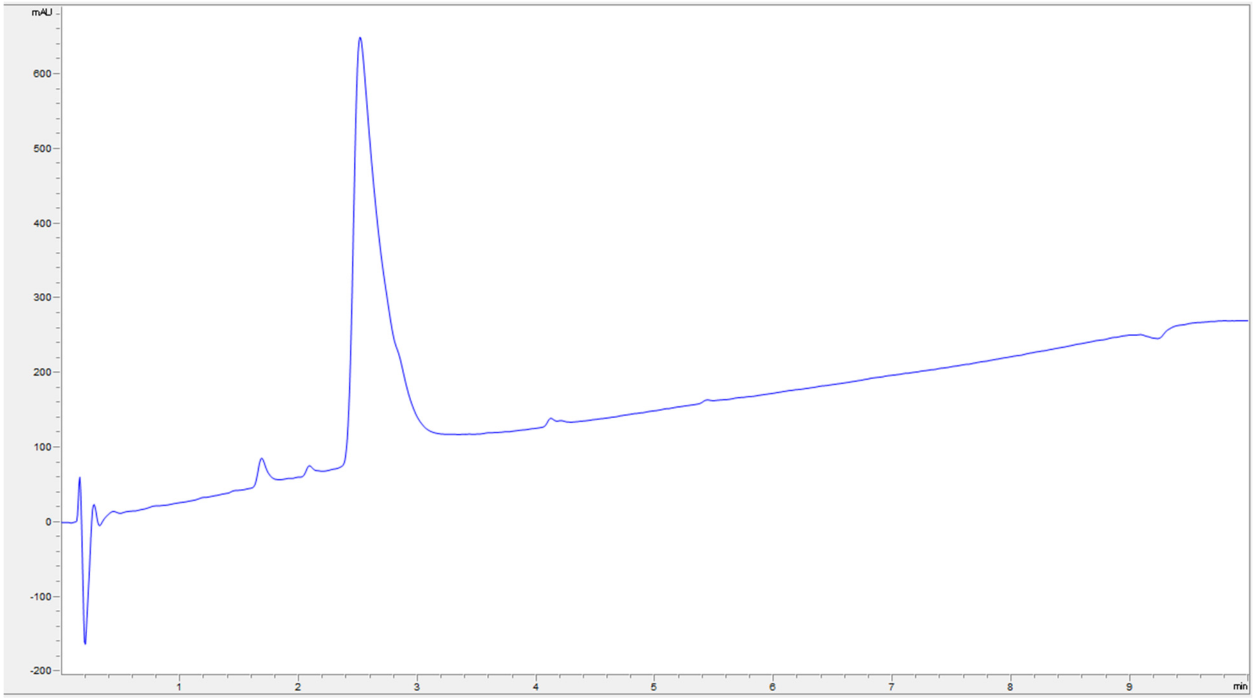

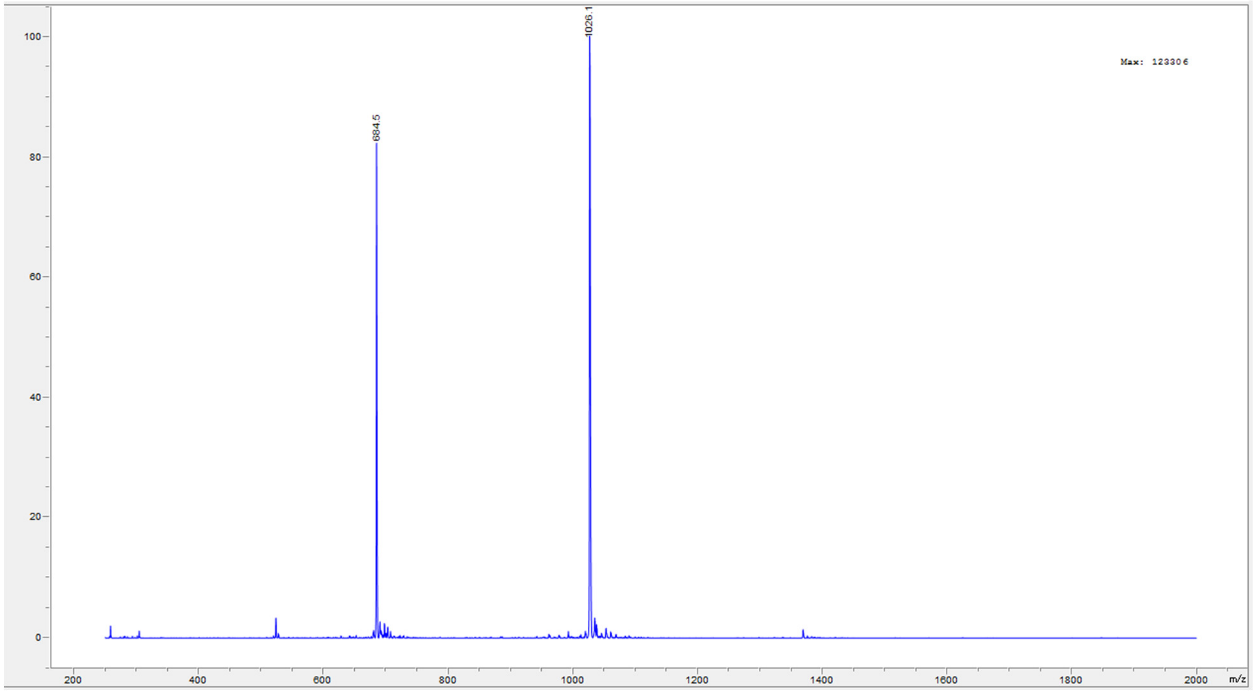

Supplementary Figure 85. LC-MS Spectra for peptide **54.**

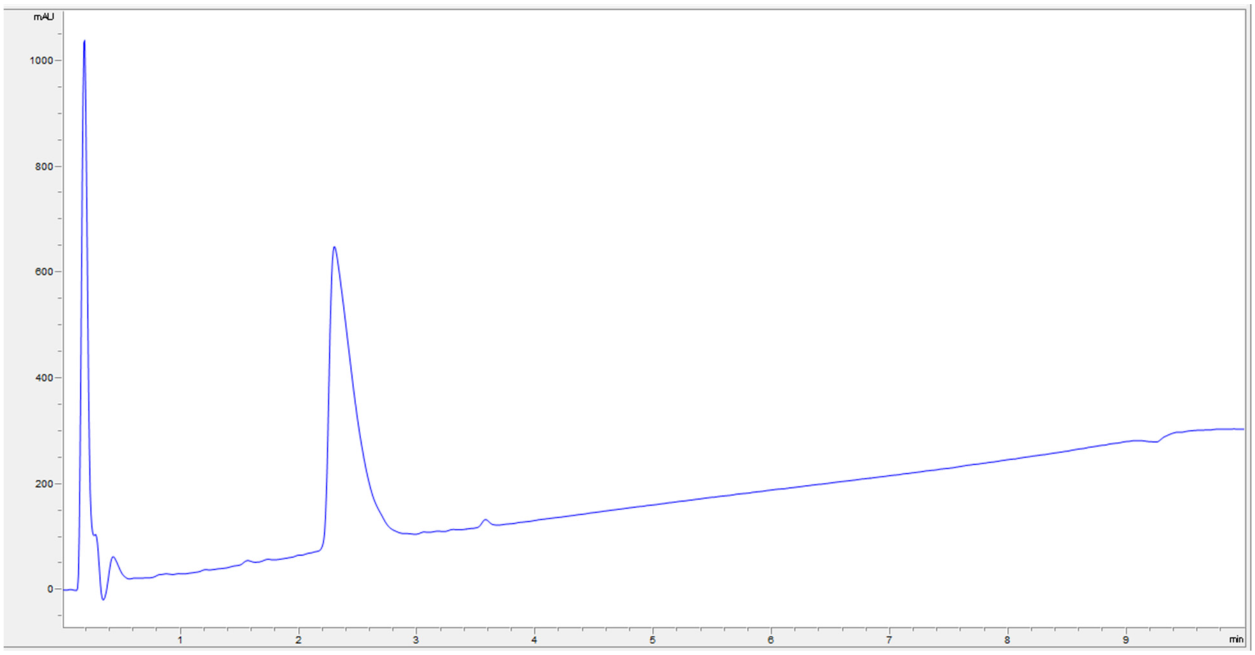

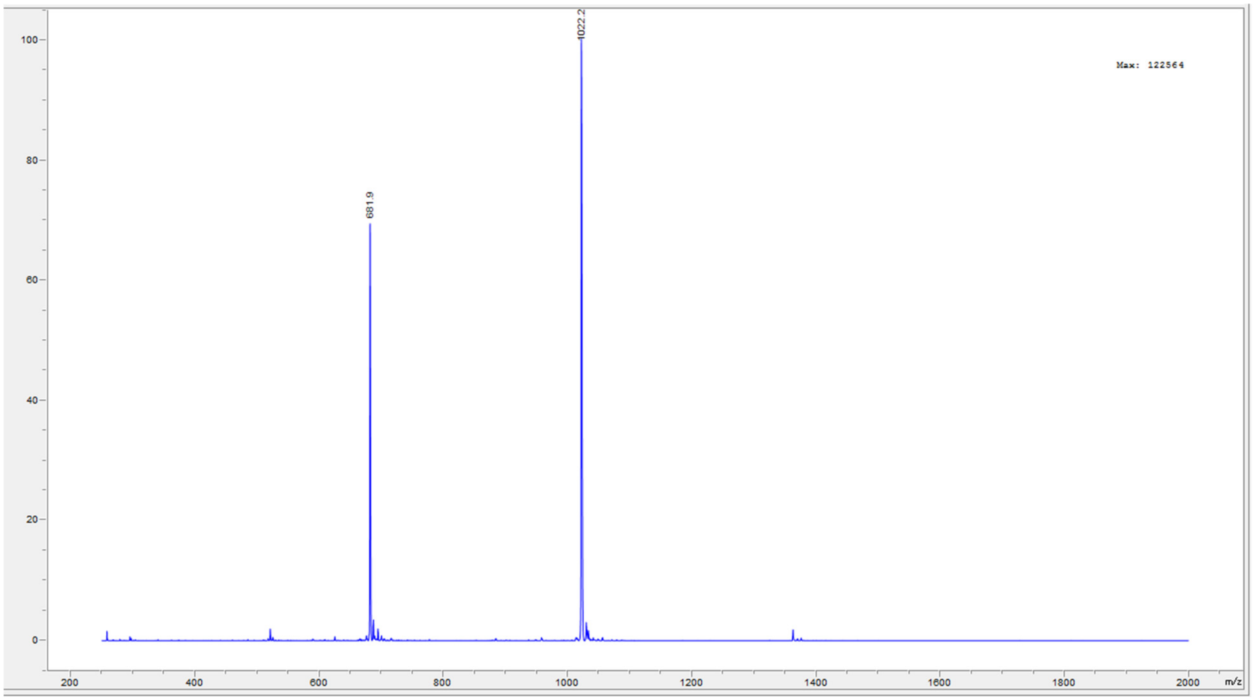

Supplementary Figure 86. LC-MS Spectra for peptide **55.**

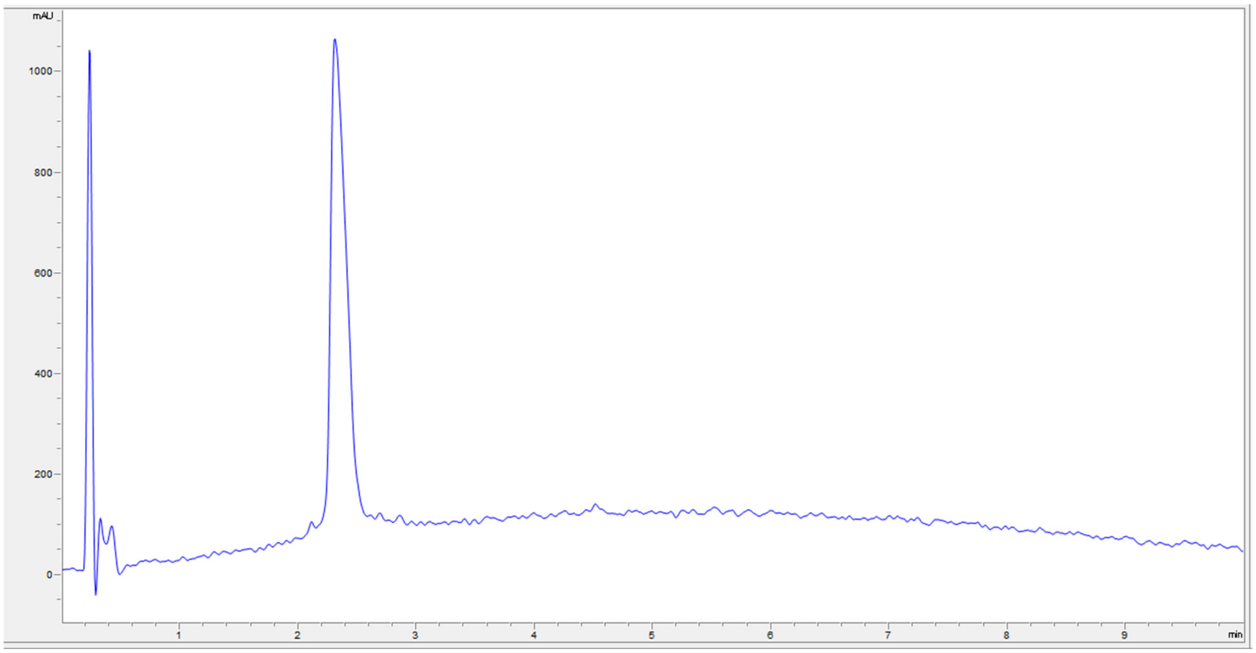

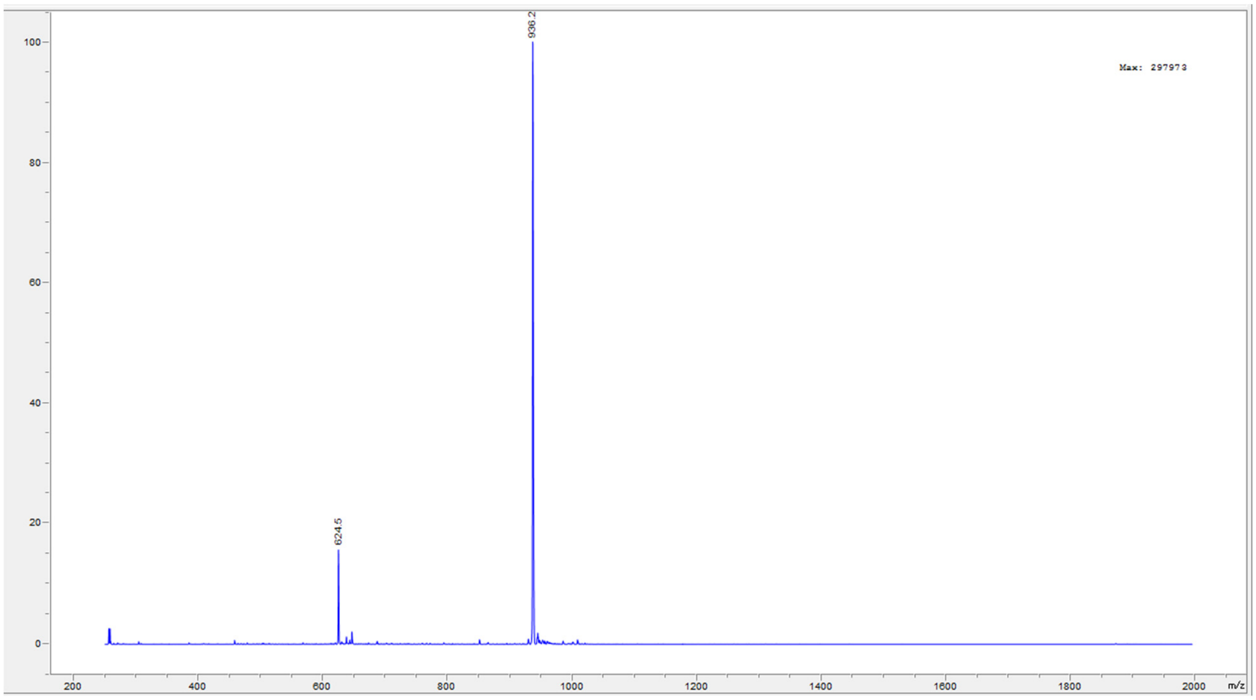

Supplementary Figure 87. LC-MS Spectra for peptide **56.**

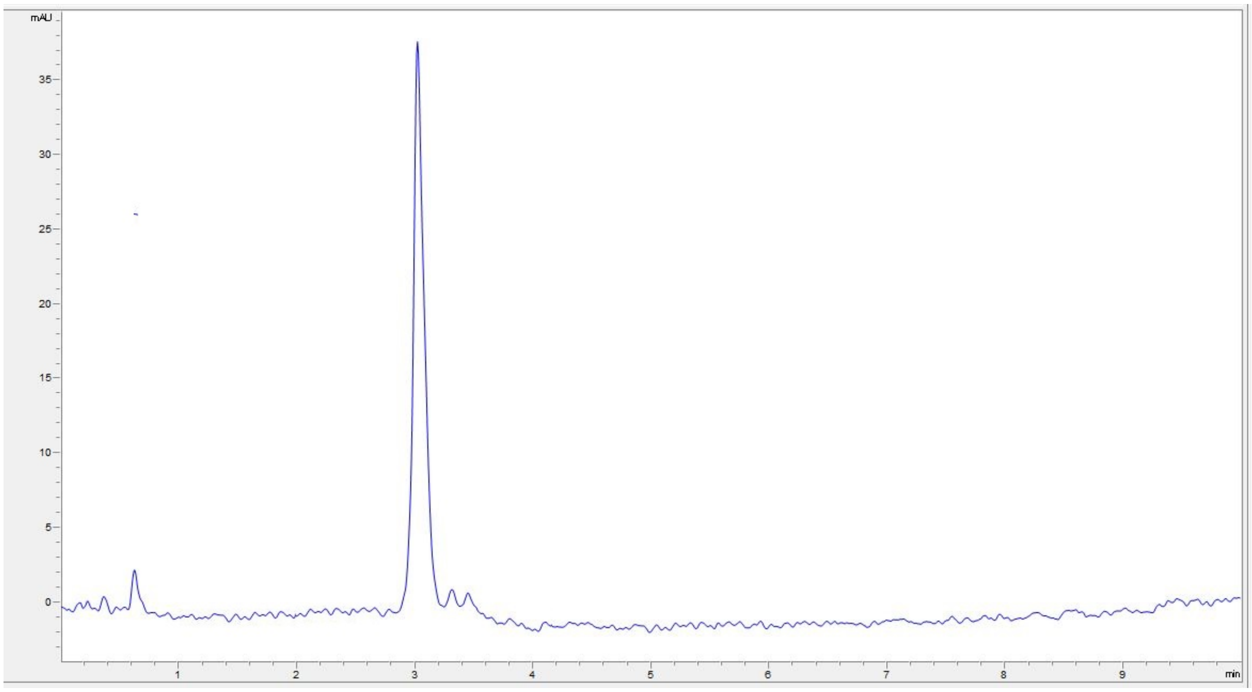

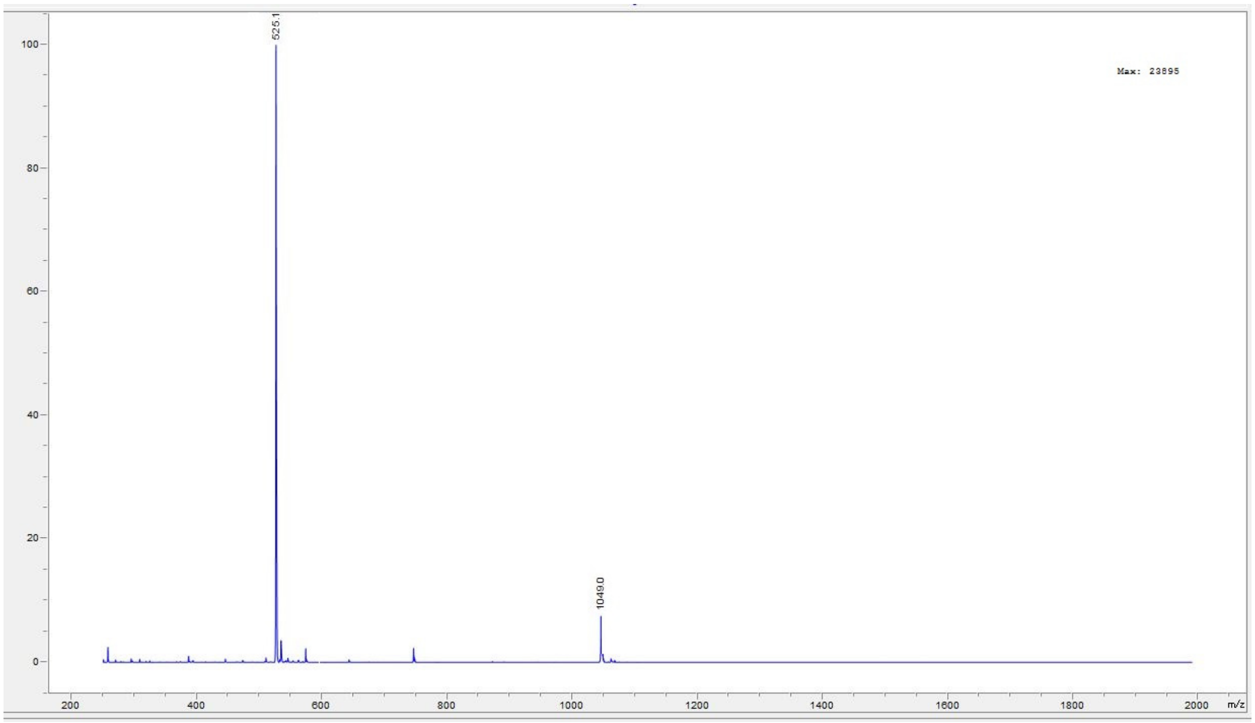

Supplementary Figure 88. LC-MS Spectra for peptide **57.**

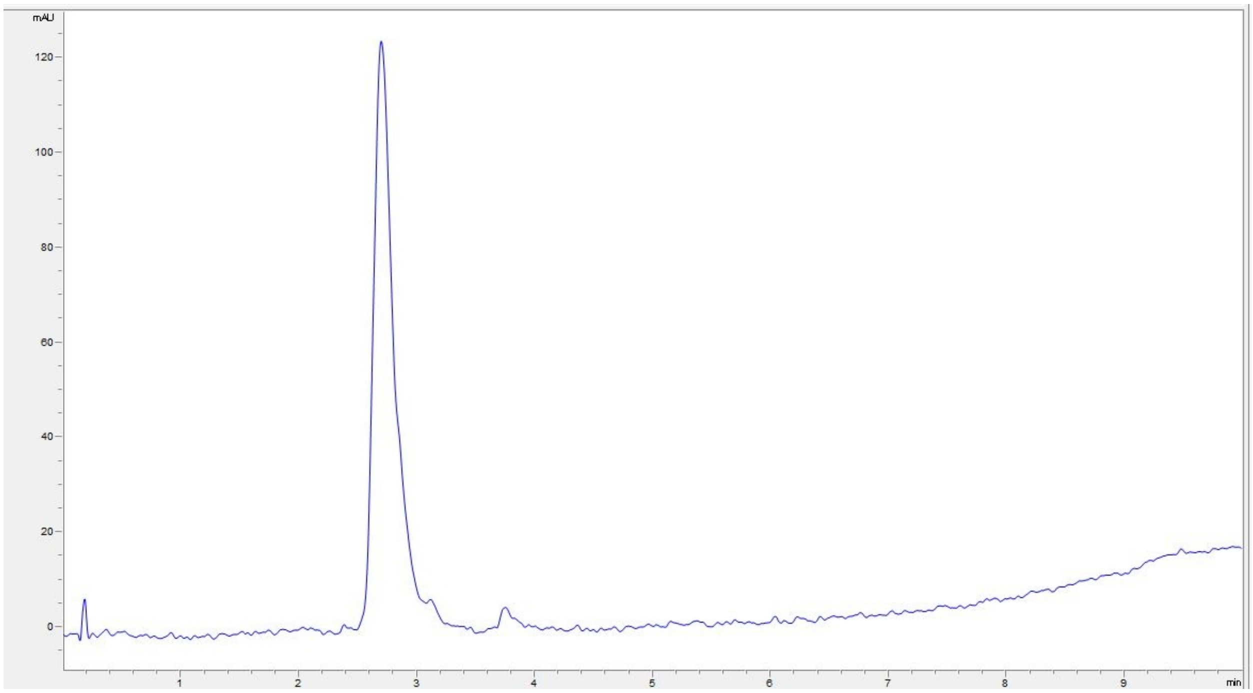

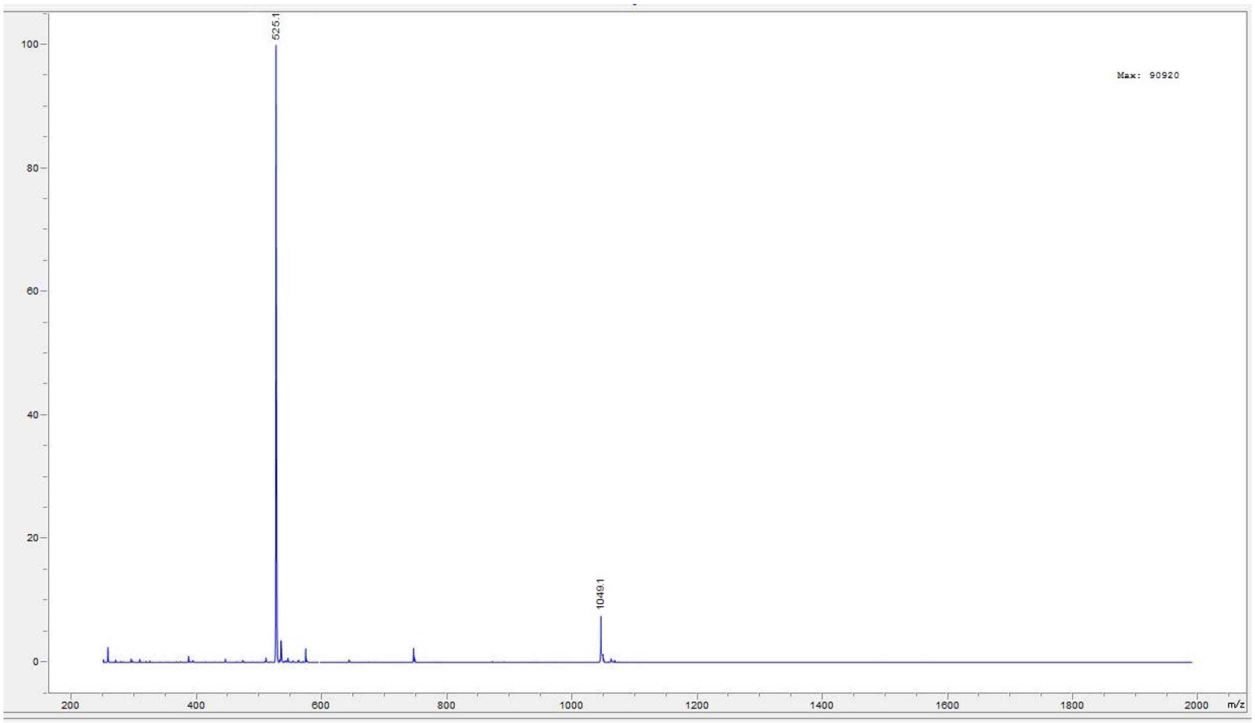

Supplementary Figure 89. LC-MS Spectra for peptide **58.**

Supplementary Figure 90. LC-MS Spectra for peptide 59.

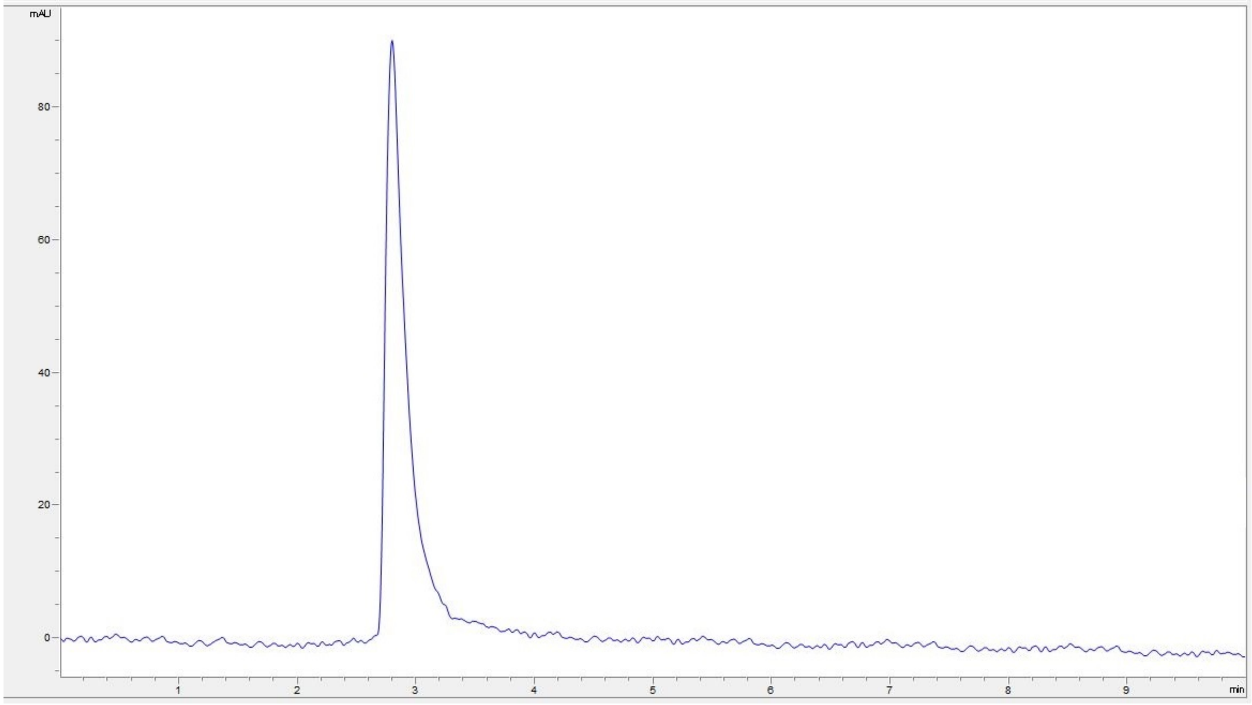

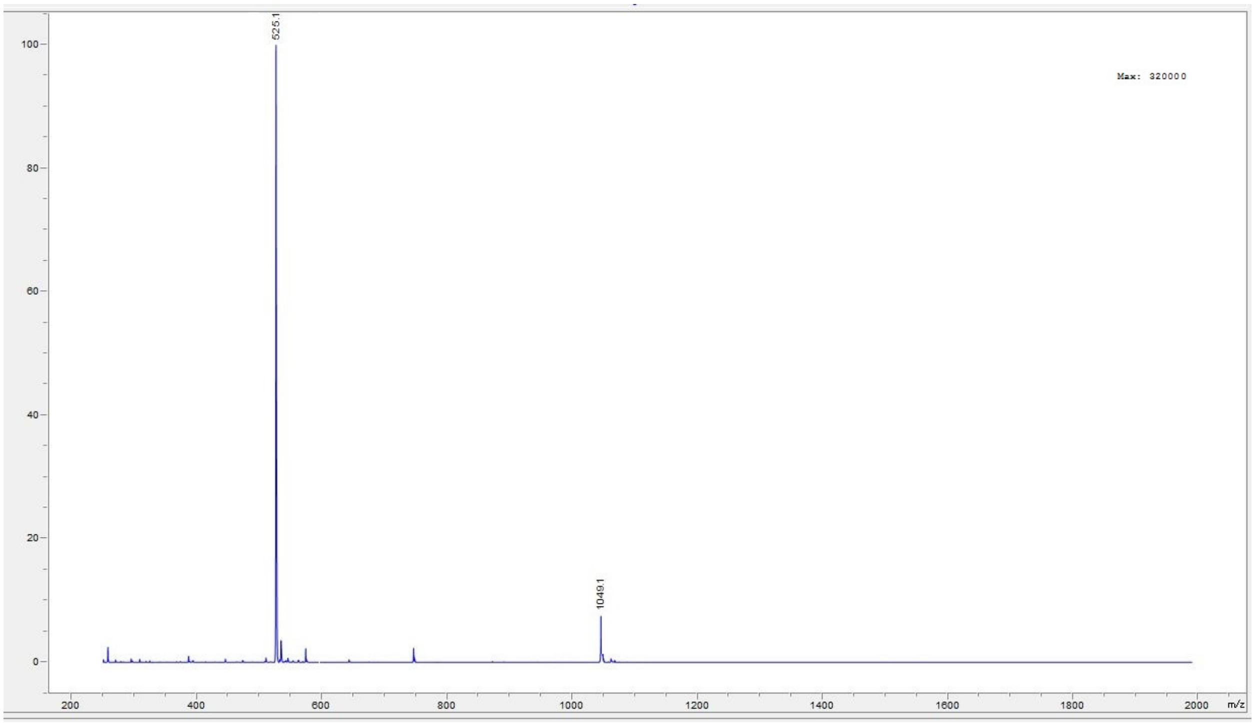

Supplementary Figure 90. LC-MS Spectra for peptide **59.**

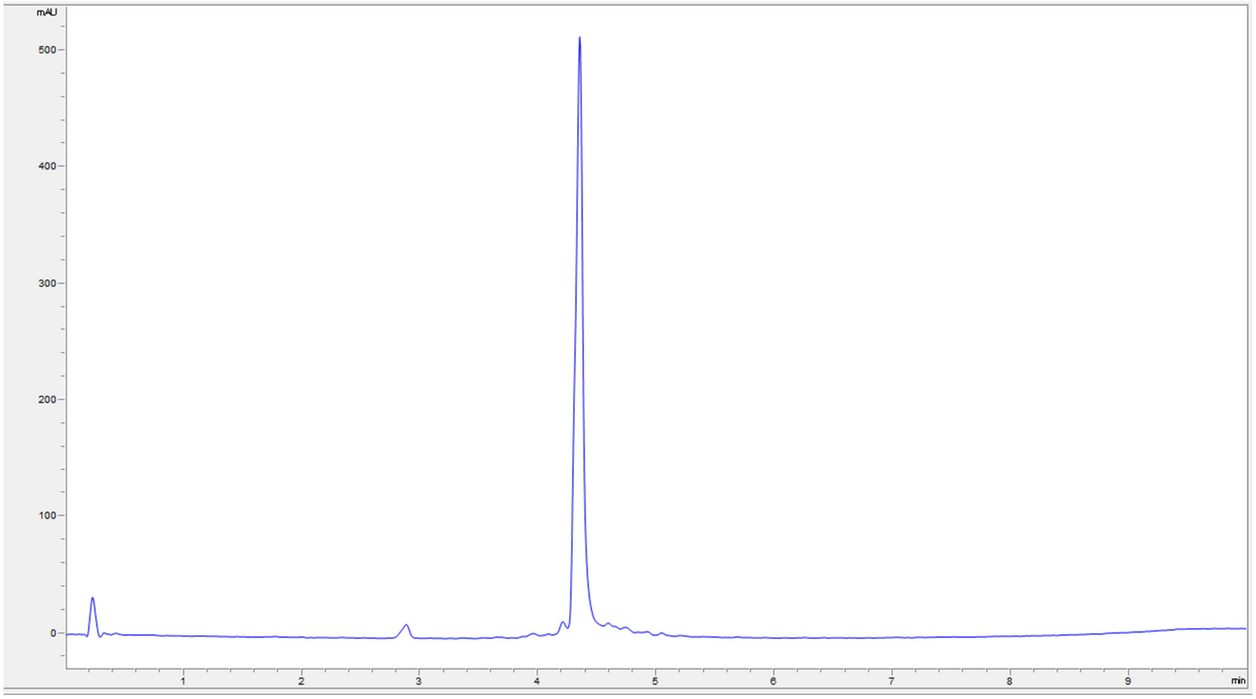

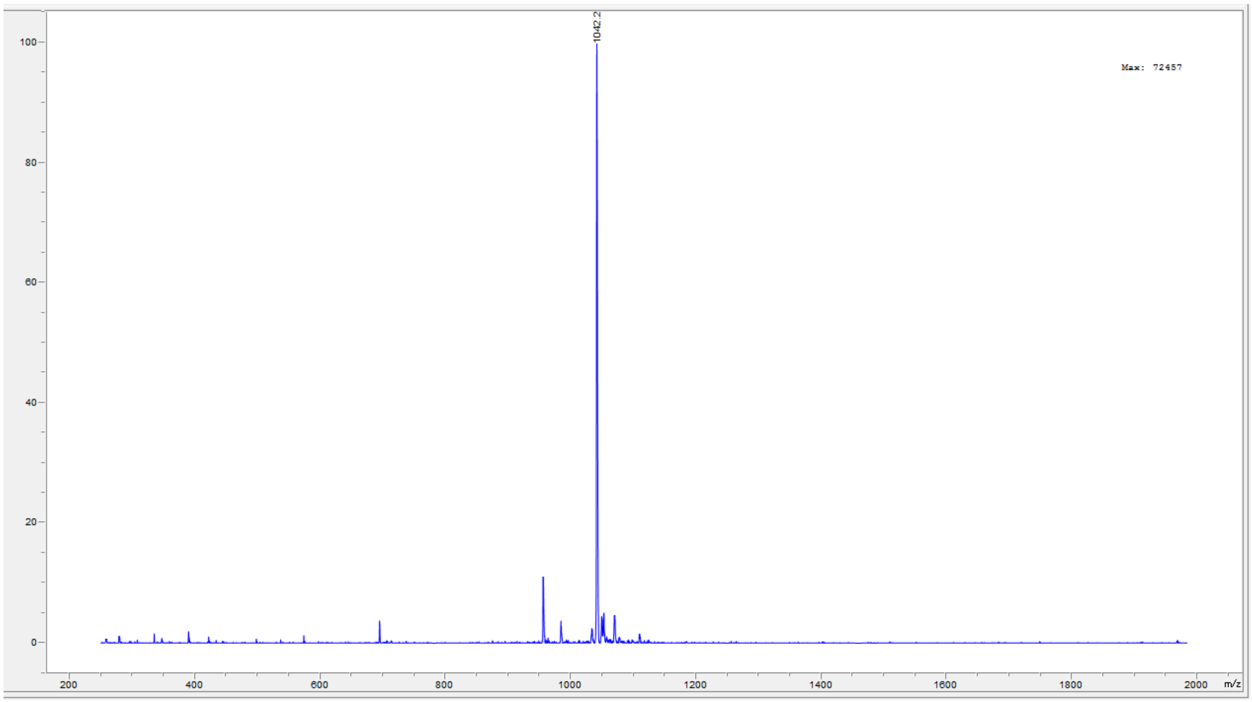

Supplementary Figure 91. LC-MS Spectra for peptide **60.**

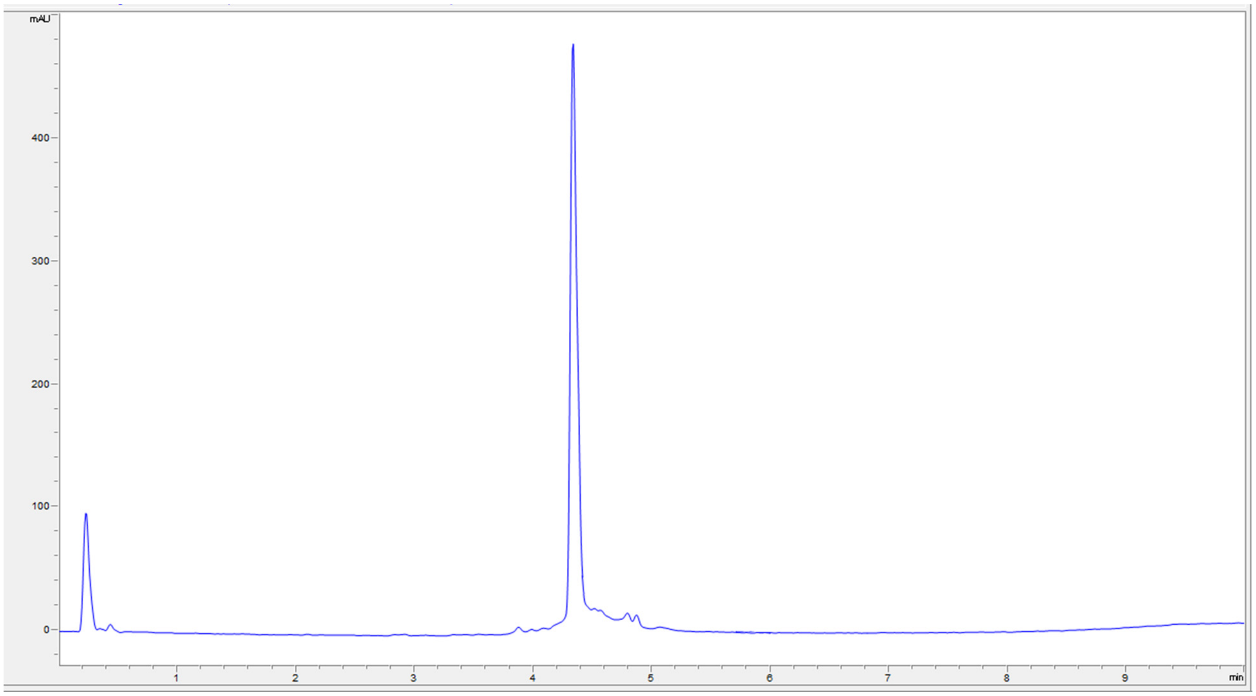

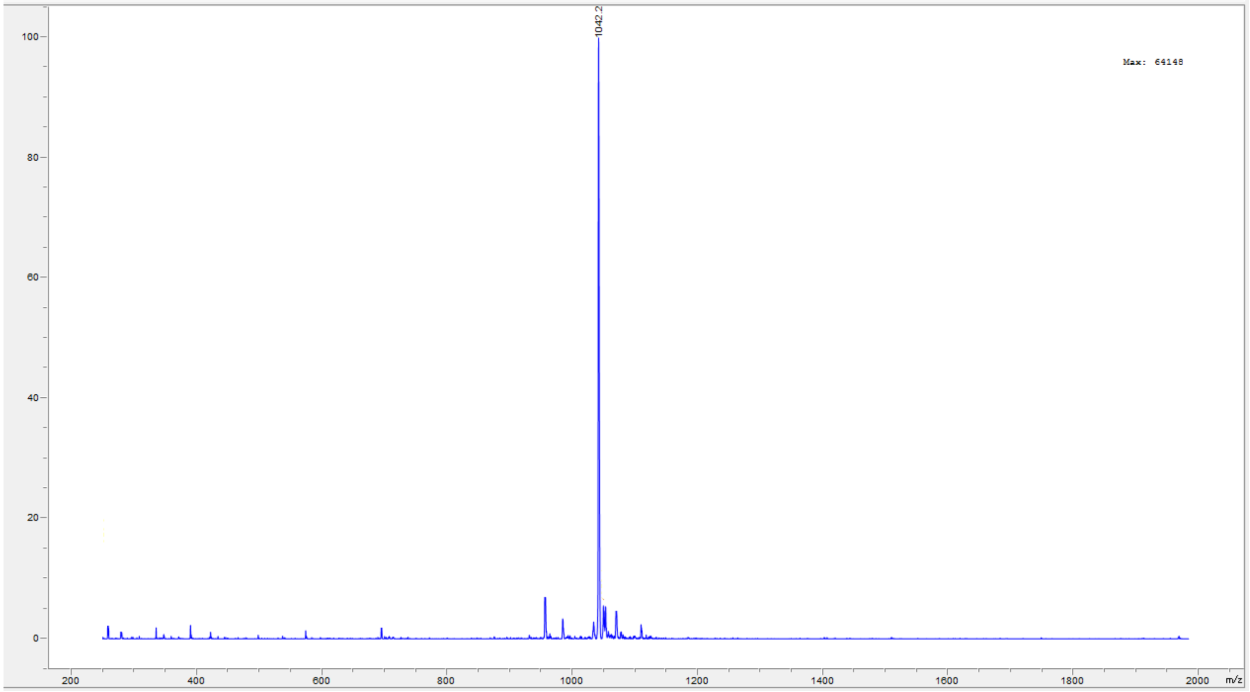

Supplementary Figure 92. LC-MS Spectra for peptide **61.**

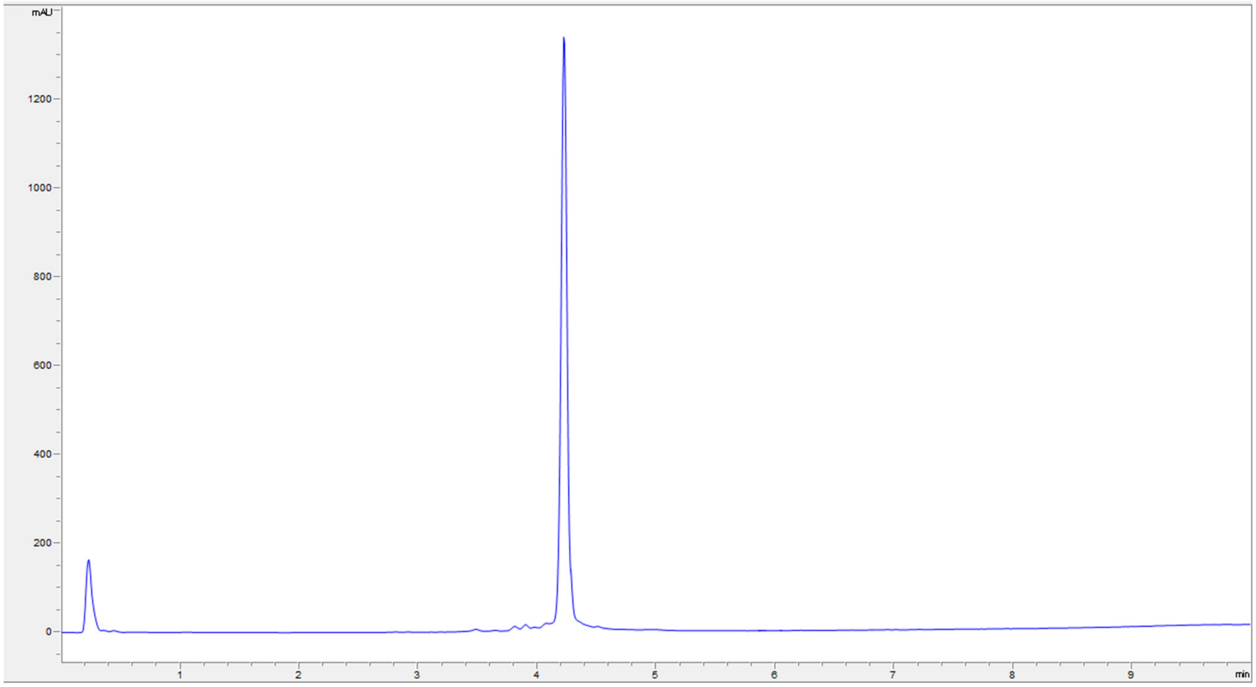

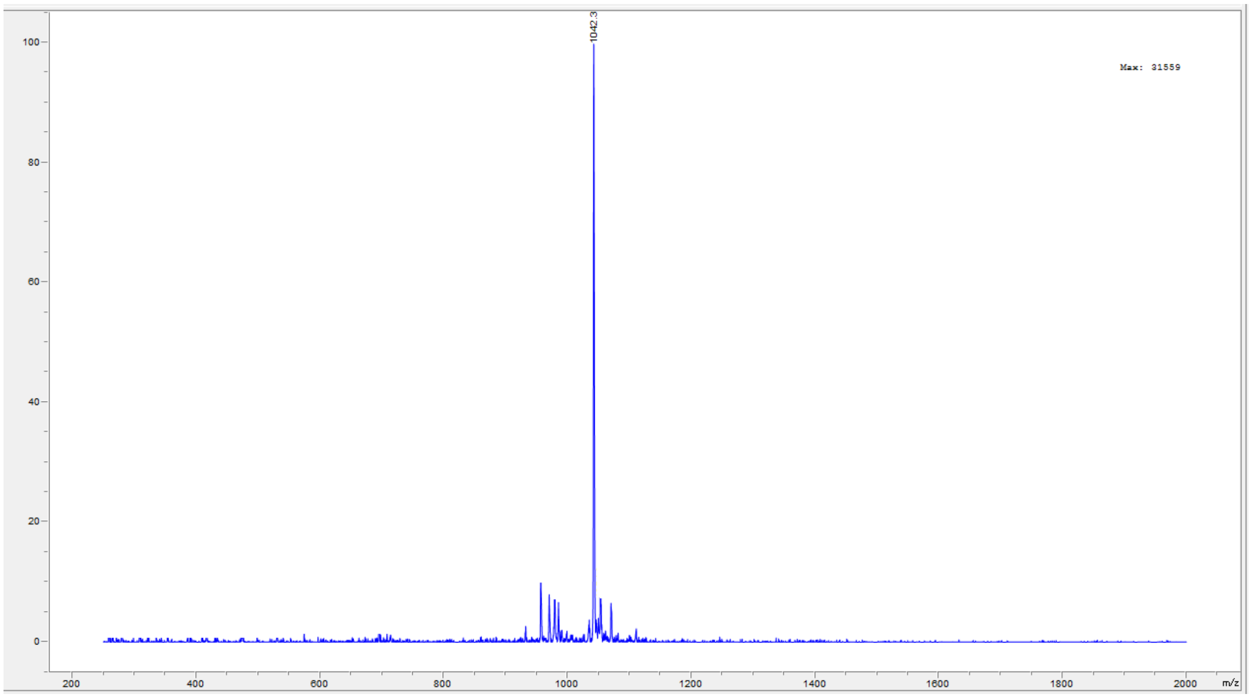

Supplementary Figure 93. LC-MS Spectra for peptide **62.**

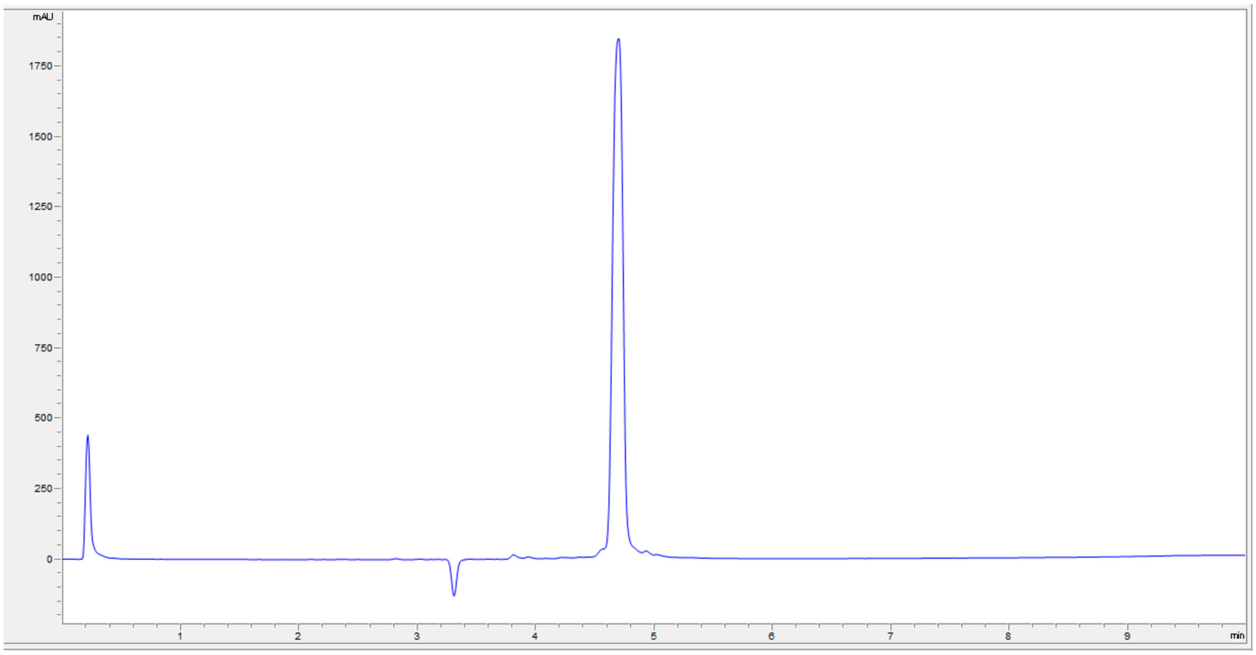

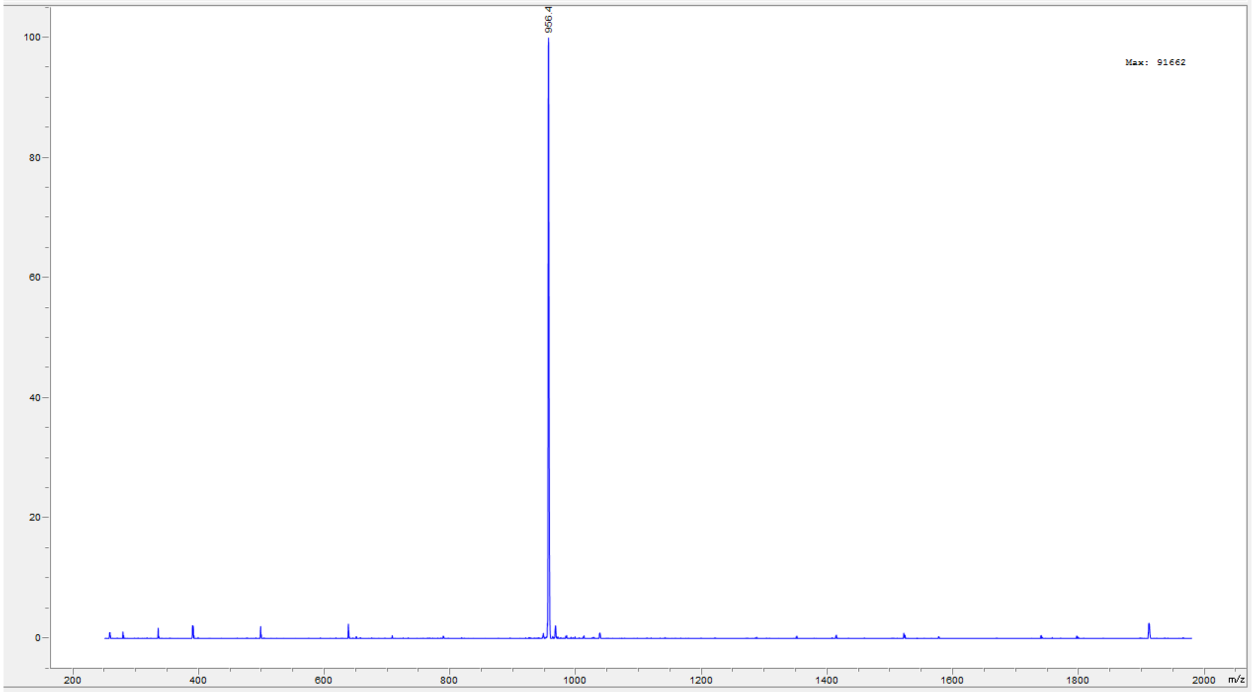

Supplementary Figure 94. LC-MS Spectra for peptide **63.**

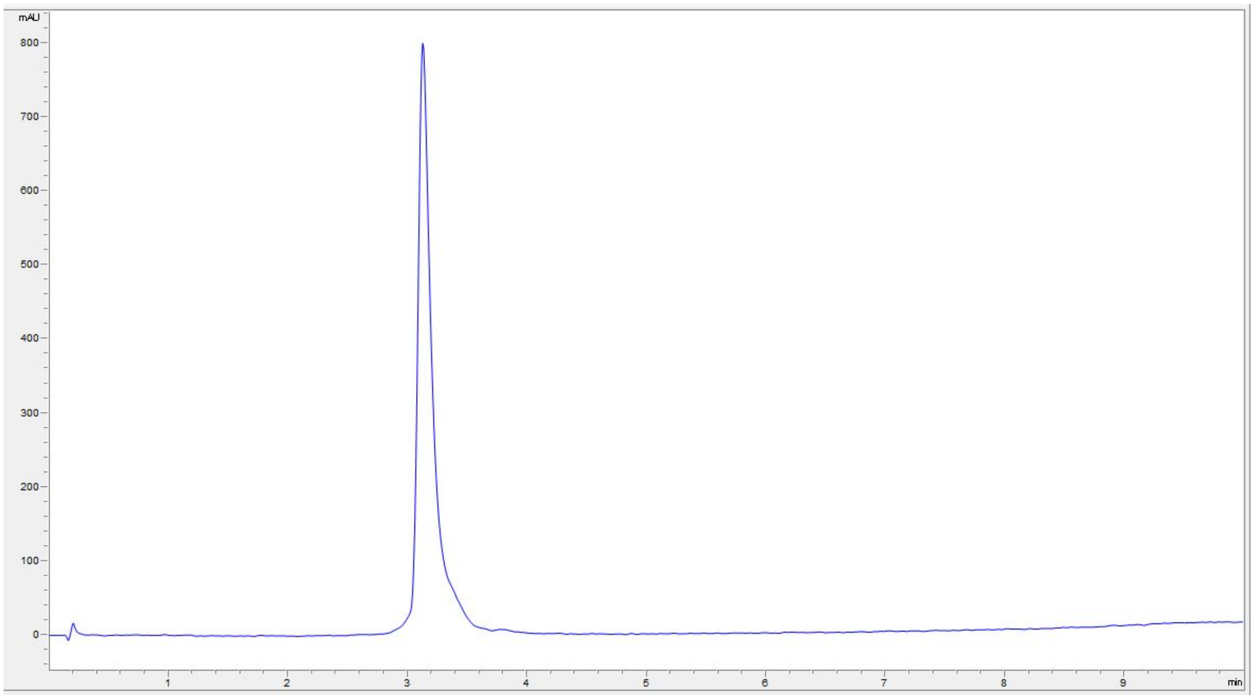

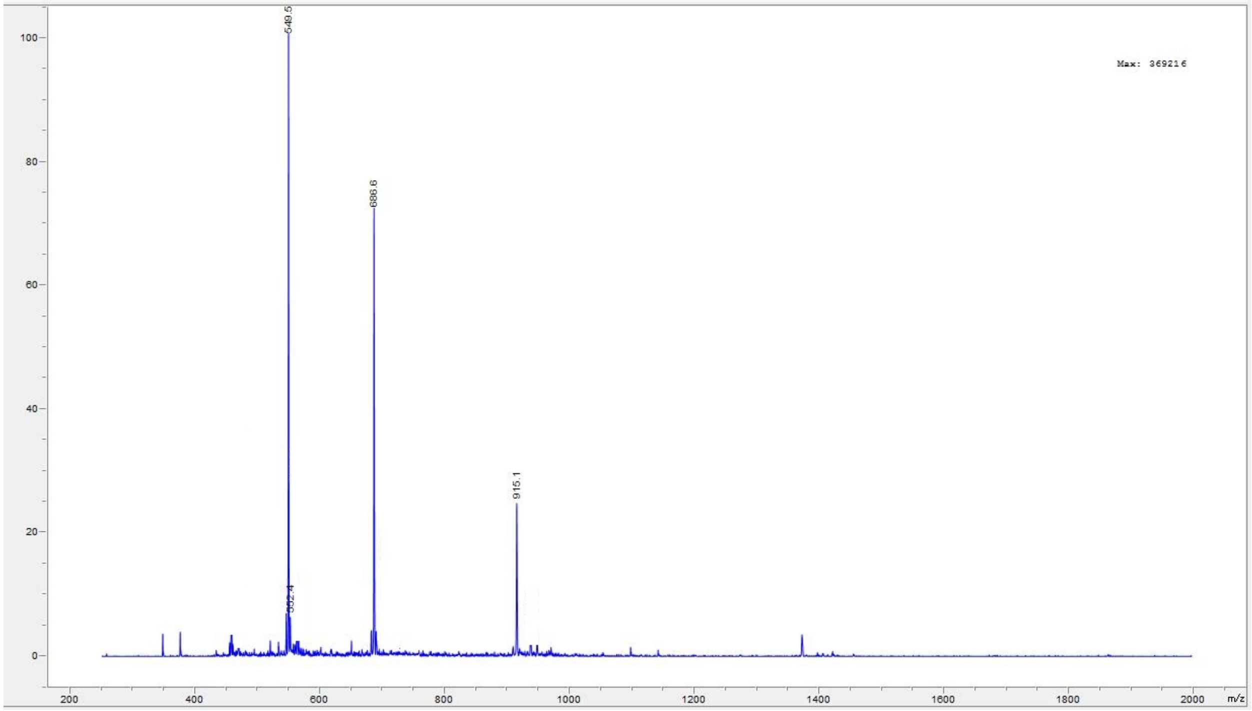

Supplementary Figure 95. LC-MS Spectra for peptide **64.**

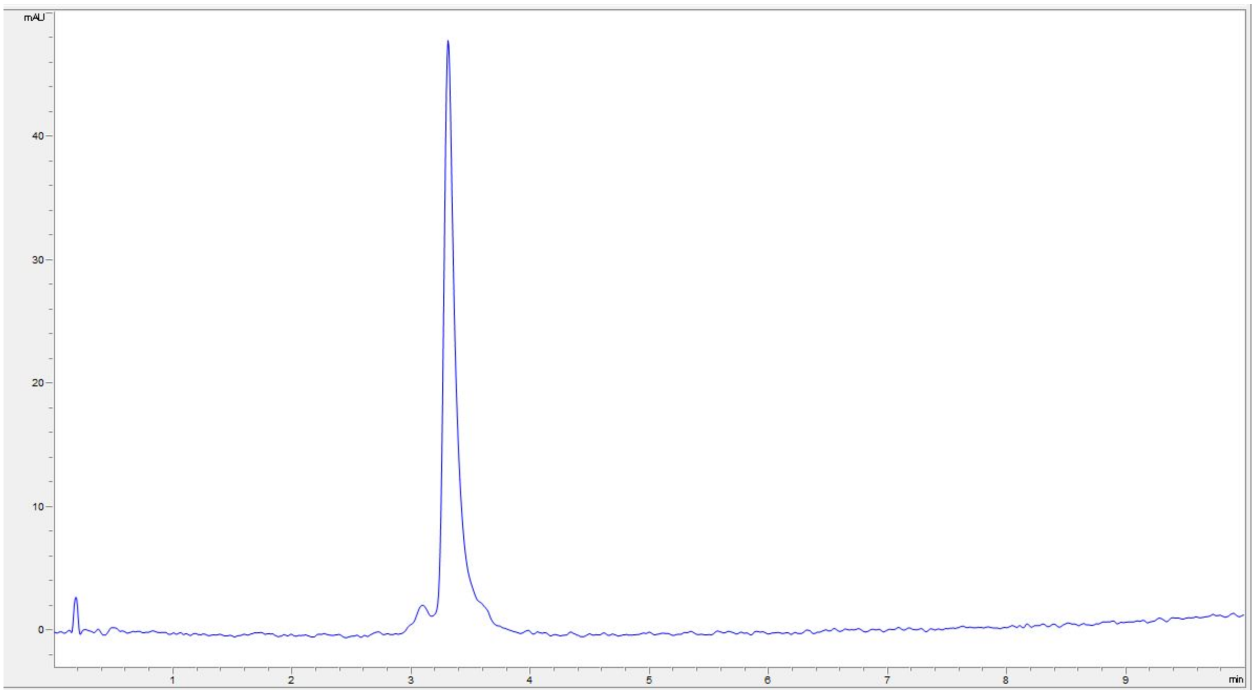

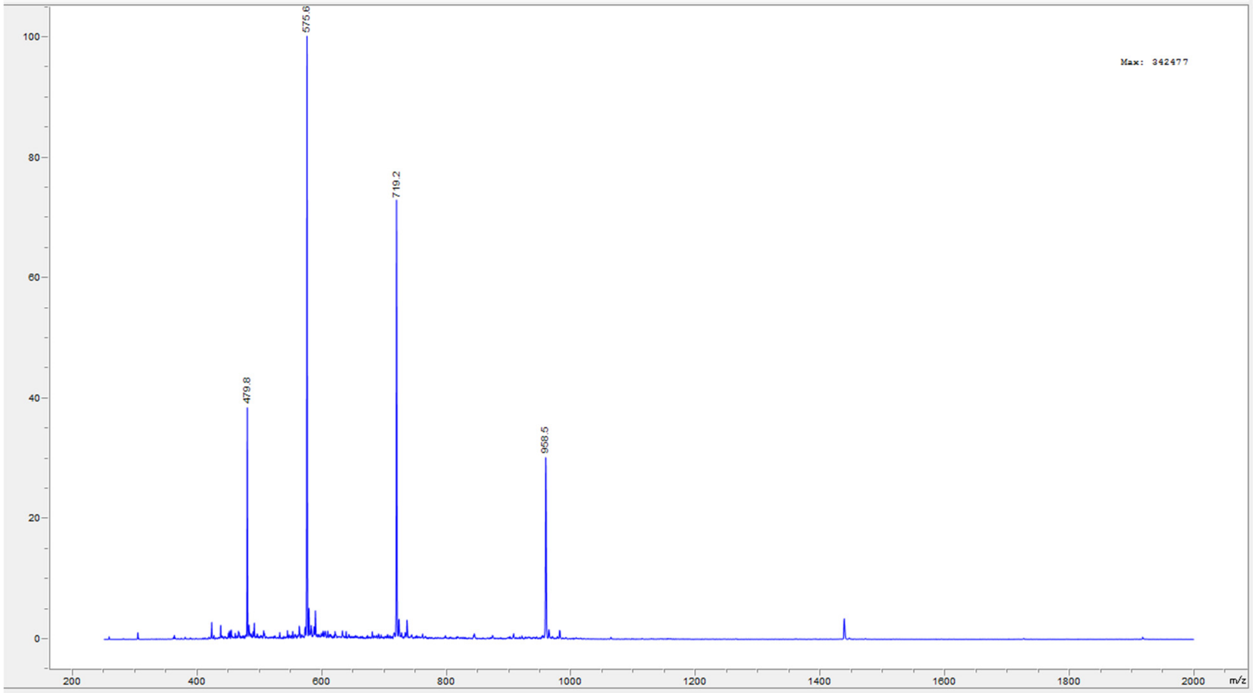

Supplementary Figure 96. LC-MS Spectra for peptide **65.**

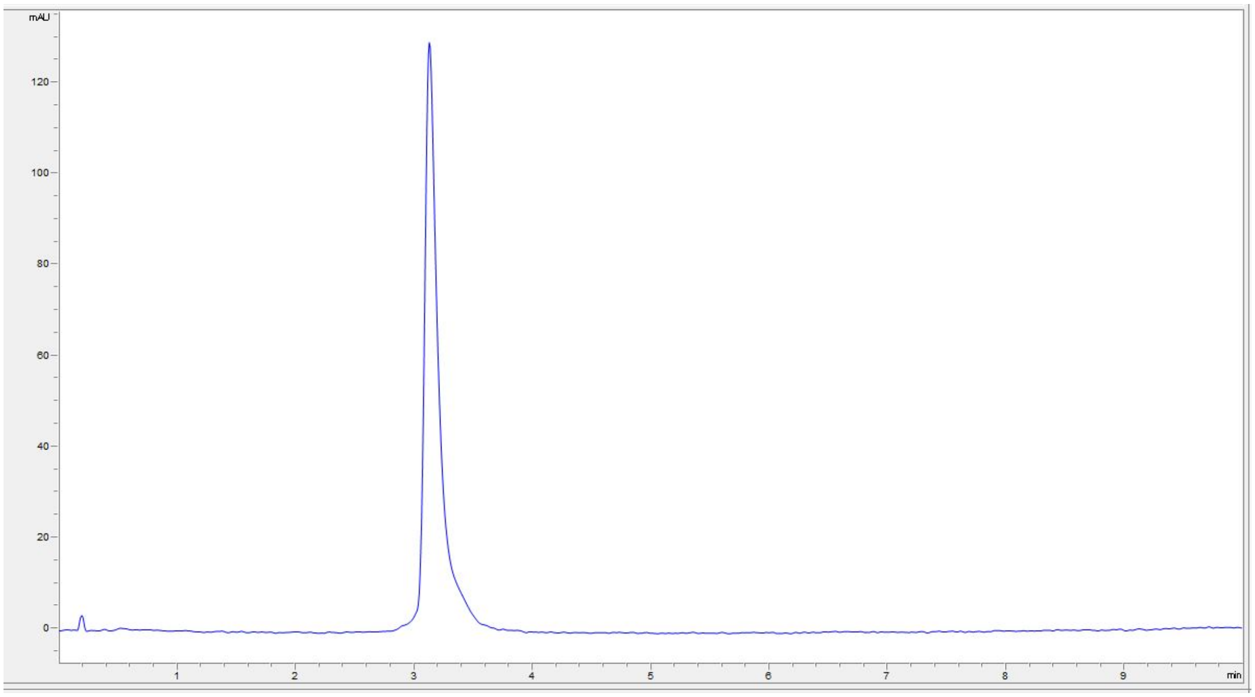

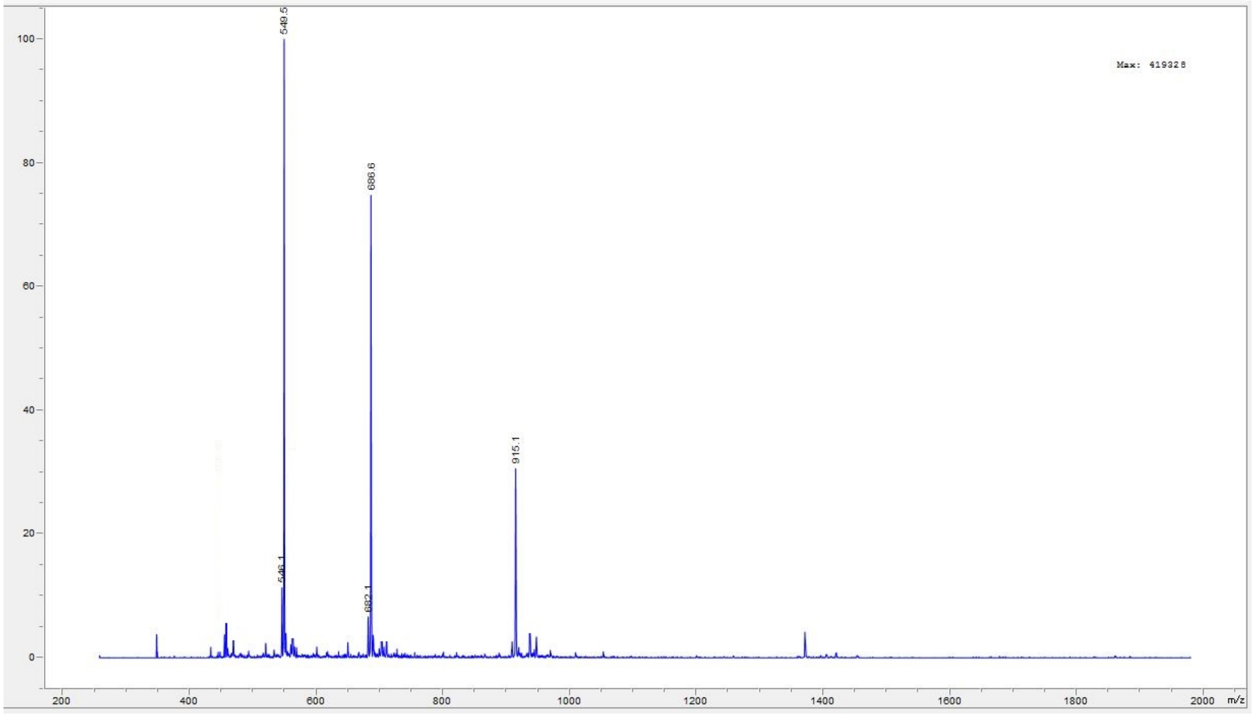

Supplementary Figure 97. LC-MS Spectra for peptide **66.**

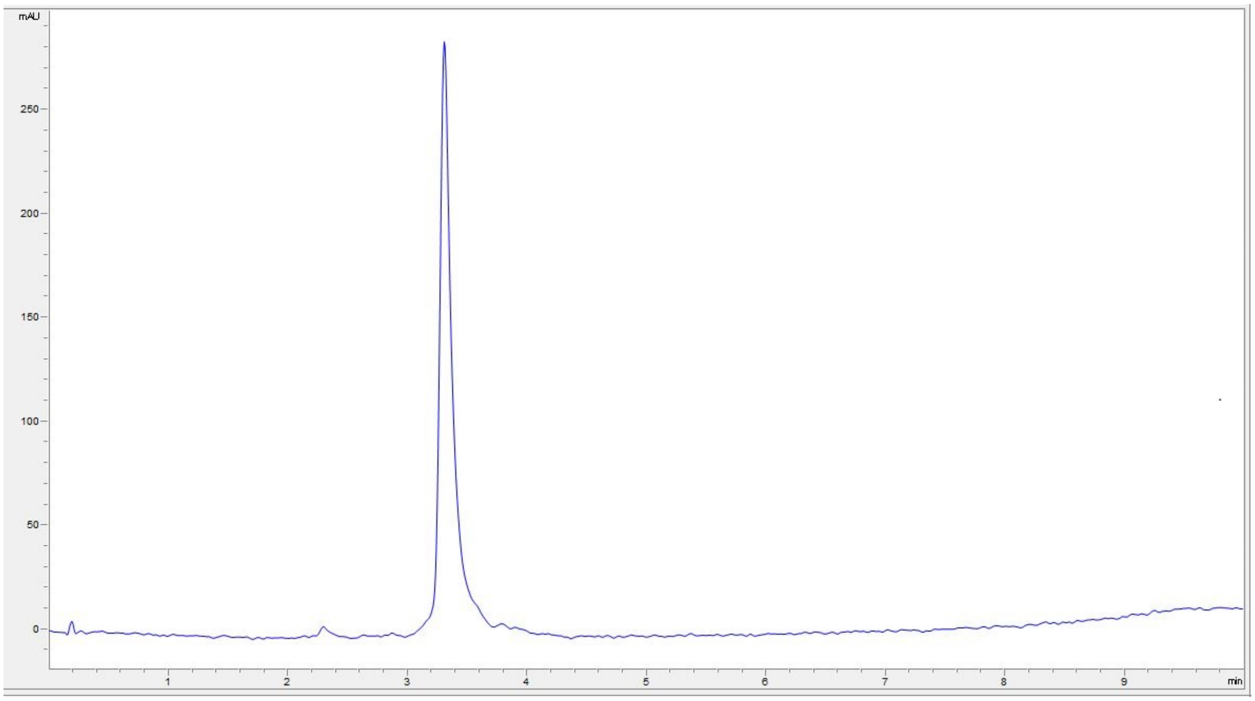

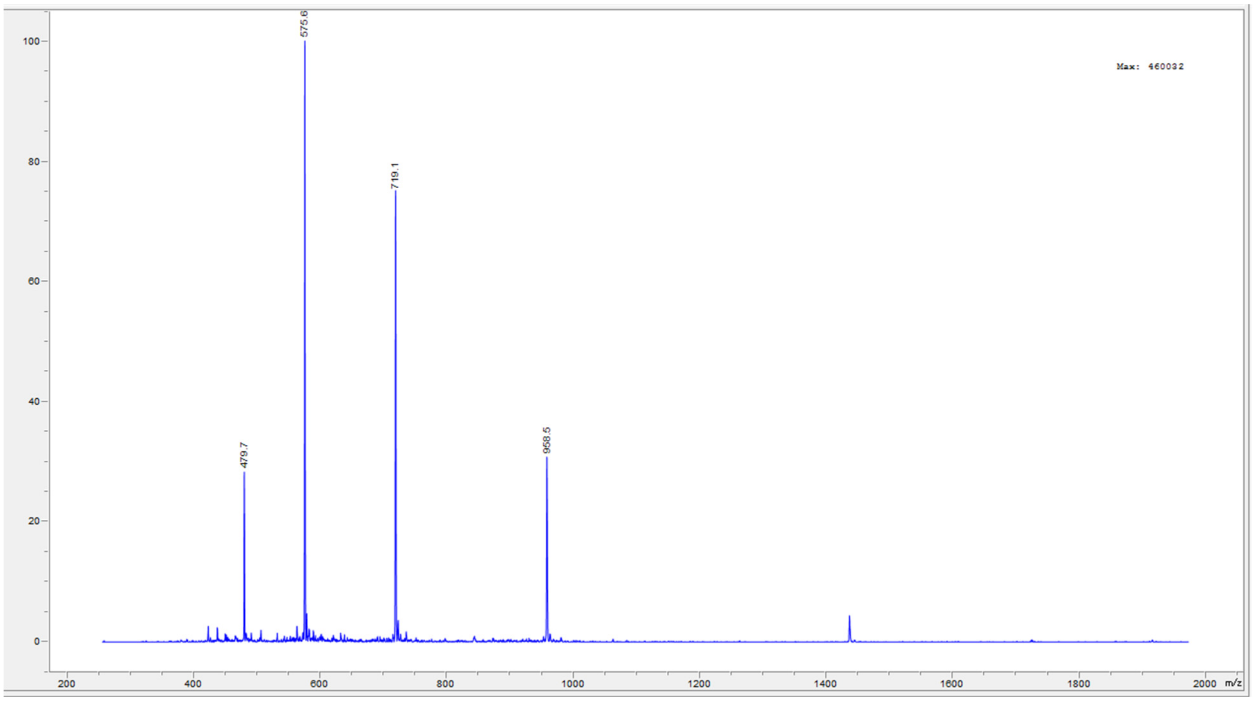

Supplementary Figure 98. LC-MS Spectra for peptide **67.**

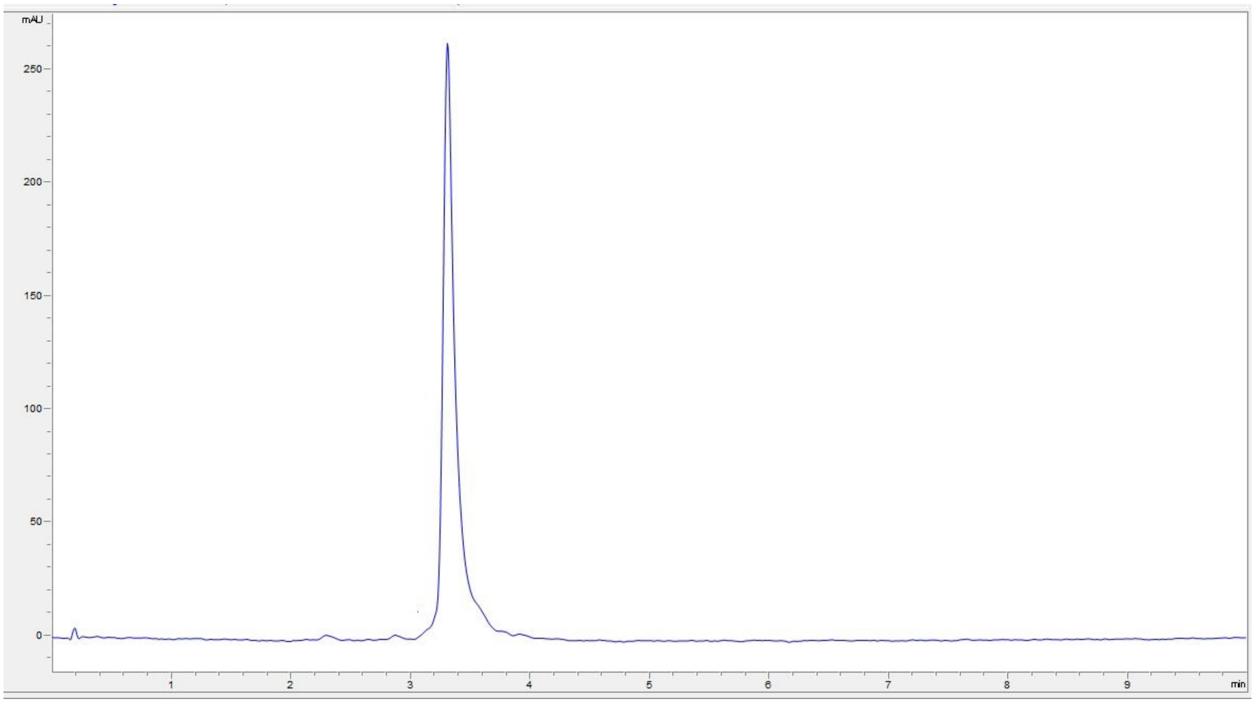

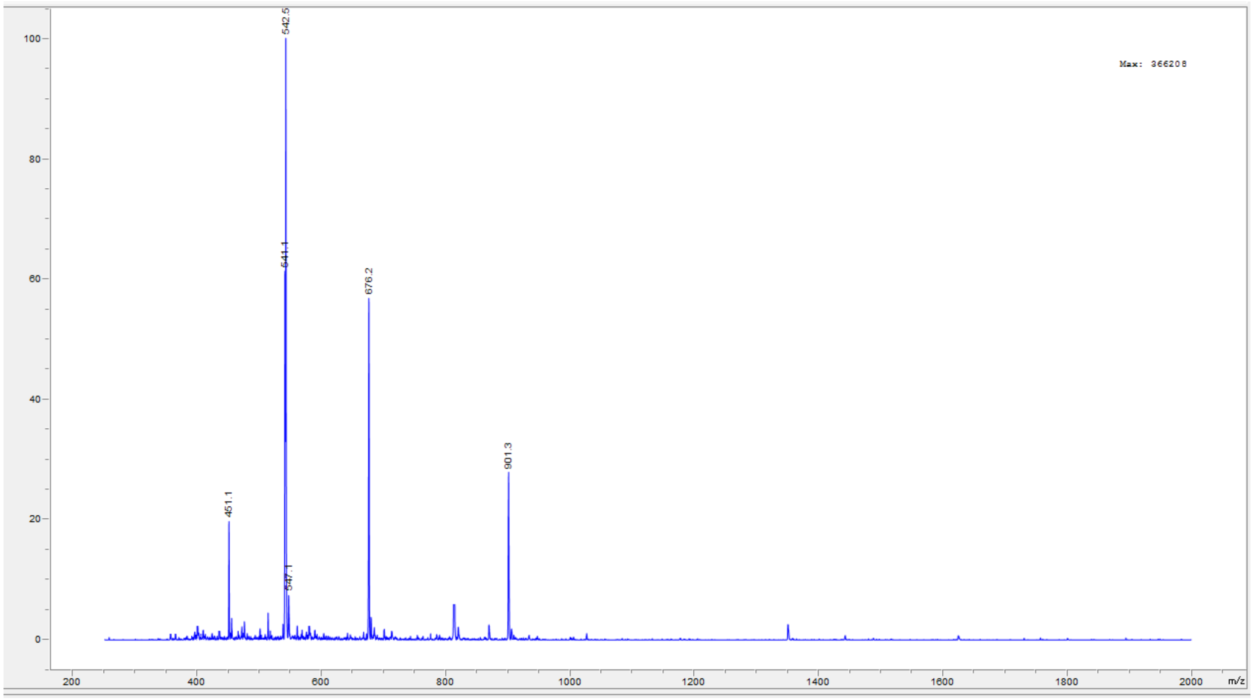

Supplementary Figure 99. LC-MS Spectra for peptide **68.**

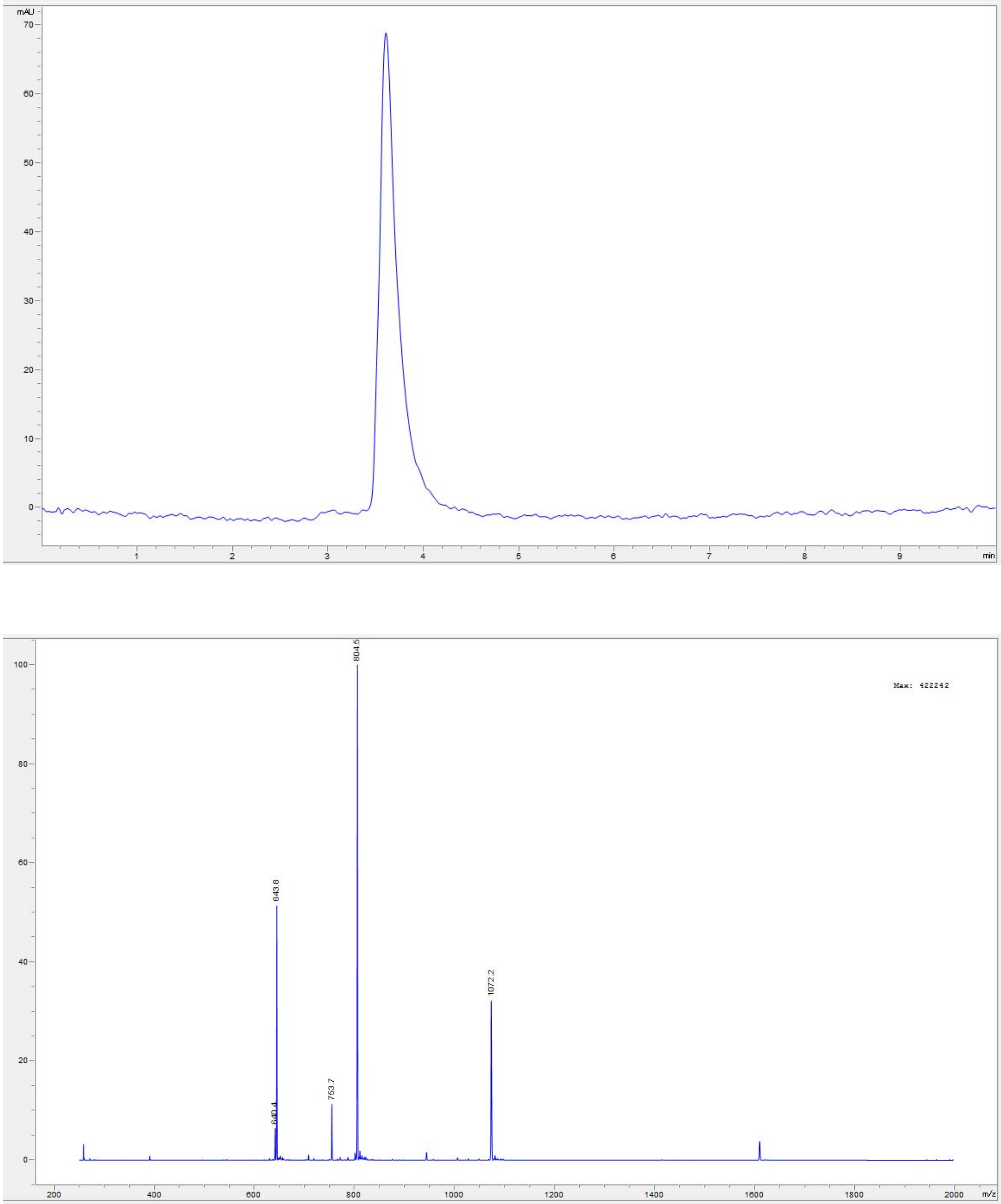

Supplementary Figure 100. LC-MS Spectra for peptide **69.**

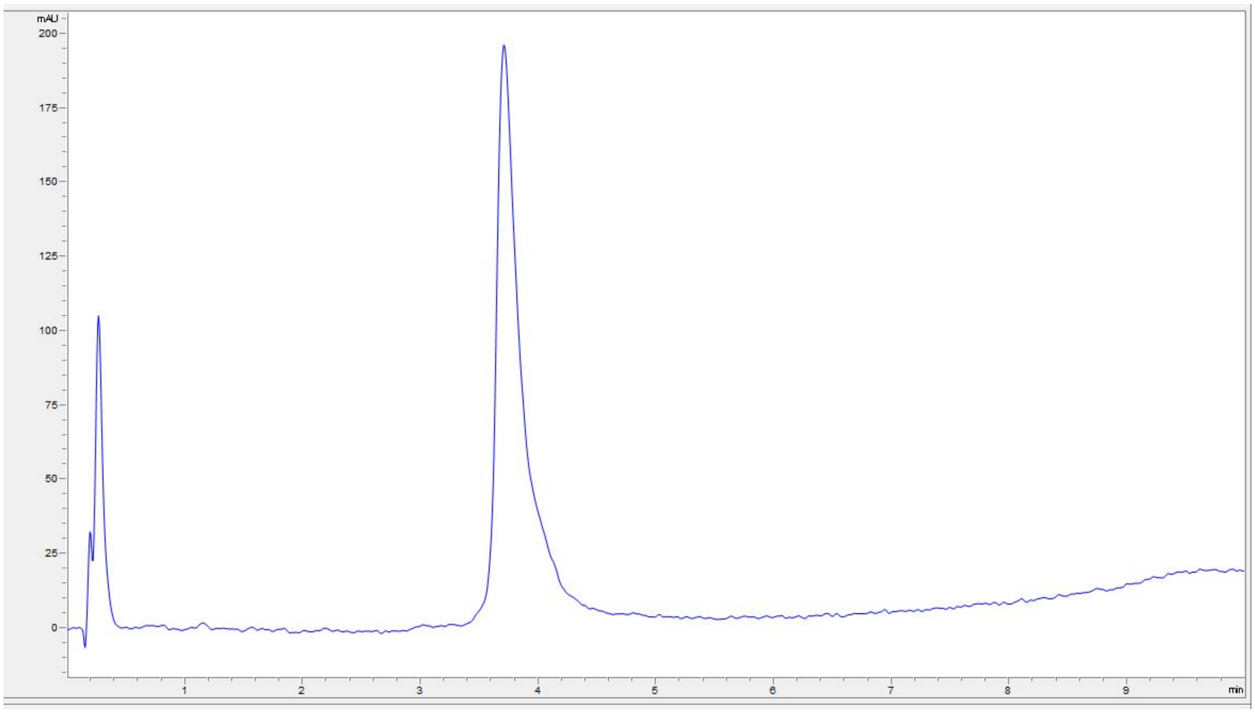

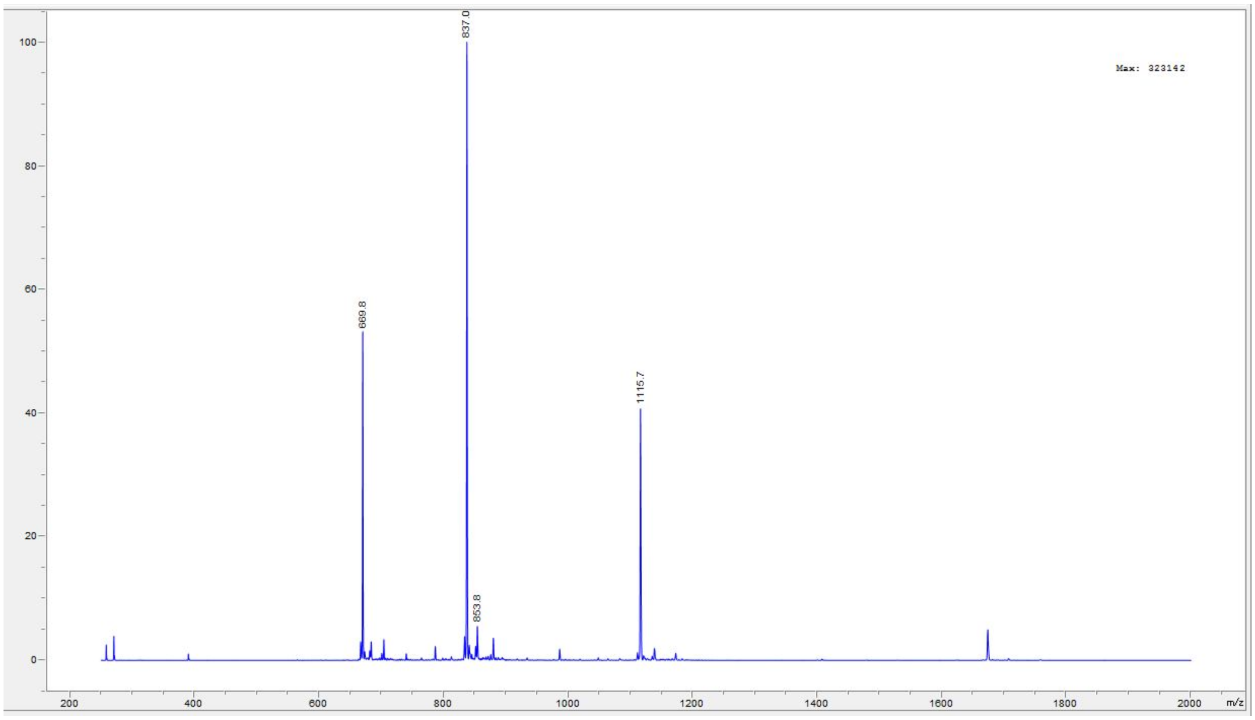

Supplementary Figure 101. LC-MS Spectra for peptide **70.**

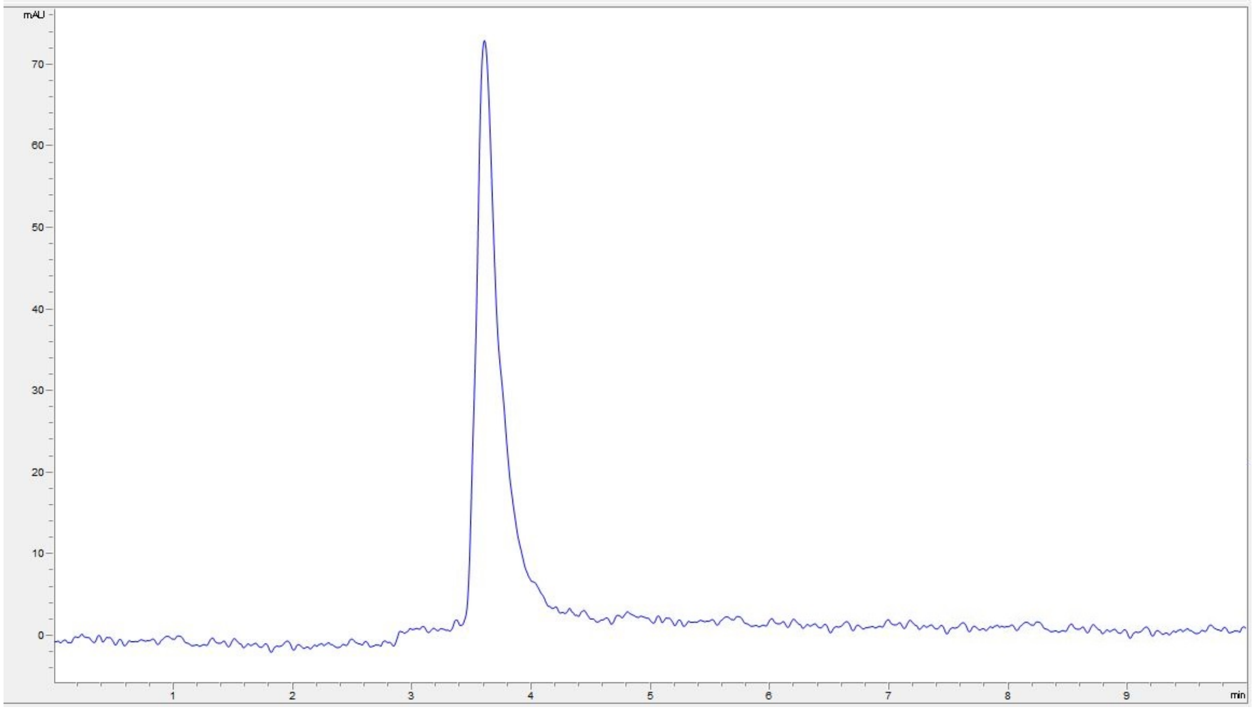

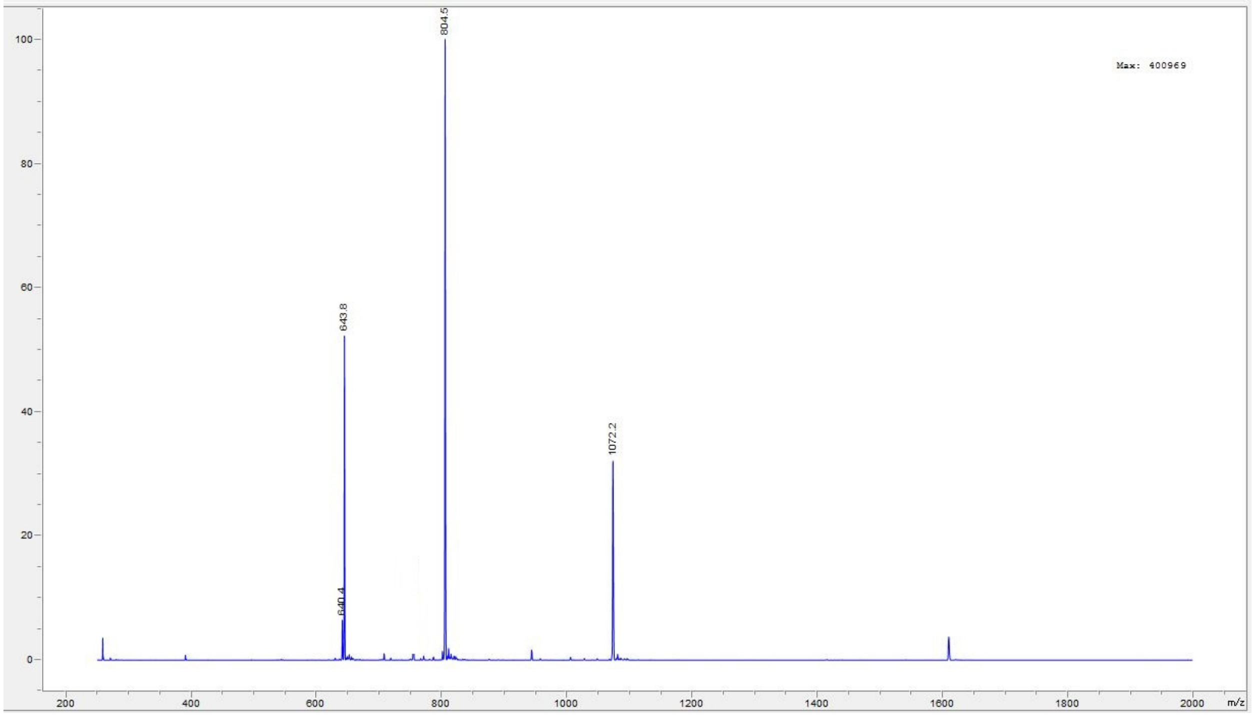

Supplementary Figure 102. LC-MS Spectra for peptide **71.**

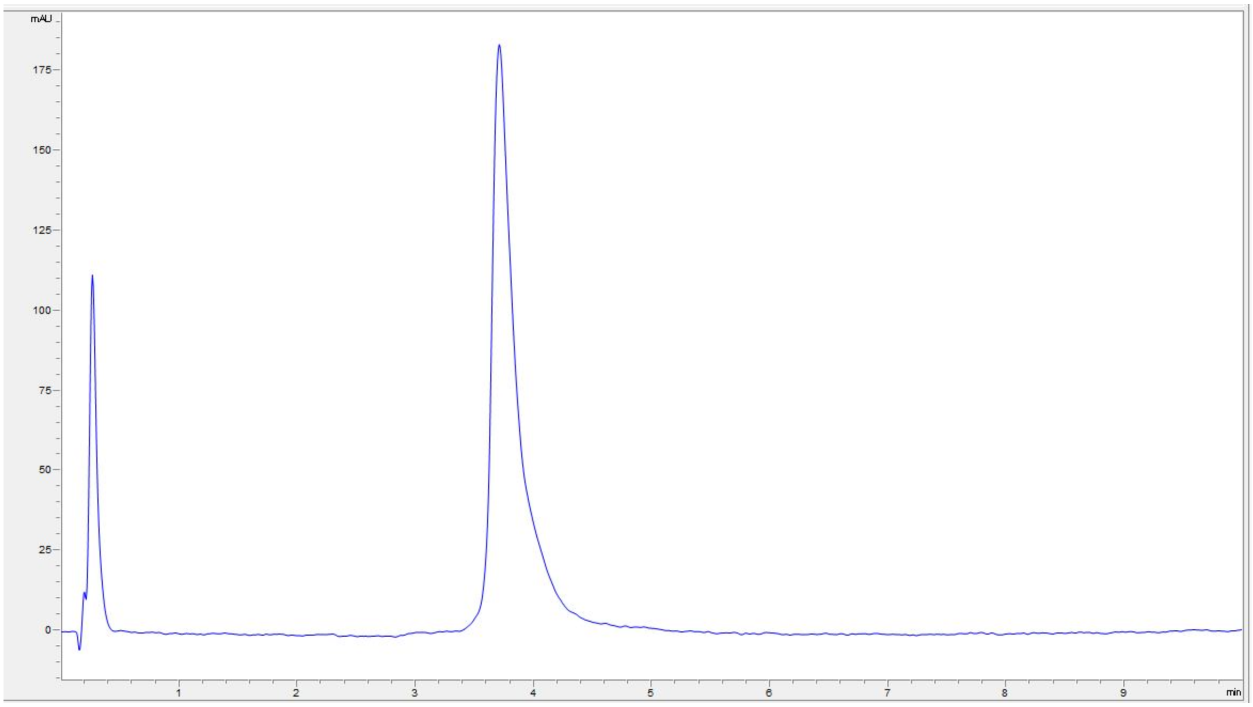

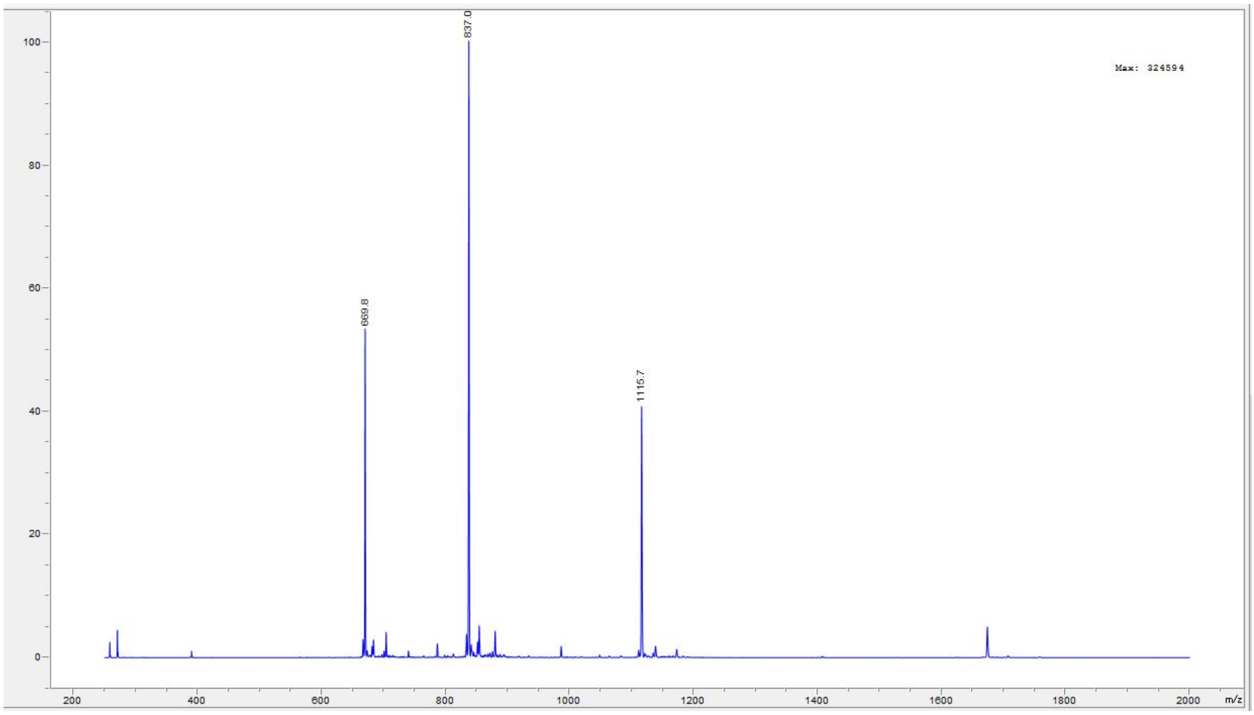

Supplementary Figure 103. LC-MS Spectra for peptide **72.**

S108

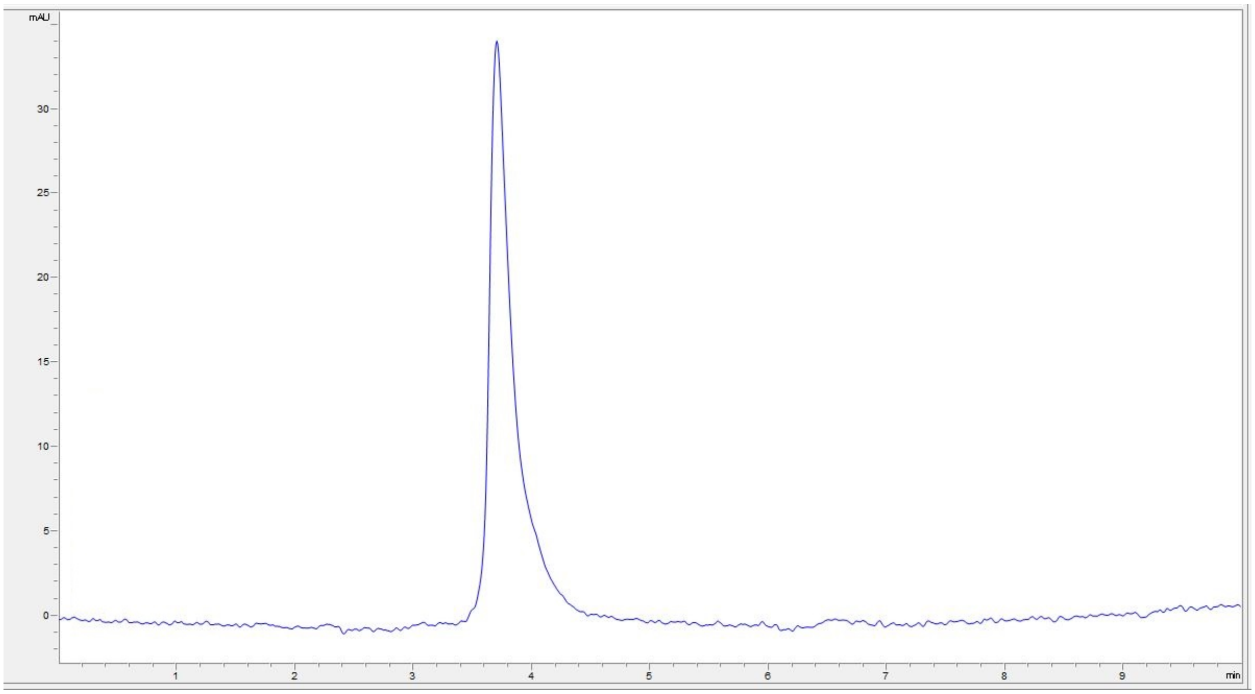

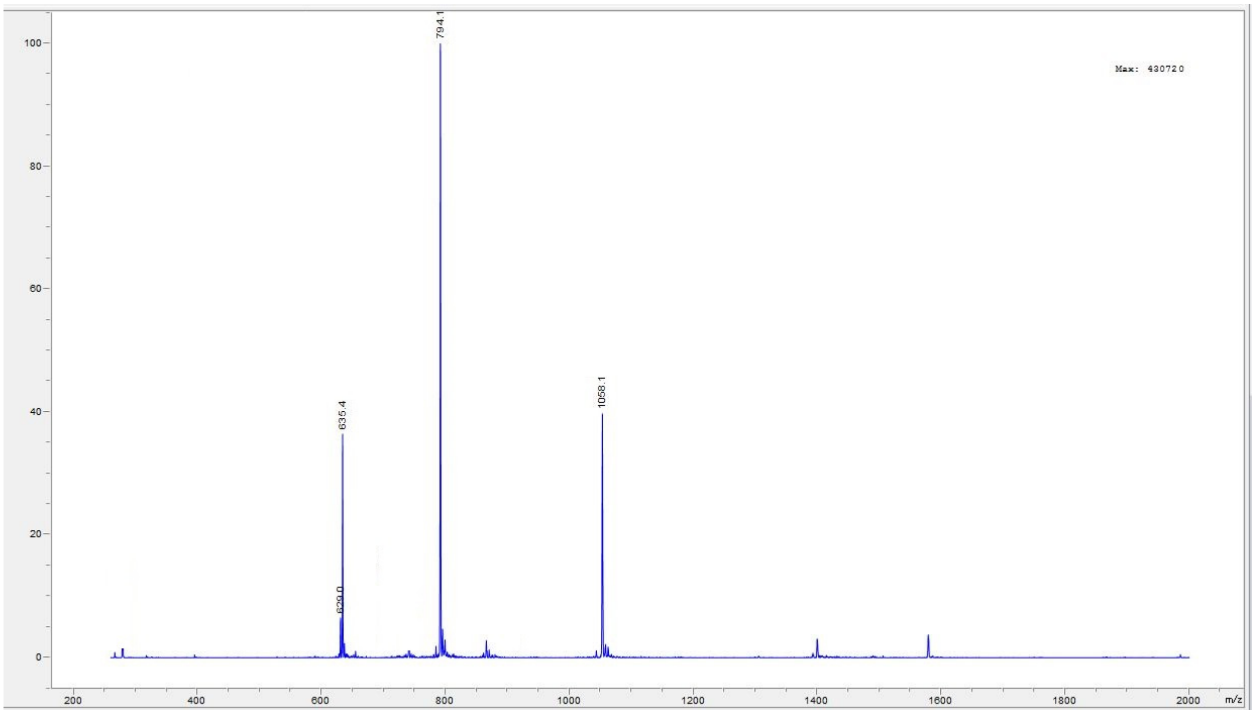

Supplementary Figure 104. LC-MS Spectra for peptide **73.**

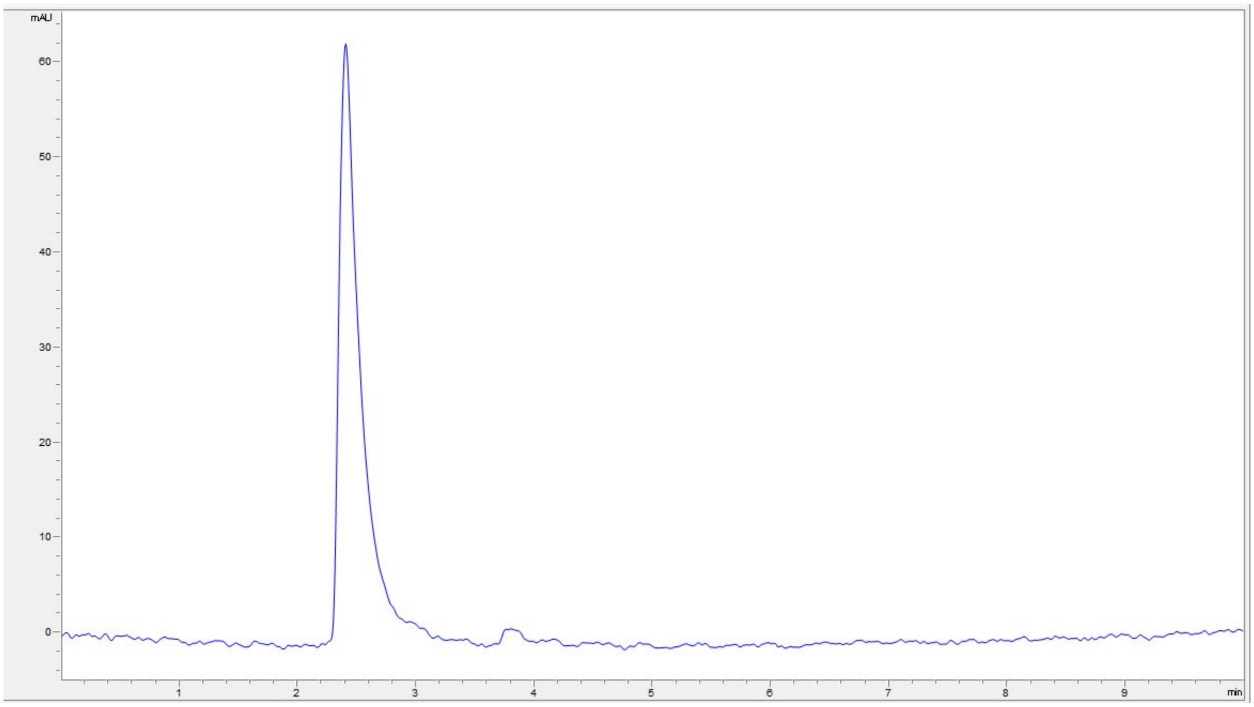

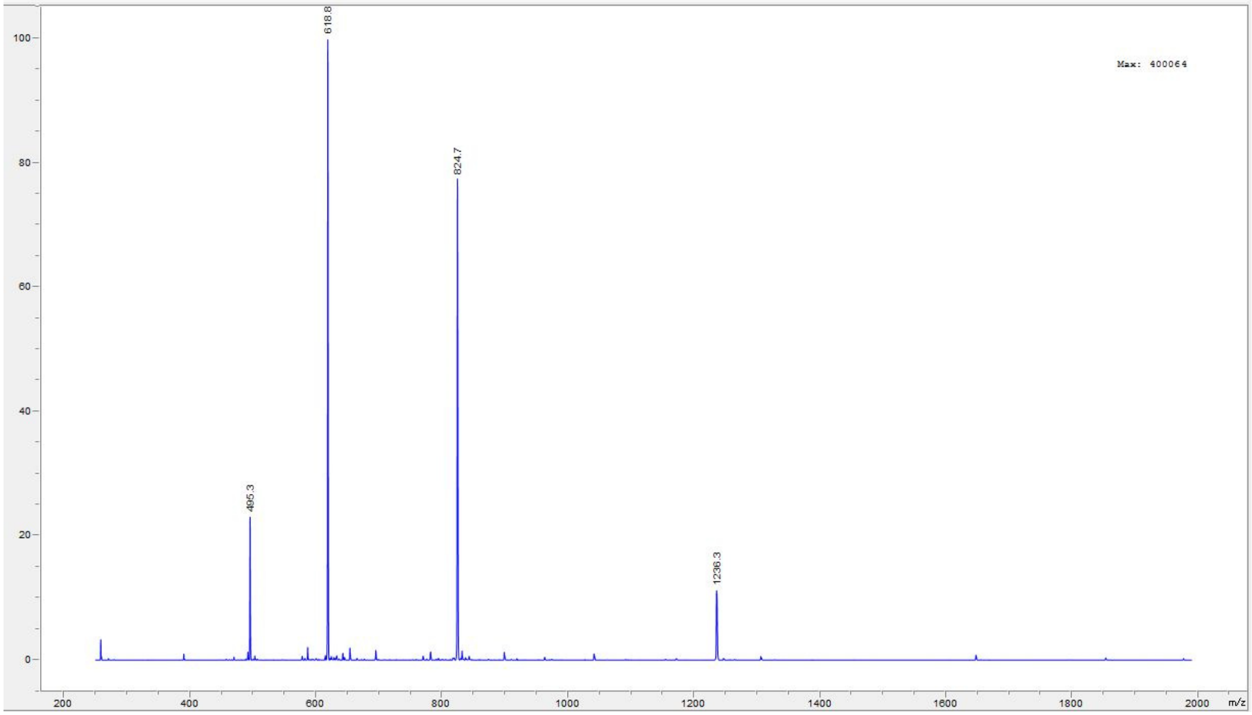

Supplementary Figure 105. LC-MS Spectra for peptide **74.**

Supplementary Figure 106. LC-MS Spectra for peptide 75.

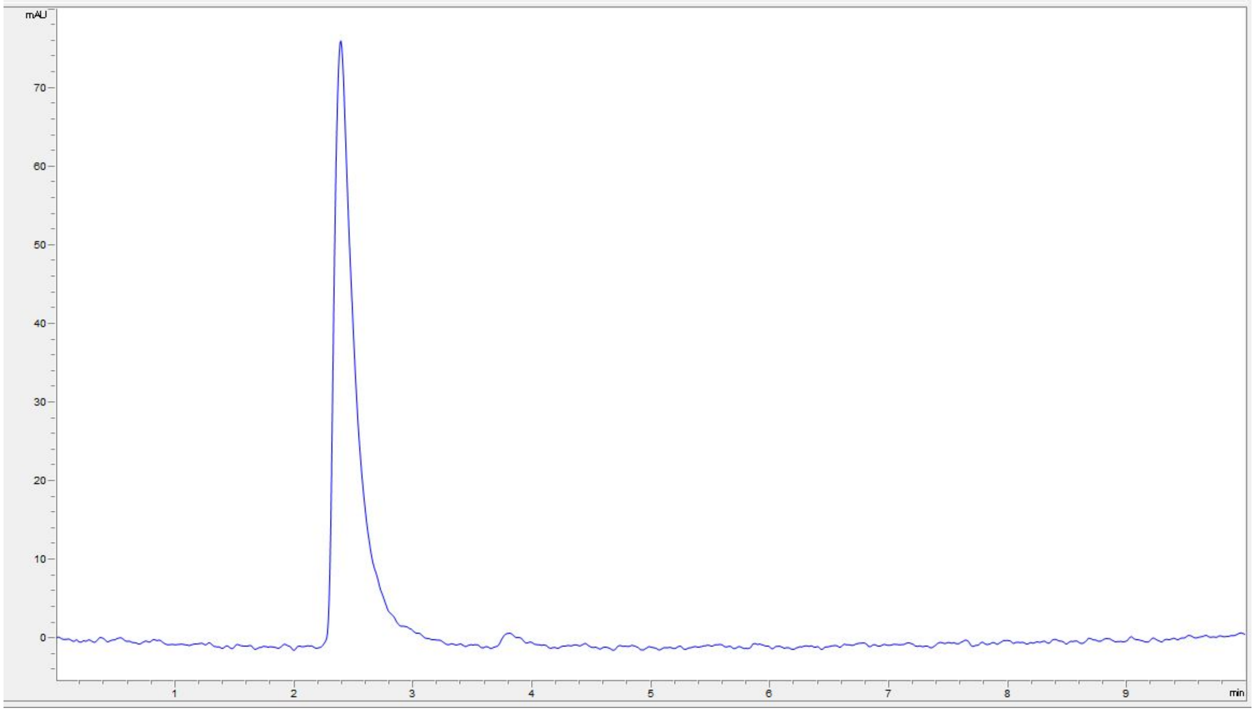

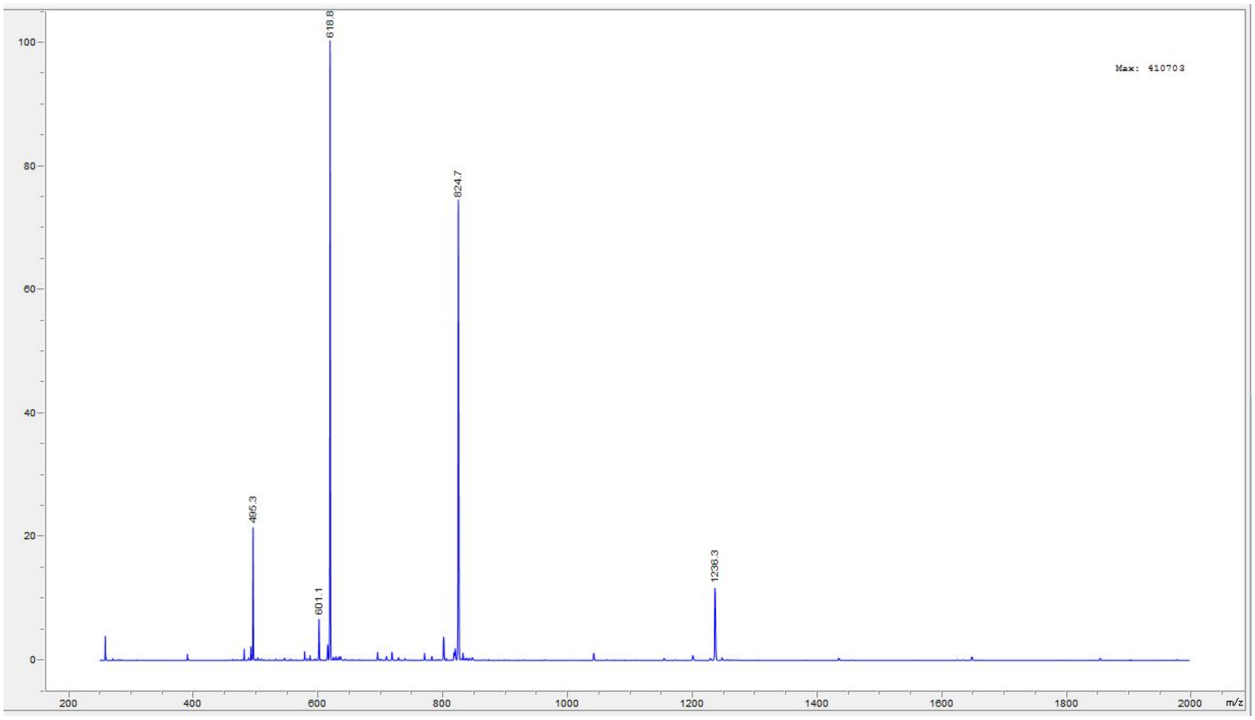

Supplementary Figure 107. LC-MS Spectra for peptide **76.**

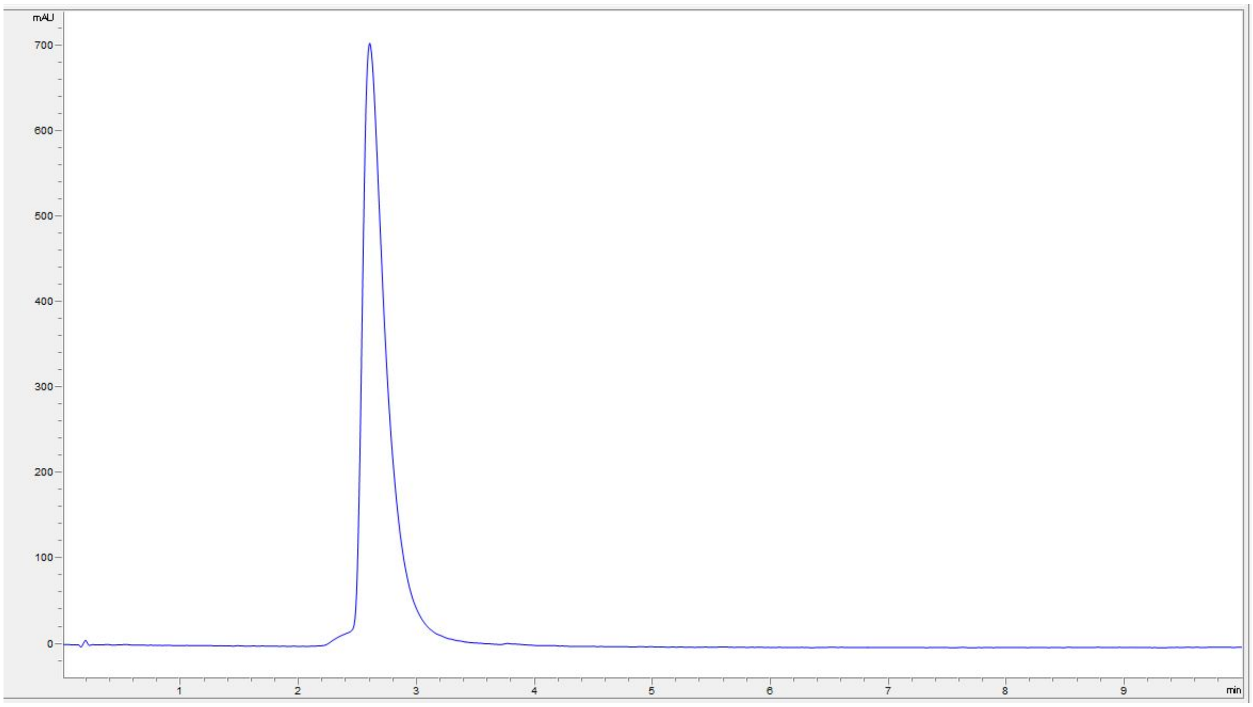

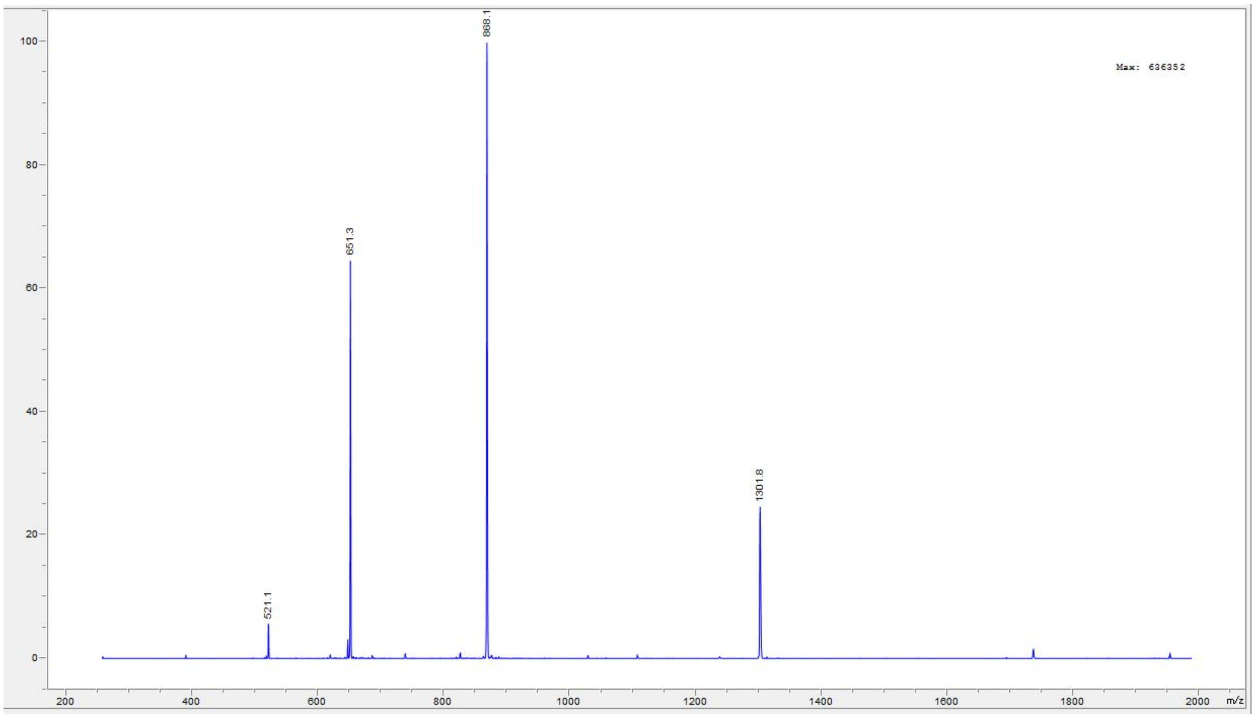

Supplementary Figure 108. LC-MS Spectra for peptide **77.**

Supplementary Figure 109 (the heading at the top of the page)

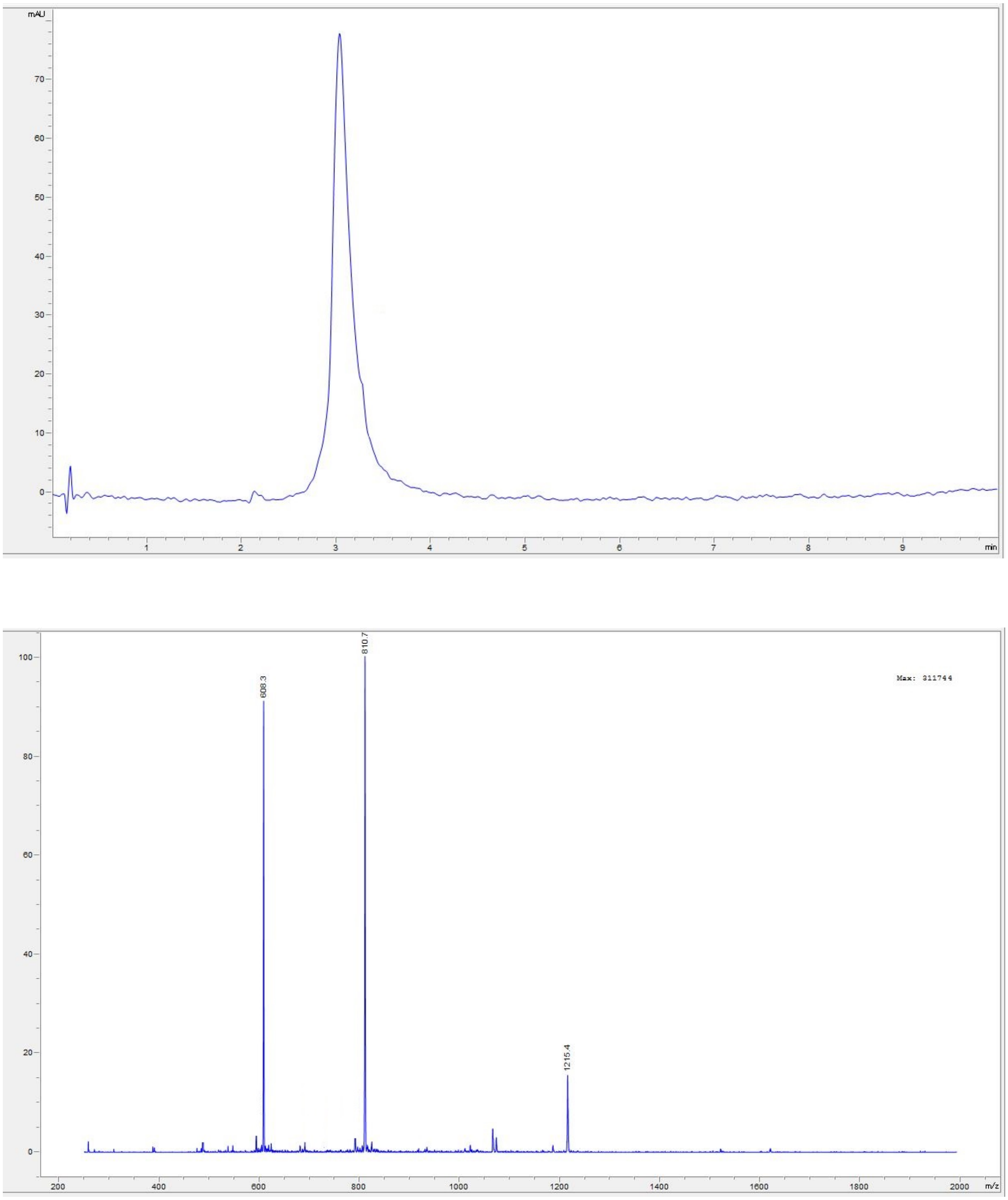

Supplementary Figure 109. LC-MS Spectra for peptide **78.**

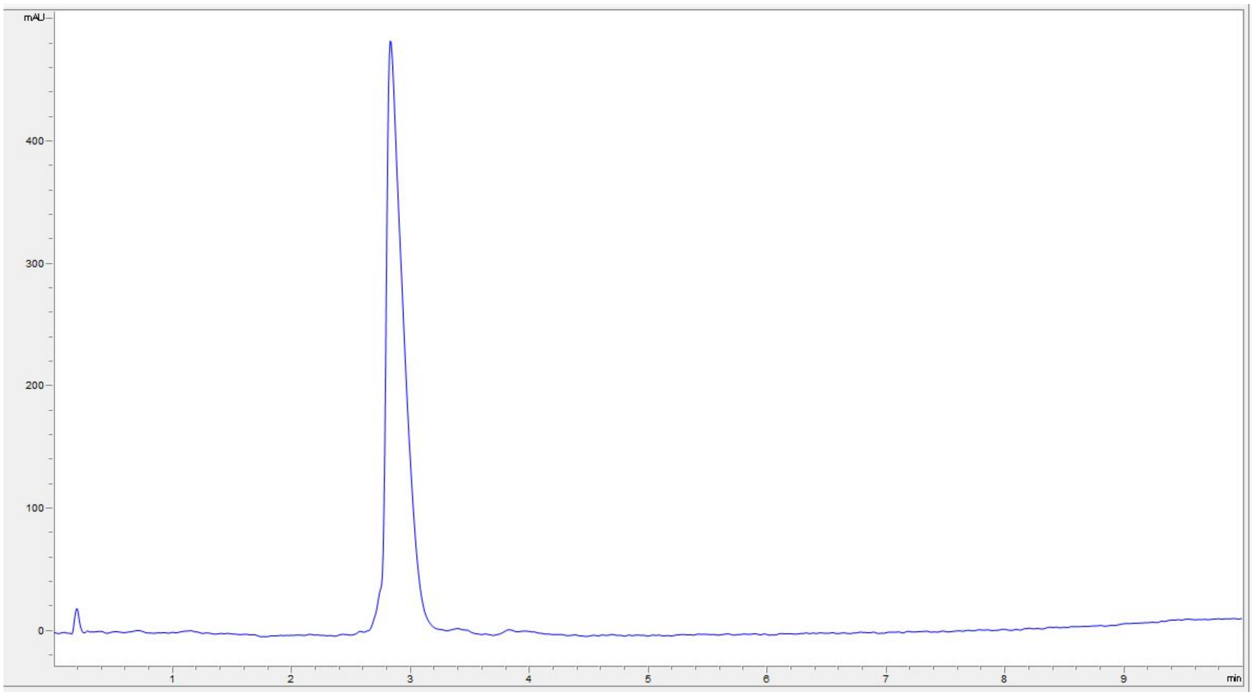

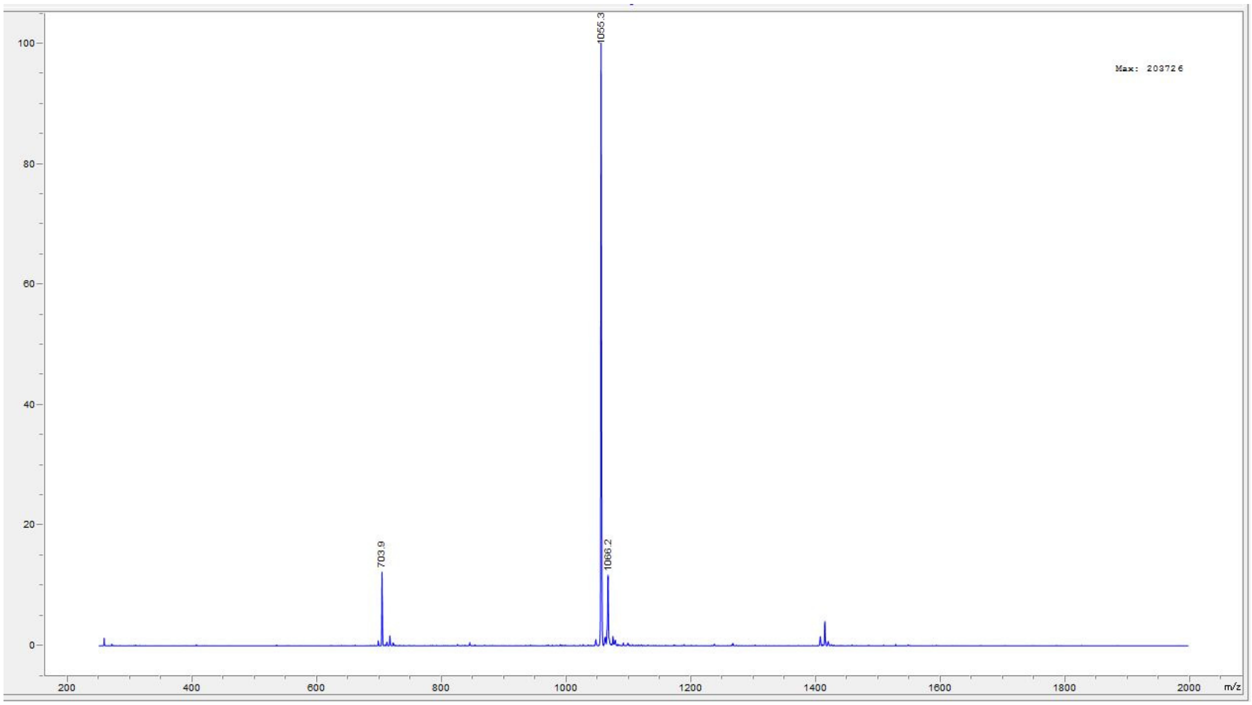

Supplementary Figure 110. LC-MS Spectra for peptide **79.**

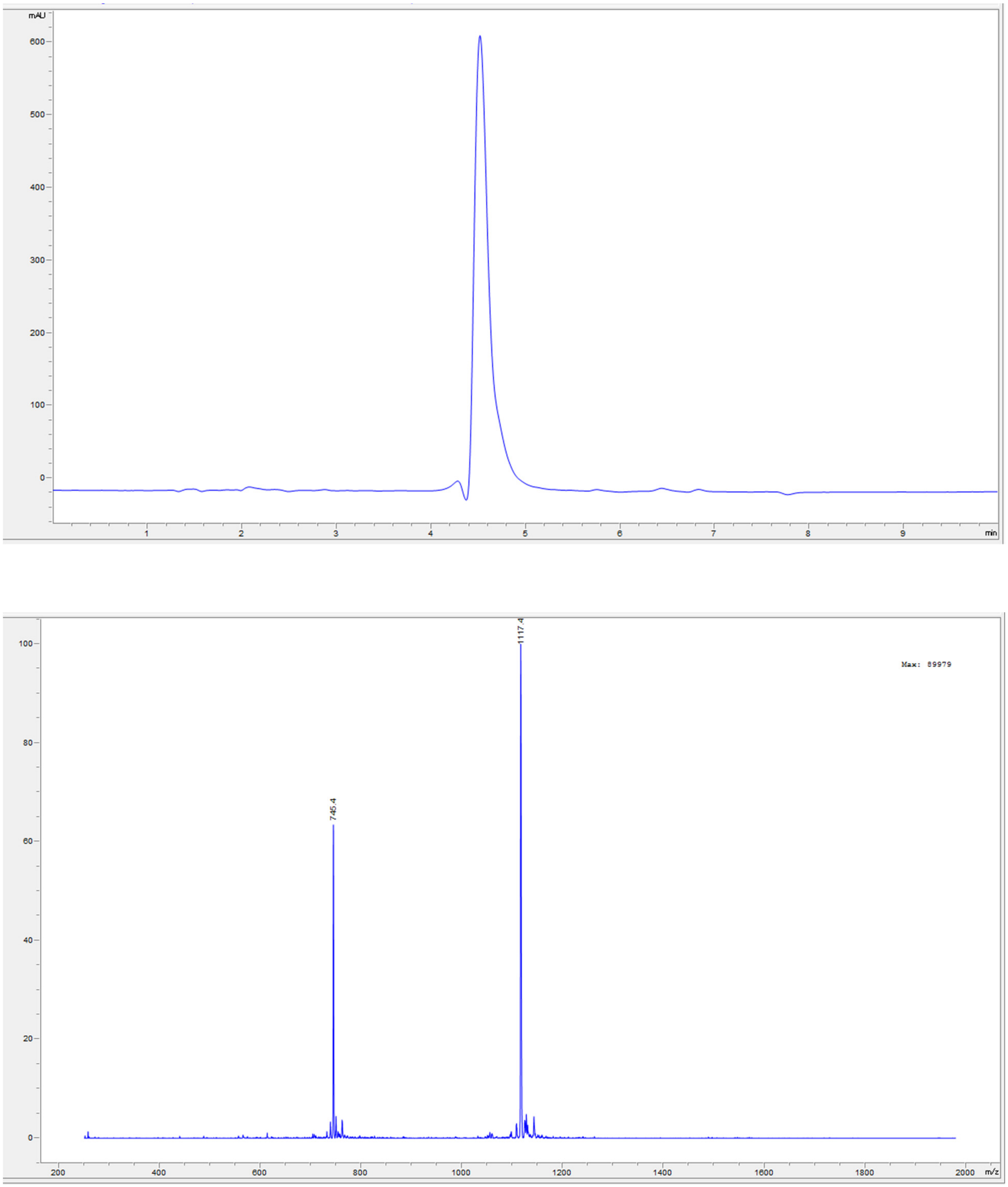

Supplementary Figure 111. LC-MS Spectra for peptide **80.**

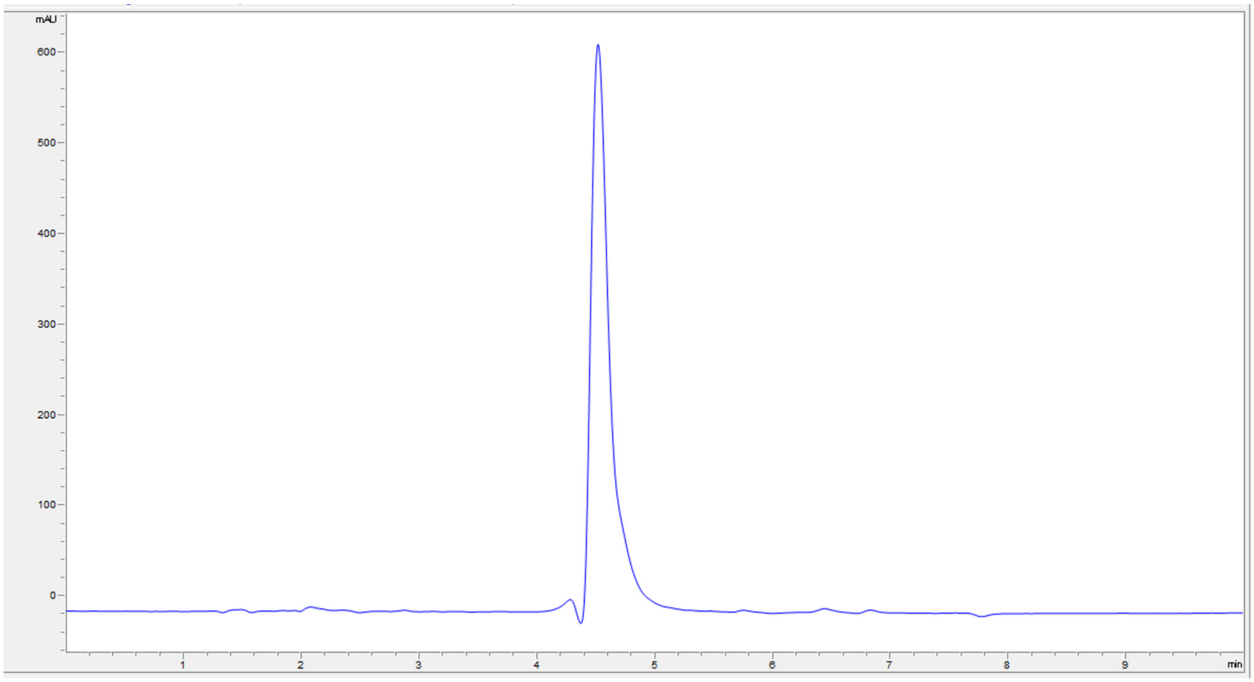

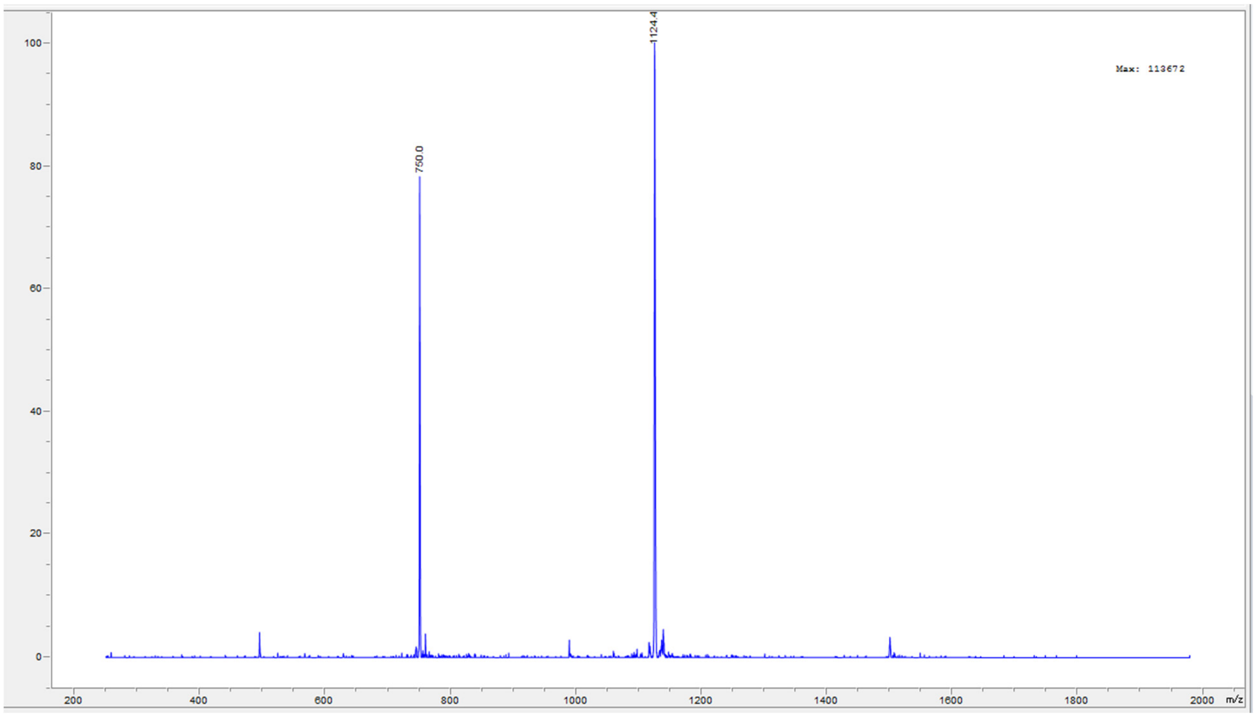

Supplementary Figure 112. LC-MS Spectra for peptide **81.**

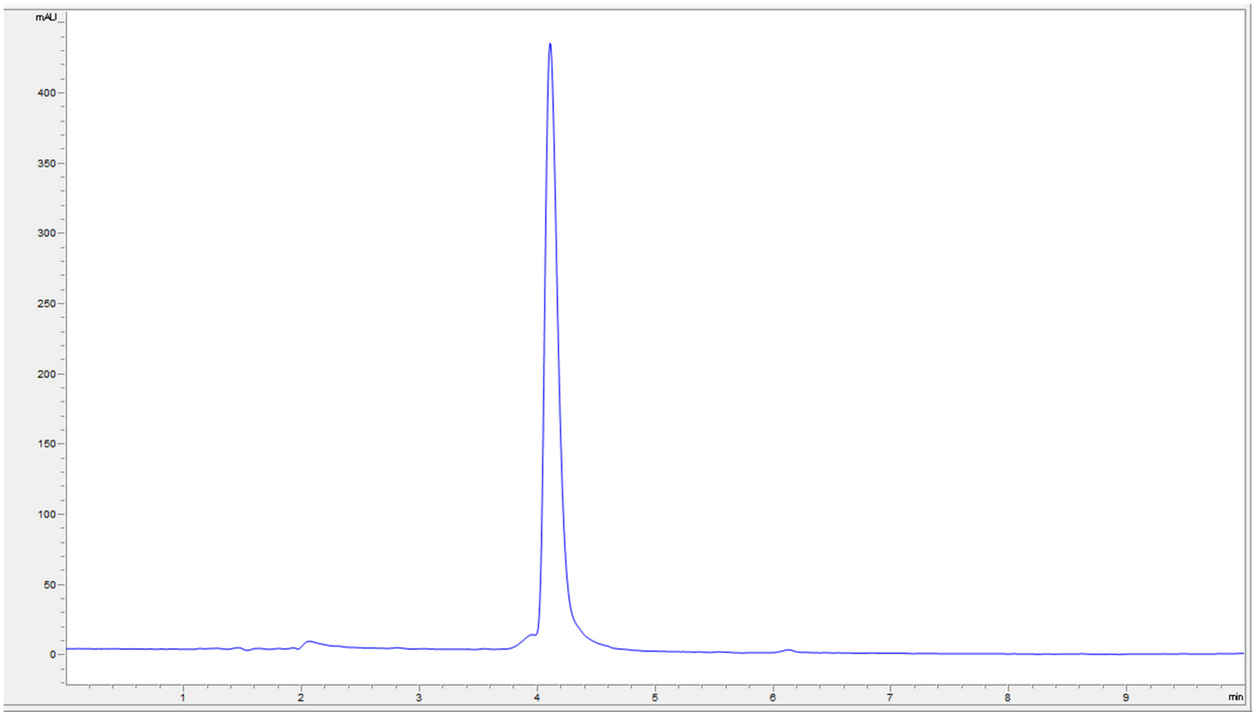

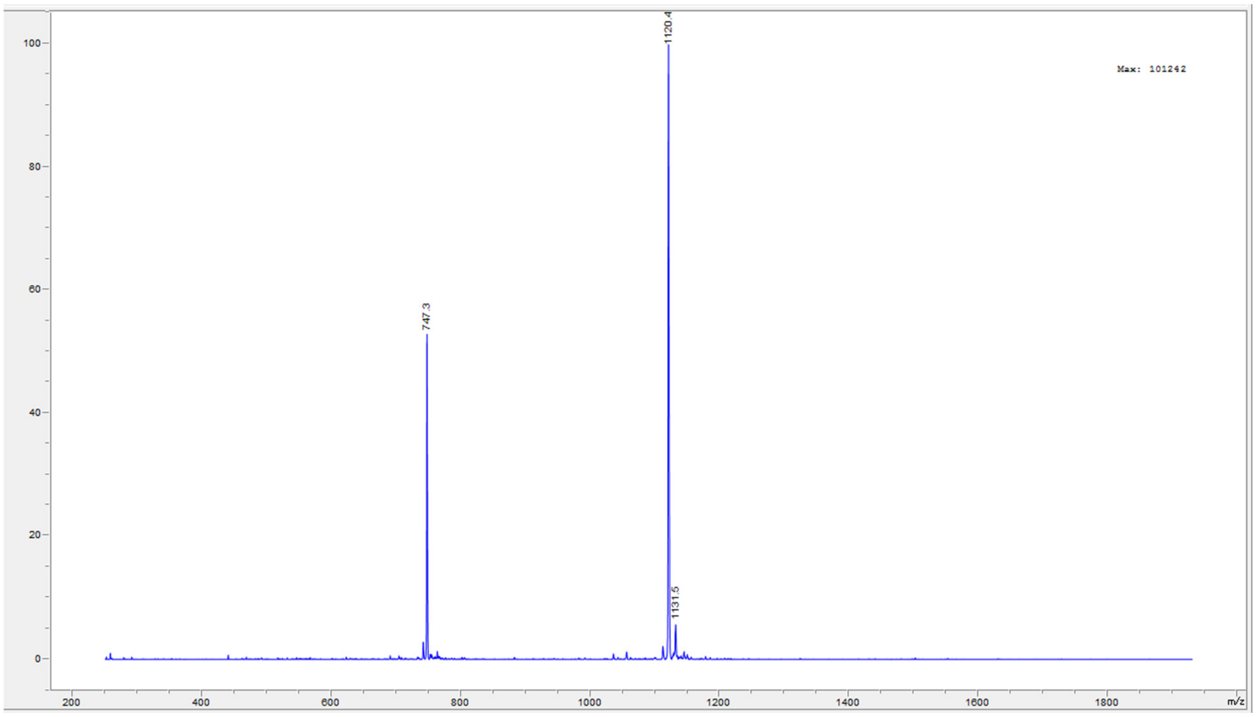

Supplementary Figure 113. LC-MS Spectra for peptide **82.**

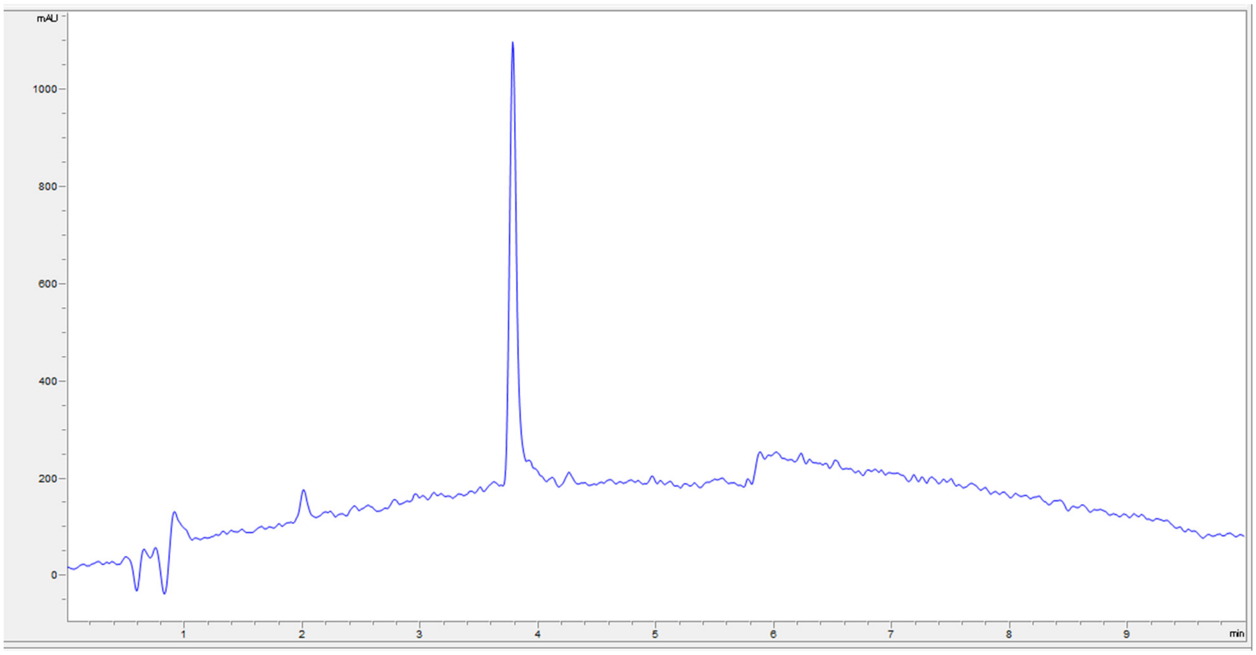

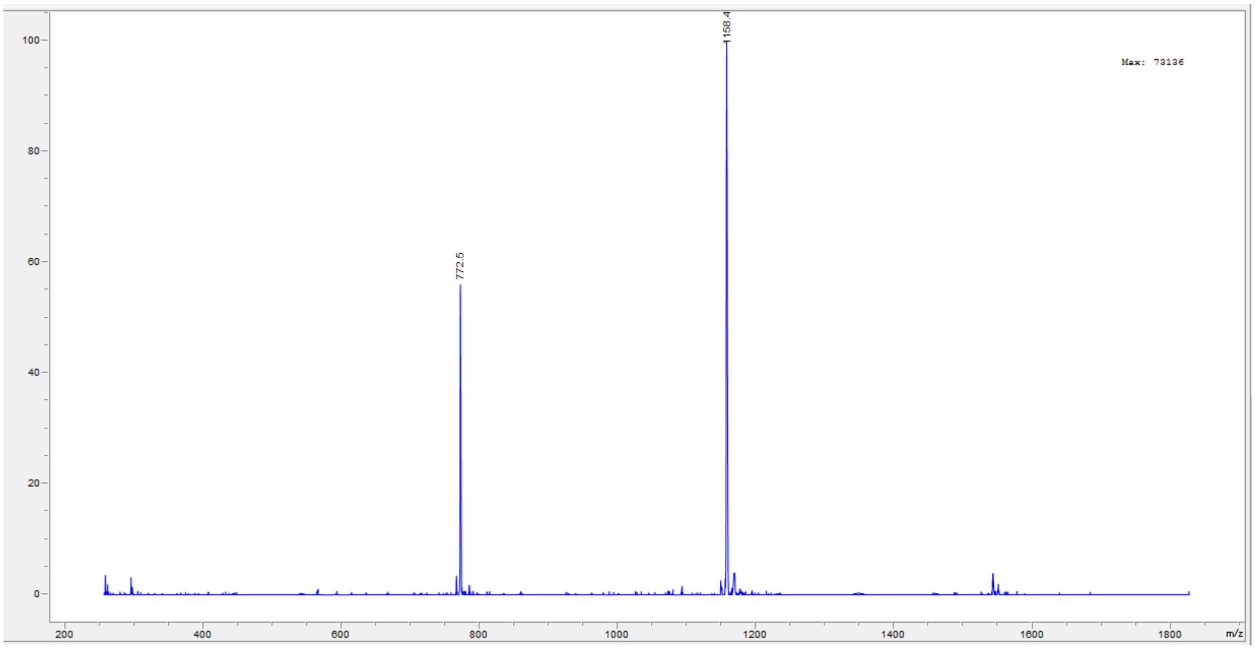

Supplementary Figure 114. LC-MS Spectra for peptide **83.**

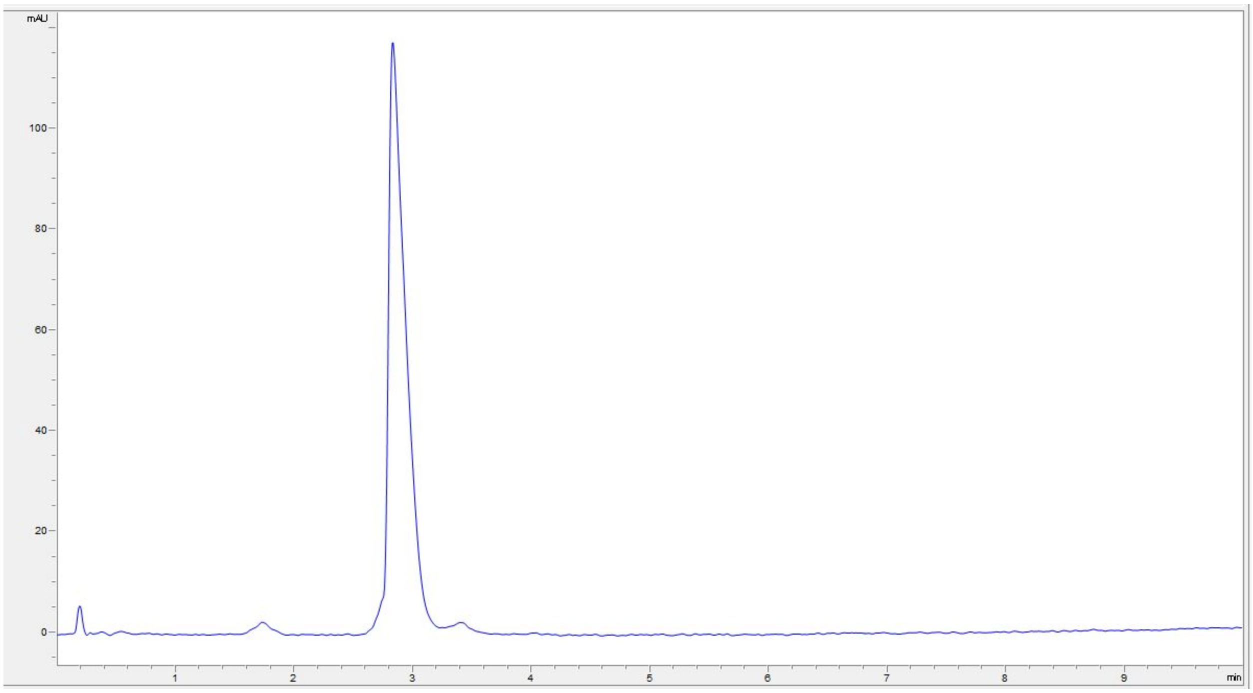

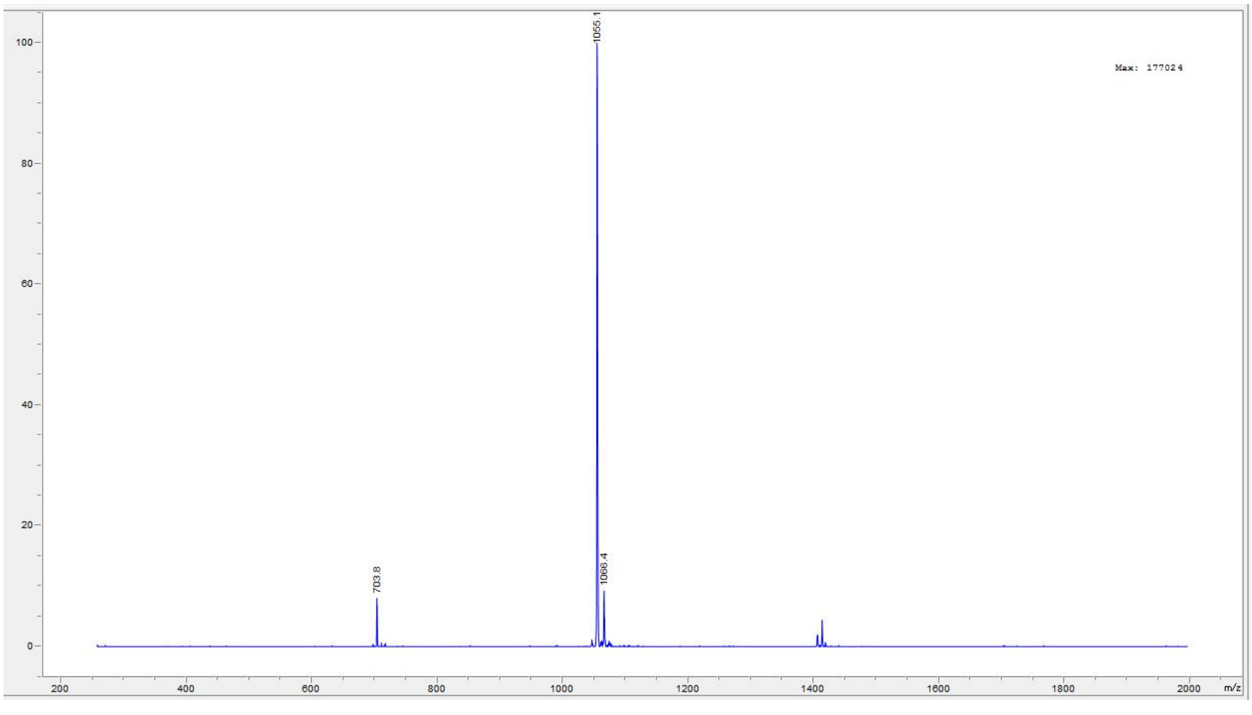

Supplementary Figure 115. LC-MS Spectra for peptide **84.**

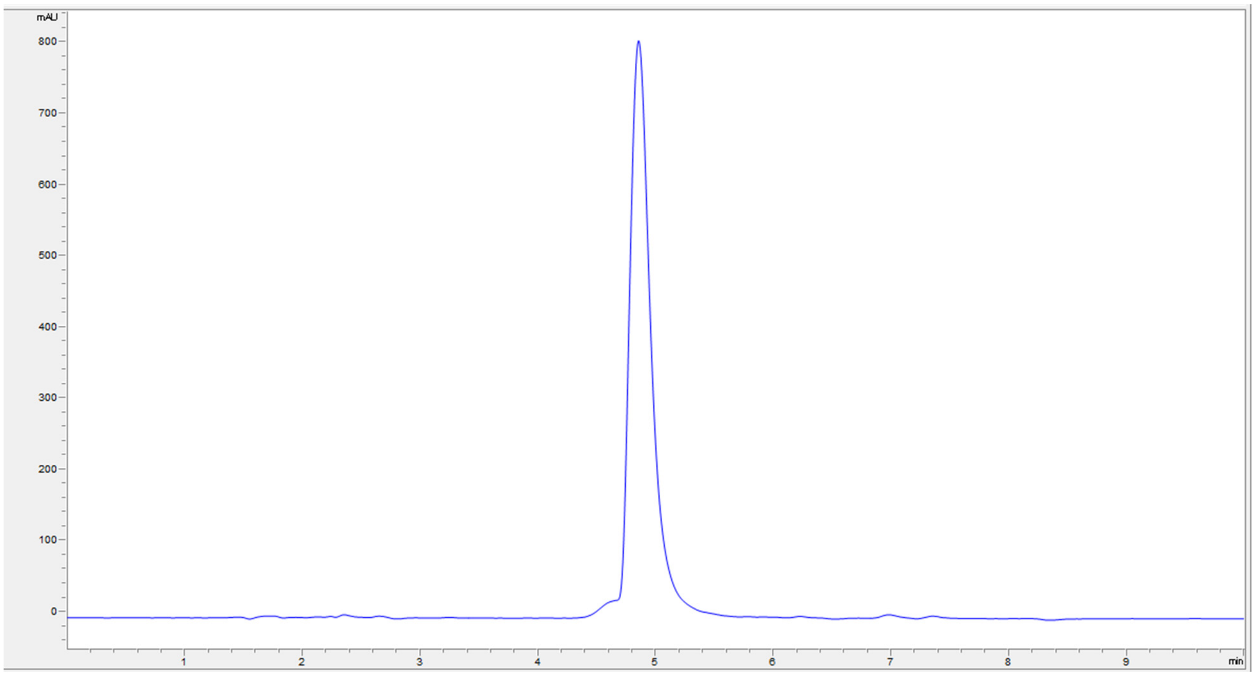

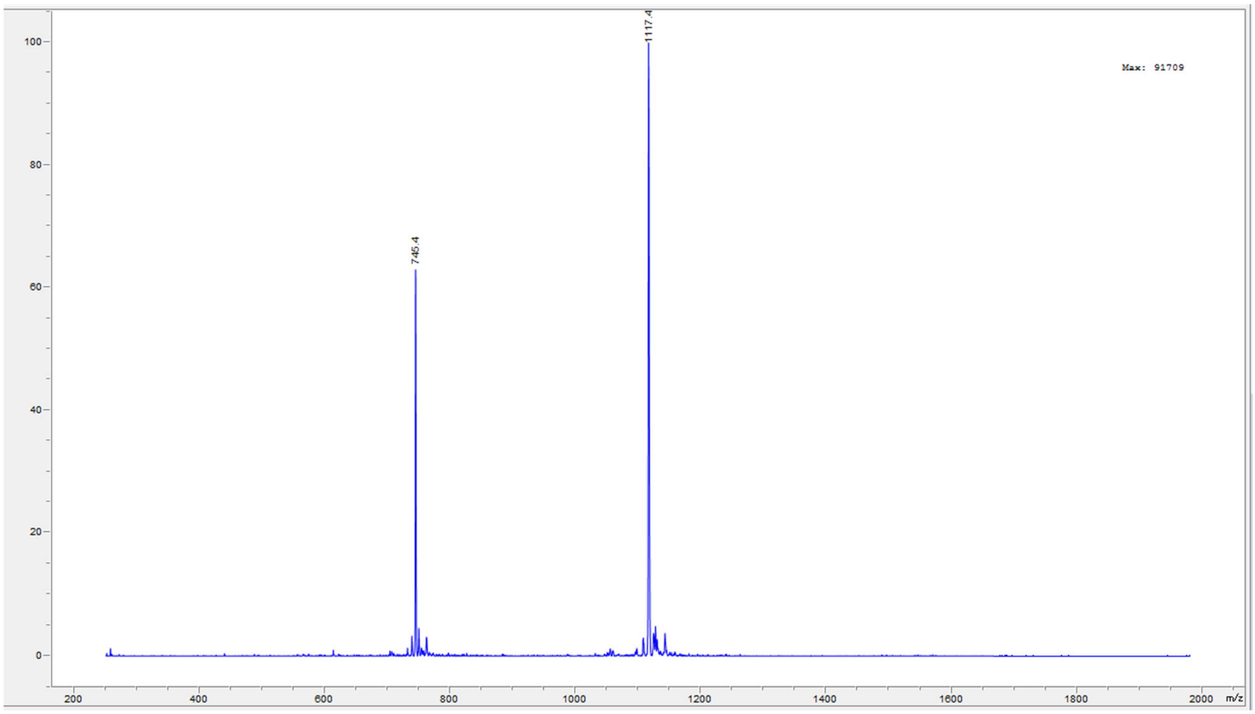

Supplementary Figure 116. LC-MS Spectra for peptide **85.**

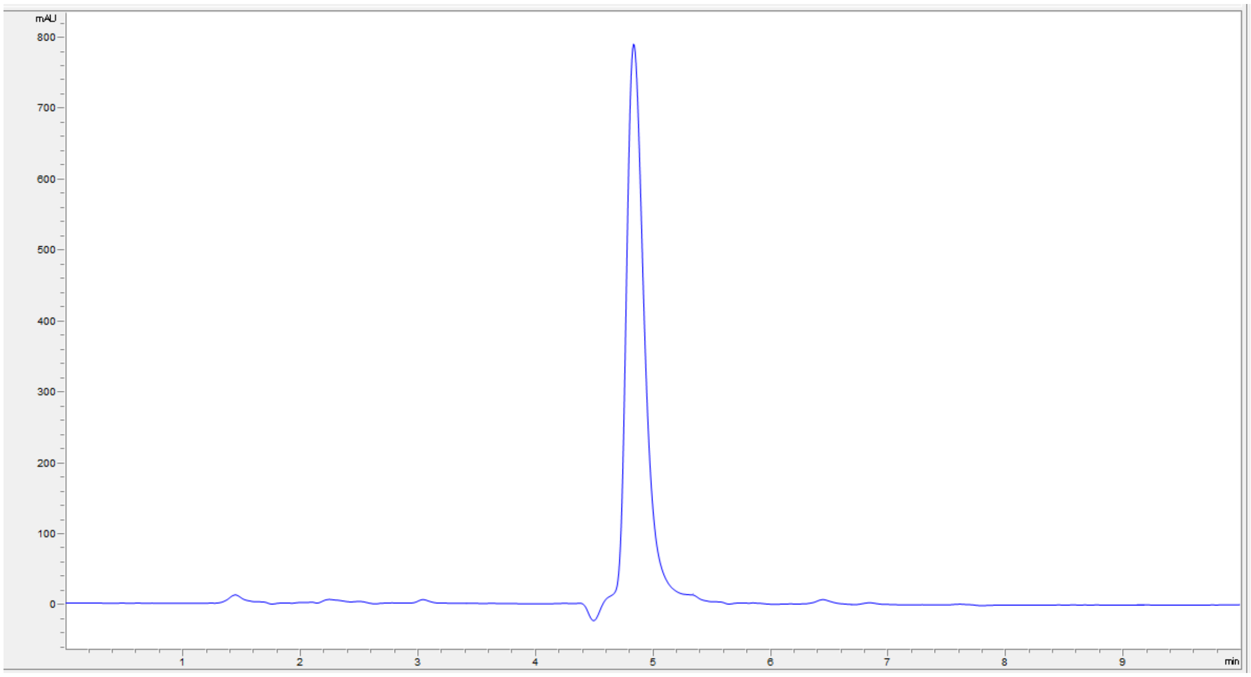

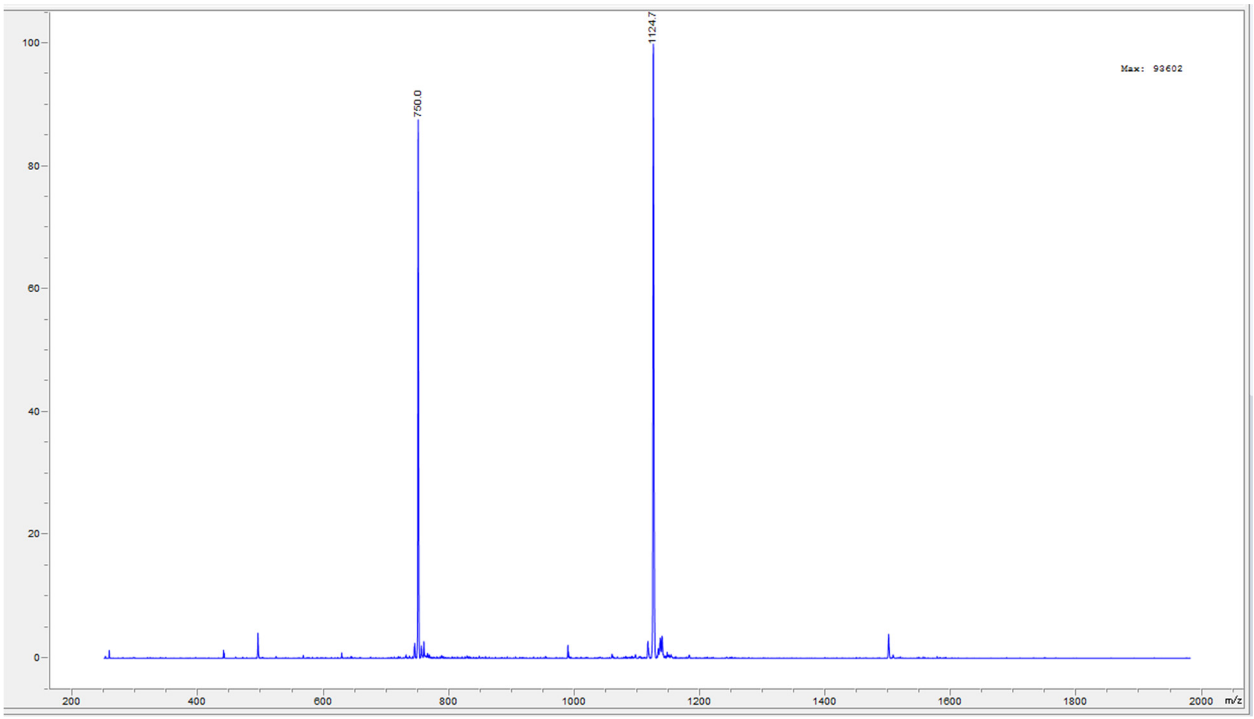

Supplementary Figure 117. LC-MS Spectra for peptide **86.**

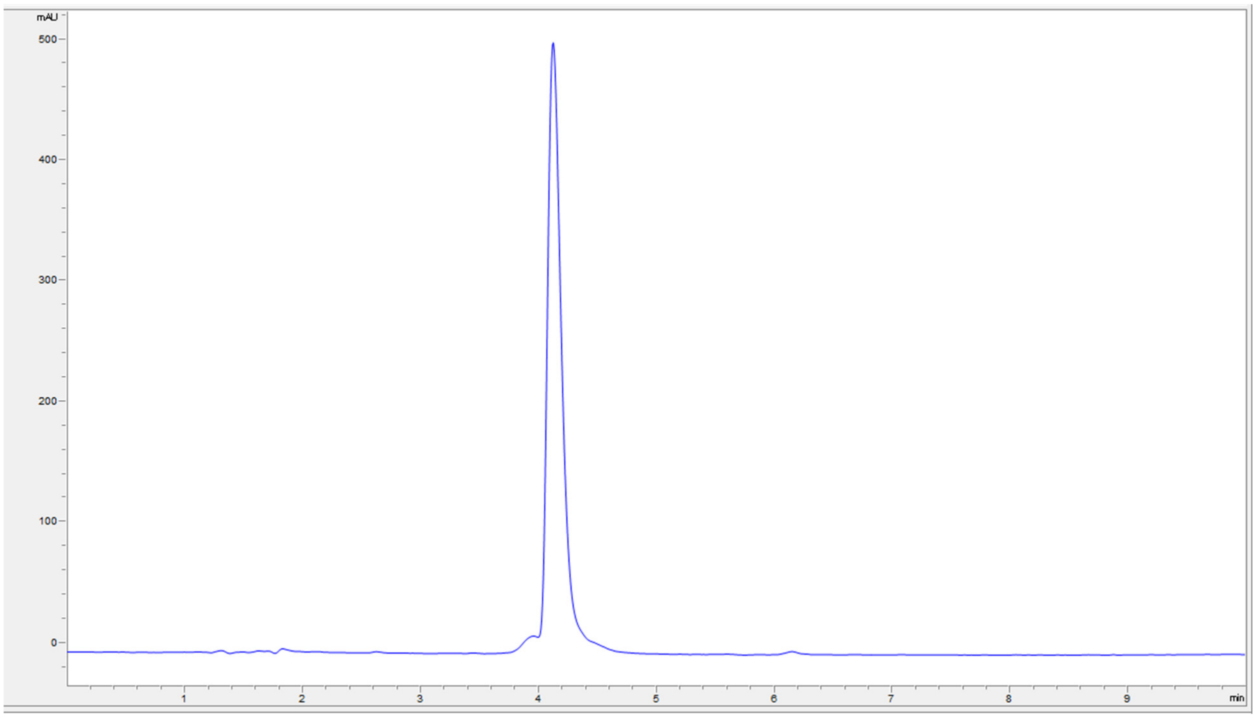

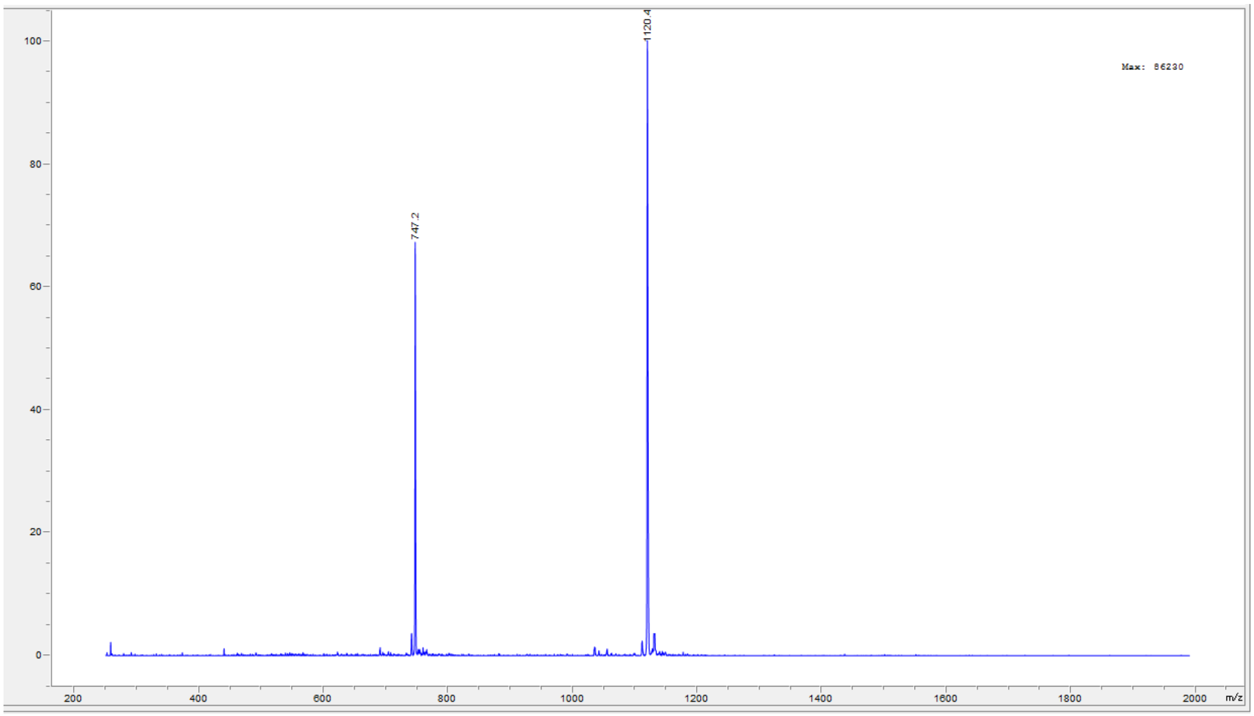

Supplementary Figure 118. LC-MS Spectra for peptide **87.**

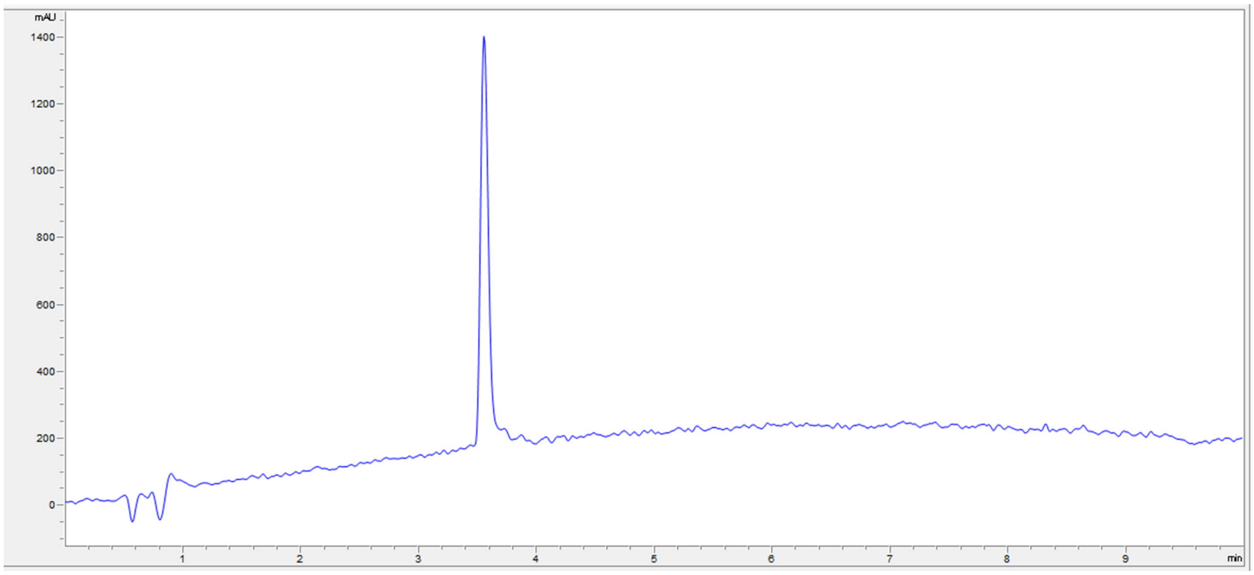

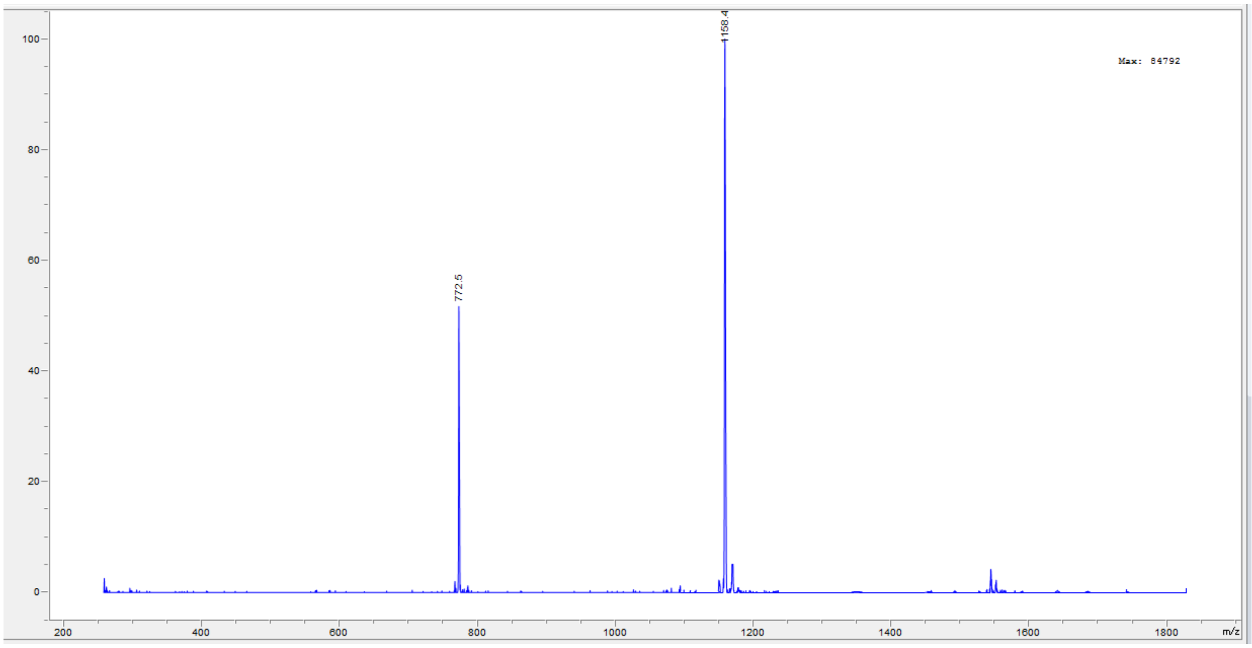

Supplementary Figure 119. LC-MS Spectra for peptide **88.**

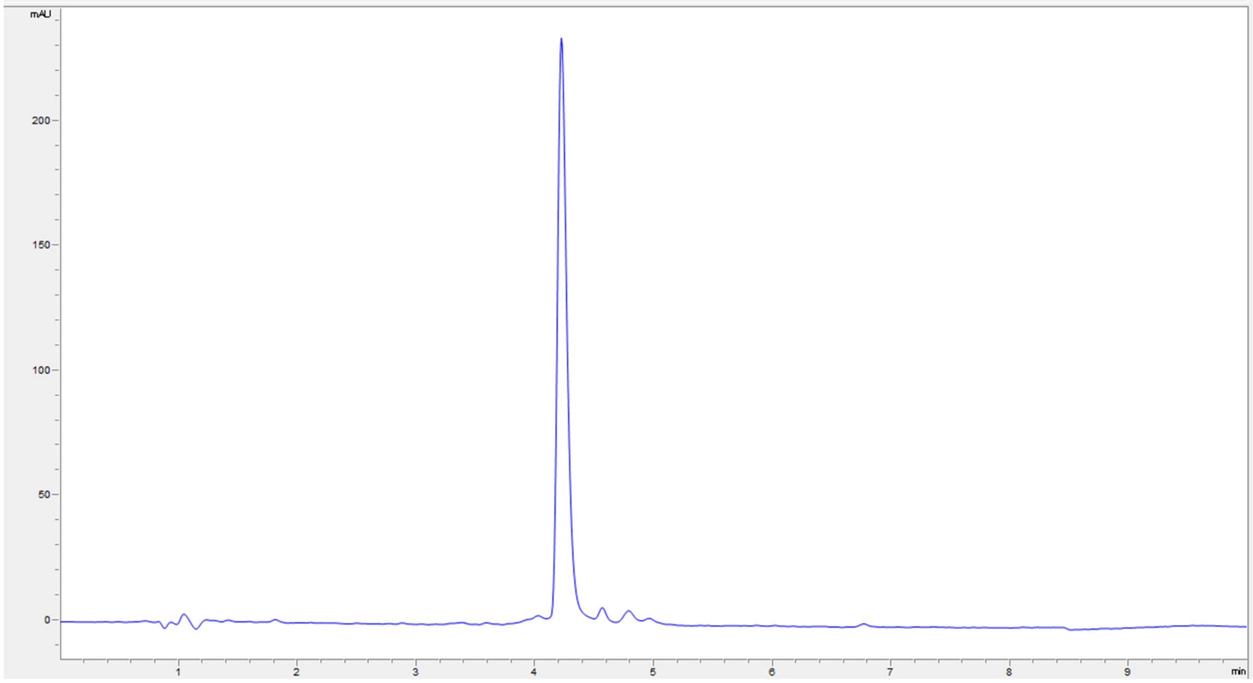

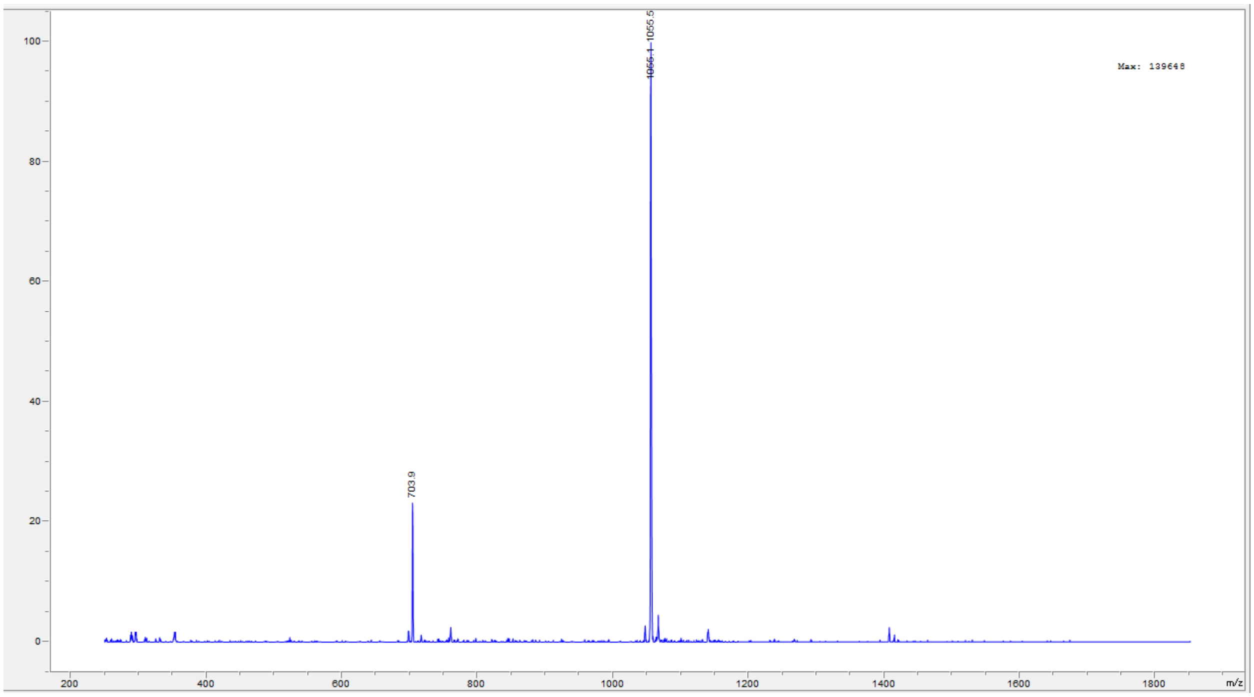

Supplementary Figure 120. LC-MS Spectra for peptide **89.**

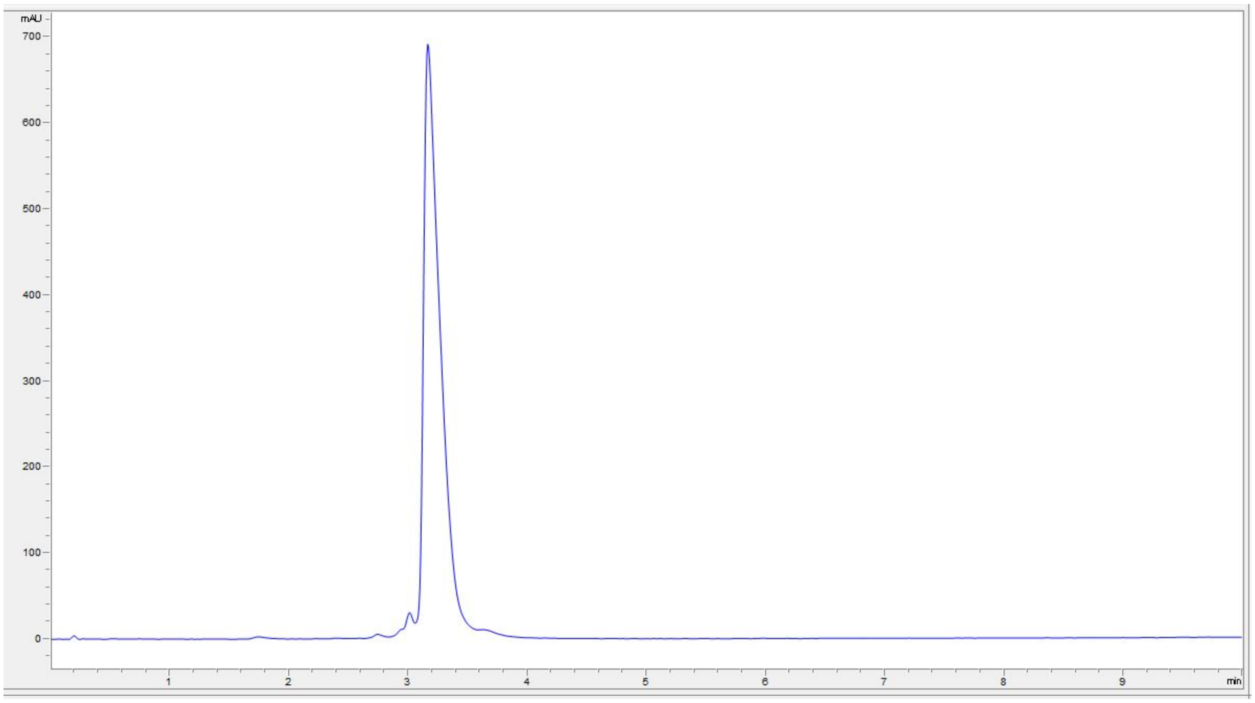

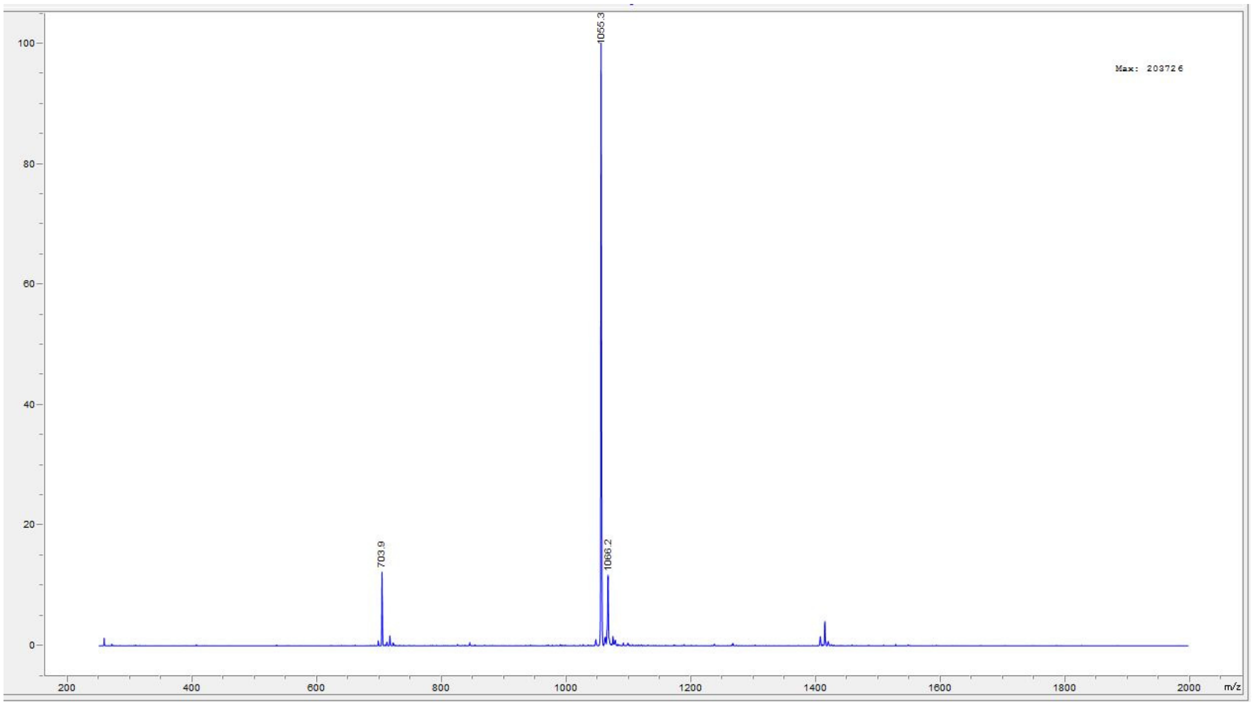

Supplementary Figure 121. LC-MS Spectra for peptide **90.**

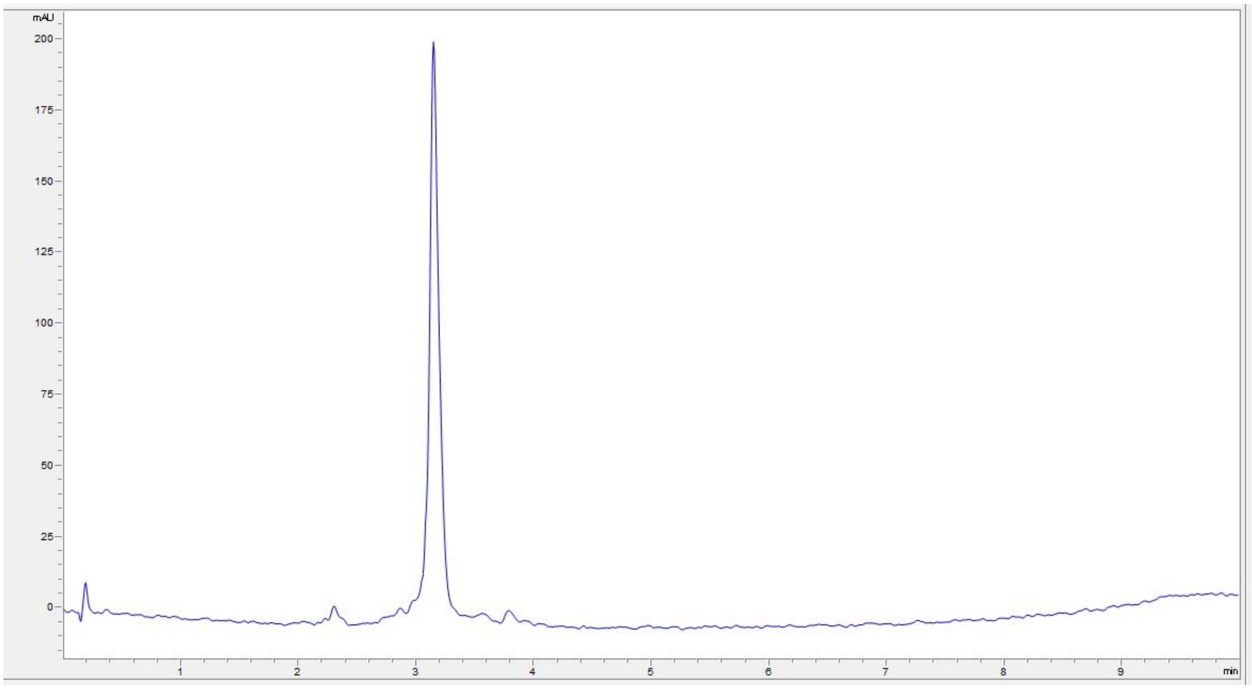

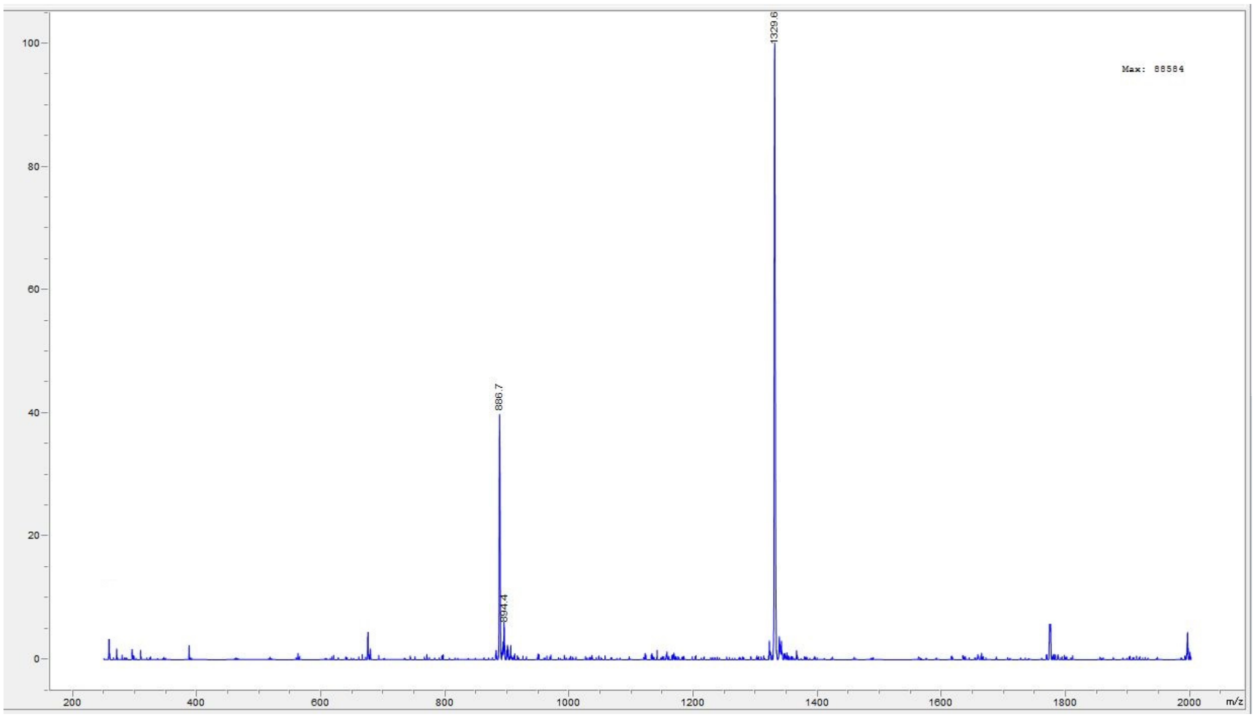

Supplementary Figure 122. LC-MS Spectra for peptide **91.**

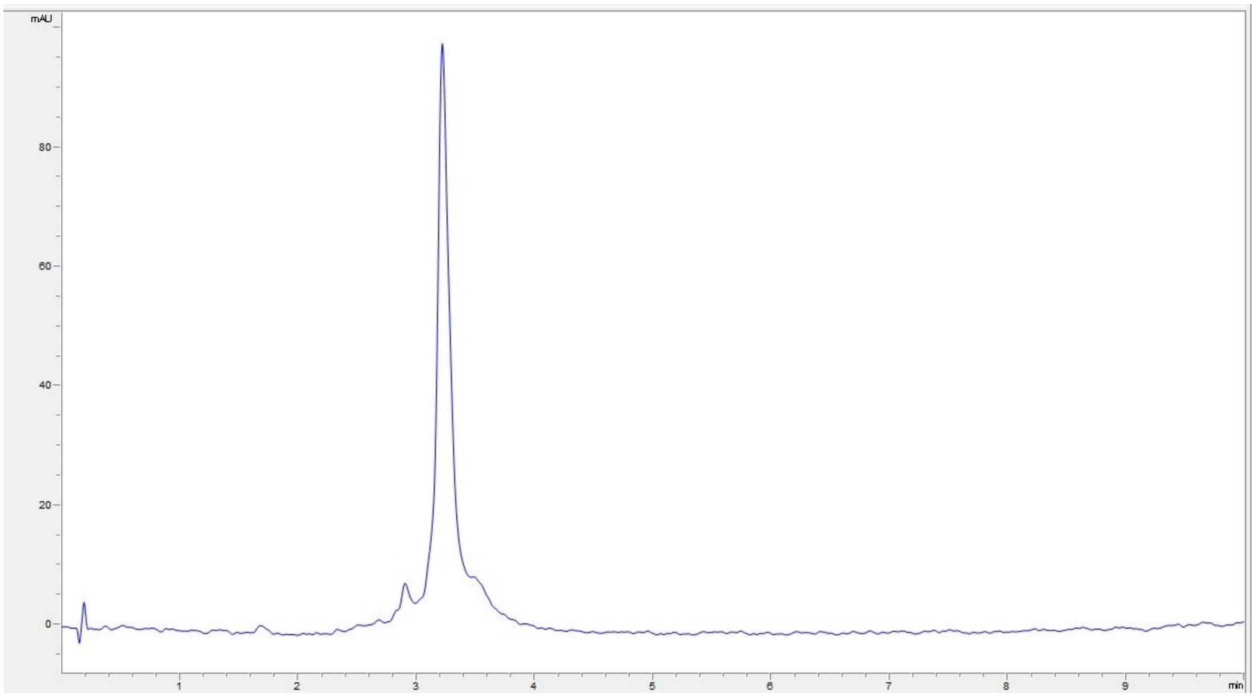

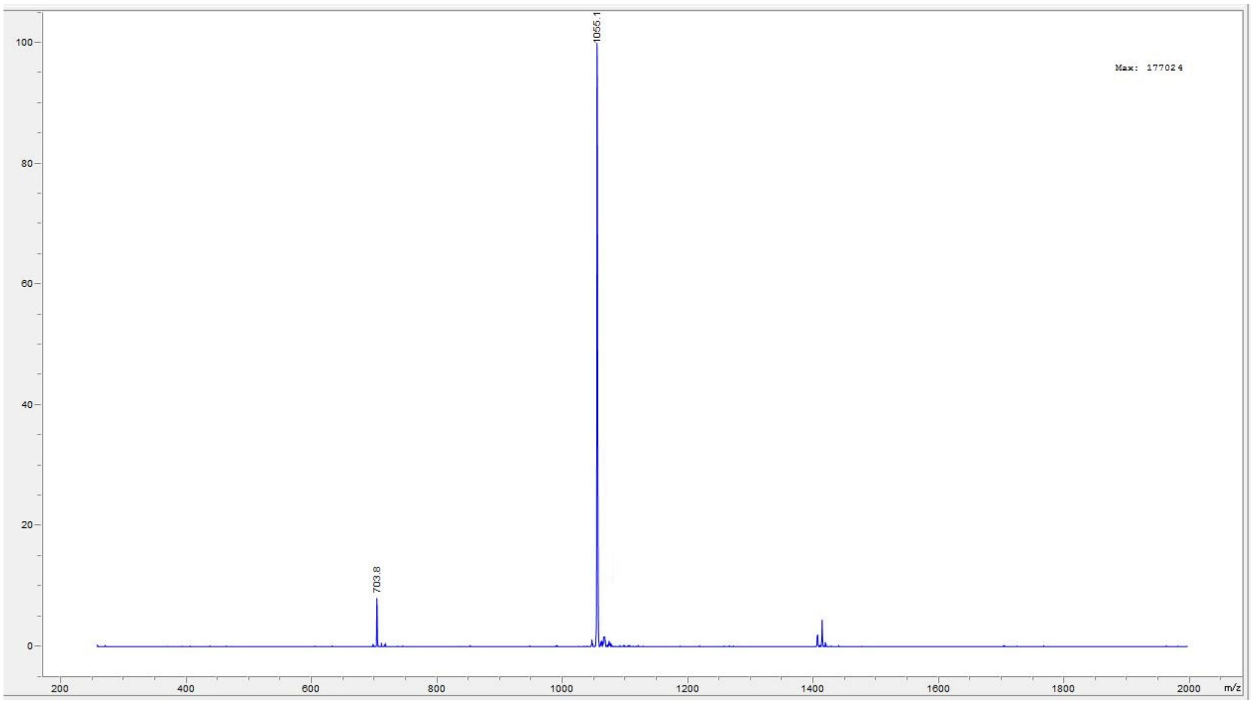

Supplementary Figure 123. LC-MS Spectra for peptide **92.**

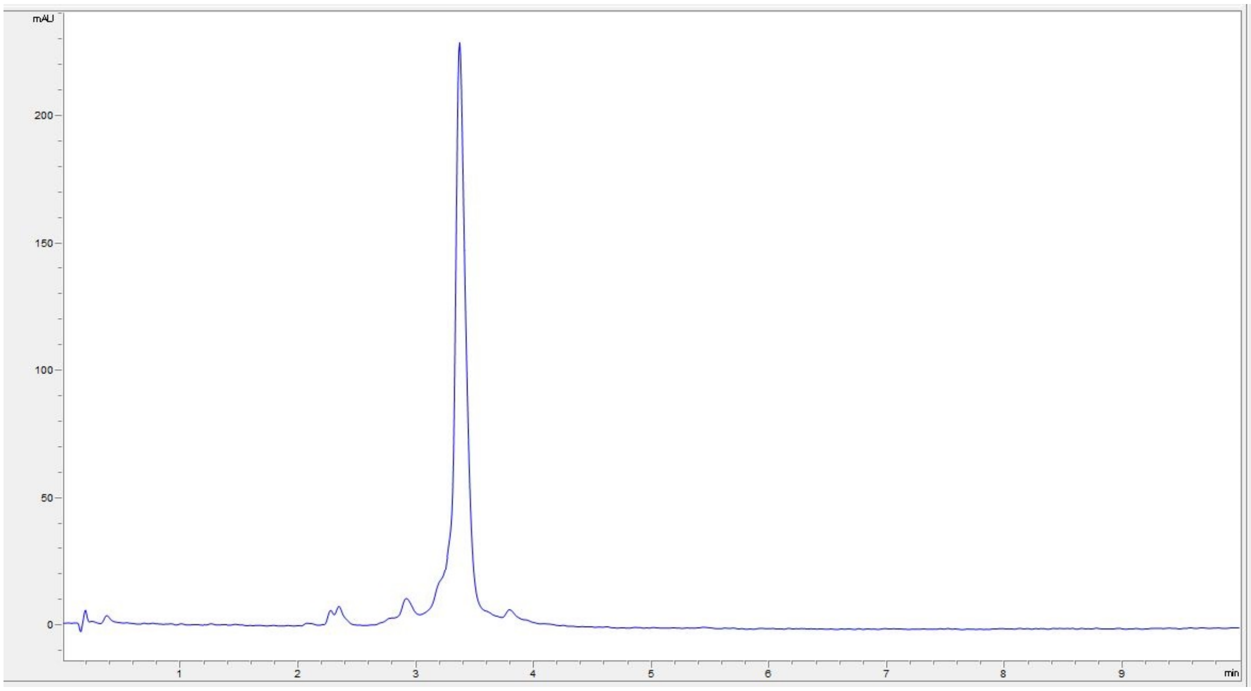

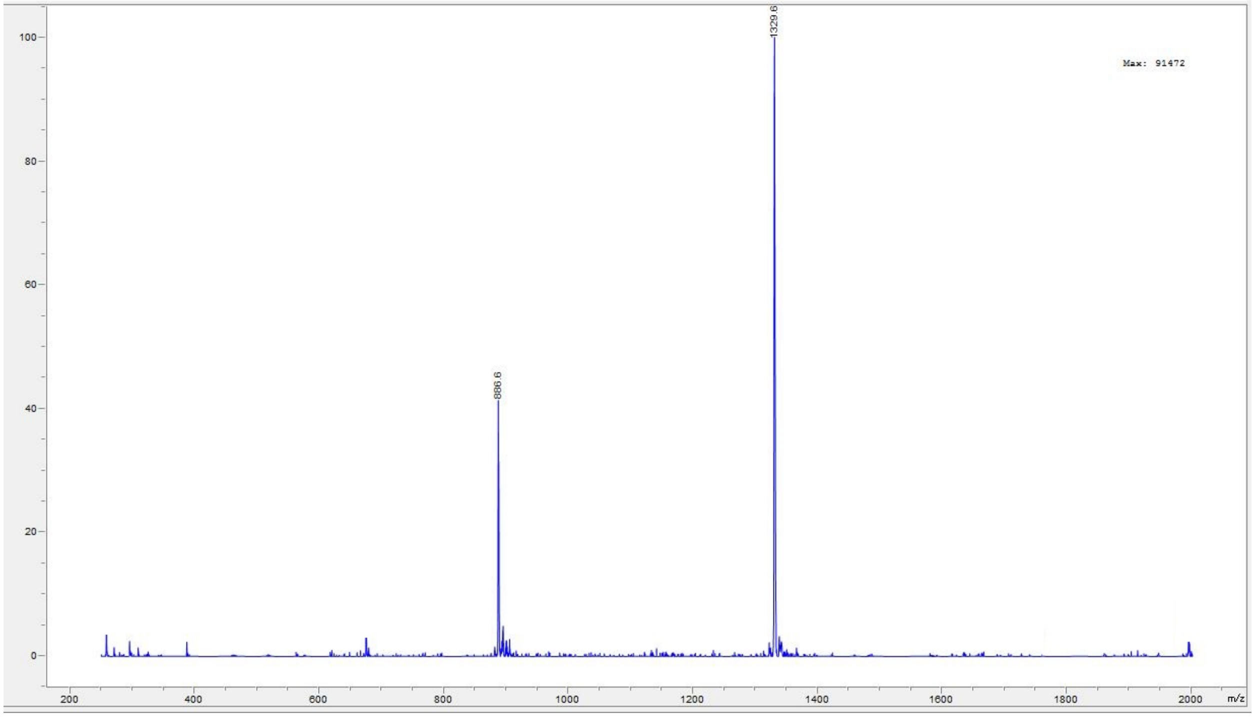

Supplementary Figure 124. LC-MS Spectra for peptide **93.**

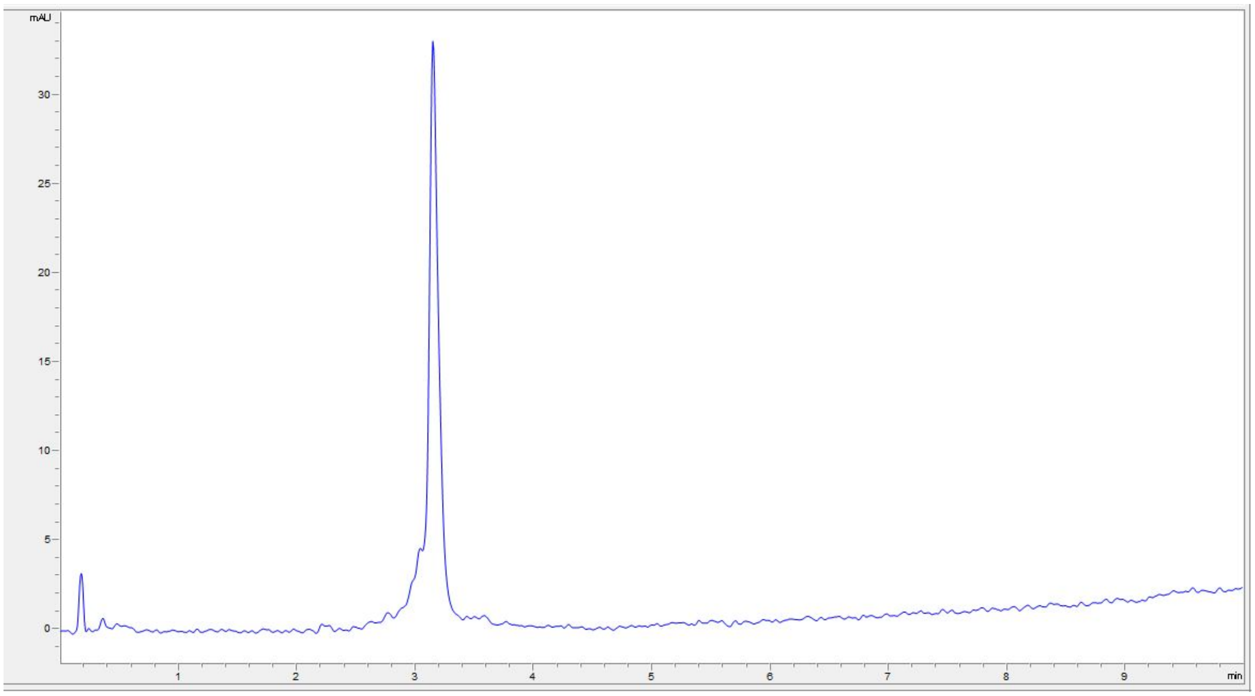

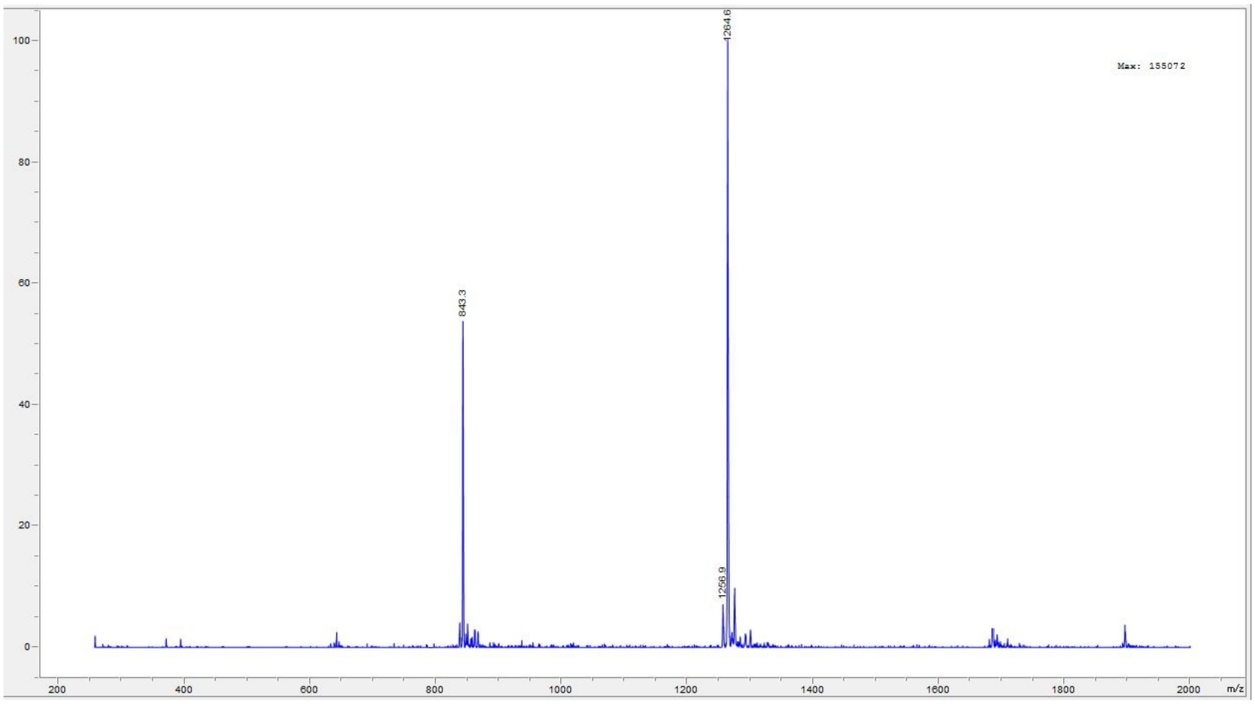

Supplementary Figure 125. LC-MS Spectra for peptide **94.**
