# Supplementary material for: Unprotected peptide macrocyclization and stapling via a fluorine-thiol displacement reaction
Source: Nat Commun. 2022 Jan 17;13:350. doi: 10.1038/s41467-022-27995-5 (PMC8763920; doi:10.1038/s41467-022-27995-5)
Supplement: Supplementary file 3 — Reporting Summary [file 41467_2022_27995_MOESM3_ESM.pdf]

nature portfolio

Corresponding author(s): Rongsheng E. Wang

Last updated by author(s): November 19, 2021

Reporting Summary

Nature Portfolio wishes to improve the reproducibility of the work that we publish. This form provides structure for consistency and transparency in reporting. For further information on Nature Portfolio policies, see our Editorial Policies and the Editorial Policy Checklist.

Statistics

For all statistical analyses, confirm that the following items are present in the figure legend, table legend, main text, or Methods section.

n/a	Confirmed	
☐	☒	The exact sample size (n) for each experimental group/condition, given as a discrete number and unit of measurement
☐	☒	A statement on whether measurements were taken from distinct samples or whether the same sample was measured repeatedly
☐	☒	The statistical test(s) used AND whether they are one- or two-sided<br>*Only common tests should be described solely by name; describe more complex techniques in the Methods section.*
☒	☐	A description of all covariates tested
☒	☐	A description of any assumptions or corrections, such as tests of normality and adjustment for multiple comparisons
☐	☒	A full description of the statistical parameters including central tendency (e.g. means) or other basic estimates (e.g. regression coefficient) AND variation (e.g. standard deviation) or associated estimates of uncertainty (e.g. confidence intervals)
☐	☒	For null hypothesis testing, the test statistic (e.g. F, t, r) with confidence intervals, effect sizes, degrees of freedom and P value noted<br>*Give P values as exact values whenever suitable.*
☒	☐	For Bayesian analysis, information on the choice of priors and Markov chain Monte Carlo settings
☒	☐	For hierarchical and complex designs, identification of the appropriate level for tests and full reporting of outcomes
☒	☐	Estimates of effect sizes (e.g. Cohen's d, Pearson's r), indicating how they were calculated

Our web collection on statistics for biologists contains articles on many of the points above.

Software and code

Policy information about availability of computer code

Data collection	CD data were collected by Jasco Spectra Manager. NMR spectra were collected by Bruker TopSpin and processed by MestreNova V14.2. All cell viability/growth inhibition data, luciferase reporter assay data, and ELISA-based data were detected using Synergy H1 plate reader Gen5 program. Confocal Imaging spectra were obtained by Olympus FV3000 RS Fluoview. Simulations were performed using the molecular dynamics package OpenMM version 7.0 (https://github.com/openmm/openmm) on the Folding@home distributed computing platform (https://foldingathome.org). Peptide residues were parameterized using AmberTools V17. Covalent hydrogen bonds were constrained by the LINCS algorithm. The MSMBuilder 3.8.0 software package (http://msmbuilder.org/3.8.0/) was used to construct MSMs. GMRQ cross-validation algorithm was used to decide the optimal number of states. The MDTraj package (https://mdtraj.org/1.9.3/installation.html) was used to compute the secondary structure content of simulation snapshots. All the custom code simulated for peptides are available @ (https://doi.org/10.5281/zenodo.5570769)
Data analysis	Most experimental assay data were analyzed and plotted by Graphpad Prism V6.0. NMR and MS spectra were analyzed by MestreNova V14.2. Confocal images were processed by NIH ImageJ software.

For manuscripts utilizing custom algorithms or software that are central to the research but not yet described in published literature, software must be made available to editors and reviewers. We strongly encourage code deposition in a community repository (e.g. GitHub). See the Nature Portfolio guidelines for submitting code & software for further information.

Data

Policy information about availability of data

All manuscripts must include a data availability statement. This statement should provide the following information, where applicable:

- Accession codes, unique identifiers, or web links for publicly available datasets
- A description of any restrictions on data availability
- For clinical datasets or third party data, please ensure that the statement adheres to our policy

The data supporting the results and findings of this study are available within the paper and the Supplementary Information files. Most raw data have been already included in the Supplementary Information. Additional raw data that support the plots within this paper are available from the corresponding author upon reasonable request. All simulated structure files and trajectory data for peptides are available at https://doi.org/10.5281/zenodo.5570769.

Field-specific reporting

Please select the one below that is the best fit for your research. If you are not sure, read the appropriate sections before making your selection.

☒ Life sciences ☐ Behavioural & social sciences ☐ Ecological, evolutionary & environmental sciences

For a reference copy of the document with all sections, see nature.com/documents/nr-reporting-summary-flat.pdf

Life sciences study design

All studies must disclose on these points even when the disclosure is negative.

Sample size	Sample size for the confocal imaging experiments have n >/= 100 cells per treatment group which was based on typical ranges used for this type of work and the published imaging studies and will afford statistically significant cell imaging results. Sample size for all other bio assays have n = 3 independent biological samples. Uncertainty is reported as SE or SD.
Data exclusions	No data were excluded from analysis.
Replication	All experiments were replicated at least three times in an independent manner and were successful.
Randomization	The n >/= 100 cells in each imaging study group were randomly selected. Randomization was not possible for other types of experiments included in this work.
Blinding	For all the biological assays included in this work, all the samples were treated in the same way, and therefore blinding is not relevant.

Reporting for specific materials, systems and methods

We require information from authors about some types of materials, experimental systems and methods used in many studies. Here, indicate whether each material, system or method listed is relevant to your study. If you are not sure if a list item applies to your research, read the appropriate section before selecting a response.

Materials & experimental systems

n/a	Involved in the study	
☐	☒	Antibodies
☐	☒	Eukaryotic cell lines
☒	☐	Palaeontology and archaeology
☒	☐	Animals and other organisms
☒	☐	Human research participants
☒	☐	Clinical data
☒	☐	Dual use research of concern

Methods

n/a	Involved in the study	
☒	☐	ChIP-seq
☒	☐	Flow cytometry
☒	☐	MRI-based neuroimaging

Antibodies

Antibodies used	Anti-FITC (HRP conjugated, 1:500 dilution, Abcam, catalog# ab196968); Anti-p53 (1:250 dilution, Enzo Life Sciences, catalog# ADI-960-070); Anti-HDM2 (biotin conjugated, 1:250 dilution, Enzo Life Sciences, catalog #ADI-960-070)
Validation	All antibodies have been validated by the manufacturer, in other publications, or in house using established protocols. Specifically for the ELISA based assays in this work, the presence or absence of the ELISA signals in the sandwich assay with or without the targeted antigens were used for validation.

Eukaryotic cell lines

Policy information about cell lines

Cell line source(s)	All cell lines were obtained from ATCC. They were HEK293T, DLD-1, HCT-116 p53 +/-, Jurkat, HeLa cells.
Authentication	The cultured cell lines have been routinely tested (once every two months) for viability, mycoplasma, and assayed by short tandem repeat (STR) profiling.
Mycoplasma contamination	All cell lines have been tested routinely and no contamination has been detected.
Commonly misidentified lines (See ICLAC register)	No commonly misidentified cell lines were used in the study.
